# Hierarchical Bayesian Regression for experimental psychology: a case study of cognitive control

**DOI:** 10.3389/fpsyg.2026.1643463

**Published:** 2026-03-19

**Authors:** Thomas A. Dudey, Joshua J. Jackson, Shelly R. Cooper, Todd S. Braver

**Affiliations:** Department of Psychological and Brain Sciences, Washington University in St. Louis, St. Louis, MO, United States

**Keywords:** cognitive control, dual mechanisms of control (DMC), Hierarchical Bayesian Regression, hierarchical models, proactive control, reactive control, sequential updating

## Abstract

Arising from the so-called ‘replication crisis’ in the experimental psychology literature, there has been a growing call to reassess whether specific analytic practices might enhance the accuracy and precision of reported findings. This issue is explored here, through a case study examination of two previously collected datasets from the Dual Mechanisms of Cognitive Control (DMCC) task battery. This case study highlights the unique advantages afforded by Hierarchical Bayesian Regression (HBR) models as a potentially more rigorous analytic approach to statistical inference. In the DMCC datasets, two sets of HBR models are presented, with the estimates of the former used as priors for the latter. In addition to systematically generating cumulative posterior distributions for all effects of theoretical interest, we further illustrate how our particular application of HBR models provides novel insights regarding specific indicators of proactive/reactive control in each of the four DMCC tasks, by: (1) estimating the consistency of effects across datasets; (2) estimating the relative strength of null effects; (3) accurately modeling the specific properties of response time distributions; and (4) appropriately modeling accuracy patterns at the trial level.

## Introduction

Over the past decade, the field of experimental psychology has grappled with the discovery that a startling proportion of highly influential findings do not hold up under increased scrutiny. One large-scale project found that among 100 initial psychology experiments with mostly significant results, over half of them no longer met the conventional significance criterion (*p* < 0.05) and had diminished effect sizes after undergoing well-powered replications (Open Science Foundation, 2015). This ‘replication crisis’ may seem like a significant step backwards in our current knowledge of psychological phenomena. Alternatively, however, the present circumstance could be perceived as an opportunity from which to reassess the analytic practices that best ensure the validity and robustness of a given finding. It is becoming increasingly appreciated that even though *t*-tests and ANOVAs are some of the most frequently used approaches in the psychological literature, due to the relative ease in which they can be computed, they are ultimately limited in terms of the statistical inferences they enable (Lindley, 1957; Wacholder et al., 2004; Ioannidis, 2005; Erceg-Hurn and Mirosevich, 2008; Zhou and Skidmore, 2017).

Two sets of critiques have been leveled against the prevalent adoption of such conventional statistical analysis methods. First, their validity is contingent upon a set of underlying assumptions regarding the observed data (e.g., normality and independence). As a result, the systematic violation of these assumptions will contribute to the inflation of false positive and negative rates within the field (Erceg-Hurn and Mirosevich, 2008; Zhou and Skidmore, 2017). In response to this issue, hierarchical models^1^ assuming non-Gaussian distributions (i.e., generalized hierarchical models) have arisen as an alternative analytic tool that can more accurately and fully model the properties of the data (Singmann and Kellen, 2019; Meteyard and Davies, 2020; Brown, 2021).

A second and more general concern regarding both conventional and more contemporary statistical approaches is that they are situated within the frequentist framework, which has its own set of inherent limitations (O’Hagan, 2008). Besides relying on isolated samples to make inferences about the entire population, they also do not enable estimation of the relative probability that the alternative or null hypothesis is true (see Greenland et al., 2016; Wagenmakers et al., 2018; Rouder et al., 2018, for extensive discussions about the limitations of NHST). Due to such problems, there has been another emerging movement within the field to transition toward the more intuitive and flexible Bayesian framework, which not only allows for the incorporation of prior information outside of the available data to generate more precise findings, but also enables direct probabilistic estimates regarding specific parameters and hypotheses of interest.

The benefits afforded by generalized hierarchical models and the Bayesian framework are not mutually exclusive. Indeed, the broad goal of this paper is to describe how the intersection of these methods through Hierarchical Bayesian Regression (HBR) modeling can provide a comprehensive and rigorous analytic approach that addresses the limitations of previous methods (Rouder and Lu, 2005; Nalborczyk et al., 2019; Veenman et al., 2024). Although HBR is more computationally intensive than standard approaches, due to the increased complexity of its inferential algorithms, the goal here is to illustrate the manifold advantages of the HBR approach through concrete examples. In particular, we provide an illustrative case study example utilizing two datasets (N > 100), that were previously acquired to validate the online task battery for the Dual Mechanisms of Cognitive Control (DMCC) project described below.

The Dual Mechanisms of Cognitive Control (DMC) framework provides a neurobiologically-based, mechanistic account of cognitive control that postulates two qualitatively distinct modes—proactive and reactive. Proactive control is characterized as a sustained and anticipatory mode of cognitive control that actively maintains goal-related information, while reactive control reflects a more transient mode that retrieves goal-related information in response to conflict (Braver, 2012). In addition to individual- and group-level differences influencing the tendency or ability to adopt one control mode versus the other, careful experimental manipulations have revealed contextual or situational factors that can elicit intra-individual modulations in how the control modes are deployed (Braver, 2012; Gonthier et al., 2016a, 2016b). Based on these findings, the Dual Mechanisms of Cognitive Control (DMCC) project was initiated by our group to develop and validate paradigms that would reliably produce within-subject shifts in cognitive control settings across different conditions (Braver et al., 2021).

The DMCC project involved the development and validation of a new cognitive control task battery, including baseline, proactive, and reactive variants for each of four different experimental paradigms, reflecting distinct cognitive domains: the AX-CPT for context processing, the Sternberg WM for working memory, the Stroop task for selective attention, and the Cued-TS paradigm for multi-tasking. While the baseline versions for each task were constructed to maximize variability in the cognitive control mode utilized, the proactive and reactive versions were manipulated to bias participants toward each specific mode of control. One aim of the DMCC project was to demonstrate the task battery’s replicability and generalizability, at least in terms of behavioral metrics. As such, each task and its variants were assessed via multiple behavioral indicators of proactive or reactive control that were compared across conditions to identify general indices of cognitive control (i.e., proactive and reactive versus baseline conditions), as well as double dissociations in control modes (i.e., proactive versus reactive conditions).

Tang et al. (2023) provided the first systematic validation of the online DMCC task battery, with an initial dataset collected in 2018. However, the analysis approach utilized conventional NHST methods, with paired *t*-tests used to identify within- and between-condition effects. Since that time, an additional sample was also collected [in 2020], with participants undergoing a nearly identical protocol, to test the generalizability of the findings. Here, we examine both datasets in relation to each other, with one goal being to replicate and extend the results of Tang et al. (2023), using an alternative HBR framework for analyses^2^. Yet a potentially more primary goal was to highlight the various advantageous features of the HBR framework, as detailed further below.

A primary advantage of HBR models is the use of Bayesian frameworks for statistical inference. In parameter estimation, both frequentist and Bayesian regression models can compute point estimates with surrounding intervals to determine the influence of predictors upon an outcome variable. In Bayesian models, however, these estimates are directly constructed as probability distributions, through Bayes Theorem. By utilizing both initial prior beliefs about parameter estimates, P(θ) , and the likelihood of the data, P(data∣θ), it is possible to compute posterior distributions, P(θ∣data) . These derived posteriors inherently offer a richer understanding of each estimate, providing the relative certainty for its possible values, versus a single most likely value and its standard error. Critically, these distributions can be continually updated with new information, through a sequential learning procedure (i.e., previous posteriors serving as priors for the analysis of subsequent datasets) which ultimately reduces uncertainty regarding possible values as well as enables more precise and valid predictions to be made toward future observations (Wagenmakers et al., 2016).

In the analyses of the DMCC datasets, we utilized this sequential updating procedure to inform our understanding regarding the degree of replicability and consistency in key effects of interest. In particular, two types of “objective priors” were employed in a complementary manner across the two datasets (see Berger et al., 2015; Goldstein, 2006; Efron, 2012; Torsen, 2015, for debates between the usage of “objective” versus “subjective” priors). The first set of priors were noninformative/weak, which minimize the degree to which the prior influences the posterior distribution. The use of noninformative/weak priors will cause the computed parameters to closely match with the central values estimated by data-driven approaches like frequentist maximum likelihood estimation (MLE) (Dunson, 2001; O’Hagan, 2008). Consequently, we used noninformative/weak priors as a conservative choice for our re-analysis of the 2018 DMCC dataset, to better compare with the findings reported by Tang et al. (2023). However, it is often the case that researchers *do* have some degree of information that should be used to shape predictions regarding the most likely estimates of particular phenomena (Lindley, 2004; Wagenmakers et al., 2018). Indeed, this was the case for the current study, as we were able to make use of the posterior estimates derived from the 2018 DMCC dataset as data-based priors in analyses of the 2020 dataset. This approach optimizes the available data to generate posterior distributions with relatively narrow credible intervals; such distributions represent the accumulated state of current knowledge regarding behavioral indicators of proactive and reactive control.

A related advantage of the Bayesian framework is that it enables more diverse hypothesis tests to be conducted to investigate specific hypotheses. With the replication crisis as an underlying problem that shapes the goals of this project, one important hypothesis of interest is to formally test how well new results align with previous findings on the same phenomenon. Indeed, one of the main reasons behind our choice to implement a sequential updating procedure was to simultaneously generate posterior estimates as well as to provide a more nuanced test of replication across datasets, in terms of the relationship between prior and posterior distributions. In particular, the Savage-Dickey Ratio (SDR) metric represents an approximation of Bayes Factor (BF) to test the strength of specific alternative hypotheses, by computing the ratio of the posterior and prior distributions at a selected point value. For our purposes, this value was chosen at the mean of the prior as a form of meta-analysis (Wagenmakers et al., 2010; Verhagen and Wagenmakers, 2014; Ly et al., 2019; Lin et al., 2024). With this approach, an SDR value greater than one indicates an increased probability in the original estimate with the addition of new information (i.e., there is inter-sample reliability in the magnitude and direction of the given effect), whereas an SDR value of less than one instead suggests that this additional data caused a notable shift away from the original estimate and toward a new more probable one (i.e., there is inter-sample variability regarding the quantitative properties of the given effect). In cases where theoretical predictions of the DMCC task battery have been strongly validated during initial analyses, this approach addresses the goal of determining whether key DMCC task manipulations induce estimates of proactive and reactive control that are reliable and consistent, or instead vary substantially, across datasets and participant samples.

Another unique aspect of the Bayesian framework is that it can be used to test the strength of evidence both in favor and against a hypothesis of interest. First, probability of direction (pd) scores and highest density intervals (HDIs) can be employed in a manner that is analogous to *p*-values and confidence intervals respectively, but they offer more interpretable assessments of significance than their frequentist counterparts, via an empirical posterior distribution versus a hypothetical null distribution (Makowski et al., 2019). While pd. scores reference the proportion of the posterior with the same sign as the median, HDIs are the most probable values that make up a certain density within the posterior^3^. Conversely, Bayesian hypothesis tests can also be employed to assess whether there is instead evidence in favor of the null hypothesis (Wagenmakers et al., 2018). There are several ways to perform hypothesis testing for null effects. One approach that has been most frequently employed in the psychological research literature is a Bayes Factor (BF) model comparison approach that directly compares the predictive accuracy of two models: an alternative hypothesis model (*M_1_*) that contains the parameter of interest, and a null hypothesis model (*M_0_*) that does not. This approach provides a direct quantification of the strength of one hypothesis/model versus the other (Schmalz et al., 2023).

Yet it is also possible to utilize a complementary approach within a Bayesian framework to evaluate null effects. After estimating the HDI, we can easily assess the degree to which this interval falls inside or outside a region of practical equivalence (ROPE). Since ROPE is an interval around zero which represents a negligible effect (i.e., one of no interest), this assessment can check the proportion of parameter values within a given posterior that support or challenge the given null hypothesis. By using both Bayesian metrics in a convergent manner, more nuanced inferences are possible regarding the evidence for a null effect than is possible with NHST. Specifically, results can be divided into three possible outcomes: (1) strong evidence for a meaningful effect (i.e., BF_10_ > 10, or HDI is fully outside ROPE); (2) strong evidence for a result practically equivalent to zero (i.e., BF_10_ < 1/10, or HDI is fully inside ROPE); and (3) little or inconclusive evidence in either direction (i.e., 1/10 < BF_10_ < 10, or partial overlap of HDI and ROPE). By distinguishing between these latter two possibilities, it becomes easier to determine the most practical next step for future investigations. For instance, inconclusive evidence for an effect of interest may indicate that collecting more data is necessary to ascertain its presence, while strong evidence for a null effect would indicate that current task manipulations did not successfully induce it. More generally, the convergent use of both ROPE and BF model comparison methods offers the opportunity to provide fine-grained evidence for a strong, inconclusive or null effect (Kruschke, 2014; Rouder et al., 2018).

A broader advantage of the HBR approach is that it also incorporates all the benefits that accrue from generalized hierarchical modeling. While conventional methods like paired t-tests and repeated-measures ANOVAs require a first stage of data aggregation across trials for each participant and condition, hierarchical models enable the full use of trial-level data. Specifically, the hierarchical structuring of this trial-level data will produce more reliable estimates, by not only incorporating random intercepts and slopes to better model subject-level variability, but also by shrinking outlying estimates toward their average/fixed effects (Veenman et al., 2024). The application of these hierarchical models has become increasingly common within cognitive control research, such as in studies examining reliability and individual differences in Stroop task effects (Rouder and Haaf, 2019; Viviani et al., 2023b; Viviani et al., 2024). Moreover, generalized versions of these models can utilize various likelihood functions to better model data that do not follow a normal/Gaussian distribution.

Within cognitive science, the domain of response time (RT) analysis has been quite impacted by these advances in modeling approach. Specifically, RTs tend to follow a skewed, rather than symmetric, distribution (Ratcliff, 1979). Although there have been longstanding attempts to consider this factor by trimming or transforming the data, these approaches bring with them their own complications and limitations (Heathcote et al., 1991; Ratcliff, 1993; Lo and Andrews, 2015; Zhou and Krott, 2016; Schramm and Rouder N., 2019). As a result, there has been increased support for the use of generalized models that can directly model the properties of the RT data through a set of parameters that appropriately represent its distributional shape. Indeed, such models also make it possible to specify and compare results when assuming different distributions (i.e., likelihood functions) for the data. In analyses of the DMCC dataset, for cases in which it was useful to explore whether observed effects were related to the form of RT distribution, HBR models utilizing more skewed distributions (i.e., shifted log-normal, ex-Gaussian) were compared to examine whether these modeling choices would affect the results. More broadly, generalized hierarchical models offer the opportunity to implement likelihood functions that more accurately capture the true properties of the RT data, and as such, can increase confidence in the validity of HBR analysis results.

A parallel benefit arises with error rate data. In other words, the same benefits from generalized mixed effect models can be applied to binary outcomes, such as correct versus incorrect responses. While standard approaches operationalize these outcomes as proportions, this approach is sub-optimal relative to hierarchical logistic regression, even with the incorporation of available transformation methods (e.g., arcsine, empirical logit) (Jaeger, 2008; Dixon, 2008; Houpt and Bittner, 2018). First, logistic regression models provide better fits of the outcome data, by operationalizing the binary outcomes as probabilities and assuming a Bernoulli rather than Gaussian distribution. Furthermore, they can more appropriately capture any non-linear relationships between predictors and the dependent variable, via the ‘logit’ link function. Finally, logistic regression integrates well with hierarchical modeling, by allowing for predictors to vary both by subject and condition, through access to each person’s trial-level data. This information is otherwise lost in typical aggregation-based procedures for accuracy/error rate data.

The overarching goal of this paper is to illustrate the combined power and flexibility afforded by an HBR approach, relative to more conventional ones, through a set of case study examples that directly highlight such benefits, taken from representative analyses of the DMCC task battery. To provide a roadmap of the results, the key conceptual principle linked to each task is described next. First, within the context of the AX-CPT paradigm, we highlight the advantages of the sequential updating approach and use of SDR to test for replication patterns across the 2018 and 2020 datasets, focusing on the BX error interference effect as a general cognitive control index. We demonstrate the different conclusions drawn regarding the stability of this effect, when considering either the proactive or reactive control indicator. Second, within the context of the Sternberg working memory (WM) task, we apply tests of evidence in favor of the null hypothesis, focusing on the novel positive (NP) RT effect as an indicator of proactive control. We demonstrate how strong evidence for and against the null hypothesis can be obtained using both ROPE and BF approaches in a convergent manner. Third, within the context of the Stroop task, we examine how more precisely modeling RT distributions can impact statistical inference, focusing on the congruency cost as an indicator of proactive control. We demonstrate how theoretically aligned RT distributions, such as the shifted log-normal and ex-Gaussian, can provide stronger evidence for a reliable effect than standard Gaussian distributions, which are clearly inappropriate. Fourth, within the context of cued task-switching (Cued-TS), we examine how the use of hierarchical (i.e., trial-by-trial) modeling of error rate data can also impact statistical inference, focusing on the error task-rule congruency effect (TRCE) as an index of reactive control. We demonstrate how hierarchical logistic regression approaches more sensitively model effects near floor or ceiling, relative to conventional analyses of error rate. Together, these collective findings not only provide additional validation of the DMCC task battery across four key dimensions, beyond that established in Tang et al. (2023), but also potentially more importantly, demonstrate the inherent and wide-ranging advantages of the HBR modeling framework to the broader experimental psychology community.

## Methods

### Participants

The data comprise two participant samples, who each completed the online DMCC task battery. The samples were collected through the Amazon Mechanical Turk (MTurk) online platform, with 178 participants collected in 2018 and 185 collected in 2020. Participants were not excluded by age range^4^ for either the 2018 sample^5^ (21–64, M = 36.03, SD = 9.92, 104 F and 74 M) or the 2020 sample (18–77, M = 38.41, SD = 10.87, 91 F and 93 M and 1 ‘prefer not to say’). For each sample and task, only participants with complete datasets for the battery were retained for analysis (the 2018 and 2020 datasets, respectively, yielded the following sample sizes for AX-CPT: 132 and 125; Sternberg: 133 and 131; Stroop: 123 and 127; Cued-TS: 133 and 135).

### Design and procedure

Besides the different time points in which participants completed the study, there were additional differences between the 2018 and 2020 samples. First, while participants in the 2018 sample completed the DMCC task battery twice, when possible (i.e., finishing 15 sessions in the ‘test’ phase and 15 in the ‘retest’ phase), those in the 2020 sample went through the task battery only once. Tang et al. (2023) only used the ‘test’ phase data from the 2018 sample to control for potential practice effects. In contrast, we chose to include all of the available trial-level data for each participant in the 2018 dataset, given that one of the conceptual goals of this paper was to utilize all the data within and across samples, in order to generate cumulative posterior distributions based on the full breadth of our current knowledge regarding behavioral metrics of proactive and reactive control.

Another key difference between samples is the order in which participants completed their sessions: the 2018 sample completed the task battery in the Baseline-Reactive-Proactive order, while the 2020 sample completed it in the Baseline-Proactive-Reactive order to counter-balance potential carry-over order effects. Thus, when aggregated across the 2018 and 2020 datasets, analyses should provide a more comprehensive and robust estimate of differences between proactive and reactive control indicators that controls for session order. Conversely, any differences in estimates between the 2018 and 2020 datasets could be due to session order differences, although without additional data it would be impossible draw this inference conclusively.

All participants were expected to perform approximately 5 sessions per week, ensuring that the study would take 3–6 weeks to complete. Each session was 20–40 min long, except for a 1-h first session that included a Stroop practice session to validate vocal responses as well as a battery of demographic and self-report questionnaires. Completed sessions were examined for accuracy and compliance (see Tang et al., 2023). Subjects were discontinued from the study if they did not complete the sessions in a timely manner or did not comply with task instructions. The AX-CPT, Sternberg, and Cued-TS were programmed with in-house JavaScript code (available upon request at https://sites.wustl.edu/dualmechanisms/request-form/), while the Stroop task was programmed and delivered using Inquisit software, as it allowed for the collection of online vocal responses (also available at the above link).

### Tasks

**Figure 1:**
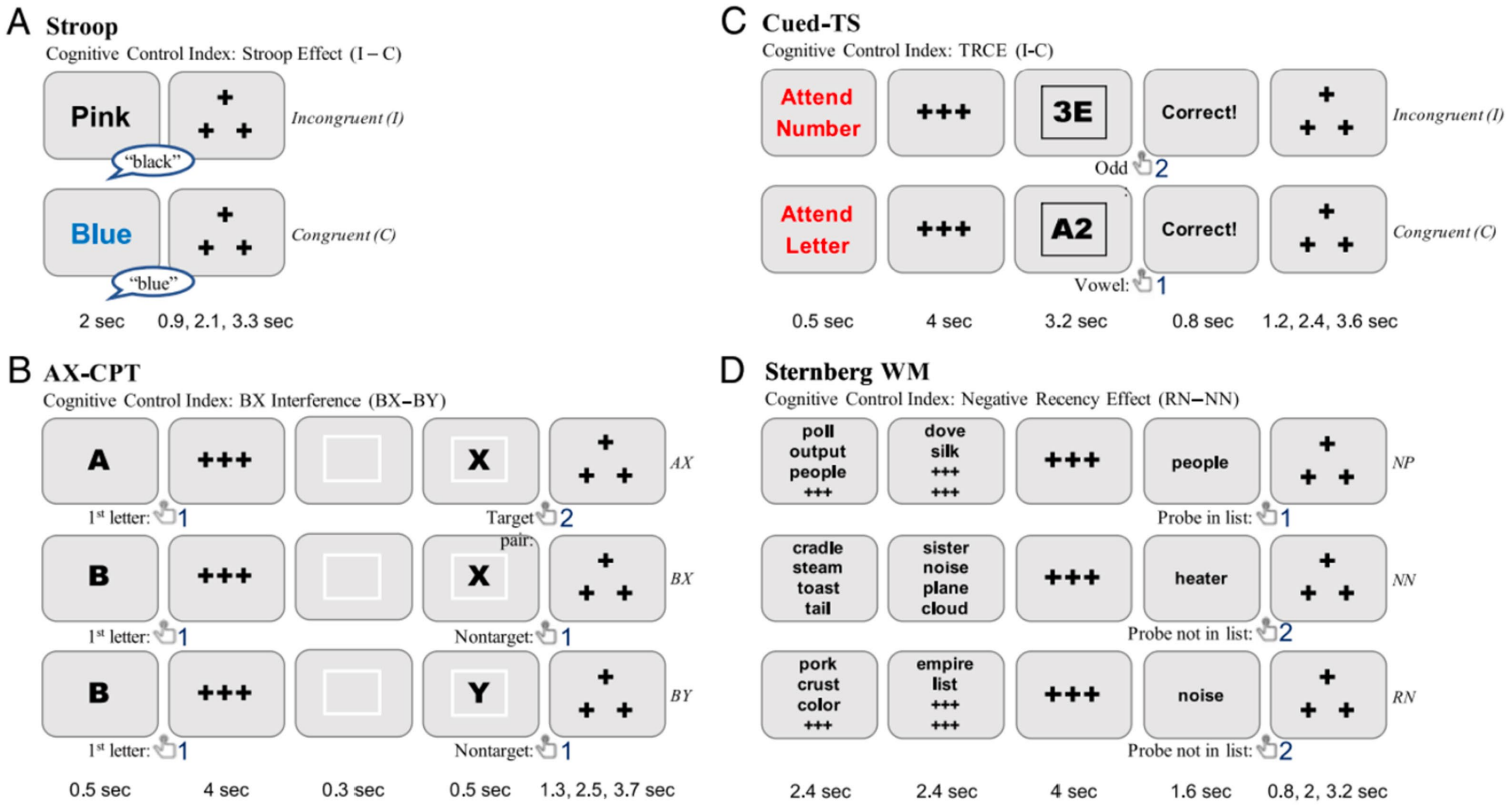
Illustration of the DMCC task battery.

As a detailed description of the task manipulations and theoretical rationale for the DMCC battery has already been provided in other companion papers (Braver et al., 2021; Tang et al., 2023; Snijder et al., 2023), here we provide only a brief description of each task paradigm and its variants below, along with a task illustration of the battery (Figure 1).

#### AX-CPT

In the AX-CPT, each trial includes a cue (i.e., A or B), a delay period, and a probe (i.e., X, Y, or number). When an “A” cue precedes the “X” probe, a target response should be made; however, the presence of a “B” cue (i.e., any cue letter besides “A”) and/or a “Y” probe (i.e., any letter besides “X”) indicates that a non-target response should instead be made; digit probes (i.e., 1–9) indicate no-go trials, for which participants were instructed to withhold their response. These no-go trials were included to reduce the inherent bias to fully prepare probe responses following the cue. The proactive and reactive versions of the task had specific modifications designed to induce each mode of control, respectively. The proactive variant explicitly instructed participants to prepare a target response after seeing an “A” cue and a non-target response after seeing a non-A cue; practice sessions and reminders to use the strategy were meant to reinforce the use of proactive control for this session. The reactive variant instead included a red border color at a unique location presented immediately before probe onset to indicate an upcoming high-conflict trial (i.e., AY, BX, no-go trials). The proactive version was designed to bias participants to engage and maintain cognitive control during the cue and delay period; instead, the reactive version included a specific probe-linked feature that alerted participants to actively retrieve the cue and abstract rule only at the time of probe presentation.

#### Sternberg

In the Sternberg task, participants had to determine whether a probe word was also present in the previous word list (memory set) on trials that varied possible working memory loads (i.e., the lists could contain 2–8 words, which were presented across two encoding displays). If the probe word was indeed part of the memory set, the trial would be categorized as a novel positive (NP) one, and a target response would be correct. On both novel negative (NN) and recent negative (RN) trials, the probe item was not part of the memory set for the current trial; however, the latter was characterized RN because the probe item *was* present in the memory set of the immediately preceding trial (i.e., was recently encoded into memory), thereby causing increased familiarity and false positives for RN versus NN trials. Both the number of items in a word list and the proportion of RN, NN, and NP trials were manipulated in different ways to induce proactive, reactive, or neither/baseline mode of control. In the proactive condition, most trials had low-load (2–4 items) memory sets, which biased participants to actively maintain these items as an effective strategy to bias attention toward the probe. In both the baseline and reactive conditions, most trials instead had high-load (6–8 items) memory sets, which biased participants to instead use a familiarity strategy to determine whether to make a target response to the probe item. In the reactive condition, however, the high frequency of RN trials would instead cause probe familiarity to serve as an alerting signal for full memory set retrieval. Finally, each condition included a matched set of 5-item lists that were treated as “critical items” and used for cross-condition comparisons.

#### Stroop

Although all versions of the color-word Stroop task utilized the classic contrast in performance between low-conflict congruent items (the font color and word match, e.g., BLUE in blue font) and high-conflict incongruent items (i.e., the font color and word do not, e.g., BLUE in red font), the proportion congruence (PC) within a task block was uniquely manipulated across conditions to induce different degrees of cognitive control use. A subset of diagnostic items that were equally likely to be congruent or incongruent (i.e., PC-50/diagnostic items) was included, so that directly matched comparisons could be made between conditions. Another subset of items was either mostly congruent or mostly incongruent (i.e., biased/inducer items), which distinguished the different conditions from one another. The baseline condition was designed to have high list-wide PC, in which congruent trials were relatively frequent and incongruent trials were rare, so that cognitive control demands would be relatively low throughout the task block. In contrast, the proactive variant was designed to have low list-wide PC which would bias participants to prospectively (i.e., before stimulus onset in a trial) utilize cognitive control throughout the task block, by attenuating attention toward the distracting ‘word’ dimension. In the reactive condition, the PC manipulation was item-specific such that the inducer items were mostly incongruent colors, while other filler items were always congruent; thus, the list-wide PC was matched to the baseline condition, but specific colors (inducer items) indicated high control demands. As a result, participants were expected use the color feature (i.e., detected after stimulus onset) as an indicator of whether cognitive control should be utilized (reactively) on that trial.

#### Cued-TS

All stimuli in this task consisted of letter-digit pairs (e.g., A-1, B-2); however, a task rule cued at the beginning of each trial indicated the relevant feature dimension to attend on that trial (i.e., attend to the letter to make a consonant/vowel discrimination, or attend to the number to make an odd/even discrimination). Two overlapping manual responses were used for each task; thus, the meaning of each manual response was dependent on the task rule, with stimuli that could either be congruent (i.e., the two task rules are associated with the same response) or incongruent (i.e., the two task rules are associated with different responses). The baseline version followed closely to this general task structure, with the task cues appearing in red font and the task stimuli appearing in black font. In contrast, within the proactive and reactive variants, a subset of incentive trials was included to bias participants toward one mode of control. The proactive version included reward incentive trials, pre-cued by the green font of the task rule, so that participants would engage in anticipatory cognitive control before stimuli onset, to make faster and more accurate responses. In contrast, the reactive version included punishment incentive trials (if an error was made), cued by the green font of the *target stimulus*, and primarily on incongruent trials, so that the processing of incongruence itself was associated with potential monetary loss. A matched set of non-incentivized trials were utilized for cross-condition comparisons. These non-incentivized trials were also biased to be mostly congruent, increasing the likelihood of conflict and interference on incongruent trials.

### Data preprocessing

To better accommodate hierarchical modeling, the preprocessing approach used here differed somewhat from that applied in Tang et al. (2023). However, preprocessing choices were also designed to be somewhat conservative, with the goal of ensuring that any different findings across the current analyses relative to Tang et al. (2023) would be driven by modeling rather than preprocessing choices. Datasets were first preprocessed by removing trials that contained outlying response times (RTs). Outlier determination was first set in a task-based manner, relative to the task RT distribution. In particular, relative to Tang et al. (2023), more restrictive cutoffs were chosen for tasks in which some trials showed clear discontinuities from the respective RT distribution, whereas more relaxed cutoffs were used in tasks for which the trials were clearly continuous with the RT distribution. Based on these criteria, the upper limit cutoffs were as follows: RT < 2000 ms for AX-CPT and Sternberg, RT < 5,000 ms for Stroop; RT < 6,000 ms for Cued-TS. The lower limit cutoff was the same for all tasks: RT > 200 milliseconds ms. Additional subject-level thresholds (i.e., RTs that were 3 sd’s above/below an individual’s mean RT) were then put in place to ensure that individual RT distributions did not include outlying observations in respect to the participants’ own performance patterns within a given task. Together, the percentage of removed trials for the 2018 and 2020 DMCC datasets for each task were AX-CPT: 3.7, 3%; Sternberg: 3.8, 4.2%; Stroop: 2.6, 4%; Cued-TS: 1.9, 1.9%.

In addition to excluding individual trials, entire participants were removed if they exhibited unusual performance patterns for a given task, whether it was due to missing data, obvious performance-related deficits as specified by Snijder et al. (2023), or inconsistent/ stereotypical response patterns^6^. More specifically, participants were removed if they: (1) had an incomplete number of trials/sessions within a task; (2) were missing more than 50% of correct responses after all trial-level filtering criteria were applied; (3) did not respond to more than 40% of trials requiring a response; (4) had incorrect responses for more than 40% of trials; (5) had more than 40% of responses faster than the task-specific minimum threshold; (6) had more than 20% of responses slower than the task-specific maximum threshold; (7) had conditional RT data forming mixed distributions with multiple peaks (i.e., inconsistent responses); and (8) showed systematic repeating or alternating responses independent of the stimulus content (i.e., stereotypical responses). Finally, participants completing the task battery on a MacOS rather than Windows operating system were removed from both Stroop datasets since the Mac platform had compatibility issues with Inquisit, thus leading to systematically slower RTs. Following these outlier exclusions, the derived 2018 and 2020 DMCC datasets, respectively, contained the following number of participants for AX-CPT: 132; 123; Sternberg: 139, 126; Stroop: 126, 114; Cued-TS: 135, 126.

### Data analysis

Hierarchical Bayesian regression (HBR) models were fit using the brms package in the R software environment (Bürkner, 2017). Logistic models were applied to trial-level data in analyzing models with a binary outcome variable (i.e., target versus non-target responses, or correct versus incorrect responses). Models with response time as the dependent variable were primarily analyzed with shifted log-normal functions on trial-level data. However, we also fit ex-Gaussian functions in cases where it was important to capture the skewed properties of the RT data yet preserve its original units (ms). Additionally, we fit Gaussian (and ex-Gaussian) distributions in cases for which we wished to directly compare the predictive accuracy of each distribution (these cases will be described in the Results section). The categorical predictor ‘mode’ (Baseline, Proactive and Reactive sessions) was dummy coded to ensure direct comparisons between conditions on the outcome variables of interest; other relevant subject-level variables were also dummy coded to operationalize these behavioral indicators of proactive or reactive control. The specific models for each task are included in both the Results and Supplementary material Results sections. While implementable Wilkinson notation is provided in both sections, fully indexed notation is also included in the Supplementary material.

The hierarchical nature of the models was accommodated by including random intercepts and slopes. Since the inclusion of these parameters substantially increases the number of parameters that must be estimated, and consequently slows the runtime of each model, only difficulty [∆] *μ* parameters besides ‘mode’ were systematically added as both fixed and random effects (e.g., intercept, congruency, item type and trial type). This choice reflects the prioritization of modeling intrinsic subject-level differences within the reference condition (i.e., ‘baseline’ performance unrelated to targeted DMCC experimental manipulations). For similar reasons, distribution-specific parameters for different likelihood functions (e.g., the shift parameter θ within a shifted log-normal distribution, the exponential decay parameter τ within the ex-Gaussian distribution, and the scale parameter σ within all models) were fixed across condition and subject.

The posterior distributions for each relevant parameter were iteratively generated using Markov chain Monte Carlo (MCMC) chains. The number of iterations across chains differed by task and sample, but the validity of each HBR model output was confirmed by utilizing diagnostic metrics of appropriate chain mixing (i.e., Rhat < 1.05), sufficient sampling across the parameter space (i.e., Bulk and Tail ESS > 400), and posterior predictive checks that simulated predictions from the model output and compared their estimations to the observed data. Besides running more iterations for either model convergence or reliable marginal likelihood estimates (typically 40,000 iterations), the re-specification of adapt_delta (from default 0.8 to 0.9–0.99) and max_treedepth (from default 10 to 12–15) arguments can ensure tractable MCMC sampling of the data (Stan Development Team, 2025). Considering the novel utilization of these models for the online DMCC task battery, the 2018 HBR models used noninformative default priors for the fixed effects as well as weakly informative default priors for the random effects. In contrast, the 2020 HBR models constructed the priors based on the fixed effects of the posterior distributions generated by their 2018 counterparts, specifically utilizing informed Gaussian prior distributions shaped by the mean and standard error of the 2018 parameter estimates.

For each parameter estimate across both sets of HBR models, we report the mean, standard error, the 95% highest density interval (HDI), and probability of direction (pd) values (scores greater than 97.5% were considered strong evidence for an effect) (Kelter, 2020). Additionally, we utilized SDR scores to implement point estimate hypothesis testing for the parameter of interest in each 2020 HBR model, specifically to determine if their prior and posterior distributions had sufficient overlap (SDR > 1), versus a significant shift in the posterior from the original estimate (SDR < 1). Visualizations of the relationships between the prior and posterior distributions are included for all measures of interest.

The Bayesian hypothesis testing metrics, ROPE and BF model comparisons, were additionally utilized in cases for which we wished to test evidence in favor of the null hypothesis. With ROPE, the HDI fully outside of the ROPE would be strong evidence for the alternative hypothesis, the HDI fully inside the ROPE would be strong evidence for the null hypothesis, and the HDI having partial overlap with ROPE would be inconclusive evidence for either hypothesis. Both default 89% and conservative 95% HDI intervals were utilized to compare their respective degree of overlap with the prespecified ROPE range (Kruschke, 2014, 2018; McElreath, R., 2018). For BF model comparison in which the alternative hypothesis (*M*_1_) is the numerator and the null hypothesis (*M*_0_) is the denominator (BF_10_), BF_10_ > 10 is standardly treated as strong evidence for the alternative hypothesis, whereas BF_10_ < 1/10 is strong evidence for the null hypothesis; 1/10 < BF_10_ < 10 is treated as inconclusive evidence for either hypothesis. Inversely, BF_01_ represents the case where *M*_0_ is now the numerator, and *M*_1_ has become the denominator.

Importantly, leave-one-out cross-validation (LOO-CV) approaches can be used alongside BF model comparison to provide converging evidence for the relative impact of various modeling choices (e.g., likelihood functions, the inclusion of a main/interaction term) on predictive accuracy. Although LOO-CV is distinct from ROPE/BF, as it does not explicitly quantify evidence in favor of a null effect, its conclusions are less affected by prior specifications, thus serving as a valuable sensitivity check when conducting any model comparisons (Lartillot, 2023). When the absolute difference in the expected log pointwise predictive density (|∆elpd_LOO_|) estimator is greater than four and two-times higher than its computed standard deviation (∆SE_LOO_), the most accurate model would be considered a substantially better fit than its competitor(s) (Stan Development Team, 2025). In contrast, either |∆ elpd_LOO_| < 4 or |∆ elpd_LOO_| < ∆SE_LOO_ x 2 would indicate that there is no meaningful difference in predictive accuracy between models. Finally, posterior predictive checks (PPCs) were additionally used to provide visualizations of how well models with different likelihood functions fit onto the actual data. Data and code for all analyses are publicly available on the OSF platform (https://osf.io/bj6fy/).

## Results

Given the case study nature of this project, we focus the results on a subset of analyses conducted with the two DMCC datasets that best illustrate the impact of HBR analyses on findings and interpretations. These involved a specific metric within each of the four tasks in the DMCC battery (AX-CPT: BX error interference effect; Sternberg: NP RT effect; Stroop: congruency cost; and Cued-TS: TRCE error effect). Since these analyses entail dissociations across different levels of our predictors (e.g., proactive versus reactive or baseline, between specific trial/item types), we chose to fit separate models onto specific subsets of data within each DMCC task. Arguably, a more powerful approach is to fit a single model to the entire DMCC dataset and subsequently extract relevant contrasts via posterior linear combinations; however, we felt that fitting separate models better aligned with our various HBR modeling applications and expository goals. Nevertheless, in the Supplementary material Results section, we implemented this alternative method to assess the impact of this modeling choice; any place where this choice might have impacted the interpretation of the primary results was also included in the manuscript. Furthermore, our Supplementary material Results provide a comprehensive set of analyses that address the complete findings and theoretical predictions tested in Tang et al. (2023), as well as descriptive statistics for each sample, and when all data are aggregated together. These comprehensive analyses almost fully replicated the patterns in Tang et al. (2023), except where discussed below.

### Estimating replicability with HBR models: AX-CPT

#### Background

Within the AX-CPT, a primary focus of theoretical interest is the BX error interference effect, an index of cognitive control which reflects the ability to appropriately utilize contextual cue information to correctly bias nontarget responses to the probe. In particular, the BX error interference effect occurs due to the relative difficulty of high-conflict BX trials (i.e., the same probe combined with an “A” cue would have required a target response) versus low-conflict BY trials (i.e., neither the probe nor the cue indicate a target response). However, the deployment of either proactive or reactive control is theoretically predicted to lead to reduced BX error interference relative to the Baseline condition; this metric should serve as a general marker of cognitive control. From this prediction, we used the HBR modeling approach as a tool to explore the consistency of predicted effects across datasets, specifically examining the magnitude of BX error interference in Reactive and Proactive conditions across the DMCC 2018 and 2020 datasets.

#### Analysis and results

In the HBR analysis, the BX interference effect was estimated as the log odds of making an incorrect response for BX versus BY trials, with the following model run on BX/BY trial-level data: *probeCorrect ~ 1 + trial type x mode + (1 + trial type x mode | ID)*^7^. The outcome variable ‘probeCorrect’ was binary coded so that the model predicts the log odds of an error (i.e., correct = 1; incorrect = 0). The categorical variable ‘trial type’ was dummy coded, with BY trials as the reference category to estimate relative BX interference. The categorical variable ‘mode’ was dummy coded with Baseline as the reference category to compare how the Proactive and Reactive modes affected this estimate; a separate HBR model was run to compare each mode of control to Baseline. The interaction effect between trial type and mode (i.e., trial type x mode) was the key parameter of interest to examine the difference in BX error interference between modes. This interaction term was also treated as a random effect within each subject to more fully account for trial-level variability.

**Figure 2:**
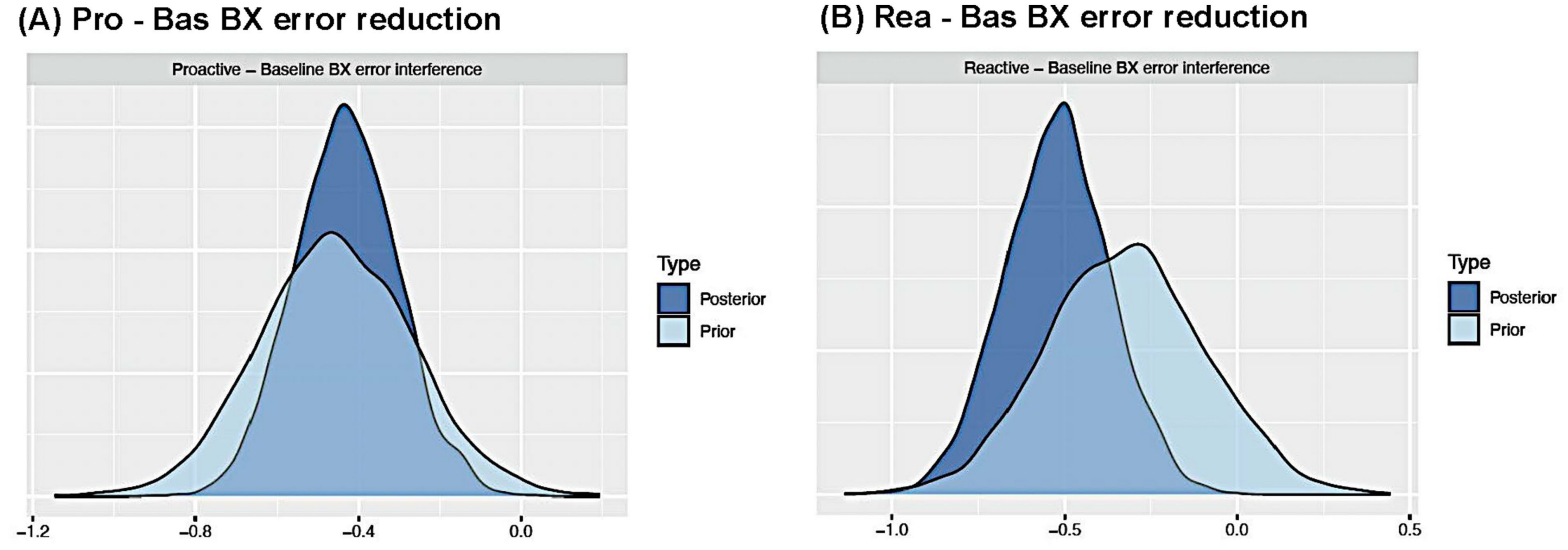
AX-CPT analyses, focused on inter-sample reliability. **(A)** Posterior and prior distributions for the Pro – Bas BX error interference reduction effect. The posterior overlaps with the prior, indicating high inter-sample consistency in the estimated parameter. **(B)** Posterior and prior distributions for the Rea – Bas BX error interference reduction effect. The posterior shifts away from the prior and zero, indicating the prior underestimates this effect.

In line with theoretical predictions and previous results from Tang et al. (2023), the 2020 HBR models indicated strong evidence for a reduction in the BX error interference effect in both the Proactive ( β = − 0.43, se = 0.13, 95% HDI = [−0.70, −0.20], pd. = 99.97%) and Reactive modes ( β = − 0.52, se = 0.15, 95% HDI = [−0.82, −0.22], pd. = 99.96%) relative to Baseline. Our key focus of interest was to compare the prior and posterior distributions for both models of BX error interference reduction (Figures 2A,B). The posteriors of this measure had higher peaks and narrower distributions relative to their priors, signifying that the addition of new data reduced uncertainty in the estimate of this parameter.

Interestingly, there was a notable difference in the SDR results across the Proactive and Reactive BX error reduction models. The SDR of the Proactive versus Baseline BX error interference estimate was greater than one (1.44; Figure 2A), which indicated a substantial overlap between the prior and posterior distributions. This result can be interpreted as evidence that the estimate of the BX error interference effect reduction in Proactive was quite consistent across the 2018 and 2020 DMCC datasets. By contrast, the SDR of the Reactive versus Baseline BX error interference estimate was clearly smaller than one (0.66; Figure 2B), which indicates that the parameter estimate from the 2018 dataset underestimated the value obtained when both 2018 and 2020 datasets were included. Furthermore, the pd. was less than 97.5% in the 2018 HBR model (pd = 92.1%) but 100% in the 2020 one. The SDR findings that compare the updated posterior with its original prior highlight how accumulating more data enables a direct test of consistency. In this case, we demonstrate that the reactive BX error interference effect can still be treated as a general indicator of cognitive control engagement; yet at least for the reactive condition, the results point to a lack of consistency in the presence and magnitude of the effect across our two datasets.

#### Summary

The BX error interference effects from the AX-CPT illustrate how the sequential learning process inherent in a Bayesian framework can provide a cumulative quantification of the most likely values for a parameter of interest. Specifically, we demonstrate both a case where the accumulation of additional data increased our confidence in the initial estimate, as well as a case in which our confidence shifted from the initial estimate toward a new more likely one. Specifically, the magnitude of the Proactive BX interference effect was stable across both datasets, signaling that the Proactive variant generated a reduction in BX interference that was highly consistent across two dataset samples. By contrast, the magnitude of the Reactive BX interference effect was found to vary substantially across the datasets. Thus, one future direction would be to explore the reason(s) behind this discrepancy in the consistency of the Proactive and Reactive BX interference effects. The increased certainty in the original Proactive BX error interference estimates across these two datasets suggests that the effect should successfully generalize to new samples and contexts. Conversely, the contrasting magnitude of the Reactive BX interference effects could be due to either a lack of replicability or of generalizability. Interestingly, one meaningful difference between the two datasets was the order of experimental conditions. Within the 2018 datasets, the Reactive condition was last, and after the Proactive condition, whereas in the 2020 dataset, the Reactive condition was performed before Proactive and immediately after Baseline. Consequently, it would be ideal to collect future datasets with the same or different condition orders to see whether this factor might serve as a significant moderating variable on Reactive BX error interference. More generally, the sequential updating approach implemented in HBR can be useful in providing clues regarding when it might be profitable to conduct additional replication studies, and further how these might be conducted, in terms of the key factors to examine.

### Evidence in favor of the null hypothesis: Sternberg

#### Background

In the Sternberg task, the novel positive (NP) effect in reaction times (RT) is of theoretical interest as a potential index of proactive control. The effect is defined as the difference in response time to make correct responses for NP trials across control modes. While low-load and high-load memory sets were used to bias participants toward or away from proactive control, each condition also included an equal number of critical trials (i.e., those with 5 items per memory set) that could be used to make direct comparisons across conditions. The key theoretical prediction was that response decisions to probe items through proactive control would be directly based on active and accessible WM representations (i.e., in the focus of attention), which would enable such decisions to be made more quickly than other conditions in which the probe item had to be elicited via a retrieval cue to reactivate information.

In Tang et al. (2023), the NP effect was found to be statistically significant, but with unclear reliability in the Proactive versus Baseline contrast, while the effect for the Proactive versus Reactive contrast was numerically in the correct direction but not statistically significant. Here, we relied upon a key feature of HBR methods to explore these effects further, by directly assessing the evidence for each contrast, regarding both the alternative (Proactive < Baseline NP RT) and null hypotheses (Proactive = Reactive NP RT) respectively.

#### Analysis and results

To specifically test for the relative evidence in favor of each hypothesis, a Bayes Factor (BF) model comparison approach was utilized, by running two types of models: one representing the null hypothesis, *RT ~ 1 + (1 | ID)*, and the other representing the alternative hypothesis, *RT ~ 1 + mode + (1 | ID)*. ‘Mode’ was dummy coded with Baseline and Reactive as the reference conditions to show NP performance in the Proactive mode relative to other conditions. Since BF model comparison was employed to determine if the fixed effect ‘mode’ had a strong impact on the NP RT results, we chose to not include ‘mode’ as a random effect for these HBR models; therefore, only the intercept was entered as a random effect nested within subject to account for trial-level variability. Additionally, we employed the ROPE method as an alternative approach to assess evidence for the null hypothesis, assuming that an RT difference within a range of 5 milliseconds would correspond to a negligible NP effect. Here we used an ex-Gaussian distribution to model RTs. As we discuss further for the Stroop task, the shifted log-normal distribution is also an excellent choice for modeling RTs; however, its transformation of the RT data into log-normal units makes it more challenging with regard to null hypothesis interpretability (i.e., which values should be used to define the ROPE). Both conventional 89% and conservative 95% HDI thresholds were used to assess whether this choice affects qualitative conclusions.

For this set of analyses, we focused on the 2020 HBR models which have the compounded benefits of incorporating informed priors to generate a cumulative posterior distribution, random effects to account for the hierarchical structure of the data, and an ex-Gaussian function to model its skewed properties. Even with these advantages, we did not find the NP RT effect to be a robust and general indicator of proactive control: although there were decisively faster responses for NP trials in Proactive versus Baseline ( β = − 19.92, se = 2.38, 95% HDI = [−24.57, −15.26], pd. = 100%), there was no evidence of an NP effect in the Proactive versus Reactive comparison ( β = 0.73, se = 2.25, 95% HDI = [−3.63, 5.19], pd. = 62.67%).

**Figure 3:**
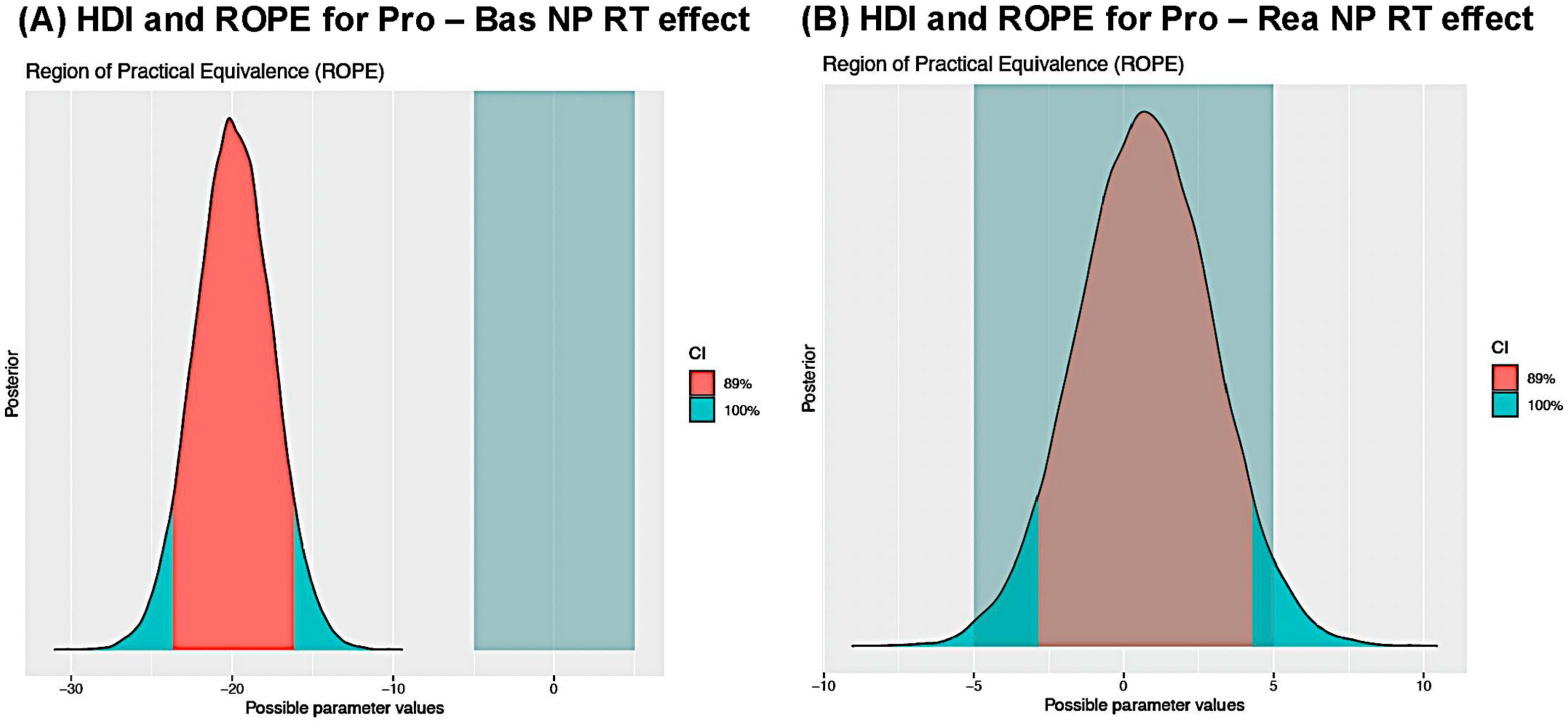
Sternberg WM analyses, focused on evaluating null effects. **(A)** The 89% highest density interval (HDI) of the posterior. The HDI is completely outside the pre-established region of practical equivalence (ROPE) range of −5 ms to 5 ms, indicating strong evidence for the significance of this effect. **(B)** The 89% HDI of the posterior. The HDI is completely within the ROPE range, indicating strong evidence for a null effect.

In both cases, ROPE and BF model comparison tests were performed to illustrate how these methods can either provide converging evidence for the alternative hypothesis in the presence of a strong effect, or how they can instead show relative evidence for the null hypothesis when an effect is essentially zero. For the Proactive versus Baseline NP effect, the BF model comparison approach indicated decisive evidence for the alternative hypothesis (*M_1_*), with a BF_10_ = 1.72 × 10^8^. In line with this result, the entirety of the 89 and 95% HDIs fell outside the ROPE region for this effect (see Figure 3A, for the 89% HDI result). In contrast, the Proactive versus Reactive NP effect exhibited strong evidence for the null hypothesis (*M*_0_), both in terms of the computed Bayes Factor, which had a BF_10_ = 0.038, and the 89% HDI falling completely inside the ROPE range (Figure 3B). However, follow-up analyses with the 95% HDI indicated that a small portion of the posterior estimate did fall outside the ROPE range (i.e., 0.43%). While this more conservative threshold would technically indicate there is inconclusive evidence for *M_0_* versus *M_1_*, the default variant of this ROPE procedure combined with BF model comparison overall indicated strong evidence favoring *M*_0_ over *M*_1_. Finally, a sensitivity analysis employing LOO-CV confirmed there was a meaningful Proactive – Baseline NP RT effect (∆_M1–M0_elpd_LOO_ = −20.4, ∆_M1–M0_SE_LOO_ = 4.8) but no evidence for a Proactive – Reactive effect (∆_M1–M0_elpd_LOO_ = −0.3, ∆_M1–M0_SE_LOO_ = 0.2). However, it is important to acknowledge that by itself, the LOO-CV metric does not directly quantify evidence for *M*_0_ in the latter case. Nevertheless, when considered together, the collective results demonstrate how the Bayesian framework provides various hypothesis testing methods, which can be used in a convergent manner to determine the degree of evidence favoring specific alternative or null hypotheses.

#### Summary

The HBR analyses strengthened both conclusions reported in Tang et al. (2023) regarding the NP RT effect. For the Proactive versus Baseline contrast, the HBR analyses confirmed that there was indeed very strong evidence for the reliability of the effect, both in terms of BF and ROPE methods. However, regarding the more theoretically and methodologically critical Proactive versus Reactive contrast, the HBR analyses even more strongly confirmed the presence of a null effect. The equivalence of Proactive and Reactive NP performance was already suggested in Tang et al. (2023), not only with RT patterns, but also with the results on NP error rates, which were in the opposite direction of theoretical predictions (and this pattern was confirmed with the current HBR analyses; see Supplementary materials). Yet, the hypothesis testing made possible through HBR analysis allowed the conclusion of a null effect in the Proactive versus Reactive comparison to be more strongly asserted beyond what was possible in Tang et al. (2023).

In contrast with the null hypothesis significance testing (NHST) framework, which permits only a binary assertion regarding whether the null hypothesis can or cannot be rejected, Bayesian hypothesis testing enables more fine-grained statements regarding the strength of evidence for both the null and alternative hypotheses. This advantageous property of Bayesian frameworks has enabled the development of heuristic (i.e., post-hoc) approaches from which to apply BF metrics, to data that were primarily analyzed with NHST approaches (e.g., as was done in Tang et al., 2023, which did report BF indices for the NP RT and error measures). Yet it is important to note that some researchers prefer the ROPE approach as a complementary and potentially stronger metric from which to identify effects that for all practical purposes can be treated as equivalent to zero. Estimation tests for ROPE are more directly aligned with the Bayesian rather than NHST framework (given that they rely upon generation of a full posterior distribution); yet because of this feature, they cannot be computed in a post-hoc, heuristic manner (Kruschke, 2014). Here, we took advantage of the fully-fledged HBR analyses, which enabled both BF and ROPE metrics to be computed.

Nevertheless, it is important to acknowledge both metrics still inherit some of the same limitations associated with NHST, in that they also rely upon categorical criteria to make somewhat broad inferences about a tested hypothesis. Furthermore, the derivations that underlie NHST and Bayesian metrics can lead to diverging conclusions in the case of large sample sizes, which will be biased toward the alternative or null hypothesis, respectively, (i.e., Lindley’s paradox) (Lindley, 1957). However, we still believe that, when utilized together, our specific application of ROPE and BF model comparison not only align with previous conclusions, but also provide more granular inferences than those allowed by NHST. More specifically, these metrics provided convergent, and thus stronger, evidence in favor of a null Proactive versus Reactive NP effect (i.e., both BF < < 1/10 and entirety of the [89%] HDI interval within the ROPE)^8^. In particular, the cumulative results across samples and analytic approaches permit the strong conclusion that, despite the goal of the DMCC project to validate a robust indicator of proactive control for the Sternberg task, this goal was not accomplished. Future work should reconsider task design alterations that have greater potential to elicit unambiguous behavioral markers of proactive control.

### Precise modeling of RT distributions: Stroop

#### Background

In the Stroop task, the congruency cost is of theoretical interest as a potential index of proactive control engagement. In particular, the deployment of proactive control is thought to bring not only performance benefits on high-demand incongruent trials, but also performance costs on congruent trials. This cost–benefit pattern should be most pertinent when contrasting Proactive and Reactive conditions, given that proactive control is postulated to involve preparatory mechanisms that enable biasing away from the ‘word’ dimension across all item types, whereas reactive control biasing is thought to occur only after detection of the relevant color feature and the presence of incongruence, so should not impact congruent items. Further, this key theoretical prediction is most stringently tested on diagnostic/PC-50 items.

Notably, the theoretical prediction regarding congruency cost was not confirmed in Tang et al. (2023). Specifically, in that analysis, the congruency cost was found to be statistically unreliable. However, the unreliability of the congruency cost effect reported in Tang et al. (2023) also contrasts with its observed robustness across multiple other companion papers (Gonthier et al., 2016a, 2016b; Bugg, 2017; Ileri-Tayar et al., 2025). Since the congruency cost is a small effect (~10 ms), making it potentially sensitive to both measurement error and incorrect assumptions regarding data structure, an important question arises on whether modeling the specific properties of the sample RT distribution impacts the identification of congruency cost effects, in terms of sensitivity and reliability. The flexibility of HBR models to assume both a hierarchical structure and non-Gaussian distribution of the data may therefore be a useful approach to elucidate the presence and strength of the congruency cost in these samples. We thus explored the robustness of the congruency cost metric as a function of the RT distribution employed in the analysis.

#### Analysis and results

To evaluate the congruency cost, the RT data were first modeled using a shifted log-normal distribution to test for differences between the Proactive and Reactive control modes, restricting analyses to correctly responded diagnostic congruent trials. The following model was used for estimation: *RT ~ 1 + mode + (1 + mode | ID)*. Mode was dummy coded with Reactive as the reference category to demonstrate the relative congruency cost (i.e., increase in reaction time) of proactive control for congruent diagnostic items. The intercept and mode were entered as random effects nested within subject, so that the congruency cost was also treated as a random effect.

In contrast to the results found in Tang et al. (2023), but in line with theoretical predictions and other reported findings, the congruency cost was reliably present in the 2018 dataset when analyzed with an HBR model that assumed a shifted log-normal distribution for RTs ( β = 0.02, se = 0.01, 95% HDI = [0, 0.05], pd. = 98.99%). Conversely, when this same effect was modeled with a conventional Gaussian distribution, the effect was not statistically reliable ( β = 8.92, se = 5.37, 95% HDI = [−1.81, 19.27], pd. = 95.09%). These contrasting patterns directly demonstrate that the choice of distribution can affect the inferences made about a small effect like the congruency cost.

To more fully understand this finding, we next sought to isolate which properties of the shifted log-normal distribution were essential for appropriately modeling this effect. To do so, the congruency cost was analyzed with a third distributional function: the ex-Gaussian. This distribution also captures the positive skew of response time data and additionally has a long prior history of work illustrating its increased sensitivity in modeling Stroop RT patterns (Heathcote et al., 1991). If capturing both the bulk of fast responses times along with a long tail of slow response times is necessary to accurately model the congruency cost, then the HBR model assuming an ex-Gaussian distribution should match the qualitative finding of the model using a shifted log-normal, but not Gaussian, function. Indeed, this prediction was confirmed, as a model assuming an ex-Gaussian distribution also found a statistically reliable congruency cost ( β = 9.87, se = 3.03, 95% HDI = [3.98, 15.8], pd. = 99.94%).

**Figure 4:**
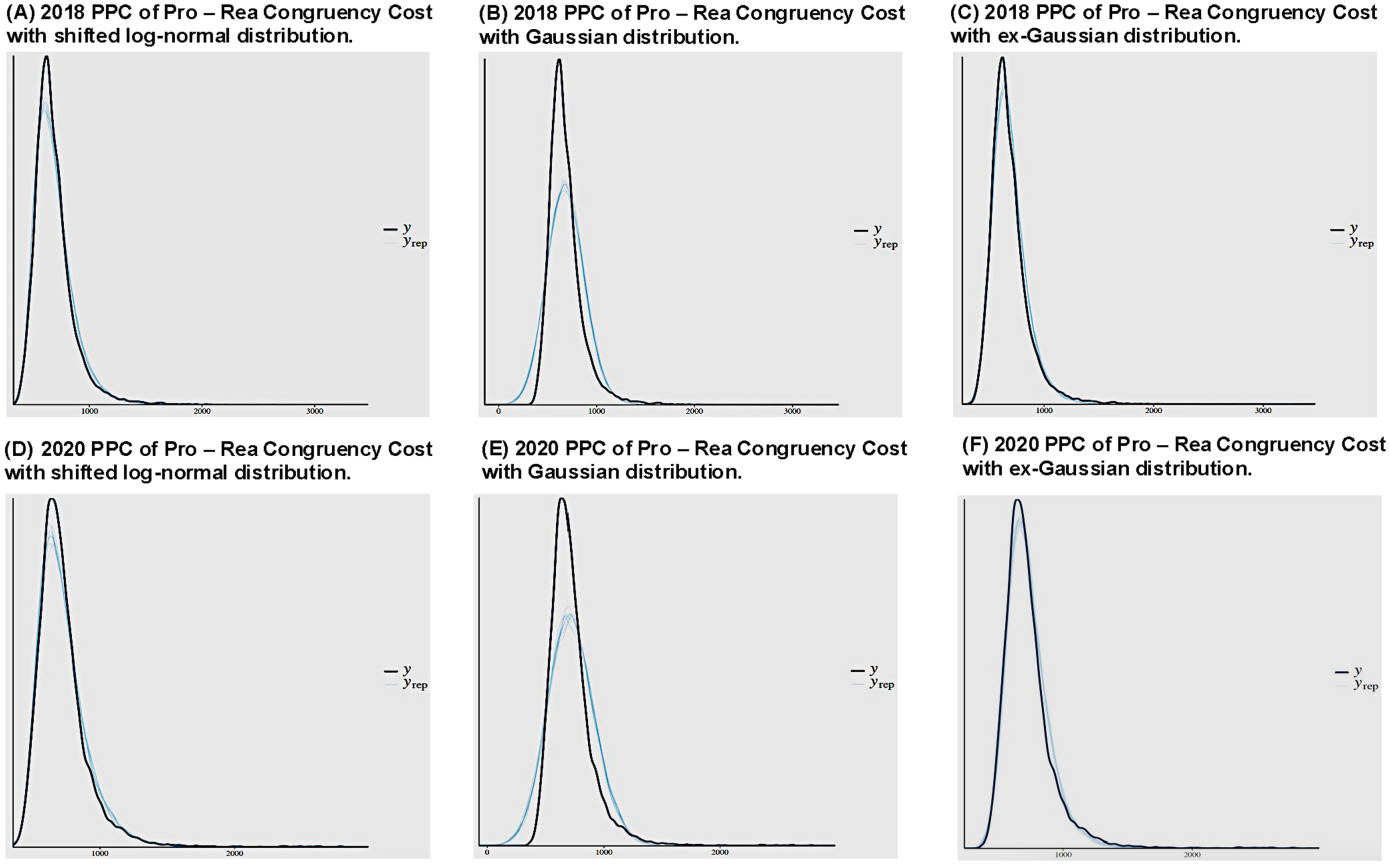
Stroop analyses, focused on modeling RT distributions. **(A)** The 2018 PPCs from a shifted lognormal distribution for RT show that the simulated data from this model consistently fits well onto the actual data. **(B)** The 2018 PPCs from a Gaussian distribution show that the simulated data from this model does not fit well onto the actual data in this case (i.e., the simulated data from a Gaussian function is more symmetric with a lower peak than the actual data). **(C)** The 2018 PPCs from an ex-Gaussian distribution show that the simulated data from this model consistently fits well onto the data. **(D)** The 2020 PPCs from a shifted lognormal distribution for RT show that the simulated data from this model consistently fits well onto the actual data. **(E)** The 2020 PPCs from a Gaussian distribution show that the simulated data from this model does not fit well onto the actual data in this case (i.e., the simulated data from a Gaussian function is more symmetric with a lower peak than the actual data). **(F)** The 2020 PPCs from an ex-Gaussian distribution show that the simulated data from this model consistently fits well onto the data.

A quick visual inspection of the posterior predictive checks (PPCs) for each of these models provides clear evidence that the shifted log-normal and ex-Gaussian distributions produce an improved fit to the observed RT data, relative to the Gaussian distribution (Figures 4A–C). However, the difference in predictive accuracy between the ex-Gaussian and shifted log-normal is less clear with this approach; we therefore next sought to more directly quantify the relative predictive accuracy of each distributional function in modeling the congruency cost. Through BF and LOO-CV model comparisons, we defined the ex-Gaussian model as *M*_0_, the shifted log-normal model as *M*_1_ and the Gaussian model as *M*_2_. The BF model comparison metric showed decisive evidence favoring the shifted log-normal (*M*_1_) over the ex-Gaussian (*M*_0_) (i.e, BF_10_ = 7.4 × 10^24^, or BF_01_ = 1.35 × 10^−25^), while the Gaussian model had the worst fit (*M*_0_ / *M*_2_; BF_20_ = 0, or BF_02_ = Inf). However, LOO-CV model comparison revealed that the difference in predictive accuracy between the shifted log-normal and ex-Gaussian was not meaningful (∆_M1–M0_elpd_LOO_ = −66.9, ∆_M1–M0_SE_LOO_ = 85.8), although the Gaussian was clearly worse than the shifted log-normal (∆_M1–M2_elpd_LOO_ = −7563.9, ∆_M1–M2_ SE_LOO_ = 338.3).

Given the discrepancy between the BF and LOO-CV model comparison conclusions, we next sought to investigate how the incorporation of additional information, from the 2020 sample, would impact the results. Interestingly, while the same quantitative and qualitative conclusions regarding the congruency cost were drawn from the cumulative 2020 HBR models that assumed either a shifted log-normal ( β = 0.03, se = 0.01, 95% HDI = [0.01, 0.04], pd. = 99.97%) or ex-Gaussian ( β = 11.11, se = 2.45, 95% HDI = [6.22, 15.77], pd. = 100%) distribution, the 2020 HBR model with a Gaussian function also showed strong evidence for this effect ( β = 11.55, se = 3.88, 95% HDI = [3.98, 19.21], pd. = 99.83%), aligning with the qualitative conclusions of the other models; however, the PPCs for the 2020 HBR models again demonstrated the same pattern of increased predictive accuracy of the skewed distributions, as before (Figures 4D–F). Furthermore, we once again ran BF and LOO-CV model comparisons. Despite the inclusion of additional data, we found the same contrasting pattern of results across model comparison metrics in which the BF measure favored the shifted log-normal over ex-Gaussian (i.e., BF_10_ = 8.4 × 10^21^), while the LOO-CV indicated no difference in predictive accuracy between the two metrics (∆_M1–M0_elpd_LOO_ = −36.4, ∆_M1–M0_SE_LOO_ = 58.3), but again that they were superior to the Gaussian (BF_20_ = 0; ∆_M1–M2_elpd_LOO_ = −4079.3, ∆_M1–M2_SE_LOO_ = 226.9). This consistent discrepancy regarding the differential benefits of the shifted log-normal and ex-Gaussian likelihood functions reinforces the value of applying multiple analytic approaches to avoid drawing potentially premature conclusions. More importantly to the conceptual principle of this section, both distributions were able to reliably identify the congruency cost within our data.

#### Summary

The results provide clear evidence that the identification of a subtle RT effect, such as the congruency cost, can be impacted by the chosen distribution to model it. With HBR modeling approaches, it is quite easy to select from a range of possible distributions, and then directly compare their performance to the available data via posterior predictive checks (PPCs) as well as BF and/or LOO-CV model comparisons. While such metrics are not meant to parse apart subtler differences in predictive accuracy between distributions that already capture the general properties of the data, the results from all model comparison metrics proved that the shifted log-normal and ex-Gaussian functions were both superior to the Gaussian distribution in reliably identifying the congruency cost. However, we also demonstrated that even the advantages garnered from skewed distributions versus the standard Gaussian distribution were influenced by the inclusion of both datasets.

At first blush, the findings related to the inclusion of both datasets may seem to indicate diminishing benefits to the use of more accurate distributions for RT data. Yet a more important interpretation relates to the efficiency and flexibility of the HBR framework. Specifically, the use of more appropriate distributions, such as the shifted log-normal or ex-Gaussian, may reliably detect RT effects of interest with less data than that required from a conventional Gaussian model. The degree of efficiency is likely to be moderated by the properties of each dataset. As a result, we advocate for a routine practice of directly comparing multiple likelihood functions to assess their relative predictive accuracy for the data on hand.

For this purpose, PPCs allow for a quick visual comparison of different likelihood functions on the observed data, while BF/LOO-CV model comparison approaches can be applied as computationally intensive, but more quantitatively direct, metrics to determine the best-fitting model. Critically however, these recommendations do not imply that predictive accuracy should be the decisive factor while selecting among different likelihood functions. Instead, likelihood functions can be selected and investigated in a flexible manner, to address various methodological goals. For example, it has been argued that a key advantage of the shifted log-normal function relates to its clear mechanistic interpretability (i.e., subject-level RT distributions should be decomposed into a non-decision time component and evidence accumulation process that results in a rightward skew) (Haines et al., 2025). Our goal here was primarily to illustrate the simultaneous predictive and theoretical limitations that come with fitting Gaussian distributions onto RT data. In addition to this more universal point however, these collective findings also practically demonstrate how the additional power gained by alternative likelihood functions can appropriately capture the congruency cost as a reliable metric of proactive control.

### Precise modeling of error distributions: cued-TS

#### Background

Within the Cued-TS paradigm, a primary indicator of interest for reactive control was the task-rule congruency effect (TRCE) for errors. The inclusion of punishment cues, presented shortly before stimulus presentation and paired with high-conflict items, was hypothesized to elicit late-stage reactive control, corresponding to enhanced performance on incongruent trials in the Reactive mode, relative to the Baseline and Proactive modes. Since a strong association should manifest between incongruence and punishment, the performance benefits were expected to extend even to non-incentivized incongruent trials, based on the detection of perceived conflict. This improved performance on incongruent trials was in turn expected to cause a relative reduction in the TRCE error effect within the Reactive mode. Initial evidence for this Reactive TRCE error effect was reported in Tang et al. (2023). However, the task design for the Reactive variant was novel at the time, so prior findings could not be considered. For such cases, the HBR approach is especially useful to either provide further validation, or to instead challenge initial findings from novel task manipulations.

When considering binary outcome variables, such as a correct or error response, the use of a logistic function operationalizes each observation in terms of the underlying probability that one outcome versus the other will occur. This statistical approach not only circumvents the distortion of results that can arise when making incorrect modeling assumptions with proportion data, but it also enables direct consideration of trial-level variability within each subject when applied through a hierarchical model. The trial-level granularity achieved by hierarchical logistic models cannot be assessed through the conventional use of error rates, which instead rely upon condition-based averages that omit such information. Indeed, this access to trial-level data allows for the effective modeling of more complex random effects within each participant. In other words, an error rate model will be more likely than a logistic regression model to run into convergence issues while modeling interaction terms that vary by subject. To effectively illustrate the specific advantages of modeling accuracy data as log odds versus error rates, both the logistic regression and error rate models were matched to include the same simple random effects (see Supplementary material Results, for qualitatively similar results from simultaneously modeling the TRCE error effect with a logistic function and maximal random effect structure). Here we illustrate how HBR models with a logistic function and a hierarchical structure (i.e., trials nested within conditions, further nested within individuals) can provide a more rigorous test of the Reactive TRCE effect than what was possible through the original analyses provided by Tang et al. (2023).

#### Analysis and results

The task-rule congruency effect (TRCE) for errors was modeled as the relative log-odds of making an error on incongruent trials versus congruent ones, with the following model estimating the difference in this effect across modes: Correct ~ 1 + con.id*mode + (1 + con.id | ID). The outcome variable ‘Correct’ was coded so the model predicts the log odds of making an error (i.e., correct = 0; incorrect = 1). The predictor ‘con.id’ was dummy coded to assess the difference in performance between incongruent and congruent trials (i.e., the TRCE effect). The predictor ‘mode’ was dummy coded, with separate models either using Baseline or Proactive as the reference category to test the relative effect of the Reactive mode on the log odds to make an error. Finally, the interaction effect between ‘mode’ and con.id’ reflects the key parameter of interest as the difference in TRCE error effect between modes. Both intercept and con.id are treated as random variables nested within subject to model the TRCE error as a random effect. Only non-incentivized trials were selected for analyses to allow for direct comparisons between the Reactive mode versus Baseline and Proactive. Furthermore, only biased (i.e., mostly congruent) trials were included since they were expected to elicit the largest TRCE error across modes. The key hypothesis was that the TRCE error would be reduced in the Reactive mode versus the Baseline and Proactive modes.

**Figure 5:**
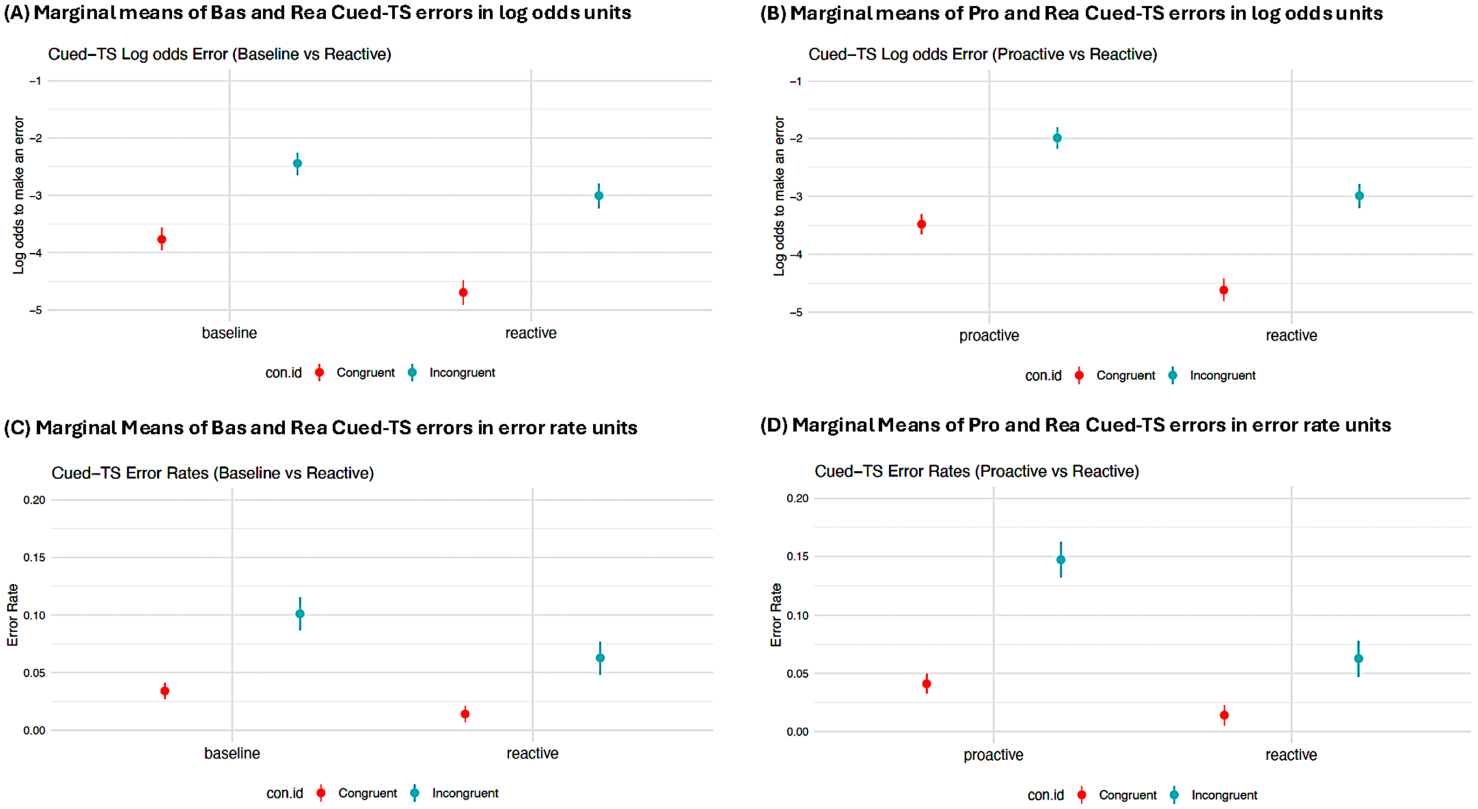
Cued-TS analyses, focused on modeling accuracy distributions. To generate these means, we removed the intercept from all models. **(A)** Marginal means of the reactive and baseline cued-TS errors in log odds units. There are significant main effects of mode and congruency as well as a positive interaction effect (which is opposite to what was found in Tang et al., 2023 and theoretical predictions). **(B)** Marginal means of the reactive and proactive cued-TS errors in log odds units. There are significant main effects of mode and congruency but no significant interaction effect (also different from what was found in Tang et al., 2023). **(C)** Marginal means of the reactive and baseline cued-TS errors in error rate units. There are significant main effects of mode and congruency as well as a negative interaction effect. **(D)** Marginal means of the reactive and proactive cued-TS errors in error rate units. There are significant main effects of mode and congruency as well as a negative interaction effect.

In contrast to these predictions however, the 2018 HBR models indicated that the TRCE error was actually increased, rather than decreased, in the Reactive as compared to Baseline mode ( β = 0.37, se = 0.11, 95% HDI = [0.15, 0.58], pd = 99.98%) (Figure 5A); furthermore there was little evidence for a difference between the Reactive and Proactive modes ( β = 0.14, se = 0.11, 95% HDI = [−0.08, 0.35], pd = 89.62%) (Figure 5B). These findings were qualitatively distinct from those reported in Tang et al. (2023). Specifically, in Tang et al. (2023), it was reported that the TRCE error was reduced in Reactive relative to both Baseline and Proactive, with the latter contrast showing high statistical reliability. Given this discrepancy in findings, we were interested in testing whether the logistic function provided a more accurate way to model the TRCE error.

Rather than summarizing the error rates as proportions and incorrectly assuming a Gaussian distribution, the use of logistic regression directly models the probability of an error through a Bernoulli distribution, transforming the data into log odds so that the function can more sensitively capture differences between low and high probabilities. While standard approaches assume a linear effect between predictors and the outcome variable, probabilities typically follow a sigmoidal function, in which the strength of probability changes is dependent on their initial values (i.e., the largest absolute change will be around *p* = 0.5, and the smallest changes will be near their extremes, due to their bounded nature). By utilizing a logit link to transform the data onto a log-odds scale, logistic regression will appropriately strengthen differences between conditions when the reference group is close to floor/ceiling. Since a ceiling effect will particularly apply to congruent trials across modes versus incongruent trials, this transformation provides a potential explanation for why the current results were in the opposite direction of initial findings. To test this interpretation of the findings, the results were re-analyzed by using error rates rather than log odds as the outcome variable. Indeed, when running a HBR model on the 2018 data, but with error rates, a consistent pattern with Tang et al. (2023) is observed, with a strong reduction in the TRCE effect observed for Reactive versus both Baseline ( β = − 0.02, se = 0.01, 95% HDI = [−0.03, 0], pd. = 99.56%) (see Figure 5C) and Proactive ( β = − 0.06, se = 0.01, 95% HDI = [−0.07, −0.04], pd. = 100%) modes (see Figure 5D).

#### Summary

The Cued-TS findings provide a clear demonstration that initial conclusions regarding effects from novel paradigms may not be consistent across different analytic approaches and metrics, thus highlighting the importance of carefully selecting statistical methods that lead to accurate and precise results. More specifically, the analyses modeling the difference in TRCE error between modes found there was only very weak evidence for a reduced Reactive effect (relative to Proactive) when utilizing a logistic function. Although these results conflict with previous conclusions drawn by Tang et al. (2023), greater confidence can be had in the validity of the results obtained from the HBR analysis, due to the now well-established benefits of hierarchical logistic regression models (Jaeger, 2008; Dixon, 2008; Houpt and Bittner, 2018).

However, it is also important to clarify that these findings are not in strong contradiction with the theoretical claims of the DMC framework, regarding benefits of Reactive control for Cued-TS performance. Indeed, when restricting analyses to just the incongruent trials, there were clear observed performance benefits present in the Reactive mode, regardless of whether the outcome variable was modeled in terms of error rates or the log odds to make an error (see Supplementary material for additional analyses on the simple and main effects of condition on accuracy performance). The primary distinctions between the models were instead on congruent trials, which had overall very low error rates (i.e., less than 5%). As a result, differences between control modes for these trials were not reliably detected in the models using summary error rate measures but were found to be consistently greater when modeled in terms of log odds. Since hierarchical logistic regression models are still not the norm for analyses of error rates in cognitive control tasks, it is possible that other effects involving low error rate conditions (e.g., congruent trials) have also been missed in prior studies. Consequently, an important direction for future work would be to better understand the circumstances in which task manipulations to the Cued-TS paradigm, such as that of the reactive control variant, might lead to performance improvements for both congruent and incongruent items.

## Discussion

The goal of this paper was to demonstrate how Hierarchical Bayesian Regression (HBR) models could be utilized to provide more detailed and valid statistical inferences, in terms of both parameter estimation and hypothesis testing, using the DMCC task battery as a case study example. In particular, we leveraged the following advantageous features of the HBR approach, to build upon prior findings with the DMCC dataset, initially reported in Tang et al. (2023): (1) the combined implementation of a sequential updating procedure and SDR metric to assess the replicability and consistency of the reduced Proactive and Reactive BX error interference effects; (2) the utilization of multiple null-hypothesis metrics (i.e., ROPE and BF model comparison) to test the relative strength of evidence against the Proactive NP RT effect (at least in contrast to reactive); (3) the library of available RT likelihood functions (e.g., ex-Gaussian and shifted log-normal) to more accurately model the Proactive congruency cost; and (4) the application of hierarchical logistic regression to appropriately model the reduced Reactive TRCE error effect. For each of these effects, more clearly refined quantitative and/or qualitative conclusions could be drawn for each of the DMCC tasks. Despite these clear benefits, a full assessment of the HBR approach also warrants an acknowledgement of its potential limitations, which we next turn to below.

### Limitations

While it is beyond the scope of this paper to compare other possible analytic approaches with our methods, we address here one clear alternative for how analyses comparing two independent samples might have been performed. In contrast to the sequential updating procedure used throughout this paper, the two available datasets could have been directly aggregated to perform a type of mega-analysis (Eisenhauer, 2021). The direct integration of two datasets and inclusion of ‘sample’ as an additional covariate (i.e., to compare the 2018 and 2020 samples) would enable a cumulative estimate to be computed, while also enabling detection of significant differences between the two samples. Consequently, it could be argued that the use of data-informative priors did not add anything meaningful to our results. Yet we believe the sequential updating procedure provided a more meaningful inference, regarding whether new information would significantly modify the original conclusions drawn regarding a particular estimate (i.e., we are not comparing the estimate between two datasets, but rather the estimate before and after the integration of new information), such as those reported for the BX error interference effect in Tang et al. (2023). Furthermore, since the original and cumulative estimates could be operationalized in terms of prior and posterior distributions, more specific hypotheses could be made regarding the relative probability of values within each distribution as well as the relative probability of values between the two distributions (Verhagen and Wagenmakers, 2014; Wagenmakers et al., 2016; Ly et al., 2019; Lin et al., 2024).

Yet it is important to acknowledge a clear limitation of sequential updating, in that the data-informative priors serve as proxy metrics that may not fully represent the attributes of the original dataset (i.e., directly combining the datasets may lead to more accurate cumulative estimates). Conversely, a standard aggregation approach not only loses the advantages associated with sequential updating, but it is inherently restricted to contexts where one has direct access to the available data as well as sufficient time and CPU memory/power to continuously aggregate new datasets (Oravecz et al., 2016). The benefits of informative priors come through their simultaneous efficiency and flexibility, which enable HBR models to not only have a stronger starting point from which to generate appropriate estimates, but also allow for the inclusion of more limited external information (e.g., fixed/random estimates from previous models). While care should be taken that these priors do not bias the results, with prior sensitivity analyses checking the degree to which the prior specifications may cause different outcomes, the ability to use data-informative priors should ultimately be considered as a clear strength of HBR models.

In our case, we took a conservative approach, only utilizing data-informed priors in sequential updating analyses testing for inter-sample reliability. However, future analyses should more fully consider the use of weakly informative priors to enable more efficient and tractable parameter estimation. Prior predictive checks can be used to carefully select a set of intermediate priors that are between the default of uninformed priors and model-based ones from previous samples (i.e., vaguely informative priors which are more widely dispersed than the latter but still more constrained than the former). The selection of these moderate priors not only lead to fewer convergence issues for more complex models but can also significantly speed up their runtime (Schad et al., 2021).

The case-study results described here highlight advantages of the HBR approach. However, these advantages are also balanced by important tradeoffs in computation time, particularly when modeling different underlying data structures (e.g., multiple sources of variability, non-linear relationships between variables and structures, and non-Gaussian likelihood functions). In our case, we were able to conduct a comprehensive set of HBR analyses on the DMCC task battery by making use of a university-wide computing cluster (i.e., the ‘Center for High Performance Computing’), which provided additional CPU resources to both speed up individual model runtime and allow multiple models to be run in parallel. Additionally, the more sophisticated computing interface ‘cmdstanr’ can utilize within-chain parallelization for each model (i.e., assigning multiple cores for each chain to reduce their execution time). Nevertheless, the inherent computational challenges of HBR models indicate the importance of acquiring additional resources for large-scale projects and following best practices to efficiently implement them (e.g., using weak rather than uninformative priors, prioritizing the most relevant random effects to model).

### General recommendations

For the interested researcher who wishes to switch away from conventional statistical tests to an HBR modeling approach, we recommend a gradual transition. By first understanding the different components of HBR and the benefits to be gained from each, one can flexibly utilize models of varying complexities to address the specific needs of a project. Through the use of DMCC case study examples, we sought to make the overarching point that conventional statistical approaches like t-tests and ANOVAs are not optimized to address the kinds of data that are routine within experimental psychology research, such as non-Gaussian outcome variables (i.e., accuracy, RT) and trial-level data nested within individuals. While the benefits of HBR modeling may be less notable for large group-level effects and sample sizes, we believe that conclusions drawn in experimental psychology research may be particularly susceptible to misinterpretations if systematic trial-level variability is ignored. In addition to each subject having notable differences in their baseline cognitive characteristics, there will also be changes that can be expected in behavioral performance as participants become more familiar with the underlying structure of a given task. For instance, Viviani et al. (2023b, 2024) highlighted the value of explicitly modeling within-subject changes in Stroop performance as each participant learned the potential statistical regularities within the task environment. As a result of these factors, we suggest that the continued use of conventional approaches will contribute to the inflation of false negative/positive results, which were identified in our own work for the Stroop congruency cost and Cued-TS TRCE error effect, respectively.

Although more sophisticated approaches are necessary to properly address issues such as cross-trial learning, it is important to acknowledge that generalized hierarchical models by themselves (i.e., non-Bayesian) may be conceptually sufficient to accurately model response time and error rate data. For example, a non-Gaussian hierarchical logistic regression model could have been applied to the TRCE error effect to reach the same qualitative conclusion regarding its failed replication. Similarly, a non-Gaussian distribution from standard generalized hierarchical modeling packages like lme4() could have been assumed for the Stroop RT data. Nevertheless, we do recommend the brms() package as the most flexible one for RT modeling, as it offers a wider library of likelihood functions, including the shifted log-normal and ex-Gaussian functions that were specifically derived for RT data in cognitive psychology paradigms.

Since frequentist generalized hierarchical models can already yield novel insights within the task battery, our overarching goal is not necessarily to argue for a Bayesian framework over a frequentist one, but instead to highlight the possible advantages that can come with utilizing more advanced analytic approaches, relative to that of conventional statistical methods. Still, we felt that HBR models were particularly well-suited for us to make more nuanced conclusions regarding key phenomenon of interest. Indeed, these models not only encompass the capabilities of earlier models but also extend beyond them to readily offer a set of additional advantages, such as in providing cumulative parameter estimation across multiple sources of information, and in allowing for more nuanced statements regarding inter-sample replicability and null evaluation.

Beyond our current application, HBR models can also incorporate even more sophisticated analytic approaches. For instance, Diffusion Decision models (DDM) extend beyond the likelihood functions described within this manuscript (i.e., shifted log-normal and ex-Gaussian, Bernoulli) by simultaneously modeling both trial-level accuracy and response time data (Ratcliff and McKoon, 2008; Myers et al., 2022). Through the inclusion of additional mechanistic parameters (i.e., boundary separation, starting point, drift rate and non-decision time), DDMs can more fully mimic the diffusion process that underlies most 2-choice cognitive control paradigms, in which participants will noisily accumulate evidence in favor of one decision over the other. Indeed, this approach can even more accurately capture the proactive/reactive control indicators within our Cued-TS, AX-CPT and SternbergWM task variants (e.g., TRCE error/RT effects, BX interference error/RT effects, and NP error/RT effects, respectively). While such models would not be appropriate for the DMCC Stroop tasks, which involve many potential vocal responses as well as ceiling-level accuracy rates, the inverse Gaussian/Wald likelihood function could be applied to simulate this diffusion process independently from the choice itself (Anders et al., 2016; Luce, 1991). Although these more complex models require additional computational time and resources to perform, DDMs and related models have become increasingly applied within experimental psychology, to gain an improved understanding of cognitive variability. For instance, Aschenbrenner and Jackson (2025) recently implemented DDMs through the ‘brms’ package to examine how healthy ageing and mild cognitive impairment impacts sustained attentional performance across all diffusion model parameters. Specifically, ageing in general was shown to strongly impact mean drift rate, boundary separation and non-decision time, with mild cognitive impairment exacerbating drift rates. Given their feasibility and practical benefits, we plan to apply such models to the DMCC datasets in a future project. More generally, the ability to also utilize DDMs and inverse Gaussian distributions further illustrate the flexibility and power of HBR approaches.

Indeed, the primary contribution of the HBR framework is that it allows for the full incorporation of the advantages found within many different analytic techniques, providing a comprehensive foundation from which to assess and characterize phenomena of interest. We therefore recommend a scaffolding perspective to understand the benefits that can be gained from adopting analytic approaches of varying complexity. Although the many differences between standard and complex analytic approaches may initially seem daunting, there has been a growing movement toward making these methods more accessible with affordable hardware, user-friendly software/packages, and detailed tutorials on how to implement them (Gelman and Hill, 2006; Kruschke, 2014; McElreath, 2018; van de Schoot et al., 2014; Nalborczyk et al., 2019; Veenman et al., 2024). As such, interested researchers will be in the position to make better informed decisions regarding when the use of HBR modeling is warranted for their scientific questions of interest.

## Data Availability

The original contributions presented in the study are included in the article/Supplementary material. Data and code for all analyses are publicly available on the OSF platform (https://osf.io/bj6fy/), further inquiries can be directed to the corresponding author.

## Footnotes

1 Note our chosen term ‘hierarchical’ is often used interchangeably with the terms ‘multilevel’ and ‘mixed-effect’ in this literature; all of these terms refer to statistical models that account for systematically clustered observations.

2 A second paper published on the 2018 dataset also used HBR approaches, but those analyses were focused on test–retest reliability and cross-task correlations (Snijder et al., 2023). Consequently, this is the first analysis of the 2018 dataset using HBR approaches for statistical inference regarding within-subject group average effects.

3 HDIs are a type of credible interval (CI) that inherently incorporates the posterior distribution’s scale. While equal tailed intervals (ETIs) are the default CI in ‘brms’ and ensure there are evenly sized tails on both sides of the distribution, HDIs are better suited to account for potentially skewed distributions and ROPE analyses (Kruschke, 2014). We therefore only report HDIs in our paper/analyses.

4 An astute reader may note that age is a known variable that impacts the ability or tendency to implement cognitive control. Therefore, the allowance of a wide age range could impact the results. However, the analyses described here are focused on the general advantages of HBR models, and the role of replication datasets. As such, the issue of age effects is not germane, and should not impact any of the conclusions being drawn.

5 The 2018 wave 1 testing sample did not collect age, so age range/mean/sd were generated through the wave 1 retesting sample and wave 2 testing sample.

6 While subject-level filtering criteria were applied at the trial/item type level for Snijder et al. (2023), these criteria were later deemed to be overly restrictive. As a result, all our criteria were only applied at the condition level (i.e., session/mode and phase).

7 To ensure that the ‘Intercept’ parameter represents the reference categories for each predictor instead of their centered values, the ‘0 + intercept’ syntax is included on the right-hand side ‘brms’ formulas, instead of conventional ‘1 + …’ in frequentist modeling packages (Bürkner, 2017). This syntax especially helps to ensure appropriate prior specifications (e.g., our other sample model output for the intercept will be matched to our current model, rather than its centered counterpart). However, we write our model formulas in the manuscript with ‘1 + …’ syntax to avoid confusion among non-brms package users.

8 These results were from two separate models fit onto different subsets of the data (i.e., Proactive and Baseline data; Proactive and Reactive data). Based on the Reviewer’s suggestion, we additionally ran models that not only included all levels of the ‘mode’ predictor but also every trial and item type (i.e., NP/RN/NN and critical/non-critical items). While BF model comparison is not feasible for such a model, we did observe that the 89% HDI for the Proactive – Reactive NP RT effect was now completely outside our ROPE interval. Still, this effect was in the opposite direction of theoretical predictions, thus serving as additional evidence against the effectiveness of our experimental manipulation.
