# Supplementary material for: Hierarchical Bayesian Regression for experimental psychology: a case study of cognitive control
Source: Front Psychol. 2026 Mar 19;17:1643463. doi: 10.3389/fpsyg.2026.1643463 (PMC13043376; doi:10.3389/fpsyg.2026.1643463)
Supplement: Supplementary file 1 [file data_sheet_1.pdf]

Supplementary Table of Contents

1 Descriptive Statistics

1.1 2018 (Test + Retest) Results
- 1.1.1 AX-CPT 7
- 1.1.2 Sternberg 9
- 1.1.3 Stroop 10
- 1.1.4 Cued-TS 11

1.2 2020 (Test only) Results
- 1.2.1 AX-CPT 12
- 1.2.2 Sternberg 14
- 1.2.3 Stroop 15
- 1.2.4 Cued-TS 16

1.3 Combined (2018 + 2020) Results
- 1.3.1 AX-CPT 17
- 1.3.2 Sternberg 19
- 1.3.3 Stroop 20
- 1.3.4 Cued-TS 21

2 AX-CPT

2.1 A-Cue Bias 2018
2.1.1 Proactive - Baseline A-Cue Bias 25
2.1.2 Proactive - Reactive A-Cue Bias 26
2.1.3 A-Cue Bias 27

2.2 A-Cue Bias 2020
2.2.1 Proactive - Baseline A-Cue Bias 29
2.2.2 Proactive - Reactive A-Cue Bias 31
2.2.3 A-Cue Bias 33

2.3 BX Error Interference 2018
2.3.1 Proactive - Baseline BX error interference 38
2.3.2 Reactive - Baseline BX error interference 39

2.4 BX Error Interference 2020
2.4.1 Proactive - Baseline BX error interference 41
2.4.2 Reactive - Baseline BX error interference 43

2.5 BX RT Interference 2018
2.5.1 Proactive - Baseline BX RT interference 46
2.5.2 Reactive - Baseline BX RT interference 47
2.5.3 Reactive - Proactive BX RT interference 48

2.6 BX RT Interference 2020
2.6.1 Proactive - Baseline BX RT interference 50
2.6.2 Reactive - Baseline BX RT interference 52
2.6.3 Reactive - Proactive BX RT interference 54

2.7 AX-CPT Single Model Output
2.7.1 2018 A-Cue Bias Single Model Output 57
2.7.2 2020 A-Cue Bias Single Model Output 58
2.7.3 2018 BX error interference Single Model Output 59
2.7.4 2020 BX Error Interference Single Model Output 60
2.7.5 2018 BX RT interference Single Model Output 61
2.7.6 2020 BX RT Interference Single Model Output 62

3 Sternberg

3.1 NP Error Effect 2018 . . . .

3.1.1 Proactive - Baseline NP Error Effect 65

3.1.2 Proactive - Reactive NP Error Effect 66

3.2 NP Error Effect 2020 . . . .

3.2.1 Proactive - Baseline NP Error Effect 68

3.2.2 Proactive - Reactive NP Error Effect 71

3.3 NP RT Effect 2018 . . . .

3.3.1 Proactive - Baseline NP RT Effect 75

3.3.2 Proactive - Reactive NP RT Effect 76

3.4 NP RT Effect 2020 . . . .

3.4.1 Proactive - Baseline NP RT Effect 78

3.4.2 Proactive - Reactive NP RT Effect 84

3.5 RN Recency Effect RT 2018 . . . .

3.5.1 Reactive - Baseline RN Recency Effect 90

3.5.2 Reactive - Proactive RN Recency Effect 91

3.6 RN Recency Effect RT 2020 . . . .

3.6.1 Reactive - Baseline RN Recency Effect 94

3.6.2 Reactive - Proactive RN Recency Effect 96

3.7 Sternberg Single Model Output . . . .

3.7.1 2018 NP Error Effect Single Model Output 99

3.7.2 2020 NP Error Effect Single Model Output 100

3.7.3 2018 NP RT Effect Single Model Output (ex-Gaussian). 101

3.7.4 2020 NP RT Effect Single Model Output (ex-Gaussian). 102

3.7.5 2018 NP RT Effect Single Model Output (shifted log-normal). 104

3.7.6 2020 NP RT Effect Single Model Output (shifted log-normal) 105

3.7.7 2018 RN Recency RT Effect Single Model Output (shifted log-normal). 106

3.7.8 2020 RN Recency RT Effect Single Model Output (shifted log-normal). 107

4 Stroop

4.1 LWPC Effect 2018 . . . .

4.1.1 Proactive - Baseline LWPC Effect for Biased Items 110

4.1.2 Proactive - Baseline LWPC Effect for PC-50 Items 111

4.2 LWPC Effect 2020 . . . .

4.2.1 Proactive - Baseline LWPC effect for Biased items 113

4.2.2 Proactive - Baseline LWPC Effect for PC-50 items 115

4.3 ISPC Effect 2018 . . . .

4.3.1 Reactive - Baseline ISPC Effect for Biased Items 118

4.3.2 Reactive - Baseline ISPC Effect for PC-50 items 119

4.4 ISPC Effect 2020 . . . .

4.4.1 Reactive - Baseline ISPC effect for Biased Items 121

4.4.2 Reactive - Baseline ISPC effect for PC-50 items 123

4.5 Transfer Cost 2018 . . . .

4.5.1 Reactive - Proactive Transfer Cost 126

4.6 Transfer Cost 2020 . . . .

4.6.1 Reactive - Proactive Transfer Cost 128

4.7 Congruency Cost 2018 . . . .

4.7.1 Proactive - Reactive Congruency Cost (Shifted Log-normal) 131

4.7.2 Proactive - Reactive Congruency Cost (Gaussian) 133

4.7.3 Proactive - Reactive Congruency Cost (ex-Gaussian) 135

4.7.4 Proactive - Reactive Congruency Cost (inverse Gaussian) 138

4.8 Congruency Cost 2020 . . . .

4.8.1 Proactive - Reactive Congruency Cost (Shifted Log-normal) 142

4.8.2 Proactive - Reactive Congruency Cost (Gaussian) 145

4.8.3 Proactive - Reactive Congruency Cost (ex-Gaussian) 148

4.9 Stroop Single Model Output . . . .

4.9.1 2018 [LW/IS]PC Single Model Output 153

4.9.2 2020 [LW/IS]PC Single Model Output 154

4.9.3 2018 Transfer Cost Single Model Output 155

4.9.4 2020 Transfer Cost Single Model Output 156

4.9.5 2018 Congruency Cost Single Model Output 157

4.9.6 2020 Congruency Cost Single Model Output 158

4.10 Stroop Results with Multimodality (MM) . . . .

4.10.1 2018 PC-Effects with MM 160

4.10.2 2020 PC-Effects with MM 161

4.10.3 2018 Transfer Cost with MM 162

4.10.4 2020 Transfer Cost with MM 163

4.10.5 2018 Congruency Cost with MM 164

4.10.6 2020 Congruency Cost with MM 165

5 Cued-TS

5.1 Non-Incentivized Congruent RT 2018 . . . .
5.1.1 Proactive - Baseline Non-Inc Congruent RT Effect 168
5.1.2 Proactive - Reactive Non-Inc Congruent RT Effect 169

5.2 Non-Incentivized Congruent RT 2020 . . . .
5.2.1 Proactive - Baseline Non-Inc Congruent RT Effect 171
5.2.2 Proactive - Reactive Non-Inc Congruent RT Effect 173

5.3 TRCE Error 2018 . . . .
5.3.1 Reactive - Baseline TRCE Error Logistic [with Max Random Effect Structure] 176
5.3.2 Reactive - Proactive TRCE Error Logistic [with Max Random Effect Structure] 177
5.3.3 Reactive - Baseline TRCE Error Logistic 178
5.3.4 Reactive - Proactive TRCE Error Logistic 179
5.3.5 Main Reactive and Baseline Logistic 180
5.3.6 Main Reactive and Proactive Logistic 181
5.3.7 Incongruent Reactive and Baseline Logistic 182
5.3.8 Incongruent Reactive and Proactive Logistic 183
5.3.9 Marginal Means Reactive and Baseline Logistic 184
5.3.10 Marginal Means Reactive and Proactive Logistic 185
5.3.11 Reactive - Baseline TRCE Error Rate 186
5.3.12 Reactive - Proactive TRCE Error Rate 187
5.3.13 Main Reactive and Baseline Error Rates 188
5.3.14 Main Reactive and Proactive Error Rates 189
5.3.15 Incongruent Reactive and Baseline Error Rates 190
5.3.16 Incongruent Reactive and Proactive Error Rates 191
5.3.17 Marginal Means Reactive and Baseline Error Rates 192
5.3.18 Marginal Means Reactive and Proactive Error Rates 193

5.4 TRCE Error 2020 . . . .
5.4.1 Reactive - Baseline TRCE Error Logistic [with Max Random Effect Structure] 195
5.4.2 Reactive - Proactive TRCE Error Logistic [with Max Random Effect Structure] 198
5.4.3 Reactive - Baseline TRCE Error Logistic 201
5.4.4 Reactive - Proactive TRCE Error Logistic 204
5.4.5 Main Reactive and Baseline Logistic 207
5.4.6 Main Reactive and Proactive Logistic 209
5.4.7 Incongruent Reactive and Baseline Logistic 211
5.4.8 Incongruent Reactive and Proactive Logistic 213
5.4.9 Marginal Means Reactive and Baseline Logistic 215
5.4.10 Marginal Means Reactive and Proactive Logistic 216
5.4.11 Reactive - Baseline TRCE Error Rates 217
5.4.12 Reactive - Proactive TRCE Error Rates 219
5.4.13 Main Reactive and Baseline Error Rates 221
5.4.14 Main Reactive and Proactive Error Rates 223
5.4.15 Incongruent Reactive and Baseline Error Rates 225

5.4.16 IncongruentReactive and Proactive Error Rates 227
5.4.17 Marginal Means Reactive and Baseline Error Rates 229
5.4.18 Marginal Means Reactive and Proactive Error Rates 230

5.5 TRCE RT 2018 . . . .
5.5.1 Reactive - Baseline TRCE RT 232
5.5.2 Reactive - Proactive TRCE RT 235

5.6 TRCE RT 2020 . . . .
5.6.1 Reactive - Baseline TRCE RT 239
5.6.2 Reactive - Proactive TRCE RT 247

5.7 Cued-TS Single Model Output . . . .
5.7.1 2018 Cued-TS Non-Inc Congruent RT Single Model Output 256
5.7.2 2020 Cued-TS Non-Inc Congruent RT Single Model Output 257
5.7.3 2018 TRCE Error Single Model Output, Logistic 258
5.7.4 2020 TRCE Error Single Model Output, Logistic 259
5.7.5 2018 Cued-TS Error Main Effect Single Model Output, Logistic 260
5.7.6 2020 Cued-TS Error Main Effect Single Model Output, Logistic 261
5.7.7 2018 Cued-TS Error Incongruent Effect Single Model Output, Logistic . . . 262
5.7.8 2020 Cued-TS Error Incongruent Effect Single Model Output, Logistic . . . 263
5.7.9 2018 TRCE Error Single Model Output, Error Rates 264
5.7.10 2020 TRCE Error Single Model Output, Error Rates 265
5.7.11 2018 Cued-TS Main Effect Single Model Output, Error Rates 266
5.7.12 2020 Cued-TS Error Main Effect Single Model Output, Error Rates 267
5.7.13 2018 Cued-TS Error Incongruent Effect Single Model Output, Error Rates 268
5.7.14 2020 Cued-TS Error Incongruent Effect Single Model Output, Error Rates . 269
5.7.15 2018 TRCE RT Single Model Output 270
5.7.16 2020 TRCE RT Single Model Output 271

Supplemental Table 1.1: 2018 (Test + Retest) AX-CPT Results Across Trial Types and Conditions

Condition	Trial Type	RT Mean (SD)	Error Mean (SD)	
Baseline	AX	443.14 (70.73)	0.06 (0.06)	
	AY	523.80 (66.38)	0.07 (0.07)	
	A-nogo	–	0.12 (0.10)	
	BX	486.01 (101.05)	0.13 (0.11)	
	BY	437.39 (61.86)	0.01 (0.01)	
	B-nogo	–	0.22 (0.15)	
Proactive	AX	408.66 (73.73)	0.05 (0.05)	
	AY	520.80 (74.62)	0.19 (0.17)	
	A-nogo	–	0.17 (0.17)	
	BX	438.61 (106.07)	0.10 (0.10)	
	BY	404.53 (67.62)	0.01 (0.01)	
	B-nogo	–	0.33 (0.22)	
Reactive	AX	427.60 (75.81)	0.07 (0.06)	
	AY	544.23 (78.40)	0.07 (0.07)	
	A-nogo	–	0.09 (0.07)	
	BX	520.73 (91.08)	0.10 (0.11)	
	BY	417.00 (67.23)	0.01 (0.01)	
	B-nogo	–	0.13 (0.10)	
Condition	Derived Measure	RT Mean (SD)	Error Mean (SD)	Z-scores Mean (SD)
Baseline	A-cue Bias	–	–	0.06 (0.28)
	BX Interference	48.62 (60.65)	0.13 (0.10)	–
	d'-context	–	–	2.90 (0.78)
	PBI	0.04 (0.07)	-0.23 (0.46)	–
Proactive	A-cue Bias	–	–	0.40 (0.41)
	BX Interference	34.08 (55.59)	0.10 (0.09)	–
	d'-context	–	–	3.21 (0.88)
	PBI	0.09 (0.08)	0.21 (0.53)	–
Reactive	A-cue Bias	–	–	0.03 (0.30)

BX Interference	103.73 (54.01)	0.10 (0.11)	–
d'-context	–	–	2.99 (0.89)
PBI	0.02 (0.05)	-0.09 (0.52)	–

Supplemental Table 1.2: 2018 (Test + Retest) Sternberg Results Across Trial Types and Conditions

Condition	Trial Type	RT Mean (SD)	Error Mean (SD)
Baseline	NN	Critical: 829.06 (152.27)	Critical: 0.04 (0.06)
		High: 871.10 (154.26)	High: 0.08 (0.07)
	NP	Critical: 864.15 (152.05)	Critical: 0.14 (0.12)
		High: 882.46 (157.78)	High: 0.20 (0.11)
	RN	Critical: 929.82 (162.58)	Critical: 0.18 (0.14)
		High: 998.23 (189.15)	High: 0.40 (0.21)
Proactive	NN	Critical: 824.72 (144.26)	Critical: 0.05 (0.07)
		Low: 800.26 (144.16)	Low: 0.04 (0.06)
	NP	Critical: 837.77 (137.52)	Critical: 0.13 (0.11)
		Low: 769.43 (135.23)	Low: 0.05 (0.06)
	RN	Critical: 971.26 (181.87)	Critical: 0.27 (0.19)
		Low: 846.23 (171.96)	Low: 0.07 (0.12)
Reactive	NN	Critical: 844.55 (153.87)	Critical: 0.04 (0.08)
		High: 898.51 (174.77)	High: 0.07 (0.12)
	NP	Critical: 854.37 (137.07)	Critical: 0.11 (0.09)
		High: 891.44 (137.07)	High: 0.21 (0.11)
	RN	Critical: 930.11 (159.32)	Critical: 0.13 (0.11)
		High: 992.51 (152.31)	High: 0.28 (0.15)
**Condition**	**Derived Measure**	**RT Mean (SD)**	**Error Mean (SD)**
Baseline	Recent Negative Effect	Critical: 100.75 (79.51)	Critical: 0.14 (0.12)
		Non-critical: 127.13 (117.06)	Non-critical: 0.32 (0.20)
Proactive		Critical: 146.54 (84.52)	Critical: 0.22 (0.17)
		Non-critical: 45.97 (88.97)	Non-critical: 0.03 (0.10)
Reactive		Critical: 85.56 (73.47)	Critical: 0.08 (0.10)
		Non-critical: 94.00 (102.83)	Non-critical: 0.21 (0.14)

Supplemental Table 1.3: 2018 (Test + Retest) Stroop Results Across Trial Types and Conditions

Condition	Trial Type	RT Mean (SD)	Error Mean (SD)
Baseline	Biased	Congruent: 665.06 (101.89)	Congruent: 0.02 (0.03)
		Incongruent: 797.74 (123.53)	Incongruent: 0.05 (0.05)
		Stroop Effect: 132.67 (58.17)	Stroop Effect: 0.03 (0.04)
	PC-50	Congruent: 682.21 (102.99)	Congruent: 0.03 (0.04)
		Incongruent: 797.46 (116.89)	Incongruent: 0.04 (0.05)
		Stroop Effect: 115.25 (59.25)	Stroop Effect: 0.02 (0.03)
Proactive	Biased	Congruent: 684.21 (125.69)	Congruent: 0.01 (0.03)
		Incongruent: 766.62 (137.91)	Incongruent: 0.03 (0.04)
		Stroop Effect: 82.41 (45.93)	Stroop Effect: 0.02 (0.02)
	PC-50	Congruent: 690.62 (122.86)	Congruent: 0.01 (0.03)
		Incongruent: 787.80 (131.24)	Incongruent: 0.03 (0.04)
		Stroop Effect: 97.18 (56.64)	Stroop Effect: 0.02 (0.02)
Reactive	Biased	Congruent: 658.49 (117.91)	Congruent: 0.01 (0.02)
		Incongruent: 751.31 (127.22)	Incongruent: 0.04 (0.03)
		MC Filler: xxxx	MC Filler: xxxxx
		Stroop Effect: 92.82 (49.23)	Stroop Effect: 0.03 (0.03)
	PC-50	Congruent: 681.83 (113.42)	Congruent: 0.02 (0.04)
		Incongruent: 801.38 (125.63)	Incongruent: 0.04 (0.05)
		Stroop Effect: 119.55 (60.64)	Stroop Effect: 0.03 (0.04)

Condition	Derived Measure	Trial Type	RT Mean (SD)
Proactive	Congruency Cost	Biased	19.14 (83.55)
		PC-50	8.41 (72.69)
	Transfer Cost	–	14.76 (35.06)
Reactive	Congruency Cost	Biased	-6.58 (85.29)
		PC-50	-.38 (75.38)
	Transfer Cost	–	26.73 (39.98)

Supplemental Table 1.4: 2018 (Test + Retest) Cued-TS Results Across Trial Types and Conditions

Condition	Trial Type	RT Mean (SD)	Error Mean(SD)
Baseline	Biased (Non-Incentivized)	Congruent: 905.52 (259.69)<br>Incongruent: 979.61 (293.06)<br>TRCE: 74.09 (126.23)	Congruent: 0.03 (0.04)<br>Incongruent: 0.10 (0.10)<br>TRCE: 0.07 (0.09)
	Unbiased (Non-Incentivized)	Congruent: 945.57 (285.43)<br>Incongruent: 1021.05 (289.48)<br>TRCE: 75.48 (112.59)	Congruent: 0.05 (0.05)<br>Incongruent: 0.13 (0.09)<br>TRCE: 0.08 (0.08)
Proactive	Biased (Non-Incentivized)	Congruent: 710.71 (186.57)<br>Incongruent: 769.73 (227.30)<br>TRCE: 59.02 (100.03)	Congruent: 0.04 (0.04)<br>Incongruent: 0.15 (0.11)<br>TRCE: 0.11 (0.11)
	Unbiased (Incentivized)	Congruent: 689.89 (199.48)<br>Incongruent: 738.88 (217.82)<br>TRCE: 48.99 (77.47)	Congruent: 0.05 (0.06)<br>Incongruent: 0.14 (0.08)<br>TRCE: 0.09 (0.09)
Reactive	Biased (Non-Incentivized)	Congruent: 989.14 (315.58)<br>Incongruent: 1079.42 (351.15)<br>TRCE: 90.28 (153.15)	Congruent: 0.01 (0.02)<br>Incongruent: 0.06 (0.08)<br>TRCE: 0.05 (0.07)
	Unbiased (Incentivized)	Congruent: 1130.01 (390.91)<br>Incongruent: 1193.69 (379.68)<br>TRCE: 63.67 (133.68)	Congruent: 0.01 (0.03)<br>Incongruent: 0.06 (0.06)<br>TRCE: 0.05 (0.07)

Supplemental Table 2.1: 2020 (Test only) AX-CPT Results Across Trial Types and Conditions

Condition	Trial Type	RT Mean (SD)	Error Mean (SD)	
Baseline	AX	458.47 (67.55)	0.06 (0.07)	
	AY	538.77 (86.46)	0.06 (0.07)	
	A-nogo	–	0.12 (0.11)	
	BX	514.96 (112.98)	0.16 (0.18)	
	BY	457.65 (63.11)	0.01 (0.01)	
	B-nogo	–	0.19 (0.16)	
Proactive	AX	419.32 (78.41)	0.06 (0.12)	
	AY	534.34 (94.26)	0.15 (0.16)	
	A-nogo	–	0.15 (0.18)	
	BX	467.10 (106.44)	0.12 (0.14)	
	BY	423.42 (72.74)	0.01 (0.02)	
	B-nogo	–	0.28 (0.20)	
Reactive	AX	429.33 (86.00)	0.06 (0.06)	
	AY	547.48 (96.37)	0.08 (0.09)	
	A-nogo	–	0.09 (0.09)	
	BX	518.94 (110.33)	0.08 (0.12)	
	BY	419.93 (75.34)	0.01 (0.01)	
	B-nogo	–	0.14 (0.14)	
**Condition**	**Derived Measure**	**RT Mean (SD)**	**Error Mean (SD)**	**Z-scores Mean (SD)**
Baseline	A-cue Bias	–	–	0.09 (0.32)
	BX Interference	57.30 (70.43)	0.17 (0.16)	–
	d'-context	–	–	2.78 (0.86)
	PBI	.03 (.08)	-0.26 (0.49)	–
Proactive	A-cue Bias	–	–	0.33 (0.43)
	BX Interference	43.68 (63.10)	0.12 (0.13)	–
	d'-context	–	–	3.03 (0.91)
	PBI	0.07 (0.08)	0.09 (0.55)	–
Reactive	A-cue Bias	–	–	0.12 (0.32)

BX Interference	99.00 (64.64)	0.09 (0.11)	–
d’-context	–	–	3.09 (0.85)
PBI	0.03 (0.06)	0.02 (0.48)	–

Supplemental Table 2.2: 2020 (Test only) Sternberg Results Across Trial Types and Conditions

Condition	Trial Type	RT Mean (SD)	Error Mean (SD)
Baseline	NN	Critical: 843.54 (156.87)	Critical: 0.03 (0.06)
		High: 900.89 (164.77)	High: 0.07 (0.07)
	NP	Critical: 901.80 (177.41)	Critical: 0.12 (0.11)
		High: 925.60 (176.60)	High: 0.20 (0.11)
	RN	Critical: 986.45 (195.25)	Critical: 0.20 (0.19)
		High: 1027.14 (218.86)	High: 0.50 (0.21)
Proactive	NN	Critical: 846.97 (179.80)	Critical: 0.04 (0.07)
		Low: 829.88 (182.48)	Low: 0.05 (0.07)
	NP	Critical: 870.04 (176.90)	Critical: 0.10 (0.11)
		Low: 810.26 (175.21)	Low: 0.04 (0.07)
	RN	Critical: 1009.32 (208.08)	Critical: 0.25 (0.22)
		Low: 879.92 (207.66)	Low: 0.06 (0.14)
Reactive	NN	Critical: 832.67 (178.51)	Critical: 0.04 (0.09)
		High: 881.44 (181.83)	High: 0.08 (0.14)
	NP	Critical: 846.08 (174.64)	Critical: 0.11 (0.11)
		High: 887.52 (167.36)	High: 0.21 (0.11)
	RN	Critical: 906.56 (168.45)	Critical: 0.10 (0.12)
		High: 976.43 (173.41)	High: 0.27 (0.15)

Condition	Derived Measure	RT Mean (SD)	Error Mean (SD)
Baseline	Recent Negative Effect	Critical: 142.90 (122.17)	Critical: 0.17 (0.18)
		Non-critical: 126.25 (153.94)	Non-critical: 0.42 (0.21)
Proactive		Critical: 162.35 (111.64)	Critical: 0.22 (0.19)
		Non-critical: 50.04 (109.80)	Non-critical: 0.01 (0.12)
Reactive		Critical: 73.89 (82.69)	Critical: 0.06 (0.11)
		Non-critical: 94.99 (115.07)	Non-critical: 0.19 (0.15)

Supplemental Table 2.3: 2020 (Test only) Stroop Results Across Trial Types and Conditions

Condition	Trial Type	RT Mean (SD)	Error Mean (SD)
Baseline	Biased	Congruent: 738.09 (154.57)	Congruent: 0.05 (0.09)
		Incongruent: 873.49 (164.11)	Incongruent: 0.08 (0.10)
		Stroop Effect: 135.40(70.32)	Stroop Effect: 0.03 (0.04)
	PC-50	Congruent: 750.56 (139.87)	Congruent: 0.04 (0.06)
		Incongruent: 868.87 (149.02)	Incongruent: 0.06 (0.07)
		Stroop Effect: 118.31 (72.26)	Stroop Effect: 0.02 (0.03)
Proactive	Biased	Congruent: 727.64 (135.23)	Congruent: 0.01 (0.04)
		Incongruent: 805.44 (134.19)	Incongruent: 0.03 (0.05)
		Stroop Effect: 77.80 (58.07)	Stroop Effect: 0.01 (0.02)
	PC-50	Congruent: 730.44 (129.50)	Congruent: 0.02 (0.04)
		Incongruent: 821.36 (131.43)	Incongruent: 0.03 (0.04)
		Stroop Effect: 90.92 (60.00)	Stroop Effect: 0.01 (0.03)
Reactive	Biased	Congruent: 696.55 (127.49)	Congruent: 0.01 (0.03)
		Incongruent: 775.76 (131.43)	Incongruent: 0.03 (0.05)
		MC Filler: xxx	MC Filler: xxx
		Stroop Effect: 79.22 (49.73)	Stroop Effect: 0.02 (0.03)
	PC-50	Congruent: 717.89 (125.88)	Congruent: 0.01 (0.03)
		Incongruent: 823.20 (128.54)	Incongruent: 0.04 (0.05)
		Stroop Effect: 105.32 (67.35)	Stroop Effect: 0.03 (0.04)

Condition	Derived Measure	Trial Type	RT Mean (SD)
Proactive	Congruency Cost	Biased	-20.12 (103.63)
		PC-50	-10.45 (118.73)
	Transfer Cost	–	13.13 (42.33)
Reactive	Congruency Cost	Biased	-41.54 (130.30)
		PC-50	-32.67 (115.78)
	Transfer Cost	–	26.10 (67.29)

Supplemental Table 2.4: 2020 (Test only) Cued-TS Results Across Trial Types and Conditions

Condition	Trial Type	RT Mean (SD)	Error Mean (SD)
Baseline	Biased (Non-Incentivized)	Congruent: 988.57 (333.46)	Congruent: 0.04 (0.05)
		Incongruent: 1030.87 (351.43)	Incongruent: 0.10 (0.11)
		TRCE: 42.49 (130.54)	TRCE: 0.06 (0.10)
	Unbiased (Non-Incentivized)	Congruent: 1037.29 (348.21)	Congruent: 0.05 (0.06)
		Incongruent: 1080.78 (332.16)	Incongruent: 0.11 (0.09)
		TRCE: 43.49 (102.37)	TRCE: 0.07 (0.08)
Proactive	Biased (Non-Incentivized)	Congruent: 715.64 (196.20)	Congruent: 0.05 (0.05)
		Incongruent: 740.98 (193.64)	Incongruent: 0.14 (0.13)
		TRCE: 25.34 (68.55)	TRCE: 0.09 (0.13)
	Unbiased (Incentivized)	Congruent: 707.62 (198.08)	Congruent: 0.06 (0.08)
		Incongruent: 732.09 (180.82)	Incongruent: 0.15 (0.09)
		TRCE: 24.48 (71.32)	TRCE: 0.08 (0.09)
Reactive	Biased (Non-Incentivized)	Congruent: 900.25 (309.46)	Congruent: 0.02 (0.03)
		Incongruent: 957.13 (319.86)	Incongruent: 0.08 (0.12)
		TRCE: 56.88 (117.14)	TRCE: 0.06 (0.10)
	Unbiased (Incentivized)	Congruent: 975.37 (330.47)	Congruent: 0.03 (0.07)
		Incongruent: 1025.31 (330.10)	Incongruent: 0.08 (0.09)
		TRCE: 49.94 (122.83)	TRCE: 0.05 (0.08)

Supplemental Table 3.1: Combined (2018 + 2020) AX-CPT Results Across Trial Types and Conditions

Condition	Trial Type	RT Mean (SD)	Error Mean(SD)	
Baseline	AX	450.41 (69.53)	0.06 (0.07)	
	AY	530.90 (76.77)	0.06 (0.07)	
	A-nogo	–	0.12 (0.10)	
	BX	499.73 (107.63)	0.14 (0.15)	
	BY	447.00 (63.15)	0.01 (0.01)	
	B-nogo	–	0.22 (0.15)	
Proactive	AX	413.71 (76.02)	0.05 (0.09)	
	AY	527.22 (84.60)	0.17 (0.17)	
	A-nogo	–	0.17 (0.17)	
	BX	452.12 (106.99)	0.11 (0.12)	
	BY	413.49 (70.59)	0.01 (0.01)	
	B-nogo	–	0.33 (0.22)	
Reactive	AX	428.42 (80.64)	0.07 (0.06)	
	AY	545.77 (87.22)	0.07 (0.08)	
	A-nogo	–	0.09 (0.07)	
	BX	519.88 (100.47)	0.09 (0.12)	
	BY	418.39 (71.06)	0.01 (0.01)	
	B-nogo	–	0.13 (0.10)	
Condition	Derived Measure	RT Mean (SD)	Error Mean (SD)	Z-scores Mean (SD)
Baseline	A-cue Bias	–	–	0.07 (0.30)
	BX Interference	52.74 (65.48)	0.15 (0.14)	–
	d'-context	–	–	2.84 (0.82)
	PBI	0.04 (0.07)	-0.25 (0.48)	–
Proactive	A-cue Bias	–	–	0.37 (0.42)
	BX Interference	38.63 (59.34)	0.11 (0.11)	–
	d'-context	–	–	3.12 (0.90)
	PBI	0.08 (0.08)	0.15 (0.54)	–
Reactive	A-cue Bias	–	–	0.07 (0.31)

BX Interference	101.49 (59.21)	0.10 (0.11)	–
d’-context	–	–	3.04 (0.87)
PBI	0.03 (0.06)	-0.04 (0.50)	–

Supplemental Table 3.2: Combined (2018 + 2020) Sternberg Results Across Trial Types and Conditions

Condition	Trial Type	RT Mean (SD)	Error Mean (SD)
Baseline	NN	Critical: 835.71 (154.26)	Critical: 0.04 (0.06)
		High: 884.78 (159.55)	High: 0.08 (0.07)
	NP	Critical: 881.44 (164.93)	Critical: 0.13 (0.12)
		High: 902.27 (167.74)	High: 0.20 (0.11)
	RN	Critical: 955.82 (180.20)	Critical: 0.19 (0.17)
		High: 1011.50 (203.43)	High: 0.44 (0.22)
Proactive	NN	Critical: 834.94 (161.61)	Critical: 0.05 (0.07)
		Low: 813.86 (163.22)	Low: 0.04 (0.06)
	NP	Critical: 852.59 (157.34)	Critical: 0.12 (0.11)
		Low: 788.17 (155.90)	Low: 0.05 (0.06)
	RN	Critical: 988.73 (194.89)	Critical: 0.26 (0.20)
		Low: 861.70 (189.56)	Low: 0.06 (0.13)
Reactive	NN	Critical: 839.09 (165.41)	Critical: 0.04 (0.08)
		High: 890.67 (177.90)	High: 0.07 (0.13)
	NP	Critical: 850.56 (155.38)	Critical: 0.11 (0.10)
		High: 889.64 (151.43)	High: 0.21 (0.11)
	RN	Critical: 919.30 (163.68)	Critical: 0.12 (0.12)
		High: 985.13 (162.22)	High: 0.28 (0.15)
**Condition**	**Derived Measure**	**RT Mean (SD)**	**Error Mean (SD)**
Baseline	Recent Negative Effect	Critical: 120.11 (103.30)	Critical: 0.15 (0.15)
		Non-critical: 126.72 (134.97)	Non-critical: 0.37 (0.21)
Proactive		Critical: 153.80 (98.03)	Critical: 0.22 (0.18)
		Non-critical: 47.84 (98.90)	Non-critical: 0.02 (0.11)
Reactive		Critical: 80.20 (77.90)	Critical: 0.07 (0.11)
		Non-critical: 94.45 (108.40)	Non-critical: 0.20 (0.14)

Supplemental Table 3.3: Combined (2018 + 2020) Stroop Results Across Trial Types and Conditions

Condition	Trial Type	RT Mean (SD)	Error Mean (SD)
Baseline	Biased	Congruent: 700.53 (134.95)	Congruent: 0.04 (0.07)
		Incongruent: 834.53 (149.27)	Incongruent: 0.06 (0.08)
		Stroop Effect: 134.00 (64.24)	Stroop Effect: 0.03 (0.04)
	PC-50	Congruent: 715.41 (126.76)	Congruent: 0.03 (0.06)
		Incongruent: 832.15 (137.91)	Incongruent: 0.05 (0.06)
		Stroop Effect: 116.74 (65.77)	Stroop Effect: 0.02 (0.03)
Proactive	Biased	Congruent: 705.30 (131.95)	Congruent: 0.01 (0.04)
		Incongruent: 785.47 (137.22)	Incongruent: 0.03 (0.04)
		Stroop Effect: 80.17 (52.13)	Stroop Effect: 0.02 (0.02)
	PC-50	Congruent: 709.96 (127.44)	Congruent: 0.01 (0.04)
		Incongruent: 804.10 (132.09)	Incongruent: 0.03 (0.04)
		Stroop Effect: 94.14 (58.26)	Stroop Effect: 0.01 (0.02)
Reactive	Biased	Congruent: 676.97 (123.88)	Congruent: 0.01 (0.03)
		Incongruent: 763.19 (129.60)	Incongruent: 0.03 (0.04)
		MC Filler: xxxx	MC Filler: xxxxx
		Stroop Effect: 86.22 (49.84)	Stroop Effect: 0.02 (0.03)
	PC-50	Congruent: 699.34 (120.75)	Congruent: 0.01 (0.04)
		Incongruent: 811.98 (127.26)	Incongruent: 0.04 (0.05)
		Stroop Effect: 112.64 (64.25)	Stroop Effect: 0.03 (0.04)

Condition	Derived Measure	Trial Type	RT Mean (SD)
Proactive	Congruency Cost	Biased	4.77 (103.02)
		PC-50	-5.45 (90.03)
	Transfer Cost	–	13.97 (38.69)
Reactive	Congruency Cost	Biased	-23.56 (110.65)
		PC-50	-16.06 (98.26)
	Transfer Cost	–	26.42 (54.85)

Supplemental Table 3.4: Combined (2018 + 2020) Cued-TS Results Across Trial Types and Conditions

Condition	Trial Type	RT Mean (SD)	Error Mean (SD)
Baseline	Biased (Non-Incentivized)	Congruent: 905.52 (259.69)<br>Incongruent: 979.61 (293.06)<br>TRCE: 74.09 (126.23)	Congruent: 0.03 (0.04)<br>Incongruent: 0.10 (0.10)<br>TRCE: 0.07 (0.09)
	Unbiased (Non-Incentivized)	Congruent: 945.57 (285.43)<br>Incongruent: 1021.05 (289.48)<br>TRCE: 75.48 (112.59)	Congruent: 0.05 (0.05)<br>Incongruent: 0.13 (0.09)<br>TRCE: 0.08 (0.08)
Proactive	Biased (Non-Incentivized)	Congruent: 710.71 (186.57)<br>Incongruent: 769.73 (227.30)<br>TRCE: 59.02 (100.03)	Congruent: 0.04 (0.04)<br>Incongruent: 0.15 (0.11)<br>TRCE: 0.11 (0.11)
	Unbiased (Incentivized)	Congruent: 689.89 (199.48)<br>Incongruent: 738.88 (217.82)<br>TRCE: 48.99 (77.47)	Congruent: 0.05 (0.06)<br>Incongruent: 0.14 (0.08)<br>TRCE: 0.09 (0.09)
Reactive	Biased (Non-Incentivized)	Congruent: 989.14 (315.58)<br>Incongruent: 1079.42 (351.15)<br>TRCE: 90.28 (153.15)	Congruent: 0.01 (0.02)<br>Incongruent: 0.06 (0.08)<br>TRCE: 0.05 (0.07)
	Unbiased (Incentivized)	Congruent: 1130.01 (390.91)<br>Incongruent: 1193.69 (379.68)<br>TRCE: 63.67 (133.68)	Congruent: 0.01 (0.03)<br>Incongruent: 0.06 (0.06)<br>TRCE: 0.05 (0.07)

The collected MTurk data includes two round of data collection (in 2018 and 2020 respectively). Hierarchical Bayesian Regression (HBR) models are fit onto each data set to analyze the contrasting effect of mode (Baseline, Proactive and Reactive) on behavioral indices of cognitive control.

While models for both data sets collected the parameter estimates of interest and ensured their significance or lack thereof, the 2020 Bayesian MLMs additionally incorporate the 2018 estimates as informed priors. The advantage of including these priors is the direct usage of the collected 2018 data to generate more valid parameter estimates that elucidate the true relationship between mode and the relevant behavioral indices. Savage-Dickey Density ratios (SDRs) were collected in this data set to determine if the prior and posterior density distributions for parameters of interest significantly differed from one another.

2 AX-CPT

While the below sections will go into more detail for each AX-CPT indicator, we briefly describe the expected results for differences in performance across conditions.

A-Cue Bias: We predict that subjects will have 1) greater A-Cue bias in the Proactive versus Baseline mode, 2) greater A-Cue bias in the Proactive versus Reactive mode, and 3) strong A-Cue bias only in the Proactive mode.

BX Error Interference: We predict that subjects will have 1) reduction of BX error interference in the Proactive versus Baseline mode, and 2) reduction of BX interference in the Reactive versus Baseline mode.

BX RT Interference: We predict that subjects will have 1) reduction of BX RT interference in the Proactive versus Baseline mode, 2) increased BX RT interference in the Reactive versus Baseline mode, and 3) increased BX RT interference in the Reactive versus Proactive mode.

2.1 A-Cue Bias 2018

This section reports results on A-Cue Bias, the likelihood of making a target response for AX and AY trials. A-Cue bias is modeled as the log odds of making a target response for AX and AY trials. The AXCPT data is filtered to only include AX and AY trials. This categorical variable 'trialType' is therefore effect coded to highlight how the aggregate of AX and AY target response proportions changed across mode. The categorical variable 'mode' is dummy coded to show direct comparisons between modes [baseline and reactive A-Cue bias are used as the intercept].

Wilkinson Notation

$$\text{Target Response} = \text{mode } x \text{ Trial Type} + \left(1 + \text{Trial Type} \mid \text{ID}\right), \text{ family}\left(\text{binomial}\right)$$

Fully Indexed Notation

$$\begin{gathered}
\text{Target Response}_{i,t} \sim \text{Bernoulli}\left(p_{i,t}\right) \\
\text{logit}\left(p_{i,t}\right) = \beta_{0i} + \beta_1 * mode_t \ x \ \beta_{2i} * trialType_{i,t} \\
\begin{bmatrix}\beta_{0i}\\ \beta_{2i}\end{bmatrix} \sim \text{N}\left(\begin{bmatrix}\beta_{0}\\ \beta_{2}\end{bmatrix}, \Sigma\right) \\
\beta_0, \beta_1, \beta_2 \sim \text{flat}, \Sigma \sim \text{LKJ}(1)
\end{gathered}$$

2.1.1 Proactive - Baseline A-Cue Bias

Table 1: Proactive - Baseline A-Cue Bias

Term	Estimate	SE	HDI	pd
Baseline A-cue	0.0622	0.0721	[-0.08, 0.2]	80.8%
Baseline AY	-3.0725	0.0734	[-3.21, -2.92]	100%
Proactive A-cue	0.7432	0.0452	[0.65, 0.83]	100%
AY x Proactive	0.5204	0.0452	[0.43, 0.61]	100%

Note. The intercept term 'Baseline A-Cue' refers to the average log odds to make a target response for Baseline A-Cue trials. 'Proactive A-Cue' is the key effect and refers to the difference in log odds to make a target response for A-Cue trials in the Proactive mode.

When contrasting A-Cue bias by mode, there is decisive evidence that the A-Cue bias is greater in the Proactive versus Baseline mode (β = 0.74, se = 0.05, HDI = [0.65, 0.83], pd = 100%). The pd suggests 100% likelihood that the effect is greater than zero.

2.1.2 Proactive - Reactive A-Cue Bias

Table 2: Proactive - Reactive A-Cue Bias

Term	Estimate	SE	HDI	pd
Reactive A-cue	-0.0002	0.0678	[-0.14, 0.13]	50.54%
Reactive AY	-2.9391	0.0702	[-3.08, -2.8]	100%
Proactive A-cue	0.8120	0.0441	[0.73, 0.9]	100%
AY x Proactive	0.4143	0.0446	[0.32, 0.5]	100%

Note. The intercept term 'Reactive A-Cue' refers to the average log odds to make a target response for Reactive A-Cue trials. 'Proactive A-Cue' is the key effect and refers to the difference in log odds to make a target response for A-Cue trials in the Proactive mode.

There is decisive evidence that A-Cue bias is greater in the Proactive versus Reactive mode (β = 0.81, se = 0.04, HDI = [0.73, 0.9], pd = 100%).

2.1.3 A-Cue Bias

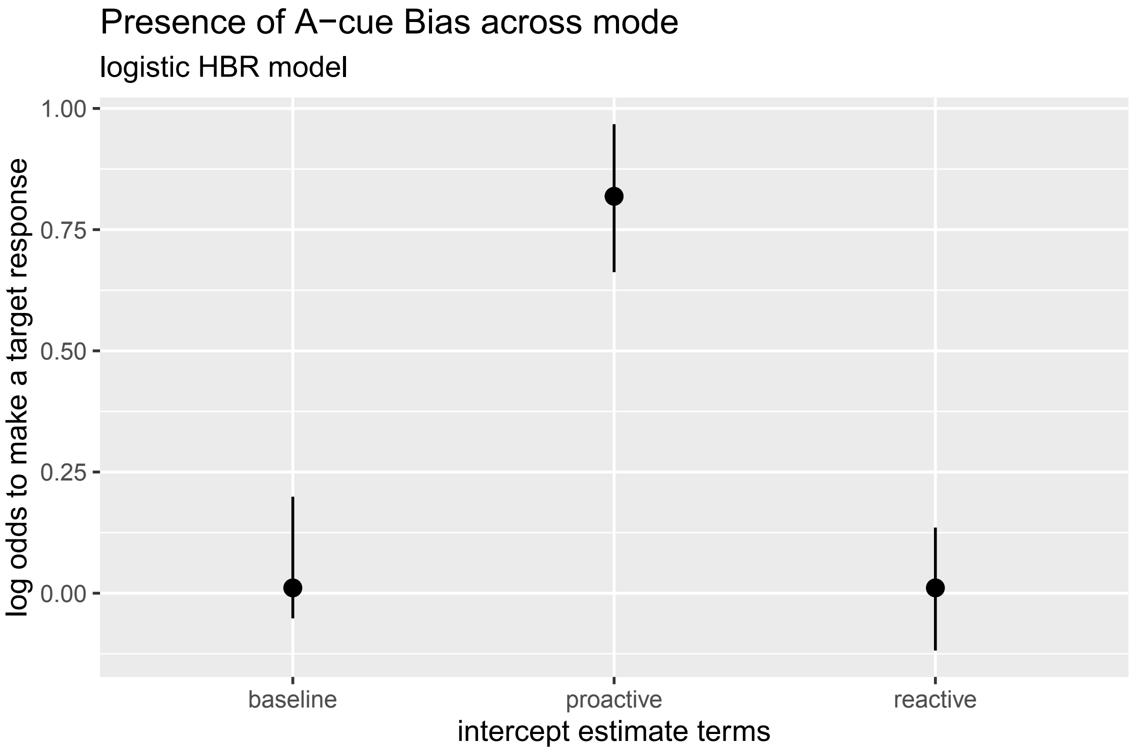

Table 3: A-Cue Bias

Term	Estimate	SE	HDI	pd
Proactive A-Cue	0.8188	0.0788	[0.66, 0.97]	100%
Baseline A-Cue	0.0784	0.0640	[-0.05, 0.2]	88.95%
Reactive A-Cue	0.0110	0.0649	[-0.12, 0.14]	57.41%

Note. Each column is represented by the A-Cue bias term for each mode.

As was the case for Tang et al., 2023, A-Cue bias is decisively positive in the Proactive mode (β = 0.82, se = 0.08, HDI = [0.66, 0.97], pd = 100%) but not in the Baseline (β = 0.08, se = 0.06, HDI = [-0.05, 0.2], pd = 88.95%) or Reactive modes (β = 0.01, se = 0.06, HDI = [-0.12, 0.14], pd = 57.41%).

2.2 A-Cue Bias 2020

These analyses test for a consistent pattern in the 2020 dataset, using the 2018 estimates as informative priors.

2.2.1 Proactive - Baseline A-Cue Bias

Table 1: Proactive - Baseline A-Cue Bias

Term	Estimate	SE	HDI	pd
Baseline A-cue	0.0378	0.0543	[-0.07, 0.14]	75.6%
Baseline AY	-3.1300	0.0526	[-3.24, -3.03]	100%
Proactive A-cue	0.6766	0.0360	[0.61, 0.75]	100%
AY x Proactive	0.5459	0.0344	[0.48, 0.62]	100%

Note. The intercept term 'Baseline A-Cue' refers to the average log odds to make a target response for Baseline A-Cue trials. 'Proactive A-Cue' is the key effect and refers to the difference in log odds to make a target response for A-Cue trials in the Proactive mode.

A-Cue bias is shown to be significantly greater in the Proactive mode relative to the Baseline mode (β = 0.68, se = 0.04, HDI = [0.61, 0.75], pd = 100%). The pd suggests 100% likelihood that the effect is greater than zero.

Table 2: Hypothesis Test for Pro-Bas A-Cue Bias

Hypothesis	Estimate	SE	HDI	SDR	Post.Prob
Proactive - Baseline A-Cue bias	-0.07	0.04	[0.61, 0.75]	0.22	0.00

Note. The key term in this table is the SDR score, which is computed as the ratio of the posterior distribution to the prior distribution at a specific point, in this case the mean of the prior distribution to investigate whether there is an increased likelihood of this value as a function of incoming data.

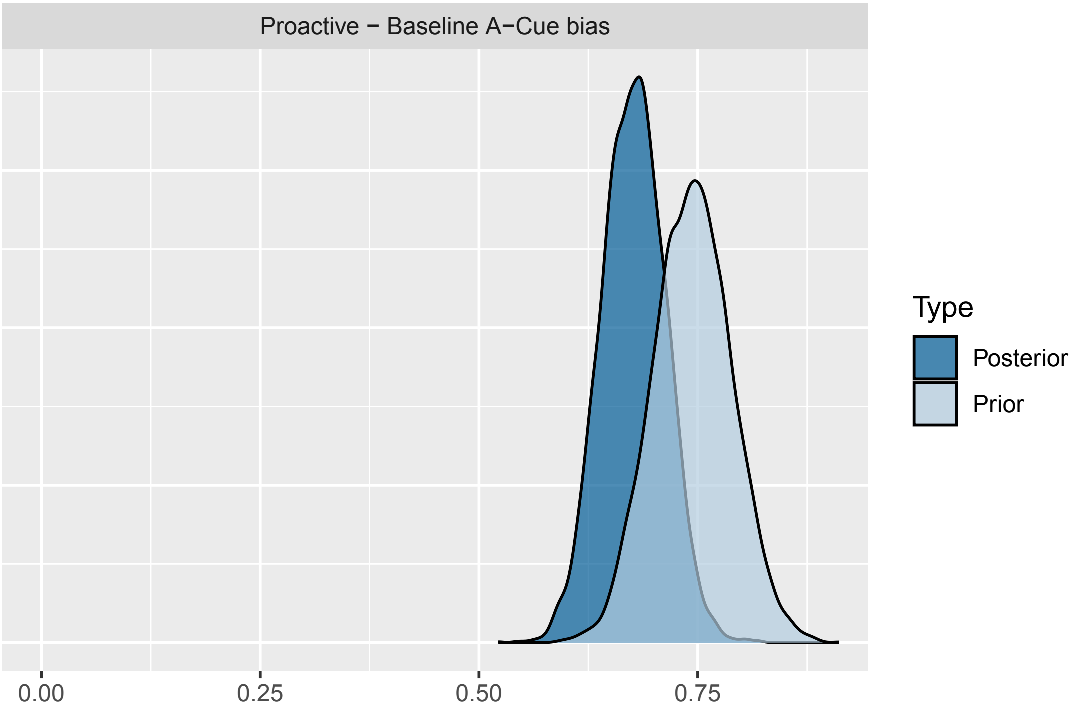

The plot indicates that the posterior is shifted away from the prior. The graph is centered at the mean value of 2018 estimate, and indicates the SDR to be less than one (0.22) at that point. This suggests that the prior overestimates the difference between Proactive and Baseline A-Cue bias. More generally, the pattern across the 2018 and 2020 datasets confirms the Proactive > Baseline A-cue Bias effect from Tang et al (2023).

2.2.2 Proactive - Reactive A-Cue Bias

Table 3: Proactive - Reactive A-Cue Bias

Term	Estimate	SE	HDI	pd
Reactive A-cue	0.0104	0.0495	[-0.09, 0.11]	58.38%
Reactive AY	-2.9806	0.0531	[-3.08, -2.87]	100%
Proactive A-cue	0.6920	0.0341	[0.63, 0.76]	100%
AY x Proactive	0.4202	0.0347	[0.35, 0.48]	100%

Note. The intercept term 'Reactive A-Cue' refers to the average log odds to make a target response for Reactive A-Cue trials. 'Proactive A-Cue' is the key effect and refers to the difference in log odds to make a target response for A-Cue trials in the Proactive mode.

A-Cue bias was shown to be significantly greater in the Proactive mode relative to the Reactive mode (β = 0.42, se = 0.03, HDI = [0.35, 0.48], pd = 100%).The pd suggests 100% likelihood that the effect is greater than zero.

Table 4: Hypothesis Test for Pro-Rea A-Cue Bias

Hypothesis	Estimate	SE	HDI	SDR	Post.Prob
Proactive - Reactive A-Cue bias	-0.12	0.03	[0.63, 0.76]	0.01	0.00

Note. The key term in this table is the SDR score, which is computed as the ratio of the posterior distribution to the prior distribution at a specific point, in this case the mean of the prior distribution to investigate whether there is an increased likelihood of this value as a function of incoming data.

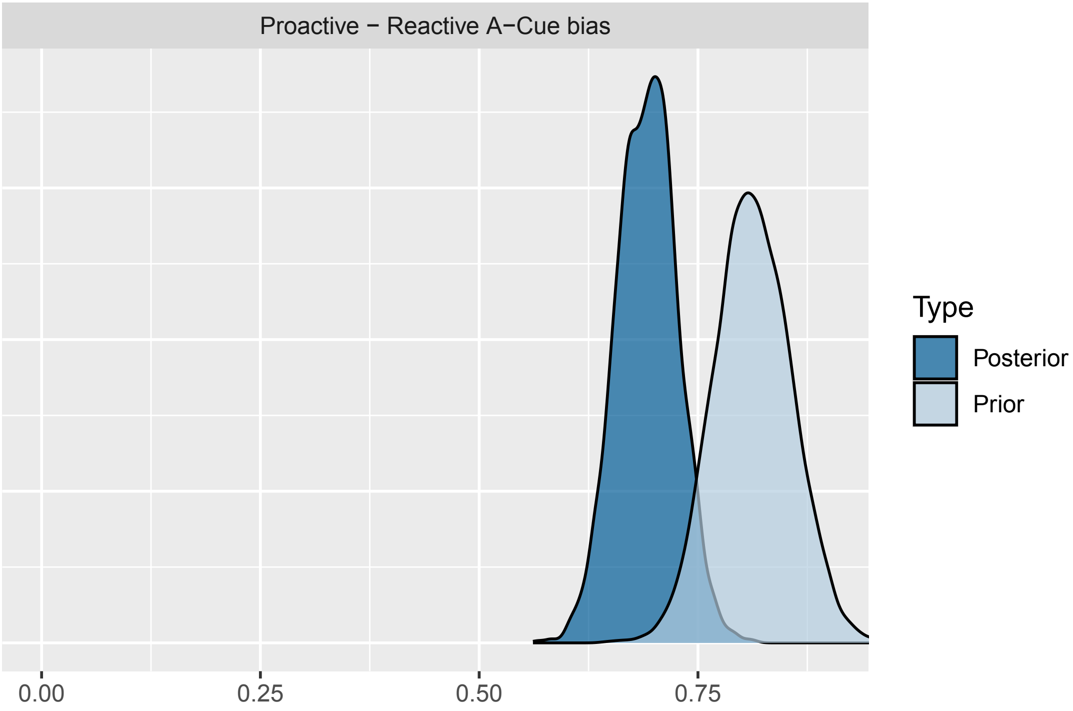

The plot indicates that the posterior is shifted away from the prior distribution. The graph is centered at the mean value of 2018 estimate, and indicates the SDR to be less than one (0.01) at that point. This suggests that the prior overestimates the difference between Proactive and Reactive A-Cue bias. More generally, the pattern across the 2018 and 2020 datasets confirms the Proactive > Reactive A-cue Bias effect from Tang et al (2023).

2.2.3 A-Cue Bias

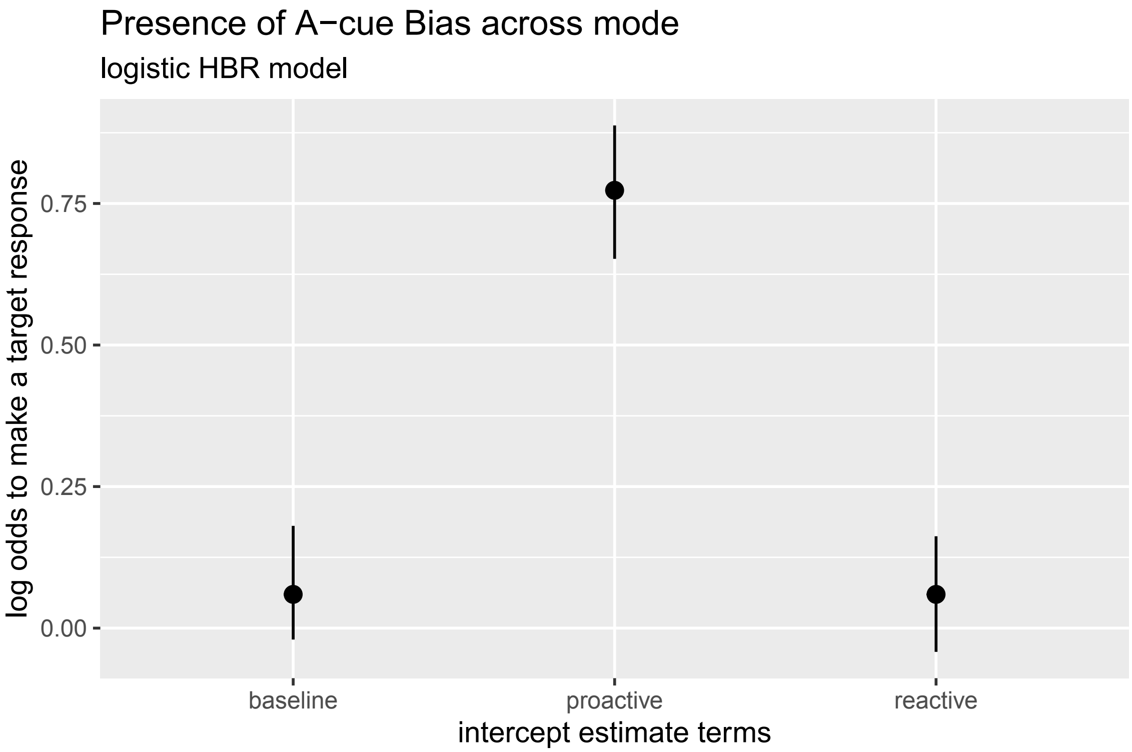

Table 5: A-Cue Bias

Term	Estimate	SE	HDI	pd
Proactive A-Cue	0.7733	0.0604	[0.65, 0.89]	100%
Baseline A-Cue	0.0836	0.0515	[-0.02, 0.18]	94.5%
Reactive A-Cue	0.0596	0.0510	[-0.04, 0.16]	88.35%

Note. Each column is represented by the A-Cue bias term for each mode.

A-Cue bias was strongly positive in the Proactive mode (β = 0.77, se = 0.06, HDI = [0.65, 0.89], pd = 100%) but not in the Baseline (β = 0.08, se = 0.05, HDI = [-0.02, 0.18], pd = 94.5%) or Reactive modes (β = 0.06, se = 0.05, HDI = [-0.04, 0.16], pd = 88.35%).

Table 6: Hypothesis Test for Pro A-Cue Bias

Hypothesis	Estimate	SE	HDI	SDR	Post.Prob
(Intercept)-(.8188) = 0	-0.05	0.06	[0.65, 0.89]	1.01	1.00

Note. The key term in this table is the SDR score, which is computed as the ratio of the posterior distribution to the prior distribution at a specific point, in this case the mean of the prior distribution to investigate whether there is an increased likelihood of this value as a function of incoming data.

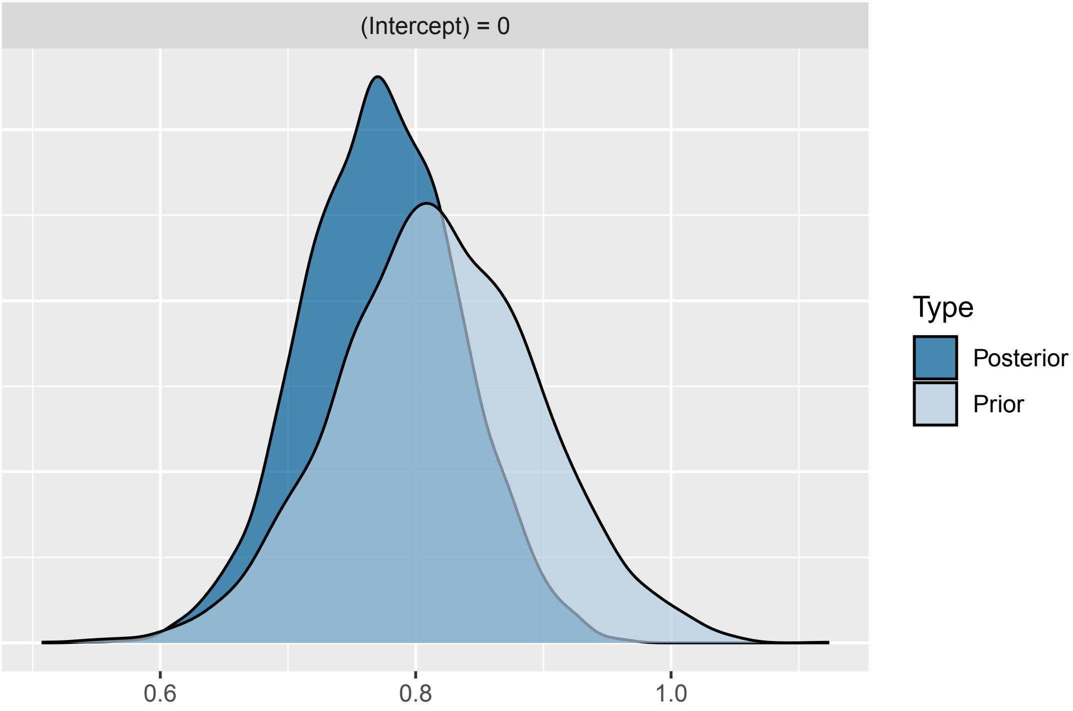

The plot indicates that the posterior closely overlaps the prior distribution. The graph is centered at the mean value of 2018 estimate, and the SDR is found to be greater than one (1.01) at that point. This suggests that the general estimate Proactive A-Cue bias does not significantly differ across samples. More generally, the pattern across the 2018 and 2020 datasets confirms the Proactive > 0 A-cue bias effect from Tang et al (2023)

Table 7: Hypothesis Test for Bas A-Cue Bias

Hypothesis	Estimate	SE	HDI	SDR	Post.Prob
(Intercept)-(.0784) = 0	0.01	0.05	[-0.02, 0.18]	1.26	1.00

Note. The key term in this table is the SDR score, which is computed as the ratio of the posterior distribution to the prior distribution at a specific point, in this case the mean of the prior distribution to investigate whether there is an increased likelihood of this value as a function of incoming data.

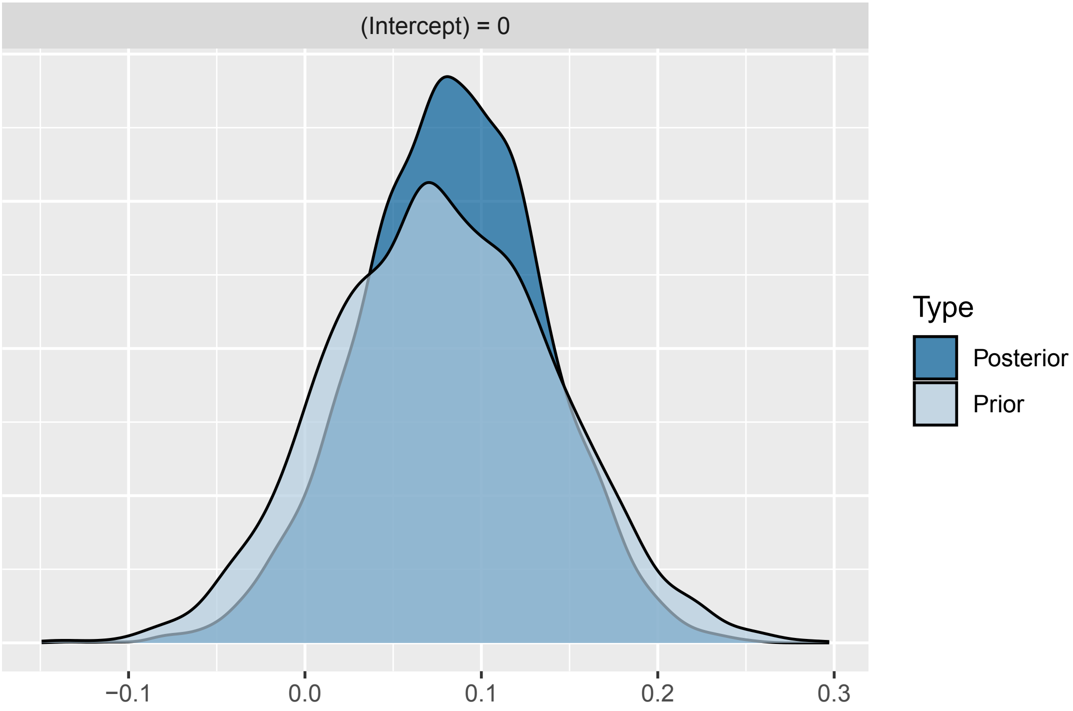

The plot indicates that the posterior closely overlaps the prior distribution. The graph is centered at the mean value of 2018 estimate, and the SDR is found to be greater than one (1.26) at that point. This suggests that the general estimate Baseline A-Cue bias does not significantly differ across samples. More generally, the pattern across the 2018 and 2020 datasets indicates that the Baseline A-cue bias effect is significantly greater than zero.

Table 8: Hypothesis Test for Rea A-Cue Bias

Hypothesis	Estimate	SE	HDI	SDR	Post.Prob
(Intercept)-(.011) = 0	0.05	0.05	[-0.04, 0.16]	0.82	0.00

Note. The key term in this table is the SDR score, which is computed as the ratio of the posterior distribution to the prior distribution at a specific point, in this case the mean of the prior distribution to investigate whether there is an increased likelihood of this value as a function of incoming data.

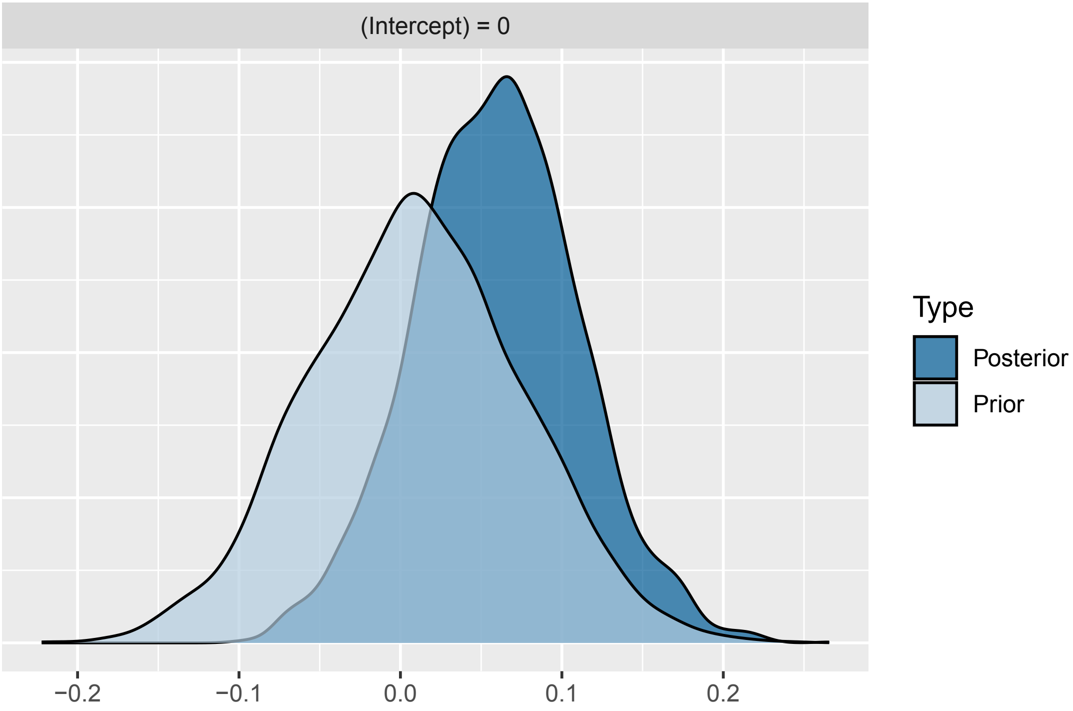

The plot indicates that the posterior is shifted away from the prior distribution. The graph is centered at the mean value of the 2018 estimate, and indicates the SDR to be less than one (0.82) at that point. This suggests that Reactive A-Cue bias is underestimated in the 2018 sample. More generally, the pattern across the 2018 and 2020 datasets confirms the pattern from Tang et al (2023), that the Reactive A-cue bias effect is not reliably different from zero.

2.3 BX Error Interference 2018

This section reports results on BX error interference, the relative likelihood of making an error for BX versus BY trials. BX error interference is modeled as the log odds of making an incorrect response for BX versus BY trials. The AX-CPT data is filtered to only include BX and BY trials. This categorical variable 'trialType' is dummy coded to see differences in performance between BX and BY trials. The categorical variable 'mode' is dummy coded to compare modes [baseline and reactive BY trials are used as the intercept]. The interaction effect between trial type and mode (i.e., trialType*mode) is the key parameter of interest to examine the difference in BX error interference between modes.

Wilkinson Notation

$$\text{Correct} = \text{mode } x \text{ Trial Type} + \left(1 + \text{ mode } x \text{ Trial Type} \mid \text{ID}\right), \text{ family}\left(\text{binomial}\right)$$

Fully Indexed Notation

$$\begin{aligned}
\text{Correct}_{i,t} &\sim \text{Bernoulli}\left(p_{i,t}\right)\\
\text{logit}\left(p_{i,t}\right) &= \beta_{0i} + \beta_{1i} * mode_{i,t} \; x \; \beta_{2i} * trialType_{i,t}\\
\begin{bmatrix}\beta_{0i}\\ \beta_{1i}\\ \beta_{2i}\end{bmatrix} &\sim \text{N}\left(\begin{bmatrix}\beta_0\\ \beta_1\\ \beta_2\end{bmatrix}, \Sigma\right)\\
\beta_0, \beta_1, \beta_2 &\sim \text{flat}, \Sigma \sim \text{LKJ}(1)
\end{aligned}$$

2.3.1 Proactive - Baseline BX error interference

Table 1: Proactive - Baseline BX error reduction

Term	Estimate	SE	HDI	pd
Baseline BY	-5.1914	0.1671	[-5.51, -4.85]	100%
Baseline BX	3.0758	0.1558	[2.77, 3.38]	100%
Proactive BY	0.0034	0.1820	[-0.34, 0.38]	51.09%
Proactive x BX	-0.4574	0.1837	[-0.83, -0.1]	99.25%

Note. The intercept term 'Baseline BY' refers to the average log odds to make an error on Baseline BY trials. 'Proactive x BX' is the key effect and refers to the difference in log odds to make an error on BX trials in the Proactive mode versus the Baseline mode.

As was the case for Tang et al., 2023, there is strong evidence for a reduction in BX error interference for the Proactive versus Baseline mode (β = -0.46, se = 0.18, HDI = [-0.34, 0.38], pd = 99.25%).

2.3.2 Reactive - Baseline BX error interference

Table 2: Reactive - Baseline BX error interference

Term	Estimate	SE	HDI	pd
Baseline BY	-5.2313	0.1745	[-5.58, -4.89]	100%
Baseline BX	3.1131	0.1665	[2.8, 3.45]	100%
Reactive BY	-0.1031	0.2158	[-0.52, 0.32]	68.03%
Reactive x BX	-0.3246	0.2293	[-0.75, 0.15]	92.1%

Note. The intercept term 'Baseline BY' refers to the average log odds to make an error on Baseline BY trials. 'Reactive x BX' is the key effect and refers to the difference in log odds to make an error on BX trials in the Reactive mode versus the Baseline mode.

However, there is little evidence for a reduction in BX error interference for the Reactive versus Baseline mode (β = -0.32, se = 0.23, HDI = [-0.52, 0.32], pd = 92.1%).

2.4 BX Error Interference 2020

These analyses test for a consistent pattern in the 2020 dataset, using the 2018 estimates as informative priors.

2.4.1 Proactive - Baseline BX error interference

Table 1: Proactive - Baseline BX error interference

Term	Estimate	SE	HDI	pd
Baseline BY	-5.1559	0.1155	[-5.38, -4.93]	100%
Baseline BX	3.1318	0.1080	[2.92, 3.34]	100%
Proactive BY	0.0226	0.1285	[-0.22, 0.27]	56.96%
Proactive x BX	-0.4345	0.1275	[-0.7, -0.2]	99.97%

Note. The intercept term 'Baseline BY' refers to the average log odds to make an error on Baseline BY trials. 'BX x Proactive' is the key effect and refers to the difference in log odds to make an error on BX trials in the Proactive mode versus the Baseline mode.

There is strong evidence for a reduced BX error interference effect in the Proactive mode versus the Baseline mode (β = -0.43, se = 0.13, HDI = [-0.7, -0.2], pd = 99.97%).

Table 2: Hypothesis Test for Pro-Bas BX error interference

Hypothesis	Estimate	SE	HDI	SDR	Post.Prob
Proactive - Baseline BX error interference	0.02	0.13	[-0.7, -0.2]	1.44	1.00

Note. The key term in this table is the SDR score, which is computed as the ratio of the posterior distribution to the prior distribution at a specific point, in this case the mean of the prior distribution to investigate whether there is an increased likelihood of this value as a function of incoming data.

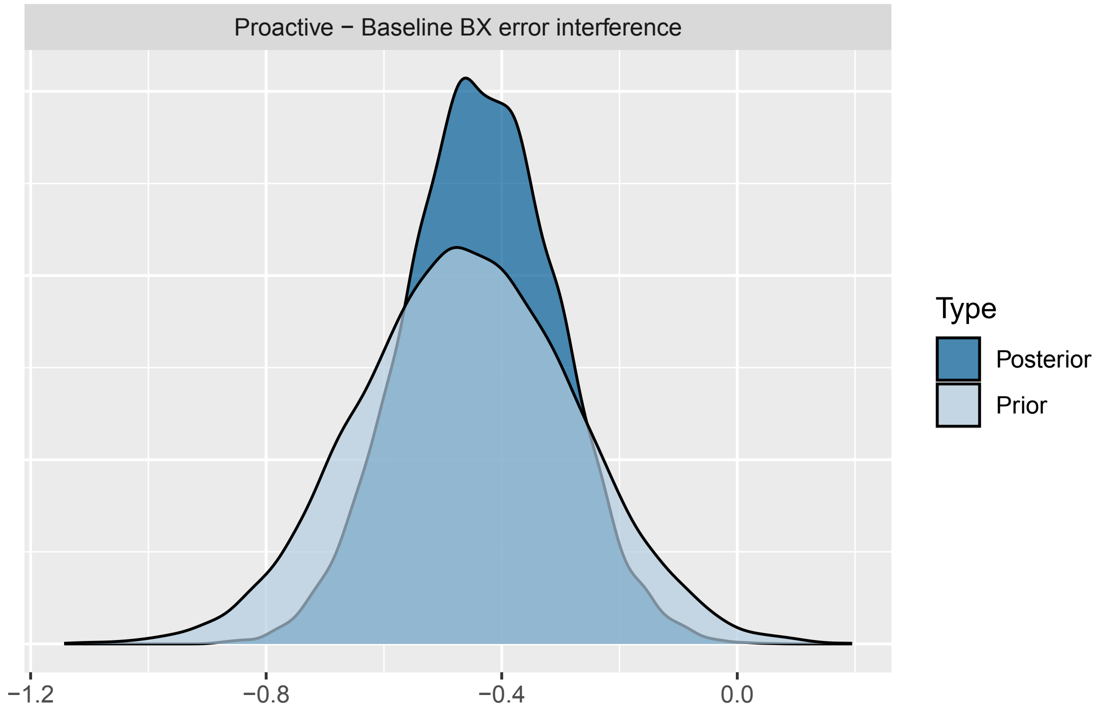

The plot indicates that the posterior closely overlaps the prior distribution. The graph is centered at the mean value of 2018 estimate, and the SDR is found to be greater than one (1.44) at that point. This suggests that the posterior values don't differ greatly from prior, showing that BX error reduction for the Proactive versus Baseline mode is stable across samples. More generally, the pattern across the 2018 and 2020 datasets confirms the Proactive < Baseline error interference pattern from Tang et al (2023).

2.4.2 Reactive - Baseline BX error interference

Table 3: Reactive - Baseline BX error interference

Term	Estimate	SE	HDI	pd
Baseline BY	-5.1935	0.1177	[-5.42, -4.96]	100%
Baseline BX	3.1298	0.1150	[2.9, 3.35]	100%
Reactive BY	-0.2063	0.1539	[-0.51, 0.09]	90.92%
Reactive x BX	-0.5182	0.1538	[-0.82, -0.22]	99.96%

Note. The intercept term 'Baseline BY' refers to the average log odds to make an error on Baseline BY trials. 'Reactive x BX' is the key effect and refers to the difference in log odds to make an error on BX trials in the Reactive mode versus the Baseline mode.

There is now decisive evidence for a reduced BX error interference effect in the Reactive mode versus the Baseline mode (β = -0.52, se = 0.15, HDI = [-0.82, -0.22], pd = 99.96%).

Table 4: Hypothesis Test for Rea-Bas BX error interference

Hypothesis	Estimate	SE	HDI	SDR	Post.Prob
Reactive - Baseline BX error interference	-0.20	0.15	[-0.82, -0.22]	0.66	0.00

Note. The key term in this table is the SDR score, which is computed as the ratio of the posterior distribution to the prior distribution at a specific point, in this case the mean of the prior distribution to investigate whether there is an increased likelihood of this value as a function of incoming data.

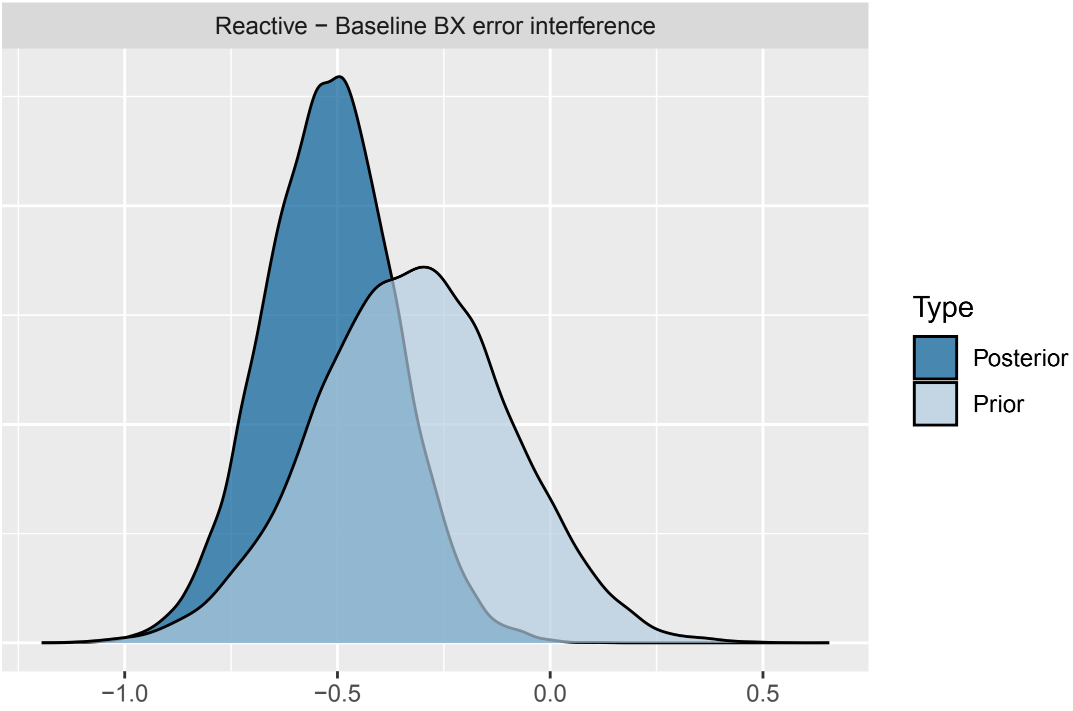

The plot indicates that the posterior is shifted away from the prior distribution. The graph is centered at the mean value of 2018 estimate, and indicates the SDR to be less than one (0.66) at that point. This suggests that the prior underestimates the difference between Reactive and Baseline BX error interference.

2.5 BX RT Interference 2018

This section reports results on BX RT interference, the relative slowing of correct responses for BX versus BY trials. BX RT interference is modeled as the [shifted log-normal] response time for correct responses on BX versus BY trials. The AX-CPT data is filtered to only include BX and BY trials. This categorical variable ‘trialType’ is dummy coded to see differences in performance between BX and BY trials. The categorical variable ‘mode’ is dummy coded to compare modes [baseline and reactive BY trials are used as the intercept]. The interaction effect between trial type and mode (i.e., trialType*mode) is the key parameter of interest to examine the difference in BX RT interference between modes.

Wilkinson Notation

$$\text{RT} = \text{mode } x \text{ Trial Type} + \left(1 + \text{Trial Type} \mid \text{ID}\right),\ \text{family}\left(\text{shifted log-normal}\right)$$

Fully Indexed Notation

$$\begin{gathered}
\text{RT}_{i,t} \sim \text{shifted log-normal}\left(\mu_{i,t}, \sigma, \theta\right) \\
\log\left(\mu_{i,t} + \theta\right) = \beta_{0i} + \beta_1 * mode_t \ x \ \beta_{2i} * trialType_{i,t} \\
\begin{bmatrix} \beta_{0i} \\ \beta_{2i} \end{bmatrix} \sim \text{N}\left(\begin{bmatrix} \beta_0 \\ \beta_2 \end{bmatrix}, \Sigma\right) \\
\beta_0, \beta_1, \beta_2 \sim \text{flat}, \Sigma \sim \text{LKJ(1)}, \sigma \sim \text{student-t(3, 0, 2.5)}, \theta \sim \text{uniform(0, minRT)}
\end{gathered}$$

2.5.1 Proactive - Baseline BX RT interference

Table 1: Proactive - Baseline BX RT interference

Term	Estimate	SE	HDI	pd
Baseline BY	5.8503	0.0175	[5.82, 5.88]	100%
Baseline BX	0.0937	0.0094	[0.08, 0.11]	100%
Proactive BY	-0.0974	0.0028	[-0.1, -0.09]	100%
BX x Proactive	-0.0309	0.0062	[-0.04, -0.02]	100%

Note. The intercept term 'Baseline BY' refers to the average [shifted log-normal] RT to make a correct response for Baseline BY trials. 'BX x Proactive' is the key effect and refers to the difference in [shifted log-normal] RT units to make a correct response for BX trials in the Proactive versus Baseline mode. Note that the log-normal RT units are much smaller than standard RT, which is usually given in milliseconds

There is decisive evidence for a reduced BX RT interference effect in the Proactive mode versus the Baseline mode (β = -0.03, se = 0.01, HDI = [-0.04, -0.02], pd = 100%).

2.5.2 Reactive - Baseline BX RT interference

Table 2: Reactive - Baseline BX RT interference

Term	Estimate	SE	HDI	pd
Baseline BY	5.8750	0.0175	[5.84, 5.91]	100%
Baseline BX	0.0929	0.0089	[0.08, 0.11]	100%
Reactive BY	-0.0607	0.0025	[-0.07, -0.06]	100%
Reactive x BX	0.1597	0.0060	[0.15, 0.17]	100%

Note. The intercept term 'Baseline BY' refers to the average [shifted log-normal] RT to make a correct response for Baseline BY trials. 'BX x Reactive' is the key effect and refers to the difference in [shifted log-normal] RT to make a correct response for BX trials in the Reactive versus Baseline mode.

In contrast, there is decisive evidence for a greater BX RT interference effect in the Reactive mode versus the Baseline mode (β = 0.16, se = 0.01, HDI = [0.15, 0.17], pd = 100%).

2.5.3 Reactive - Proactive BX RT interference

Table 3: Reactive - Proactive BX RT interference

Term	Estimate	SE	HDI	pd
Proactive BY	5.8222	0.0189	[5.79, 5.86]	100%
Proactive BX	0.0629	0.0083	[0.05, 0.08]	100%
Reactive BY	0.0327	0.0024	[0.03, 0.04]	100%
Reactive x BX	0.1833	0.0060	[0.17, 0.2]	100%

Note. The intercept term 'Proactive BY' refers to the average [shifted log-normal] RT to make a correct response for Proactive BY trials. 'Reactive x BX' is the key effect and refers to the difference in average [shifted log-normal] RT to make a correct response for BX trials in the Reactive versus Proactive mode.

Finally, there is decisive evidence for a greater BX RT interference effect in the Reactive versus Proactive mode (β = 0.18, se = 0.01, HDI = [0.17, 0.2], pd = 100%).

2.6 BX RT Interference 2020

These analyses test for a consistent pattern in the 2020 dataset, using the 2018 estimates as informative priors.

2.6.1 Proactive - Baseline BX RT interference

Table 1: Proactive - Baseline BX RT interference

Term	Estimate	SE	HDI	pd
Baseline BY	5.8531	0.0132	[5.83, 5.88]	100%
Baseline BX	0.1029	0.0074	[0.09, 0.12]	100%
Proactive BY	-0.1008	0.0022	[-0.11, -0.1]	100%
Proactive x BX	-0.0283	0.0050	[-0.04, -0.02]	100%

Note. The intercept term 'Baseline BY' refers to the average [shifted log-normal] RT to make a correct response for Baseline BY trials. 'Proactive x BX' is the key effect and refers to the difference in [shifted log-normal] RT to make a correct response for BX trials in the Proactive versus the Baseline mode.

There is decisive evidence for a reduced BX RT interference effect in the Proactive versus Baseline modes (β = -0.03, se = 0, HDI = [-0.04, -0.02], pd = 100%).

Table 2: Hypothesis Test for Pro-Bas BX RT interference

Hypothesis	Estimate	SE	HDI	SDR	Post.Prob
Proactive - Baseline BX RT interference	0.00	0.00	[-0.04, -0.02]	1.10	1.00

Note. The key term in this table is the SDR score, which is computed as the ratio of the posterior distribution to the prior distribution at a specific point, in this case the mean of the prior distribution to investigate whether there is an increased likelihood of this value as a function of incoming data.

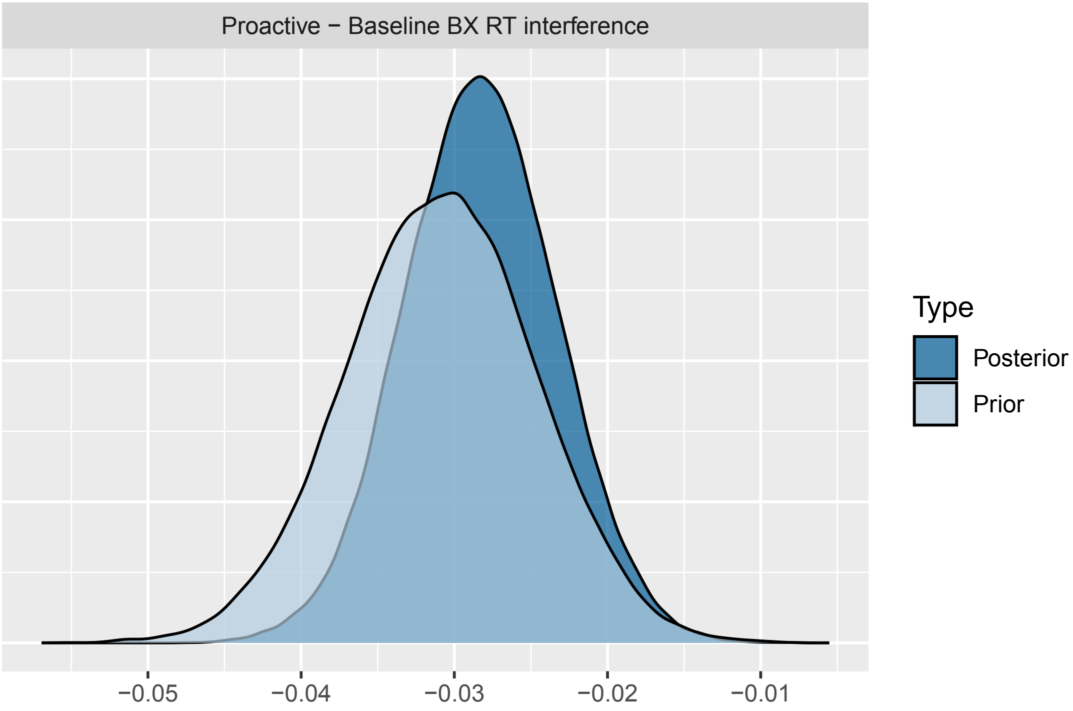

The plot indicates that the posterior closely overlaps the prior distribution. The graph is centered at the mean value of 2018 estimate, and the SDR is found to be greater than one (1.1) at that point. This suggests that the difference in the BX RT interference effect between the baseline and proactive modes does not significantly differ across samples. More generally, the pattern across the 2018 and 2020 datasets confirms the Proactive < Baseline BX RT interference pattern from Tang et al (2023).

2.6.2 Reactive - Baseline BX RT interference

Table 3: Reactive - Baseline BX RT interference

Term	Estimate	SE	HDI	pd
Baseline BY	5.9012	0.0136	[5.87, 5.93]	100%
Baseline BX	0.0955	0.0069	[0.08, 0.11]	100%
Reactive BY	-0.0802	0.0020	[-0.08, -0.08]	100%
Reactive x BX	0.1387	0.0047	[0.13, 0.15]	100%

Note. The intercept term 'Baseline BY' refers to the average [shifted log-normal] RT to make a correct response for Baseline BY trials. 'Reactive x BX' is the key effect and refers to the difference in [shifted log-normal] RT to make a correct response for BX trials in the Reactive mode versus the Baseline mode.

In contrast, there is decisive evidence for a BX RT interference effect in the Reactive mode versus Baseline mode (β = 0.14, se = 0, HDI = [0.13, 0.15], pd = 100%).

Table 4: Hypothesis Test for Rea-Bas BX RT interference

Hypothesis	Estimate	SE	HDI	SDR	Post.Prob
Reactive - Baseline BX RT interference	-0.02	0.00	[0.13, 0.15]	0.00	0.00

Note. The key term in this table is the SDR score, which is computed as the ratio of the posterior distribution to the prior distribution at a specific point, in this case the mean of the prior distribution to investigate whether there is an increased likelihood of this value as a function of incoming data.

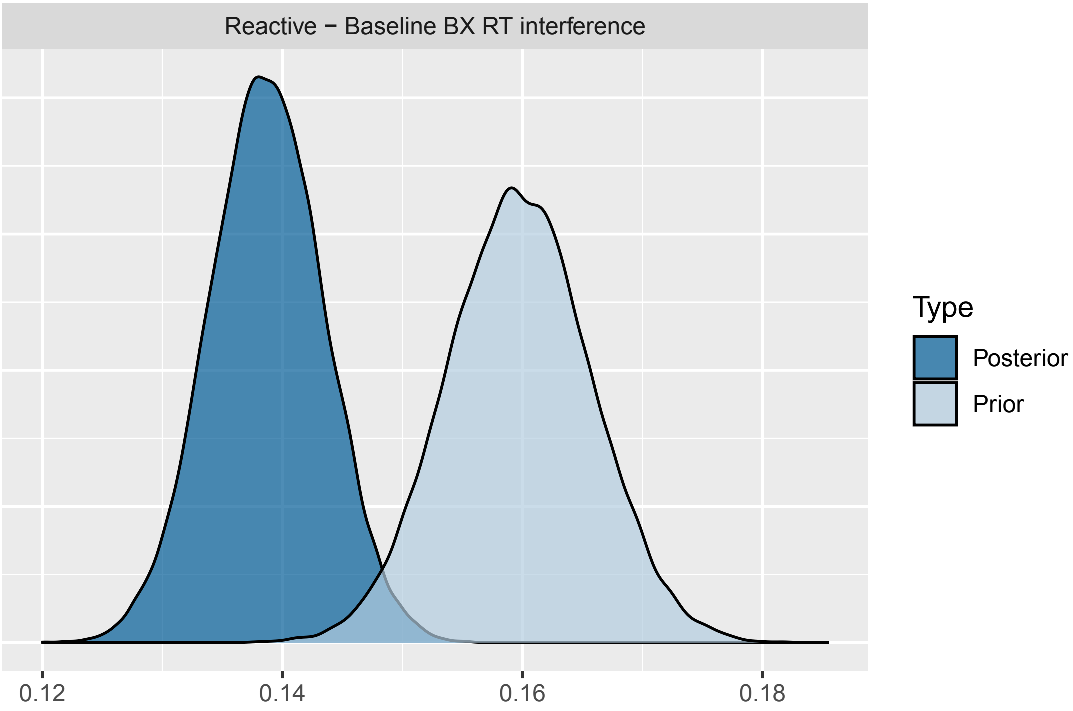

The plot indicates that the posterior is shifted away from the prior distribution. The graph is centered at the mean value of 2018 estimate, and indicates the SDR to be less than one (0) at that point. This suggests that the prior overestimates the difference between Reactive and Baseline BX RT interference. Nevertheless, the posterior distribution is also clearly shifted away from zero, indicating the reliability of the primary BX RT effect. More generally, the pattern across the 2018 and 2020 and datasets confirms the Reactive > Baseline BX RT interference pattern from Tang et al (2023).

2.6.3 Reactive - Proactive BX RT interference

Table 5: Reactive - Proactive BX RT interference

Term	Estimate	SE	HDI	pd
Proactive BY	5.8053	0.0144	[5.78, 5.83]	100%
Proactive BX	0.0721	0.0065	[0.06, 0.08]	100%
Reactive BY	0.0164	0.0020	[0.01, 0.02]	100%
Reactive x BX	0.1640	0.0048	[0.15, 0.17]	100%

Note. The intercept term 'Proactive BY' refers to the average [shifted log-normal] RT to make a correct response for Proactive BY trials. 'Reactive x BX' is the key effect and refers to the difference in [shifted log-normal] RT to make a correct response for BX trials in the Reactive mode versus the Proactive mode.

There was also decisive evidence for an increased BX RT interference effect in the Reactive mode versus the Proactive mode (β = 0.16, se = 0, HDI = [0.15, 0.17], pd = 100%).

Table 6: Hypothesis Test for Rea-Pro BX RT interference

Hypothesis	Estimate	SE	HDI	SDR	Post.Prob
Proactive - Reactive BX RT reduction	-0.02	0.00	[0.15, 0.17]	0.00	0.00

Note. The key term in this table is the SDR score, which is computed as the ratio of the posterior distribution to the prior distribution at a specific point, in this case the mean of the prior distribution to investigate whether there is an increased likelihood of this value as a function of incoming data.

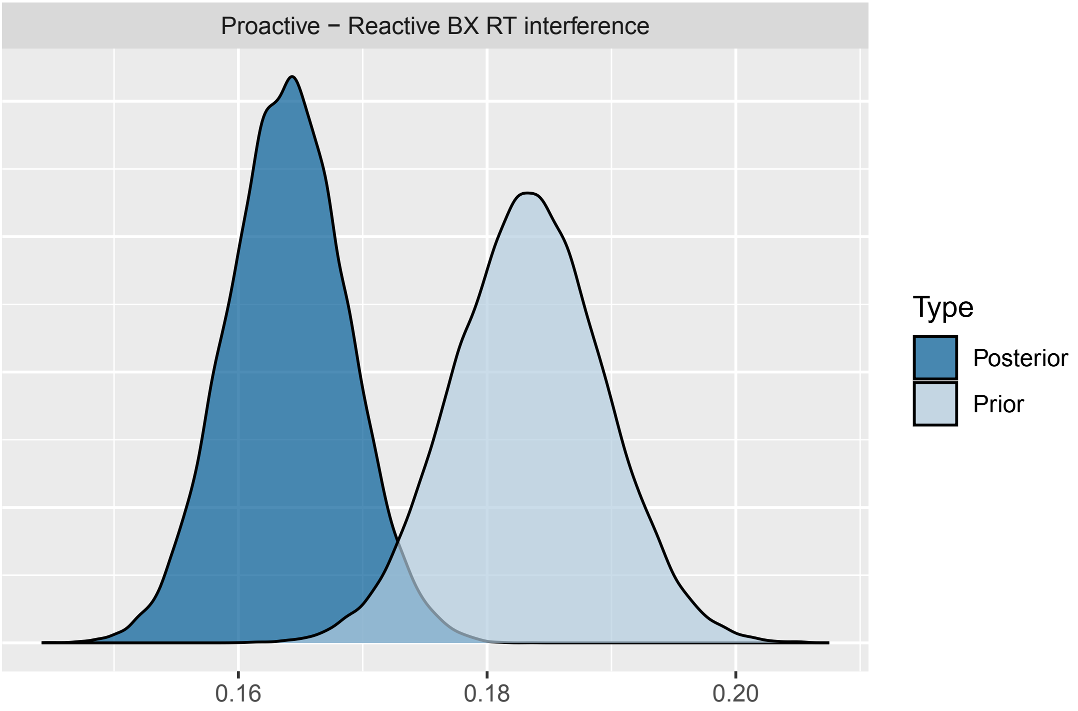

The plot indicates that the posterior is shifted away from the prior distribution. The graph is centered at the mean value of 2018 estimate, and indicates the SDR to be less than one (0) at that point. This suggests that the prior overestimates the difference between Reactive and Proactive BX RT interference. Nevertheless, the posterior distribution is also clearly shifted away from zero, indicating the reliability of the primary BX RT effect. More generally, the pattern across the 2018 and 2020 and datasets confirms the Proactive < Reactive BX RT interference pattern from Tang et al (2023).

2.7 AX-CPT Single Model Output

Based on the Reviewers' request, we also ran a single model of the 2018/2020 AX-CPT data that includes all analyzed predictor levels, and then subsequently extracted relevant contrasts via posterior linear combinations.

2.7.1 2018 A-Cue Bias Single Model Output

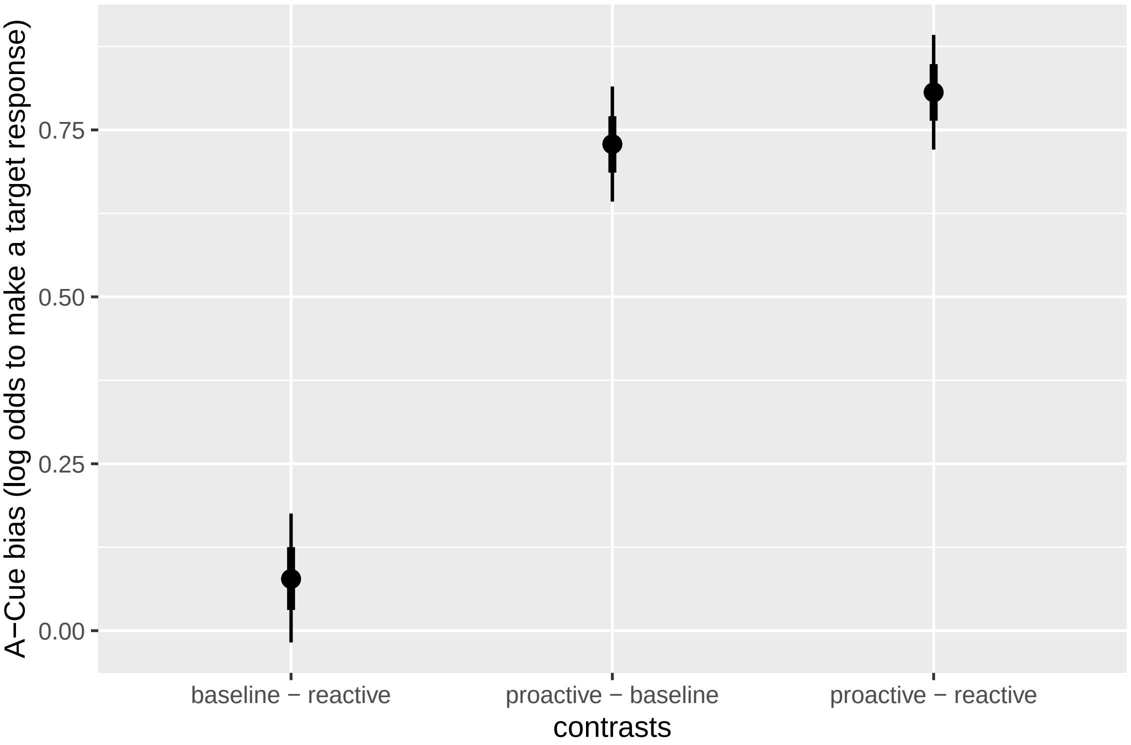

Table 1: A-Cue Bias Contrasts

Term	Estimate	HDI	pd
baseline - reactive	0.0775	[-0.02, 0.18]	94.34%
proactive - baseline	0.7286	[0.64, 0.81]	100%
proactive - reactive	0.8063	[0.72, 0.89]	100%

Note. Each of these terms reflect the main effects of mode. The key effects are 'proactive - baseline' and 'proactive - reactive', and they refer to the differences in log odds to make a target response for A-Cue trials between the Proactive mode and the other two conditions.

2.7.2 2020 A-Cue Bias Single Model Output

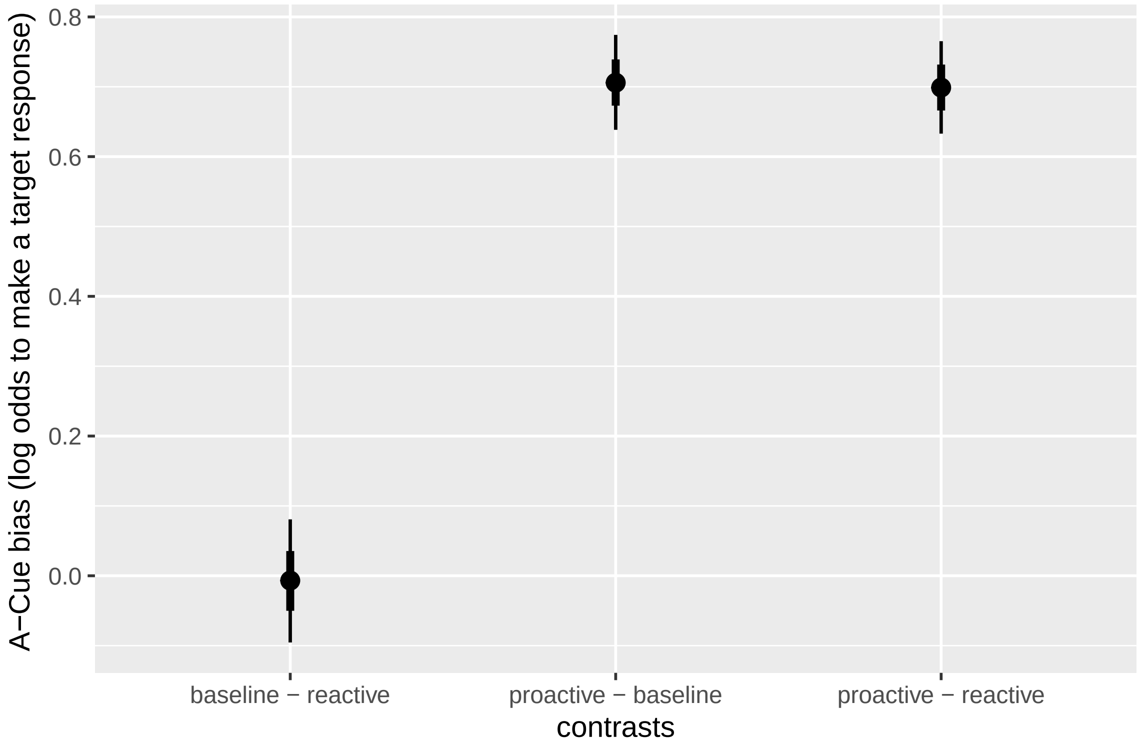

Table 2: A-Cue Bias Contrasts

Term	Estimate	HDI	pd
baseline - reactive	-0.0069	[-0.1, 0.08]	56.56%
proactive - baseline	0.7058	[0.64, 0.77]	100%
proactive - reactive	0.6989	[0.63, 0.77]	100%

Note. Each of these terms reflect the main effects of mode. The key effects are 'proactive - baseline' and 'proactive - reactive', and they refer to the differences in log odds to make a target response for A-Cue trials between the Proactive mode and the other two conditions.

2.7.3 2018 BX error interference Single Model Output

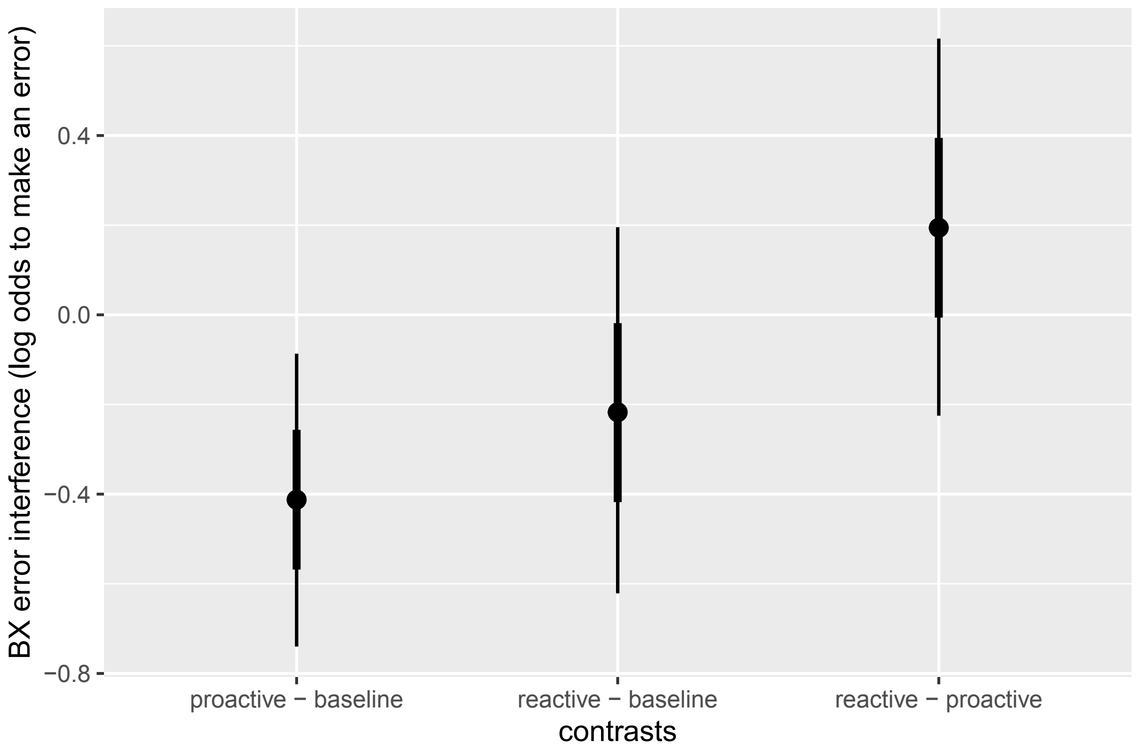

Table 3: BX error interference Contrasts

Term	Estimate	HDI	pd
proactive - baseline	-0.4124	[-0.73, -0.08]	99.3%
reactive - baseline	-0.2173	[-0.62, 0.2]	85.1%
reactive - proactive	0.1942	[-0.23, 0.61]	82.17%

Note. Each of these terms reflect the contrasting effects of mode. The key effects are 'proactive - baseline' and 'reactive - baseline', and they refer to the differences in log odds to make a correct response for BX-BY trials between the Proactive/Reactives modes versus Baseline.

2.7.4 2020 BX Error Interference Single Model Output

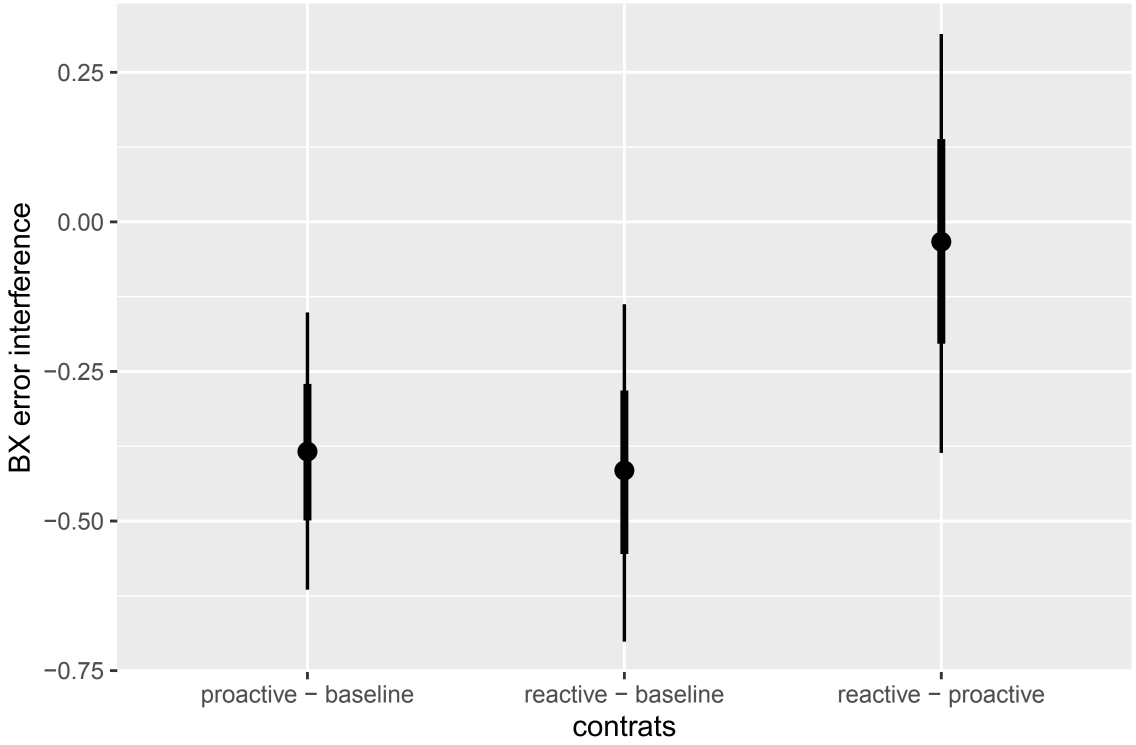

Table 4: BX error interference Contrasts

Term	Estimate	HDI	pd
proactive - baseline	-0.3838	[-0.61, -0.15]	99.85%
reactive - baseline	-0.4154	[-0.68, -0.12]	99.95%
reactive - proactive	-0.0332	[-0.37, 0.32]	57.55%

Note. Each of these terms reflect the contrasting effects of mode. The key effects are 'proactive - baseline' and 'reactive - baseline', and they refer to the differences in log odds to make a correct response for BX-BY trials between the Proactive/Reactives modes versus Baseline.

2.7.5 2018 BX RT interference Single Model Output

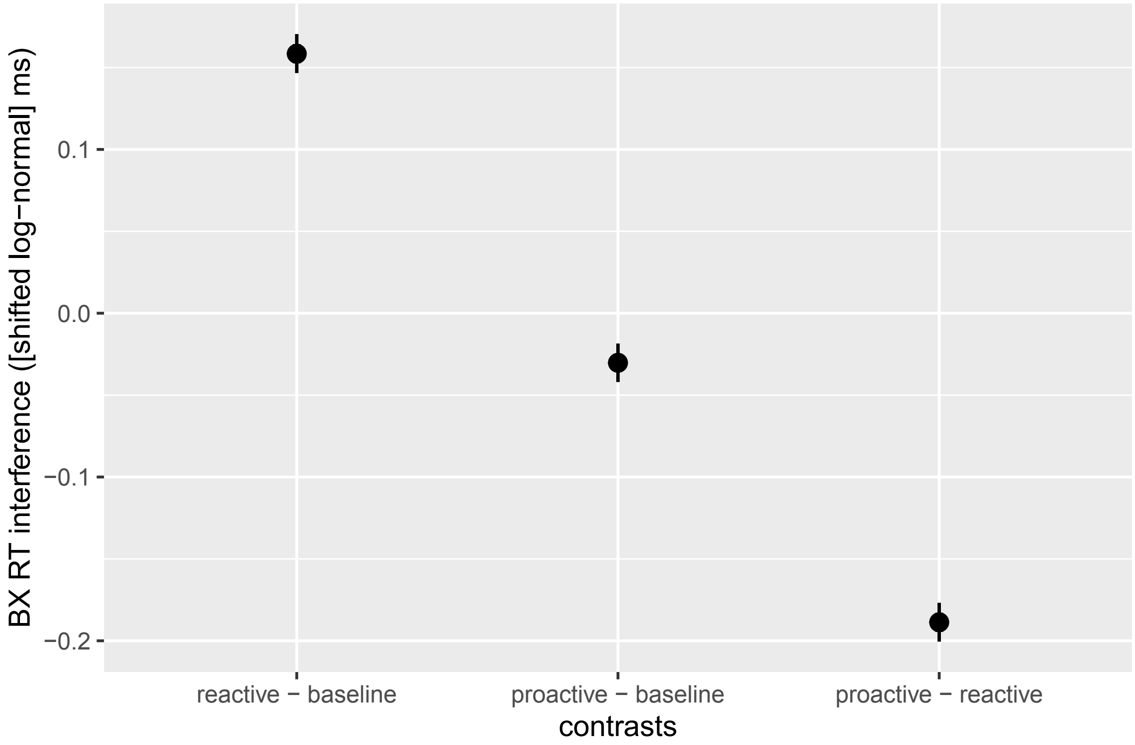

Table 5: BX RT interference Contrasts

Term	Estimate	HDI	pd
proactive - baseline	-0.0303	[-0.04, -0.02]	100%
proactive - reactive	-0.1887	[-0.2, -0.18]	100%
reactive - baseline	0.1584	[0.15, 0.17]	100%

Note. Each of these terms reflect the contrasting effects of mode. The key effects are 'proactive - baseline' and 'proactive - reactive', and they refer to the differences in the [shifted log-normal] RT to make a correct response for BX-BY trials between the Proactive mode versus the Baseline/Reactive conditions.

2.7.6 2020 BX RT Interference Single Model Output

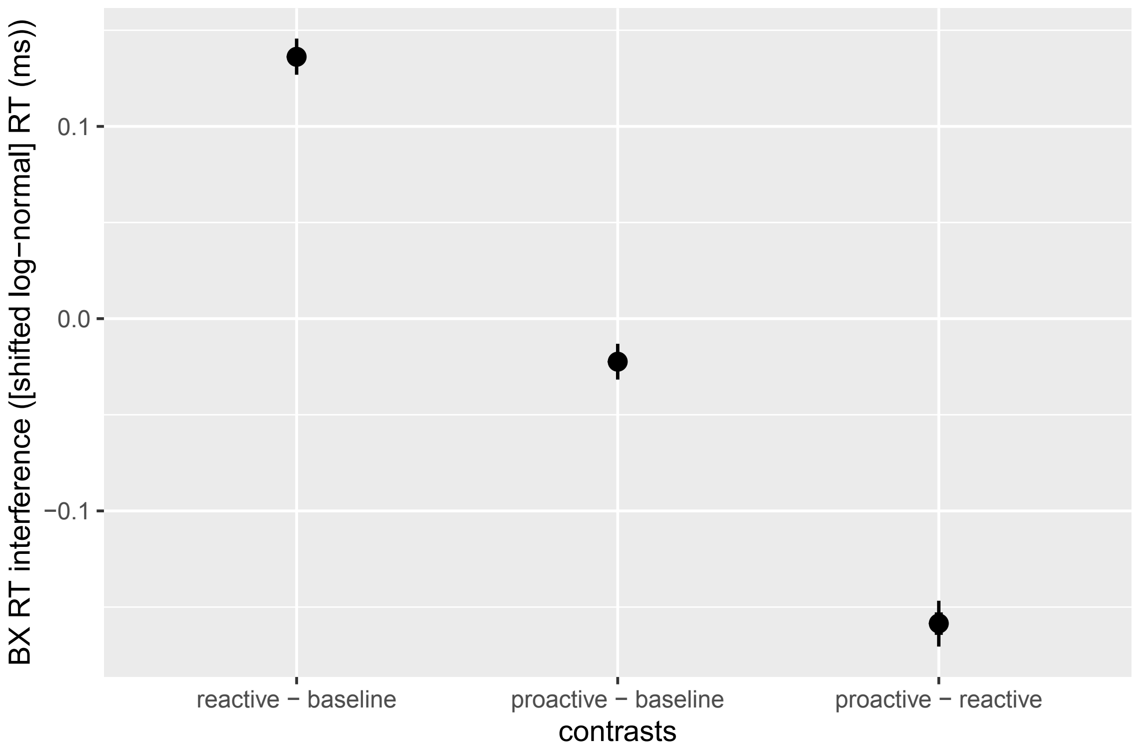

Table 6: BX RT interference Contrasts

Term	Estimate	HDI	pd
proactive - baseline	-0.0224	[-0.03, -0.01]	100%
proactive - reactive	-0.1585	[-0.17, -0.15]	100%
reactive - baseline	0.1362	[0.13, 0.15]	100%

Note. Each of these terms reflect the contrasting effects of mode. The key effects are 'proactive - baseline' and 'proactive - reactive', and they refer to the differences in the [shifted log-normal] RT to make a correct response for BX-BY trials between the Proactive mode versus the Baseline/Reactive conditions.

3 Sternberg

While the below sections will go into more detail for each Sternberg indicator, we briefly describe the expected results for differences in performance across conditions.

NP Error Effect: We predict subjects will 1) have fewer errors for NP critical trials in the Proactive mode versus Baseline mode, and 2) have fewer errors for NP critical trials in the Proactive mode versus Reactive mode.

NP RT Effect: We predict subjects will 1) be faster for NP critical trials in the Proactive mode versus Baseline mode, and 2) be faster for NP critical trials in the Proactive mode versus Reactive mode.

RN Recency RT Effect: We predict subjects will 1) have a reduced RN Recency Effect in the Reactive mode versus Baseline mode, and 2) have a reduced RN Recency Effect in the Reactive mode versus Proactive mode.

3.1 NP Error Effect 2018

The novel positive (NP) Error Effect can be modeled as the log odds to make a correct response for NP trials across modes. NP trials are defined as those that contain the probe item in the memory set of that trial. 'Mode' is dummy coded to make direct comparisons in NP Error performance between modes. Only critical trials were included in the models to make more direct comparisons between modes. Since the low number of items in a memory set should induce the proactive maintenance of the current word set in the critical trials, it was predicted that NP accuracy rates should be greater in the proactive mode versus the baseline and reactive modes.

Wilkinson Notation

$$\text{Correct} = \text{mode} + \left(1 \mid \text{ID}\right),\ \text{family}\left(\text{binomial}\right)$$

Fully Indexed Notation

$$\begin{gathered}
\text{Correct}_{i,t} \sim \text{Bernoulli}\left(p_{i,t}\right) \\
\text{logit}\left(p_{i,t}\right) = \beta_{0i} + \beta_1 * mode_t \\
\left[\beta_{0i}\right] \sim \text{N}\left(\left[\beta_0\right], \Sigma\right) \\
\beta_0, \beta_1 \sim \text{flat}, \Sigma \sim \text{LKJ}(1)
\end{gathered}$$

3.1.1 Proactive - Baseline NP Error Effect

Table 1: Proactive and Baseline NP Error Effect (null model)

Term	Estimate	SE	HDI	pd
NP	-2.1650	0.0858	[-2.33, -2]	100%

Note. The intercept term 'NP' refers to the average log odds to make an error for Baseline and Proactive NP trials.

Table 2: Proactive - Baseline NP Error Effect (full model)

Term	Estimate	SE	HDI	pd
Baseline NP	-2.1304	0.0906	[-2.31, -1.95]	100%
Proactive NP	-0.0685	0.0553	[-0.18, 0.04]	89.09%

Note. The intercept term 'Baseline NP' refers to the average log odds to make an error for Baseline NP trials. 'Proactive NP' is the key effect (NP effect) and refers to the difference in performance on NP trials across Baseline and Proactive mode.

There is little evidence for a difference in the NP Error Effect between the Proactive and Baseline modes (β = -0.07, se = 0.06, HDI = [-0.18, 0.04], pd = 89.09%).

3.1.2 Proactive - Reactive NP Error Effect

Table 3: Proactive and Reactive NP Error Effect (null model)

Term	Estimate	SE	HDI	pd
NP	-2.2872	0.0832	[-2.45, -2.13]	100%

Note. The intercept term 'NP' refers to the average log odds to make an error for the Reactive and Proactive NP trials.

Table 4: Proactive - Reactive NP Error Effect (full model)

Term	Estimate	SE	HDI	pd
Reactive NP	-2.4107	0.0882	[-2.58, -2.24]	100%
Proactive NP	0.2363	0.0579	[0.12, 0.35]	100%

Note. The intercept term 'Reactive NP' refers to the average log odds to make an error for Reactive NP trials. 'Reactive NP' is the key effect (NP effect) and refers to the difference in performance on NP trials across the Proactive and Reactive modes.

The NP Error Effect is greater in the Proactive mode relative to the Reactive mode (β = 0.24, se = 0.06, HDI = [0.12, 0.35], pd = 100%).

3.2 NP Error Effect 2020

These analyses test for a consistent pattern across the 2018 and 2020 datasets, using the 2018 estimates as prior information.

3.2.1 Proactive - Baseline NP Error Effect

Table 1: Proactive and Baseline NP Error Effect (null model)

Term	Estimate	SE	HDI	pd
NP	-2.2388	0.0634	[-2.36, -2.11]	100%

Note. The intercept term 'NP' refers to the average log odds to make an error for Baseline and Proactive NP trials.

Table 2: Proactive - Baseline NP Error Effect (full model)

Term	Estimate	SE	HDI	pd
Baseline NP	-2.1966	0.0666	[-2.33, -2.07]	100%
Proactive NP	-0.1297	0.0459	[-0.22, -0.04]	99.71%

Note. The intercept term 'Baseline NP' refers to the average log odds to make an error for Baseline NP trials. 'Proactive NP' is the key effect (NP Error Effect) and refers to the difference in performance on NP trials between the Baseline and Proactive modes.

There is strong evidence for a reduced NP Error Effect in the Proactive mode versus Baseline mode (β = -0.13, se = 0.05, HDI = [-0.22, -0.04], pd = 99.71%).

Table 3: Hypothesis Test for Pro-Bas NP Error Effect

Hypothesis	Estimate	SE	HDI	SDR	Post.Prob
Proactive - Baseline NP Error Effect	-0.06	0.05	[-0.22, -0.04]	0.50	0.00

Note. The key term in this table is the SDR score, which is computed as the ratio of the posterior distribution to the prior distribution at a specific point, in this case the mean of the prior distribution to investigate whether there is an increased likelihood of this value as a function of incoming data.

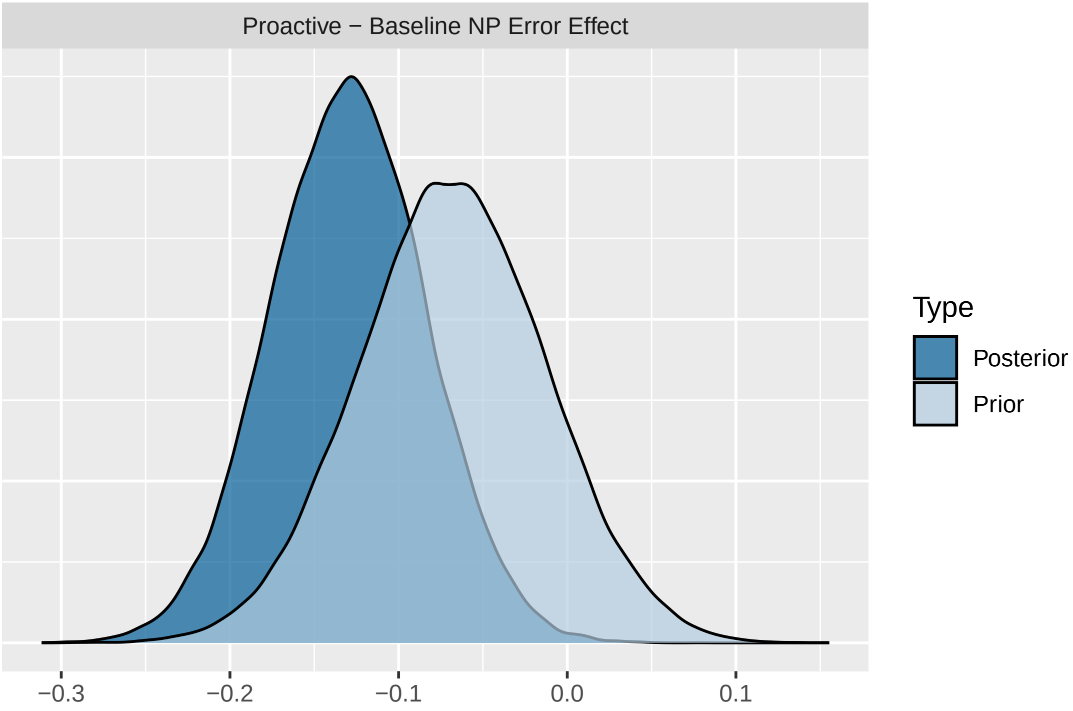

Shown that the prior and posterior of baseline versus proactive do not overlap. The graph is centered at the mean value of 2018 estimate, and finds the SDR to be less than one (0.5) at that point. This suggests that the prior underestimates the NP error effect between the Proactive and Baseline modes. This result provides stronger evidence for reduced NP errors in Proactive relative to Baseline, than was obtained in Tang et al (2023).

```
## Bayes Factors for Model Comparison
##
##     Model                              BF
## [1] 0 + Intercept + mode + (1 | ID) 15.33
##
## * Against Denominator: [2] 0 + Intercept + (1 | ID)
## *   Bayes Factor Type: marginal likelihoods (bridgesampling)
```

The BF_{10} is greater than 10, indicating stronger evidence for reduced NP errors in the Proactive versus Baseline mode than what was obtained in Tang et al. (2023).

```
##                         elpd_diff se_diff
## Sternberg_baspro         0.0       0.0
## Sternberg_baspro_null -3.3       1.6
```

For LOO-CV, the elpd difference between M_1 (full model) and M_0 (null model) was less than four. This indicates that the inclusion of the interaction term for the Proactive-Baseline model did not meaningfully improve predictive accuracy.

3.2.2 Proactive - Reactive NP Error Effect

Table 4: Reactive and Proactive NP Error Effect (null model)

Term	Estimate	SE	HDI	pd
NP	-2.3430	0.0623	[-2.47, -2.22]	100%

Note. The intercept term 'NP' refers to the average log odds to make an error for Reactive and Proactive NP trials.

Table 5: Proactive - Reactive NP Error Effect

Term	Estimate	SE	HDI	pd
Reactive NP	-2.4457	0.0666	[-2.58, -2.32]	100%
Proactive NP	0.1323	0.0474	[0.04, 0.23]	99.69%

Note. The intercept term 'Reactive NP' refers to the average log odds to make an error for Proactive NP trials. 'Proactive NP' is the key effect (NP Effect) and refers to the difference in performance on NP trials between the Proactive and Reactive mode.

There was strong evidence for an increased NP Error Effect in the Proactive mode versus Reactive mode (β = 0.13, se = 0.05, HDI = [0.04, 0.23], pd = 99.69%).

Table 6: Hypothesis Test for Pro-Rea NP Error Effect

Hypothesis	Estimate	SE	HDI	SDR	Post.Prob
Proactive - Reactive NP Error Effect	-0.10	0.05	[-0.22, -0.04]	0.11	0.00

Note. The key term in this table is the SDR score, which is computed as the ratio of the posterior distribution to the prior distribution at a specific point, in this case the mean of the prior distribution to investigate whether there is an increased likelihood of this value as a function of incoming data.

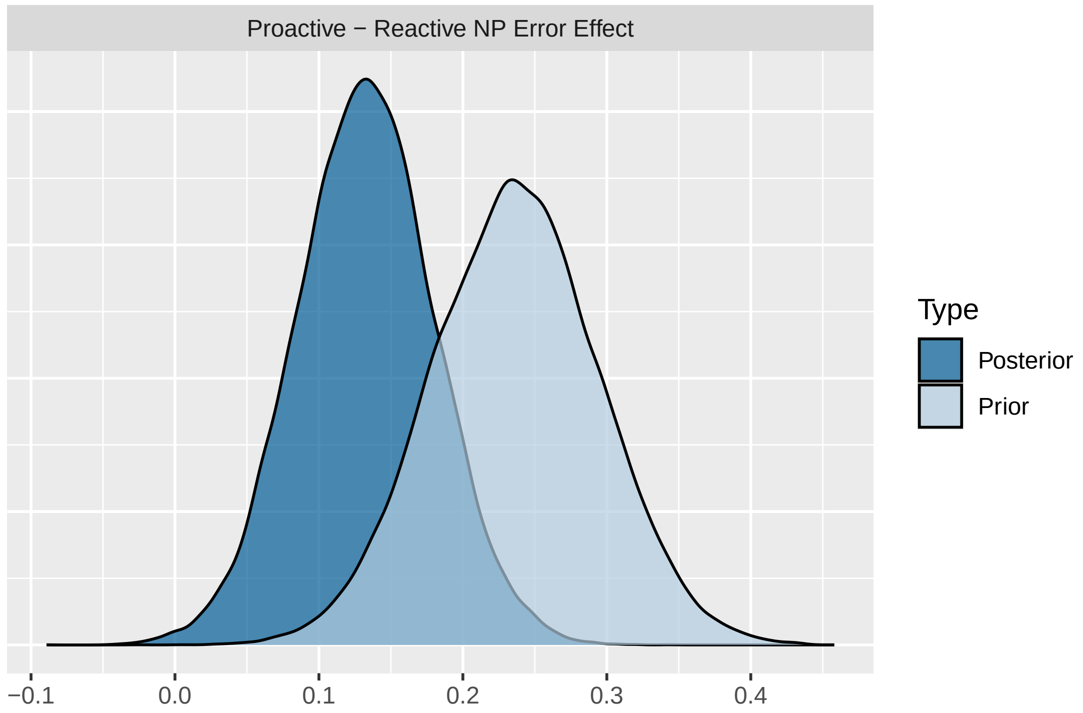

Shown that the prior and posterior of Reactive vs Proactive do not overlap. The graph is centered at the mean value of 2018 estimate, and finds the SDR to be less than one (0.11) at that point. This suggests that the prior overestimates the NP Error Effect between the Proactive and Reactive mode. This pattern is in the same direction as observed in Tang et al (2023) but provides stronger evidence against the predicted hypothesis (of reduced NP errors in Proactive).

```
## Bayes Factors for Model Comparison
##
##     Model                                 BF
## [1] 0 + Intercept + mode + (1 | ID) 0.016
##
## * Against Denominator: [2] 0 + Intercept + (1 | ID)
## *   Bayes Factor Type: marginal likelihoods (bridgesampling)
```

The BF_{10} was less than 1/10, indicating that the null model M_0 was strongly favored over the full model M_1. This finding provides stronger evidence for a Proactive effect than obtained in Tang et al (2023).

```
##                        elpd_diff se_diff
## Sternberg_reapro_null  0.0       0.0
## Sternberg_reapro      -2.8       1.6
```

For LOO-CV, the elpd difference between M_1 (full model) and M_0 (null model) was less than both four and twice its standard deviation. This indicates that the inclusion of the interaction term for the Proactive-Reactive model did not meaningfully improve predictive accuracy.

3.3 NP RT Effect 2018

The NP effect for RT is defined as the RT for correct responses for NP trials across modes. NP trials are defined as those that contain the probe item in the memory set of that trial. 'Mode' is dummy coded to make direct comparisons in NP performance between modes. Only critical trials (i.e., 5-item memory sets) were included in the models to make more direct comparisons between modes. Since the low number of items in a memory set should induce the proactive maintenance of the current word set in the critical trials, it was predicted that NP response times should be greater in the Proactive mode versus the Baseline and Reactive modes.

Wilkinson Notation

$$RT = mode\ + (1 \mid ID)\text{, family}\left(\text{ex-Gaussian}\right)$$

Fully Indexed Notation

$$\begin{gathered}
\text{RT}_{i,t} \sim \text{ex-Gaussian}\left(\mu_t, \sigma, \tau\right) \\
\left(\mu_{i,t}\right) = \beta_{0i} + \beta_1 * mode_t \\
\left[\beta_{0i}\right] \sim \text{N}\left(\left[\beta_0\right], \Sigma\right) \\
\beta_0, \beta_1 \sim \text{flat}, \Sigma \sim \text{LKJ(1)}, \sigma \sim \text{student-t(3, 0, 201.6)}, \tau \sim \text{gamma(1, .1)}
\end{gathered}$$

3.3.1 Proactive - Baseline NP RT Effect

Table 1: Proactive and Baseline NP RT Effect (null model)

Term	Estimate	SE	HDI	pd
NP	848.6506	8.4738	[832.45, 865.71]	100%

Note. The intercept term 'NP' refers to the average RT to make a correct response for baseline and proactive NP trials.

Table 2: Proactive - Baseline NP RT Effect (full model)

Term	Estimate	SE	HDI	pd
Baseline NP	855.9403	8.3468	[839.4, 872.55]	100%
Proactive NP	-16.6484	2.8341	[-22.12, -10.96]	100%

Note. The intercept term 'Baseline NP' refers to the average RT to make a correct response for Baseline NP trials. 'Proactive NP' is the key effect (NP RT Effect) and refers to the difference in performance on NP trials across Baseline and Proactive mode.

There is decisive evidence that the NP RT Effect is faster in the Proactive mode relative to the Baseline mode (β = -16.65, se = 2.83, HDI = [-22.12, -10.96], pd = 100%).

3.3.2 Proactive - Reactive NP RT Effect

Table 3: Proactive and Reactive NP RT Effect (null model)

Term	Estimate	SE	HDI	pd
NP	843.2555	8.3384	[826.5, 859.12]	100%

Note. The intercept term 'NP' refers to the average RT to make a correct response for the Reactive and Proactive NP trials.

Table 4: Proactive - Reactive NP RT Effect (full model)

Term	Estimate	SE	HDI	pd
Reactive NP	847.7299	8.7591	[829.99, 864.94]	100%
Proactive NP	-7.1860	2.7662	[-12.76, -1.95]	99.54%

Note. The intercept term 'Reactive NP' refers to the average RT to make a correct response for Reactive NP trials. 'Reactive NP' is the key effect (NP RT Effect) and refers to the difference in performance on NP trials across the Proactive and Reactive modes.

There is strong evidence that the NP RT Effect is greater in the Proactive mode relative to the Reactive mode (β = -7.19, se = 2.77, HDI = [-12.76, -1.95], pd = 99.54%).

3.4 NP RT Effect 2020

These analyses test for a consistent pattern across the 2018 and 2020 datasets, using the 2018 estimates as prior information.

3.4.1 Proactive - Baseline NP RT Effect

Table 1: Proactive and Baseline NP RT Effect (null model)

Term	Estimate	SE	HDI	pd
NP	859.7193	7.1129	[845.75, 873.7]	100%

Note. The intercept term 'NP' refers to the average RT to make a correct response for Baseline and Proactive NP trials.

Table 2: Proactive - Baseline NP RT Effect (full model)

Term	Estimate	SE	HDI	pd
Baseline NP	867.7765	7.0780	[854.03, 881.58]	100%
Proactive NP	-19.9220	2.3771	[-24.57, -15.26]	100%

Note. The intercept term 'Baseline NP' refers to the average RT to make a correct response for Baseline NP trials. 'Proactive NP' is the key effect (NP RT Effect) and refers to the difference in performance on NP trials between the Baseline and Proactive mode.

There is decisive evidence for a strong NP RT Effect in the Proactive mode relative to the Baseline mode (β = -19.92, se = 2.38, HDI = [-24.57, -15.26], pd = 100%).

Table 3: Hypothesis Test for Pro-Bas NP RT Effect

Hypothesis	Estimate	SE	HDI	SDR	Post.Prob
Proactive - Baseline NP RT Effect	-3.27	2.38	[-24.57, -15.26]	0.46	0.00

Note. The key term in this table is the SDR score, which is computed as the ratio of the posterior distribution to the prior distribution at a specific point, in this case the mean of the prior distribution to investigate whether there is an increased likelihood of this value as a function of incoming data.

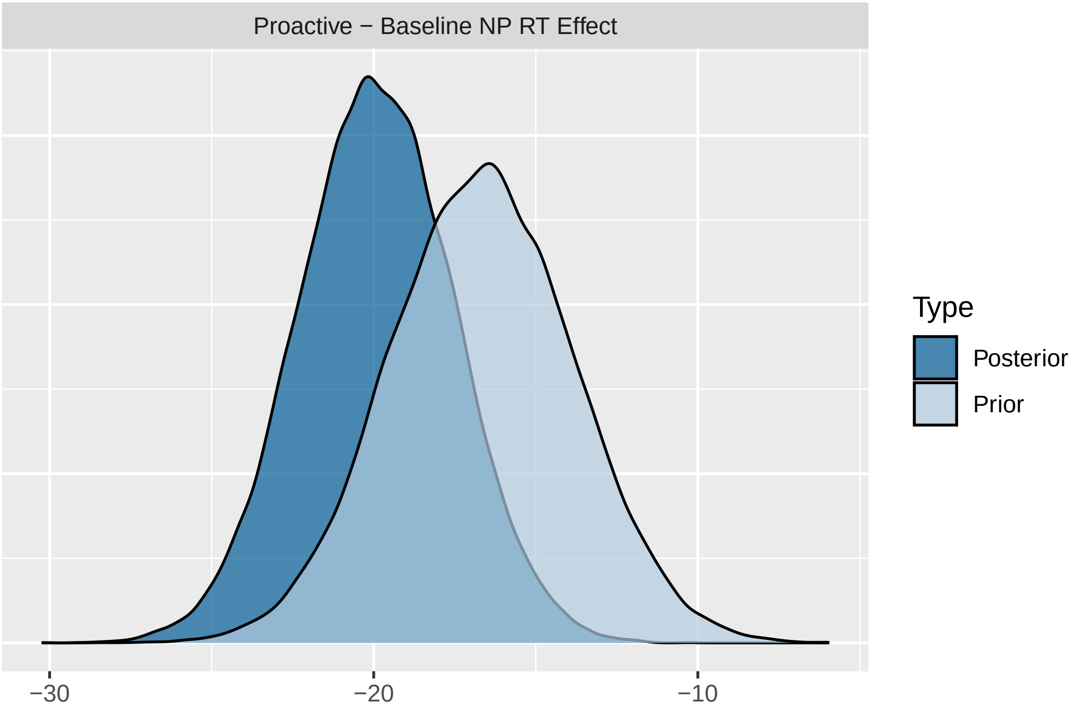

The plot indicates that the posterior is shifted away from the prior distribution. The graph is centered at the mean value of 2018 estimate, and indicates the SDR to be less than one (0.46) at that point. This suggests that the prior underestimates the NP RT effect between the Proactive and Reactive modes. This pattern replicates that observed in Tang et al (2023) but with stronger evidence.

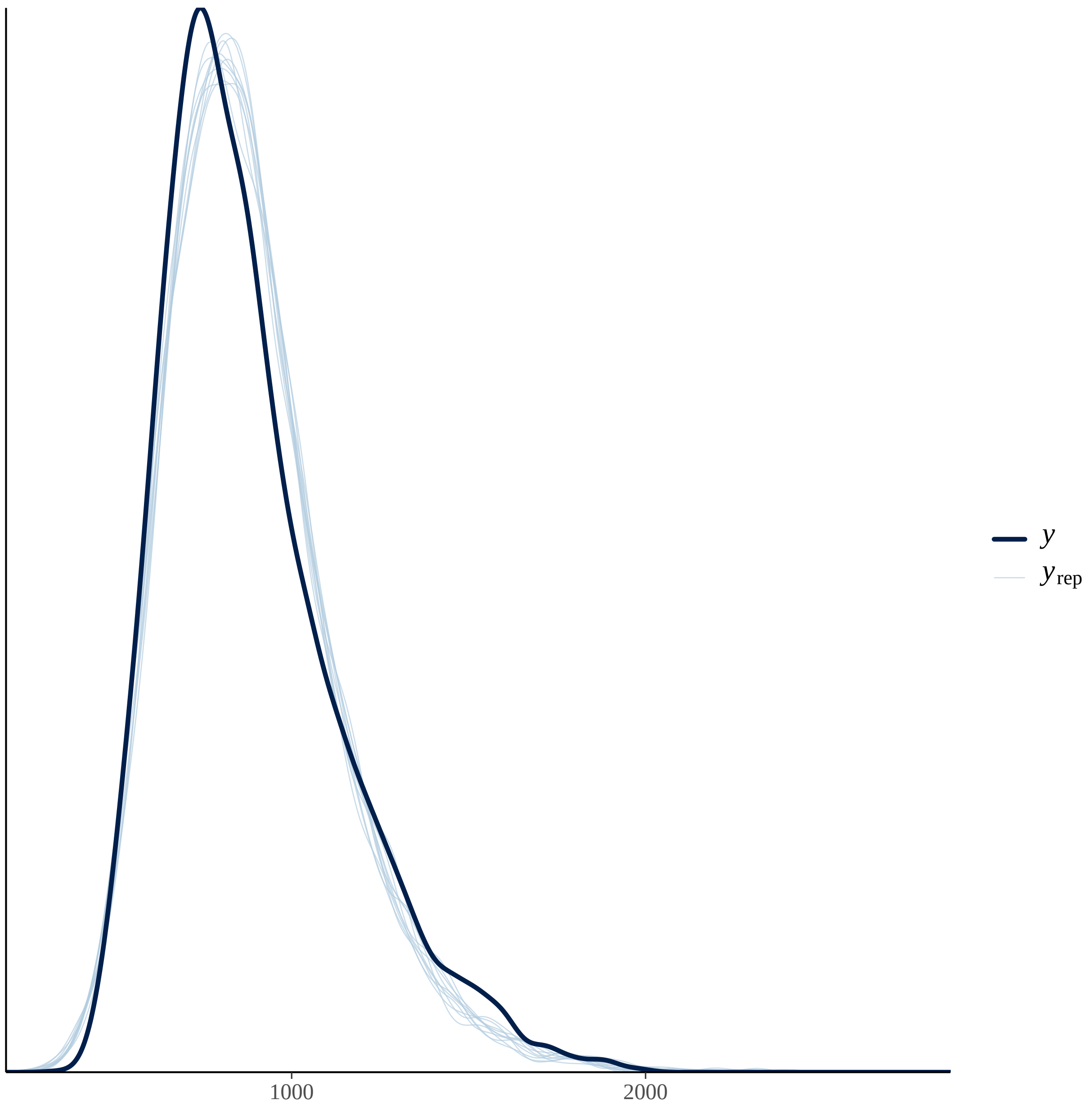

1000
2000
y
y_{rep}

```
## Bayes Factors for Model Comparison
##
##     Model                                BF
## [1] 0 + Intercept + mode + (1 | ID) 1.72e+08
##
## * Against Denominator: [2] 0 + Intercept + (1 | ID)
## *   Bayes Factor Type: marginal likelihoods (bridgesampling)
```

The BF_{10} was much greater than 10, indicating that the full model M_1 was strongly favored over the null model M_0. This finding provides stronger evidence for a Proactive effect than that obtained in Tang et al (2023).

```
##                        elpd_diff se_diff
## Sternberg_baspro         0.0       0.0
## Sternberg_baspro_null -20.4       4.8
```

For LOO-CV, the elpd difference between M_1 (full model) and M_0 (null model) was greater than both four and twice its standard deviation. This indicates that the inclusion of the interaction term for the Proactive versus Baseline model meaningfully improved predictive accuracy.

```
## # Proportion of samples inside the ROPE [-5.00, 5.00]:
##
## inside ROPE
## -----------
## 0.00 %
```

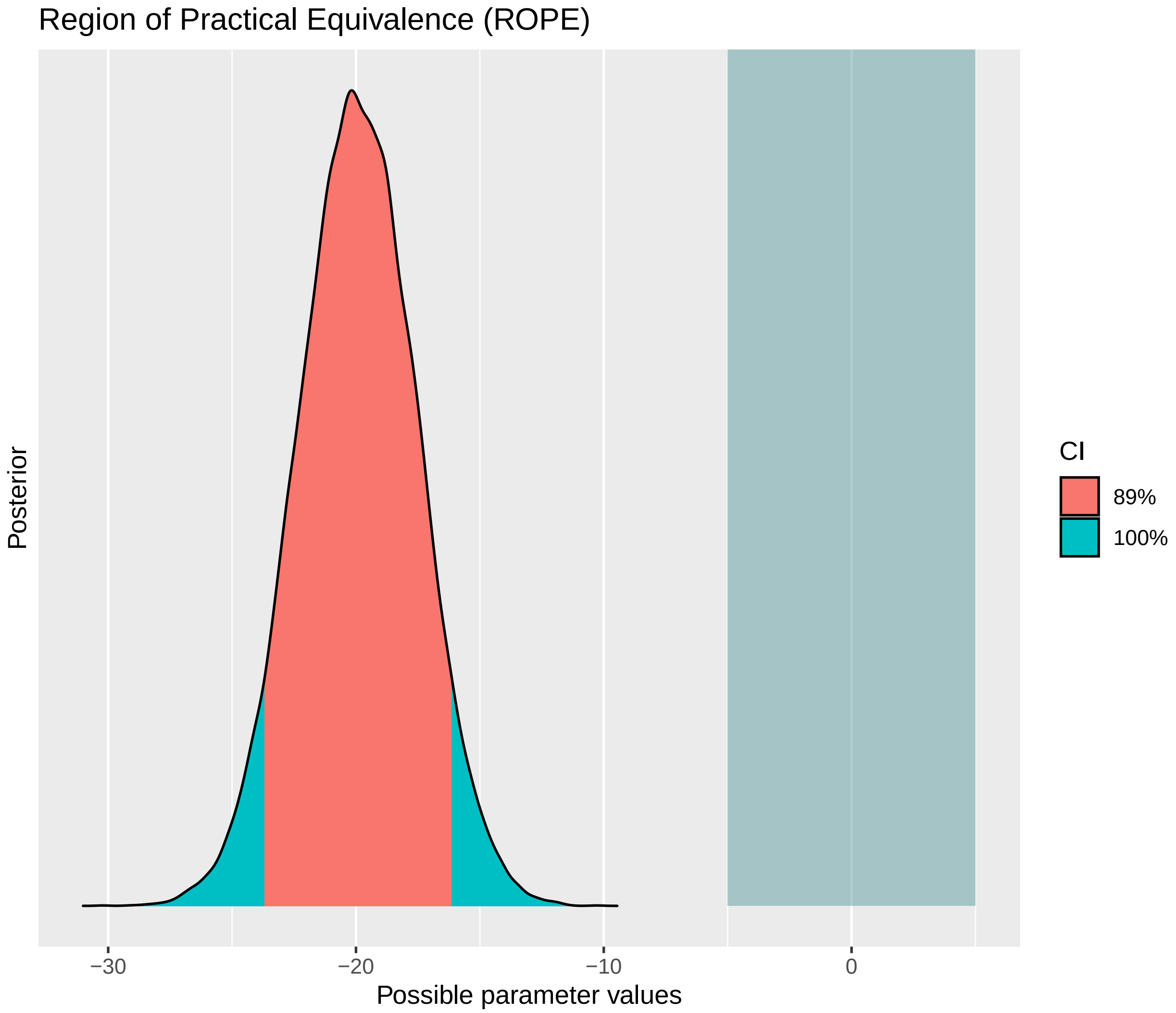

While assuming the default 89% HDI, there is strong evidence for the alternative effect regarding the difference in NP RT Effect between the Proactive and Baseline modes.

```
## # Proportion of samples inside the ROPE [-5.00, 5.00]:
##
## inside ROPE
## -----------
## 0.00 %
```

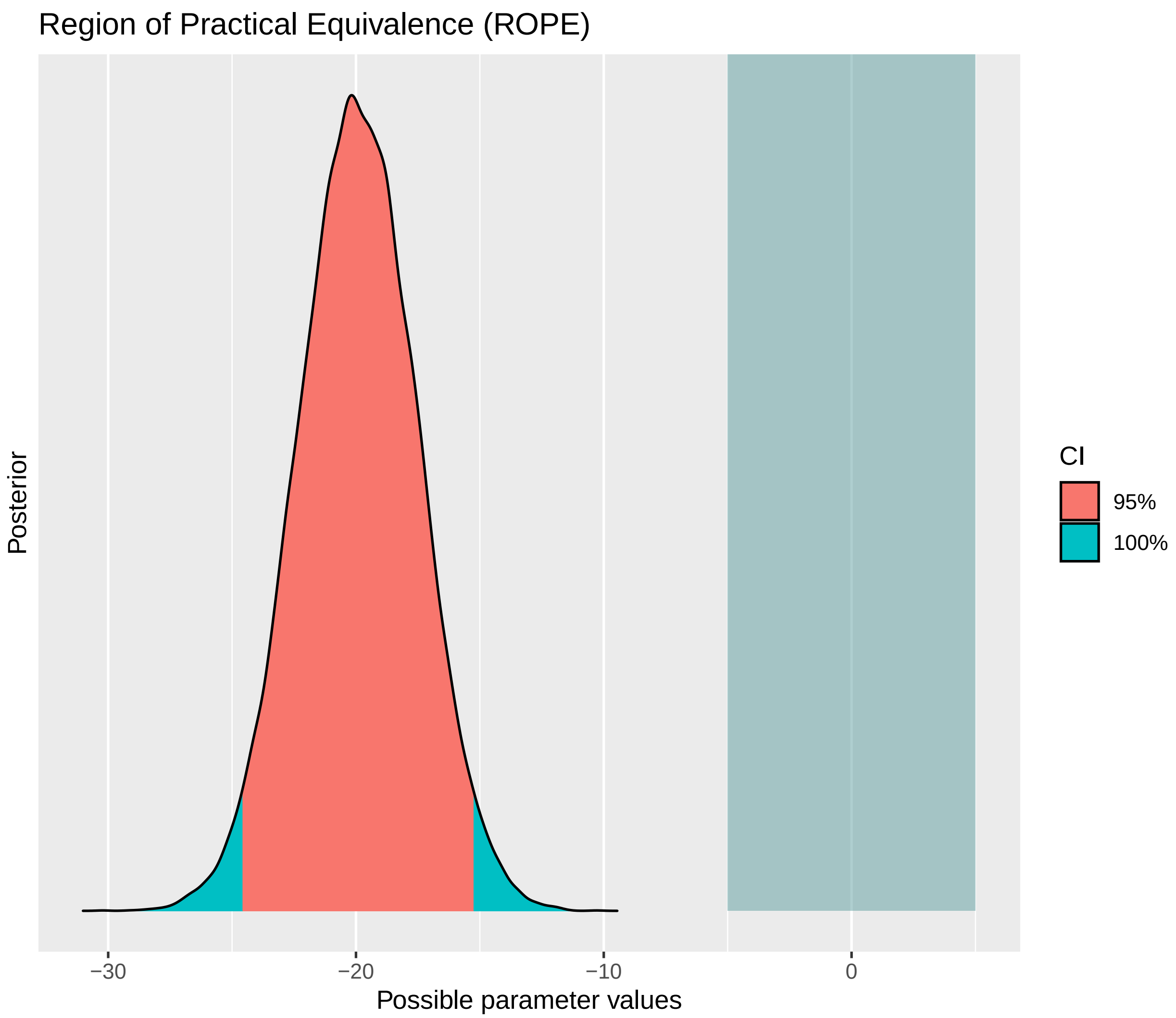

Even while assuming the more conservative 95% HDI, there is strong evidence for the alternative effect regarding the difference in NP RT Effect for the Proactive mode versus Baseline mode.

3.4.2 Proactive - Reactive NP RT Effect

Table 4: Proactive and Reactive NP RT Effect (null model)

Term	Estimate	SE	HDI	pd
NP	847.9957	6.8910	[834.76, 861.76]	100%

Note. The intercept term 'NP' refers to the average RT to make a correct response for Reactive and Proactive NP trials.

Table 5: Proactive - Reactive NP RT effect

Term	Estimate	SE	HDI	pd
Reactive NP	851.1919	7.1822	[837.04, 865.29]	100%
Proactive NP	0.7263	2.2471	[-3.63, 5.19]	62.67%

Note. The intercept term 'Reactive NP' refers to the average RT to make a correct response for Proactive NP trials. 'Proactive NP' is the key effect (NP effect) and refers to the difference in performance on NP trials between the Reactive and Proactive modes.

There was little evidence for a difference in the NP RT Effect between the Proactive and Reactive modes (β = 0.73, se = 2.25, HDI = [-3.63, 5.19], pd = 62.67%).

Table 6: Hypothesis Test for Pro-Rea NP RT Effect

Hypothesis	Estimate	SE	HDI	SDR	Post.Prob
Proactive - Reactive NP RT Effect	7.91	2.25	[-3.63, 5.19]	0.00	0.00

Note. The key term in this table is the SDR score, which is computed as the ratio of the posterior distribution to the prior distribution at a specific point, in this case the mean of the prior distribution to investigate whether there is an increased likelihood of this value as a function of incoming data.

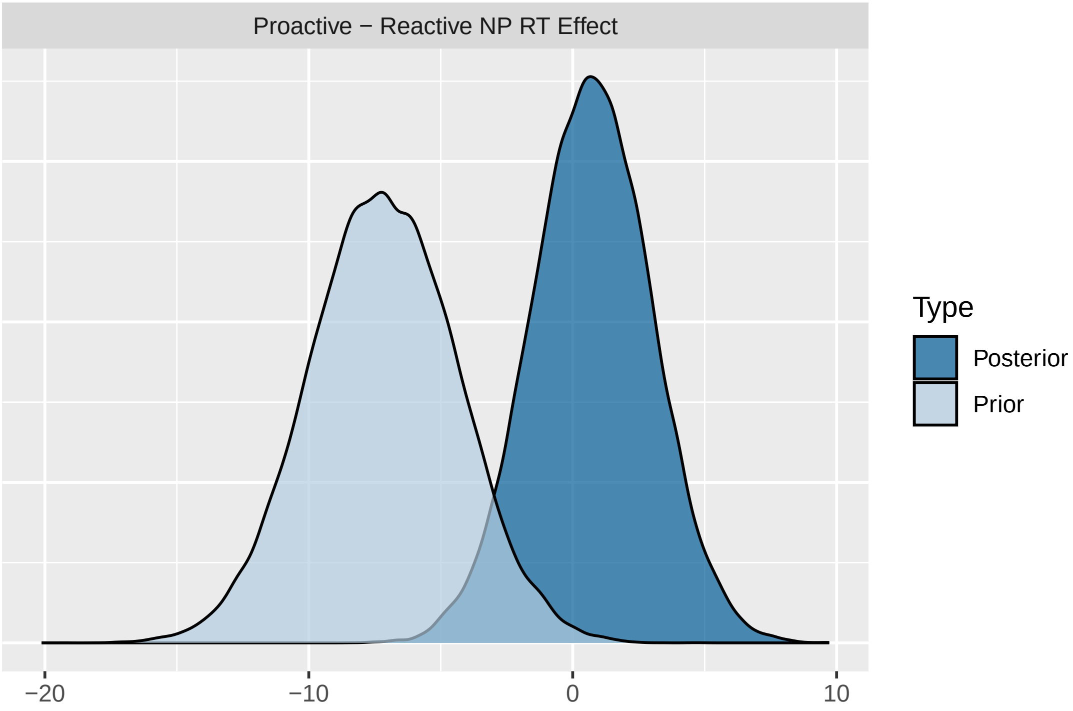

The plot indicates that the posterior is shifted away from the prior distribution. The graph is centered at the mean value of 2018 estimate, and indicates the SDR to be less than one (0) at that point. This suggests that the prior overestimates the NP RT effect between the Proactive and Reactive modes. This pattern strengthens the conclusion of a null effect reported in Tang et al (2023).

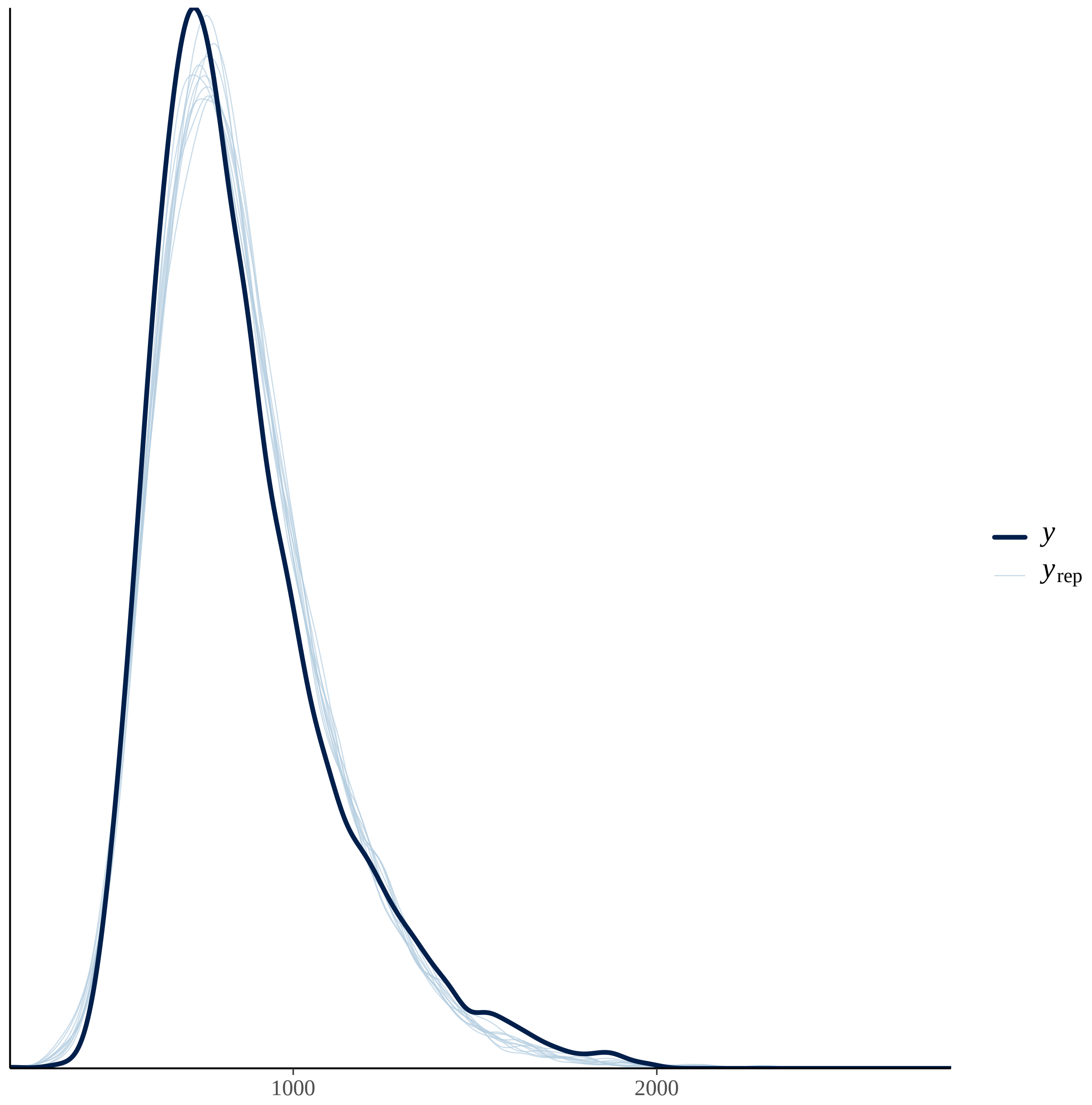

1000
2000
y
y rep

```
## Bayes Factors for Model Comparison
##
##     Model                                BF
## [1] 0 + Intercept + mode + (1 | ID) 0.038
##
## * Against Denominator: [2] 0 + Intercept + (1 | ID)
## *   Bayes Factor Type: marginal likelihoods (bridgesampling)
```

The BF_{10} was much less than 1/10, indicating strong evidence favoring the null model M_0 over the full model M_1. This pattern strengthens the conclusions reported in Tang et al (2023).

```
##                        elpd_diff se_diff
## Sternberg_reapro        0.0       0.0
## Sternberg_reapro_null -0.3       0.2
```

For LOO-CV, the elpd difference between M_1 (full model) and M_0 (null model) was less than both four and twice its standard deviation. This indicates that the inclusion of the interaction term for the Proactive versus Reactive model did not meaningfully improve predictive accuracy.

```
## # Proportion of samples inside the ROPE [-5.00, 5.00]:
##
## inside ROPE
## -----------
## 100.00 %
```

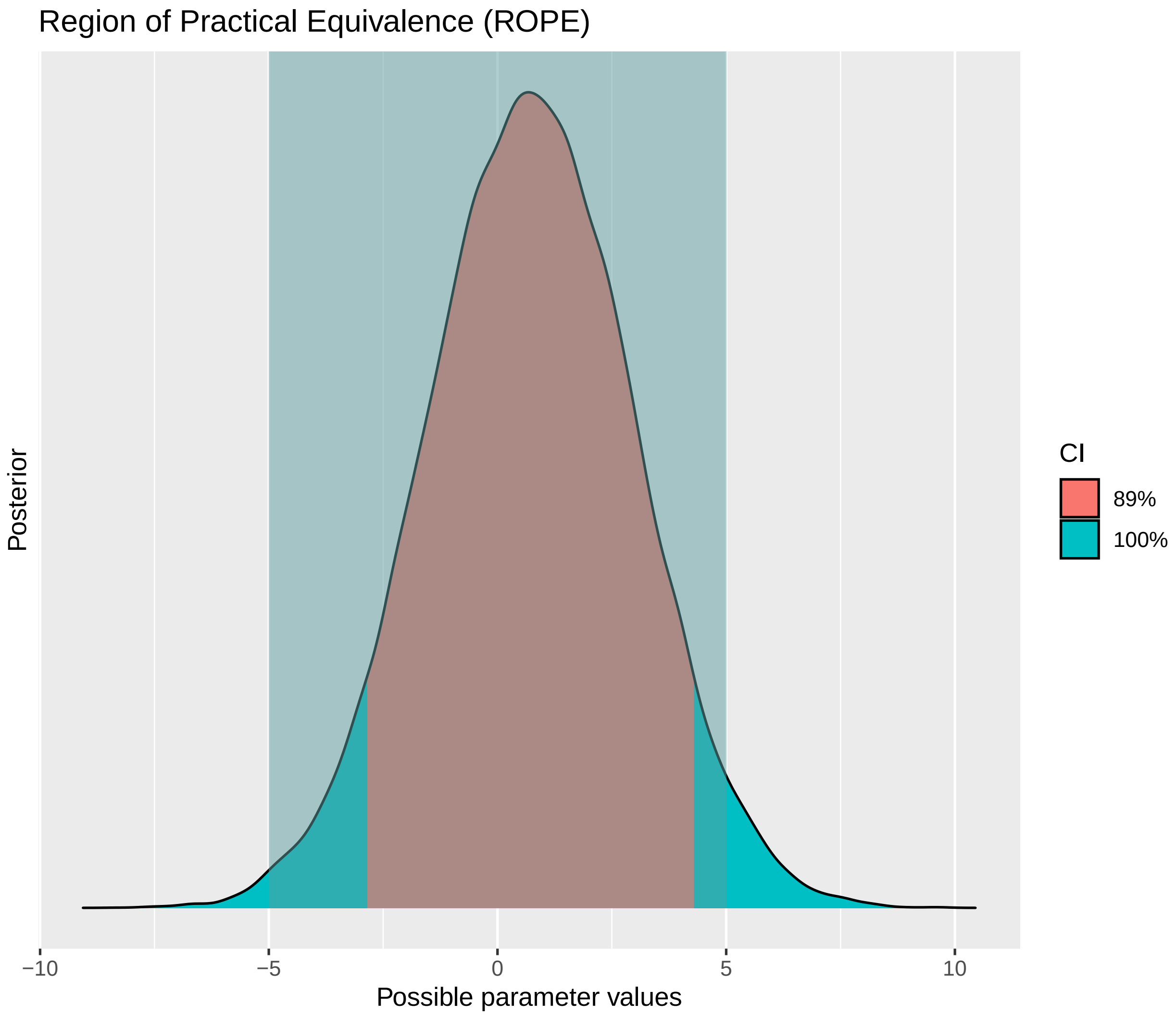

While assuming the default 89% HDI, there is strong evidence for the null effect regarding the difference in NP RT Effect for the Proactive mode versus Reactive mode.

```
## # Proportion of samples inside the ROPE [-5.00, 5.00]:
##
## inside ROPE
## -----------
## 99.57 %
```

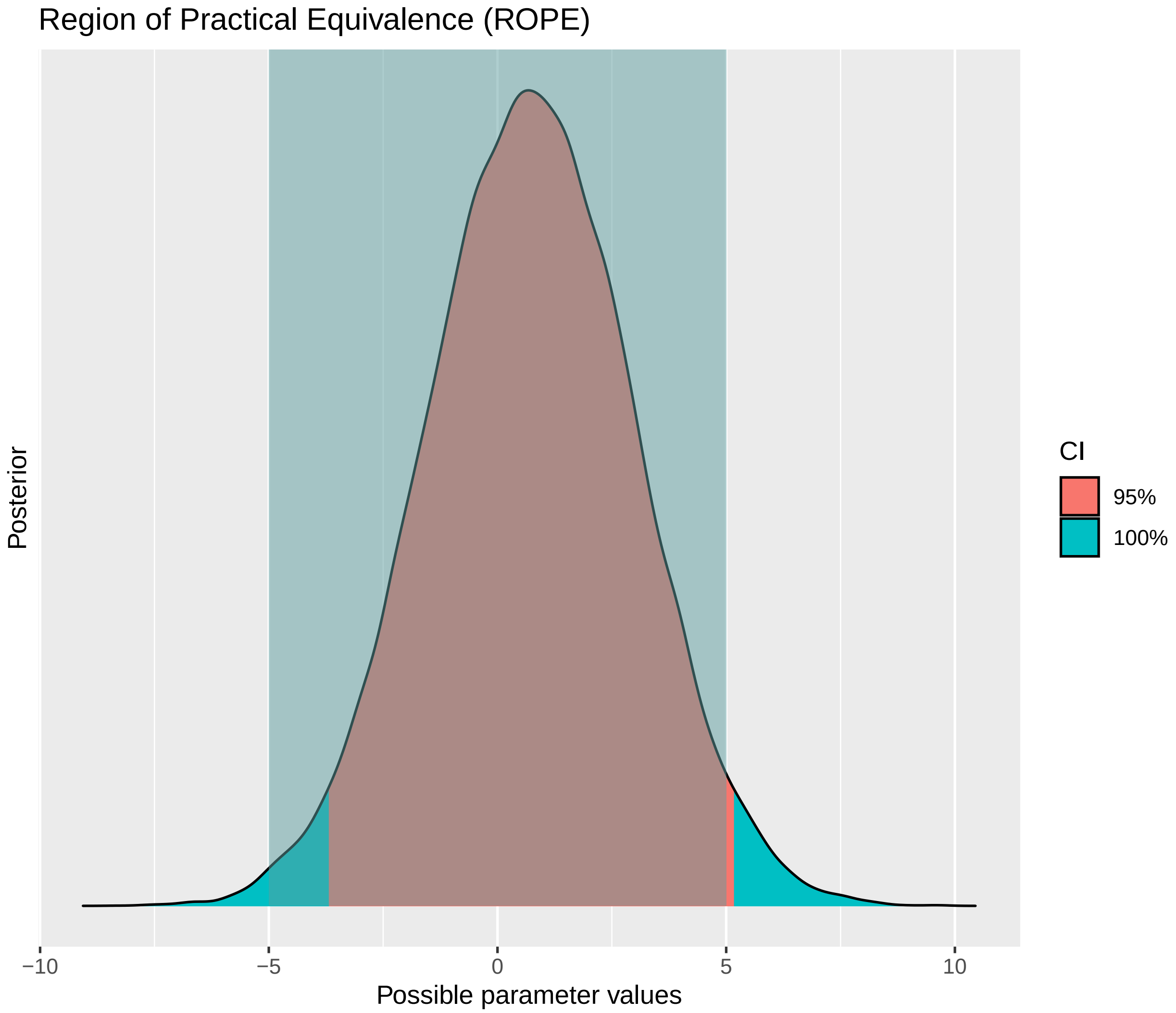

While assuming a more conservative 95% HDI, there is inconclusive evidence for the null effect regarding the difference in NP RT Effect for the Proactive versus Reactive modes.

3.5 RN Recency Effect RT 2018

The RN Recency Effect for RT is defined as the difference in the [shifted log-normal] RTs for correct responses between recent negative (RN) and novel negative (NN) trials across modes. RN trials are defined as those with memory sets that do not contain the probe item in the current trial but had the probe item in that of a recent prior trial. NN trials are those in which the probe item did not appear in the current trial or any recent prior trial. The analyses are conducted on the critical items (5-item memory set trials) which were matched across modes. Both 'mode' and 'Trial Type' were dummy coded and treated as interacting variables to allow for direct comparisons in RN recency effect between modes. The effects of RN interference are predicted to be reduced in the Reactive mode, relative to Baseline and Proactive.

Wilkinson Notation

$$RT = mode\ x\ Trial\ Type\ + \left(1 + Trial\ Type \mid ID\right)$$

Fully Indexed Notation

$$\begin{gathered}
\mathrm{RT}_{i,t} \sim \text{shifted log-normal}\left(\mu_{i,t}, \sigma, \theta\right) \\
\log\left(\mu_{i,t} + \theta\right) = \beta_{0i} + \beta_1 * mode_t\ x\ \beta_{2i} * trialType_{i,t} \\
\begin{bmatrix} \beta_{0i} \\ \beta_{2i} \end{bmatrix} \sim \mathrm{N}\left(\begin{bmatrix} \beta_0 \\ \beta_2 \end{bmatrix}, \Sigma\right) \\
\beta_0, \beta_1, \beta_2 \sim \text{flat}, \Sigma \sim \text{LKJ}(1), \sigma \sim \text{student-t(3, 0, 2.5)}, \theta \sim \text{uniform(0, minRT)}
\end{gathered}$$

3.5.1 Reactive - Baseline RN Recency Effect

Table 1: Reactive - Baseline RN Recency effect

Term	Estimate	SE	HDI	pd
Baseline NN	6.0634	0.0265	[6.01, 6.12]	100%
Baseline RN	0.1964	0.0126	[0.17, 0.22]	100%
Reactive NN	0.0342	0.0097	[0.02, 0.05]	99.96%
Reactive RN	-0.0319	0.0142	[-0.06, 0]	98.69%

Note. The intercept term 'Baseline NN' refers to the average shifted lognormal RT to make a correct response for baseline NN trials. 'Reactive RN' is the key effect (recency effect) and refers to the difference in performance of RN and NN trials across the Baseline and Reactive mode.

There was strong evidence for a reduced RN Recency Effect in the Reactive mode versus Baseline mode (β = -0.03, se = 0.01, HDI = , pd = 98.69%).

3.5.2 Reactive - Proactive RN Recency Effect

Table 2: Reactive - Proactive RN Recency effect

Term	Estimate	SE	HDI	pd
Proactive NN	6.1686	0.0246	[6.12, 6.21]	100%
Proactive RN	0.2465	0.0112	[0.23, 0.27]	100%
Reactive NN	0.0366	0.0089	[0.02, 0.05]	100%
Reactive x RN	-0.0974	0.0134	[-0.12, -0.07]	100%

Note. The intercept term 'Proactive NN' refers to the average average shifted lognormal RT to make a correct response for Proactive NN trials. 'Reactive x RN' is the key effect (recency effect) and refers to the difference in performance of RN and NN trials between the Reactive and Proactive mode.

There was decisive evidence for a reduced RN Recency Effect in the Reactive mode relative to Proactive mode (β = -0.1, se = 0.01, HDI = [-0.12, -0.07], pd = 100%).

3.6 RN Recency Effect RT 2020

These analyses test for a consistent pattern across the 2018 and 2020 datasets, using the 2018 prior estimates as prior information.

3.6.1 Reactive - Baseline RN Recency Effect

Table 1: Reactive - Baseline RN Recency Effect

Term	Estimate	SE	HDI	pd
Baseline NN	6.2271	0.0215	[6.19, 6.27]	100%
Baseline RN	0.2050	0.0091	[0.19, 0.22]	100%
Reactive NN	0.0007	0.0067	[-0.01, 0.01]	55.04%
Reactive x RN	-0.0769	0.0095	[-0.09, -0.06]	100%

Note. The intercept term 'Baseline NN' refers to the average shifted lognormal RT to make a correct response for Baseline NN trials. 'Reactive RN' is the key effect (recency effect) and refers to the difference in performance of RN and NN trials across the Reactive and Baseline modes.

There was decisive evidence for a reduced RN Recency Effect in the Reactive mode versus Baseline mode (β = -0.08, se = 0.01, HDI = [-0.09, -0.06], pd = 100%).

Table 2: Hypothesis Test for Rea-Bas RN Recency Effect

Hypothesis	Estimate	SE	HDI	SDR	Post.Prob
Reactive - Baseline RN Recency Effect	-0.05	0.01	[-0.09, -0.06]	0.00	0.00

Note. The key term in this table is the SDR score, which is computed as the ratio of the posterior distribution to the prior distribution at a specific point, in this case the mean of the prior distribution to investigate whether there is an increased likelihood of this value as a function of incoming data.

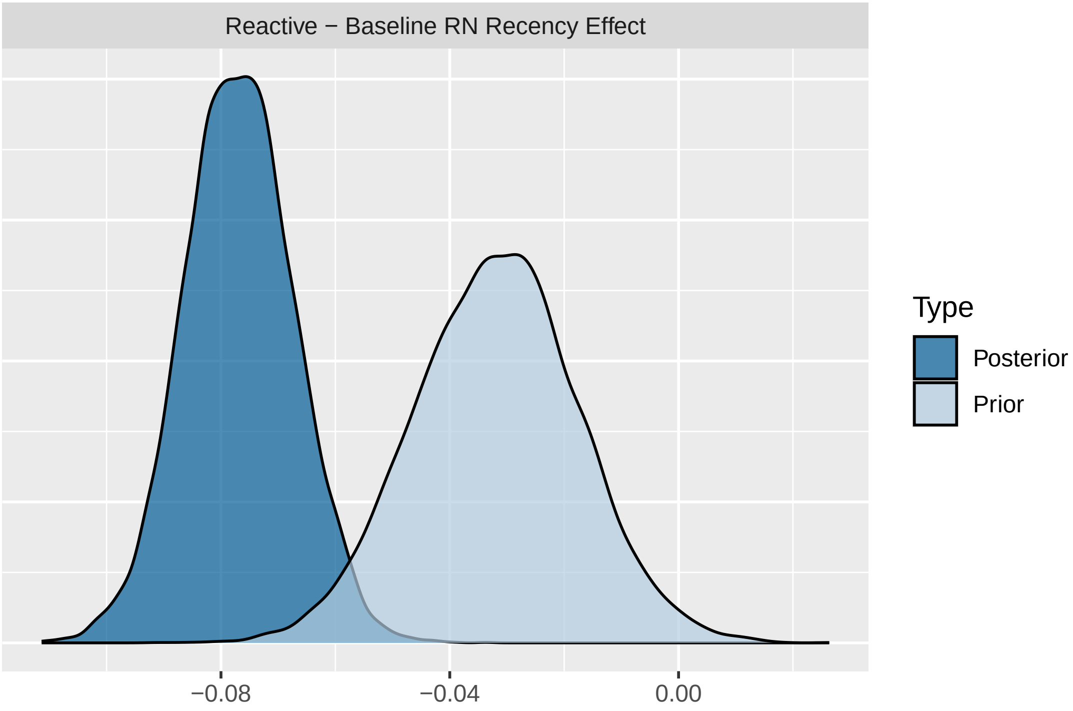

The plot indicates that the posterior is shifted away from the prior distribution. The graph is centered at the mean value of 2018 estimate, and indicates the SDR to be less than one (0) at that point. This suggests that the prior underestimates the reduction in the RN Recency Effect across the Reactive and Baseline modes. More generally, the pattern replicates that reported in Tang et al (2023).

3.6.2 Reactive - Proactive RN Recency Effect

Table 3: Reactive - Proactive RN Recency Effect

Term	Estimate	SE	HDI	pd
Proactive NN	6.2582	0.0192	[6.22, 6.3]	100%
Proactive RN	0.2443	0.0081	[0.23, 0.26]	100%
Reactive NN	0.0052	0.0065	[-0.01, 0.02]	78.7%
Reactive x RN	-0.1253	0.0091	[-0.14, -0.11]	100%

Note. The intercept term 'Proactive NN' refers to the average average shifted lognormal RT to make a correct response for Proactive NN trials. 'Reactive x RN' is the key effect (recency effect) and refers to the difference in performance of RN and NN trials between the Reactive and Proactive mode.

There was decisive evidence for a reduced RN Recency Effect in the Reactive mode relative to the Proactive mode (β = -0.13, se = 0.01, HDI = [-0.14, -0.11], pd = 100%).

Table 4: Hypothesis Test for Rea-Pro RN Recency Effect

Hypothesis	Estimate	SE	HDI	SDR	Post.Prob
Reactive - Proactive RN Recency Effect	-0.03	0.01	[-0.14, -0.11]	0.02	0.00

Note. The key term in this table is the SDR score, which is computed as the ratio of the posterior distribution to the prior distribution at a specific point, in this case the mean of the prior distribution to investigate whether there is an increased likelihood of this value as a function of incoming data.

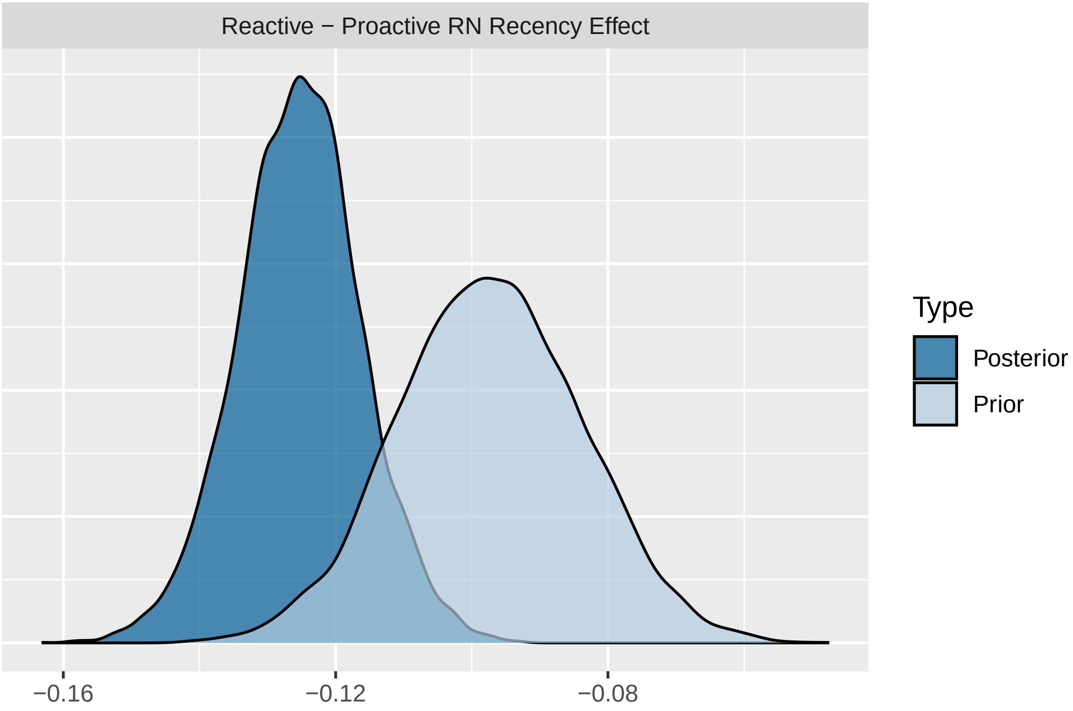

The plot indicates that the posterior is shifted away from the prior distribution. The graph is centered at the mean value of 2018 estimate, and indicates the SDR to be less than one (0.02) at that point. This suggests that the prior underestimates the reduction in the RN Recency effect across the Reactive and Proactive modes. More generally, the pattern replicates that reported in Tang et al (2023).

3.7 Sternberg Single Model Output

Based on the Reviewers' request, we also ran a single model of the 2018/2020 Sternberg data that includes all analyzed predictor levels, and then subsequently extracted relevant contrasts via posterior linear combinations.

3.7.1 2018 NP Error Effect Single Model Output

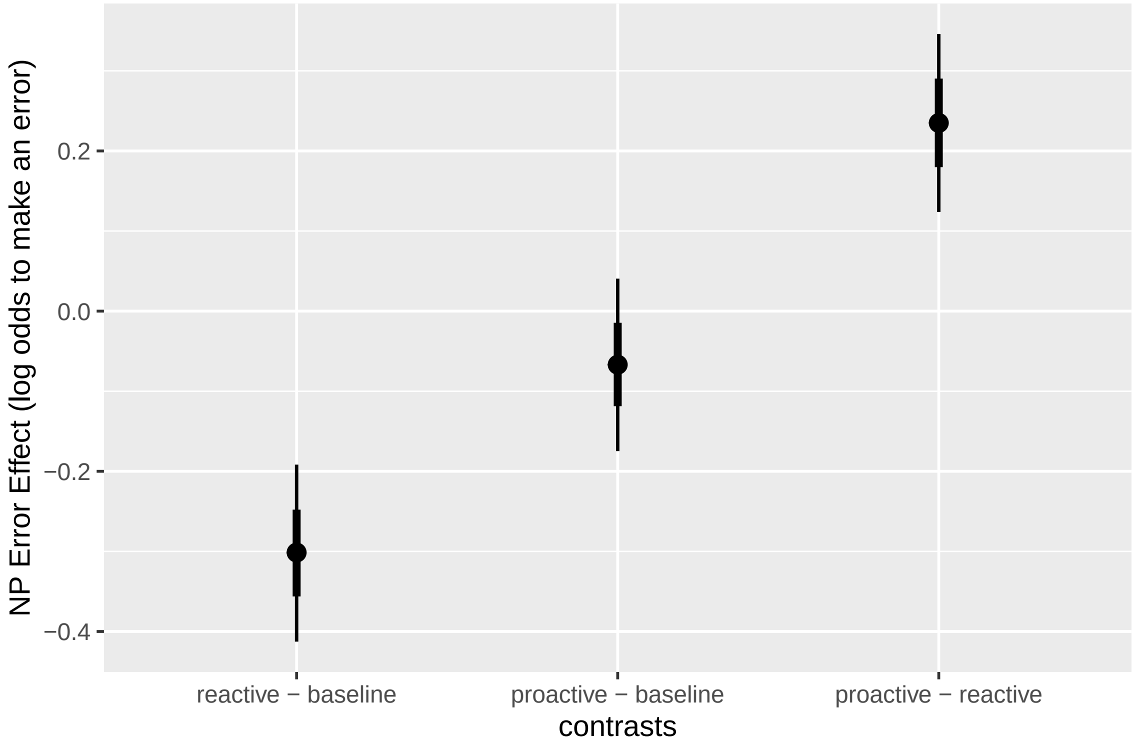

Table 1: NP Error Effect Contrasts

Term	Estimate	HDI	pd
proactive - baseline	-0.0669	[-0.17, 0.04]	88.81%
proactive - reactive	0.2349	[0.12, 0.35]	100%
reactive - baseline	-0.3014	[-0.42, -0.2]	100%

Note. Each of these terms reflect the contrasting effects of mode. The key terms are 'proactive - baseline' and 'proactive - reactive', which refer to the difference in the log odds to make an error between the Proactive and Baseline/Reactive modes for critical items.

3.7.2 2020 NP Error Effect Single Model Output

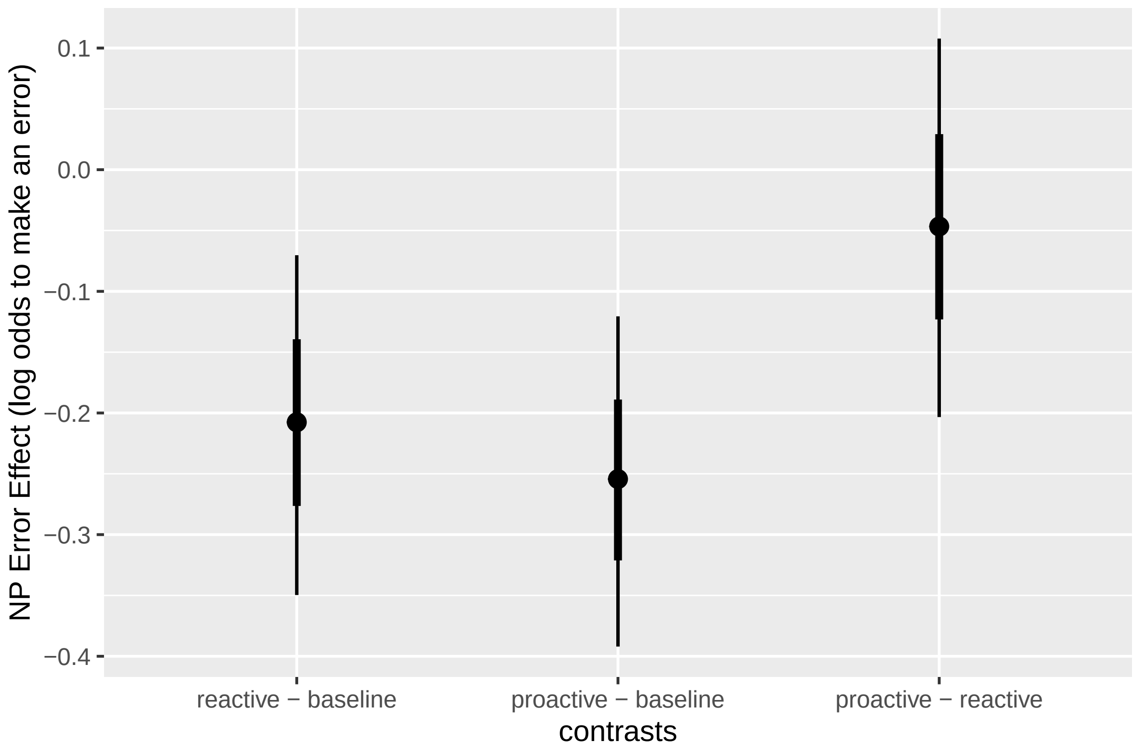

Table 2: NP Error Effect Contrasts

Term	Estimate	HDI	pd
proactive - baseline	-0.2543	[-0.39, -0.12]	99.99%
proactive - reactive	-0.0467	[-0.2, 0.11]	71.89%
reactive - baseline	-0.2076	[-0.35, -0.07]	99.87%

Note. Each of these terms reflect the contrasting effects of mode. The key terms are ’proactive - baseline’ and ’proactive - reactive’, which refer to the difference in the log odds to make an error between the Proactive and Baseline/Reactive modes for critical items.

3.7.3 2018 NP RT Effect Single Model Output (ex-Gaussian)

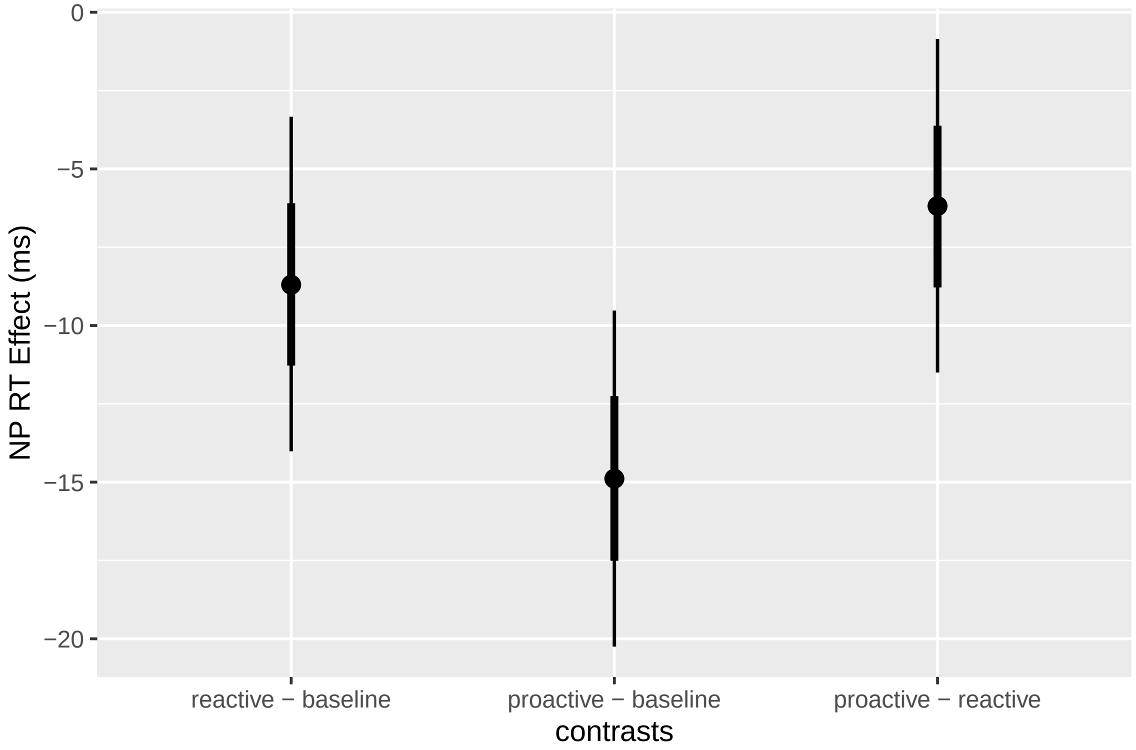

Table 3: NP RT Effect Contrasts

Term	Estimate	HDI	pd
proactive - baseline	-14.8900	[-20.36, -9.66]	100%
proactive - reactive	-6.1858	[-11.53, -0.89]	98.91%
reactive - baseline	-8.6987	[-14.05, -3.38]	99.94%

Note. Each of these terms reflect the contrasting effects of mode for critical items. The key terms are 'proactive - baseline' and 'proactive - reactive', which refer to the difference in RT between the Proactive and Baseline/Reactive modes for critical items.

3.7.4 2020 NP RT Effect Single Model Output (ex-Gaussian)

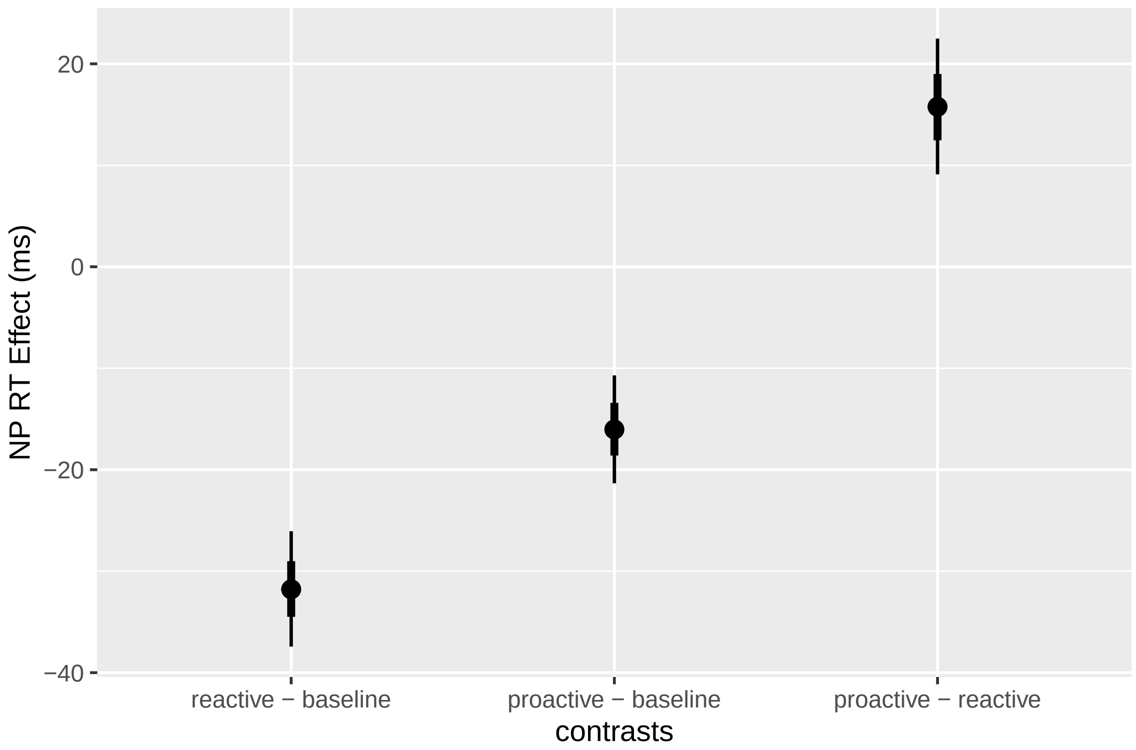

Table 4: NP RT Effect Contrasts

Term	Estimate	HDI	pd
proactive - baseline	-16.0314	[-16.03, -21.26]	100%
proactive - reactive	15.7649	[15.76, 9.28]	100%
reactive - baseline	-31.7846	[-31.78, -37.48]	100%

Note. Each of these terms reflect the contrasting effects of mode for critical items. The key terms are 'proactive - baseline' and 'proactive - reactive', which refer to the difference in the RT between the Proactive and Baseline/Reactive modes for critical items.

```
## # Proportion of samples inside the ROPE [-5.00, 5.00]:
##
## contrast              | TrialType | ItemType | inside ROPE
## -----------------------------------------------------------
## reactive - baseline  |        NP | critical |      0.00 %
## proactive - baseline |        NP | critical |      0.00 %
## proactive - reactive |        NP | critical |      0.00 %
```

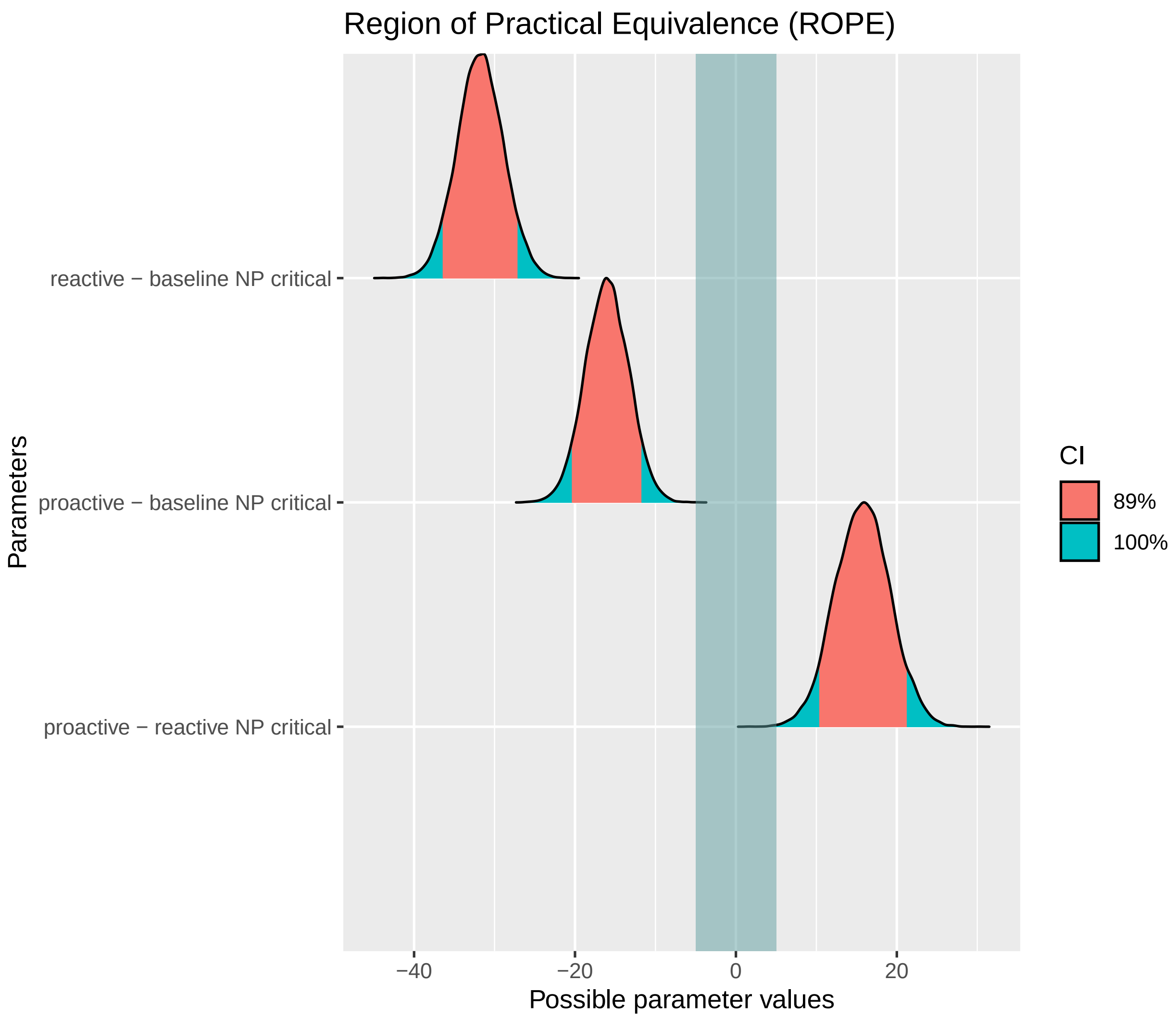

3.7.5 2018 NP RT effect Single Model Output (shifted log-normal)

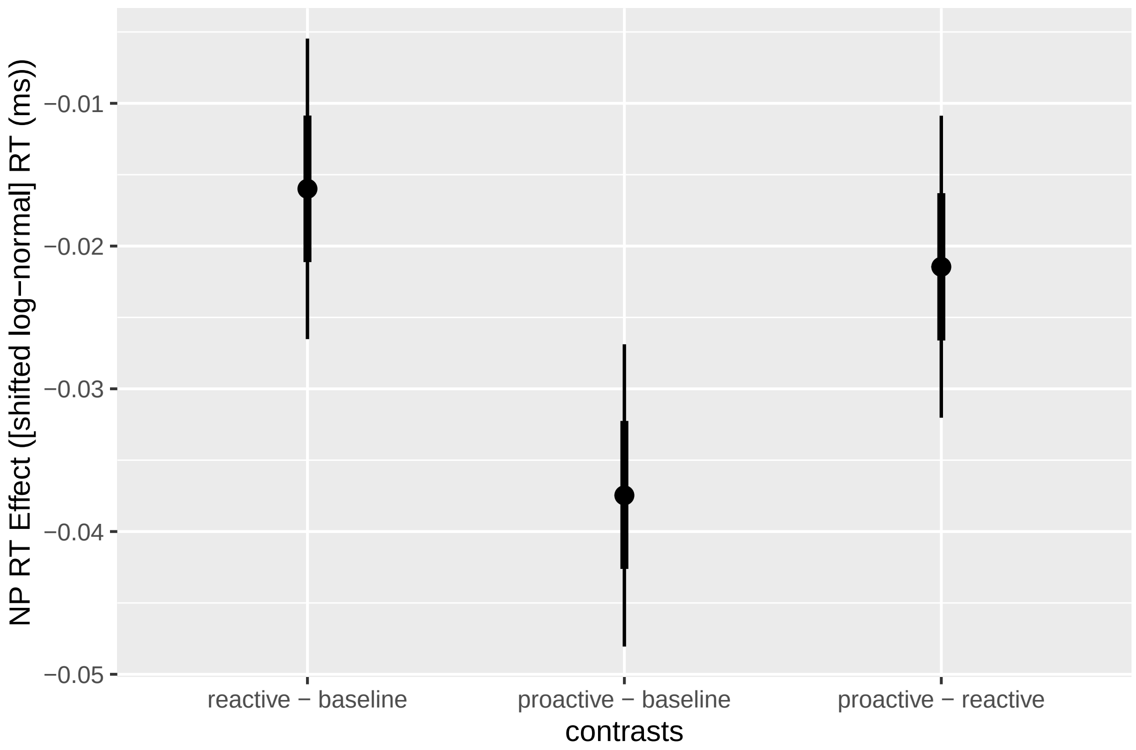

Table 5: NP RT Effect Contrasts

Term	Estimate	HDI	pd
proactive - baseline	-0.0375	[-0.05, -0.03]	100%
proactive - reactive	-0.0215	[-0.03, -0.01]	100%
reactive - baseline	-0.0160	[-0.03, -0.01]	99.88%

Note. Each of these terms reflect the contrasting effects of NP RT effect for critical items. The key terms are 'proactive - baseline' and 'proactive - reactive', which refer to the RT difference between the Proactive and Baseline/Reactive modes for critical items.

3.7.6 2018 RN Recency RT Effect Single Model Output (shifted log-normal)

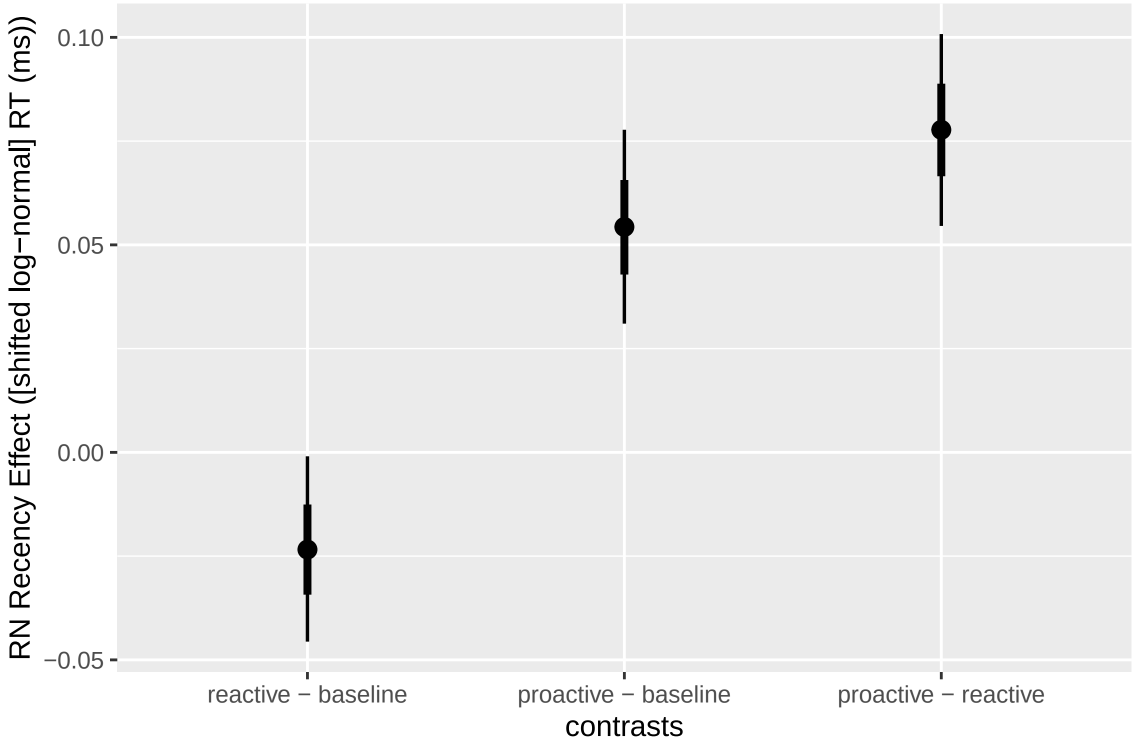

Table 6: RN Recency Effect Contrasts

Term	Estimate	HDI	pd
proactive - baseline RN - NN	0.0543	[0.03, 0.08]	100%
proactive - reactive RN - NN	0.0777	[0.05, 0.1]	100%
reactive - baseline RN - NN	-0.0234	[-0.05, 0]	97.94%

Note. Each of these terms reflect the contrasting effects of Recency RN effect for critical items. The key terms are ’proactive - baseline’ and ’proactive - reactive’, which refer to the RT difference between the Reactive and Baseline/Proactive modes for critical items.

3.7.7 2020 NP RT effect Single Model Output (shifted log-normal)

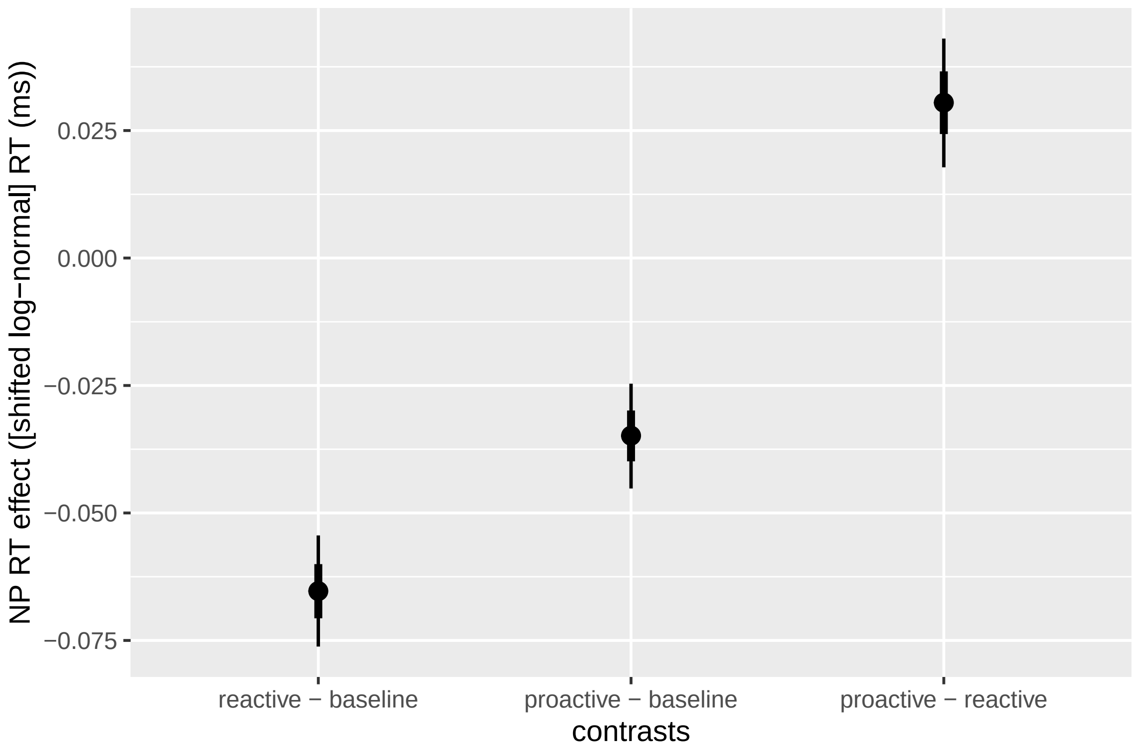

Table 7: NP RT Effect Contrasts

Term	Estimate	HDI	pd
proactive - baseline	-0.0348	[-0.05, -0.02]	100%
proactive - reactive	0.0305	[0.02, 0.04]	100%
reactive - baseline	-0.0653	[-0.08, -0.05]	100%

Note. Each of these terms reflect the contrasting effects of NP RT effect for critical items. The key terms are ’proactive - baseline’ and ’proactive - reactive’, which refer to the RT difference between the Proactive and Baseline/Reactive modes for critical items.

3.7.8 2020 RN Recency RT Effect Single Model Output (shifted log-normal)

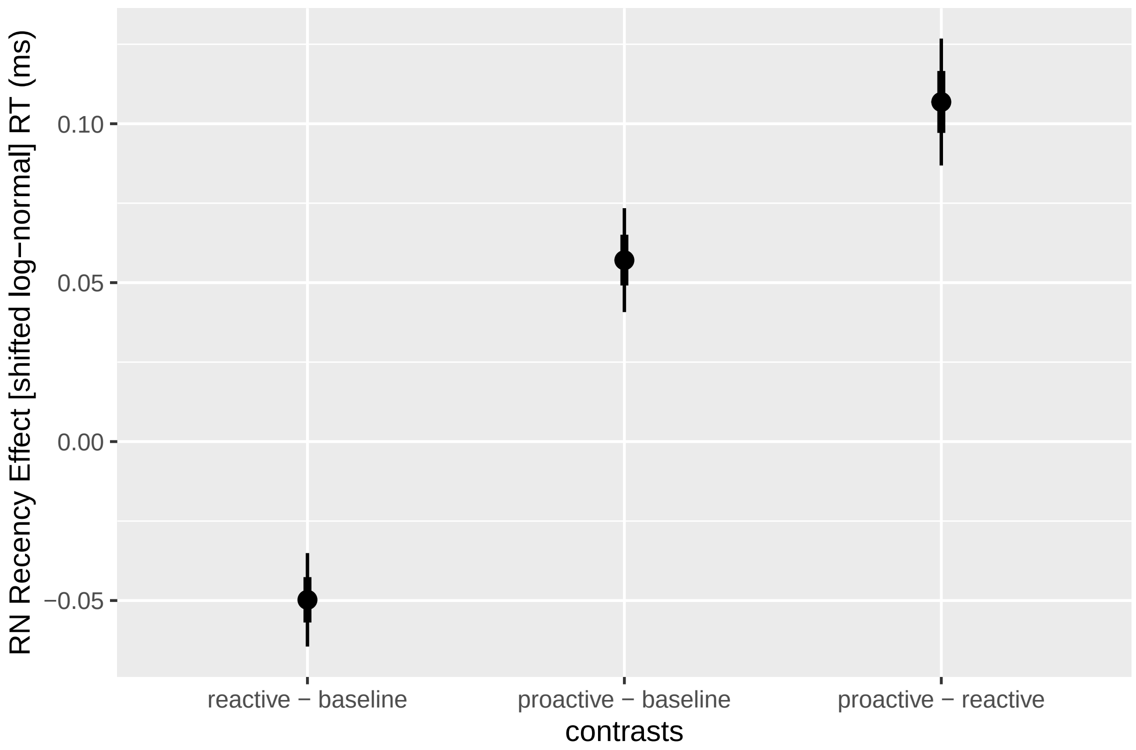

Table 8: RN Recency Effect Contrasts

Term	Estimate	HDI	pd
proactive - baseline RN - NN	0.0571	[0.04, 0.07]	100%
proactive - reactive RN - NN	0.1068	[0.09, 0.13]	100%
reactive - baseline RN - NN	-0.0498	[-0.06, -0.03]	100%

Note. Each of these terms reflect the contrasting effects of Recency RN Effect for critical items. The key terms are 'proactive - baseline' and 'proactive - reactive', which refer to the RT difference between the Reactive and Baseline/Proactive modes for critical items.

4 Stroop

While the below sections will go into more detail for each Stroop indicator, we briefly describe the expected results for differences in performance across conditions.

LWPC Effect: We predict subjects will have 1) a significant reduction of Stroop interference in the Proactive < Baseline mode for biased items, and 2) a significant reduction of Stroop effect in Proactive < Baseline mode for PC-50 items.

ISPC Effect: We predict subjects will have 1) a significant reduction of Stroop interference in the Reactive < Baseline mode for biased items, and 2) no difference in Stroop effect between Reactive and Baseline modes for PC-50 items.

Transfer Cost: We predict subjects will have a significant increase in transfer cost (i.e., increased Stroop effect for biased versus PC-50 items) for Reactive versus Proactive mode.

Congruency Cost: We predict subjects will have an increased congruency cost for Proactive versus Reactive mode.

4.1 LWPC Effect 2018

The LWPC (list-wide proportion congruent) Effect compares Proactive and Baseline modes. Proactive is list-wide mostly incongruent, but Baseline is mostly congruent. Thus Stroop interference is expected to be reduced in Proactive. Critically, the LWPC effect should transfer to the matched diagnostic (PC-50) items, so when these items are compared, a reduction on Stroop interference is also expected.

Wilkinson Notation

$$RT = mode\ x\ con.id + \left(1 + con.id \mid ID\right), \text{family}\left(\text{shifted log-normal}\right)$$

Fully Indexed Notation

$$\begin{gathered}
\text{RT}_{i,t} \sim \text{shifted log-normal}\left(\mu_{i,t}, \sigma, \theta\right) \\
\log\left(\mu_{i,t} + \theta\right) = \beta_{0i} + \beta_1 * mode_t\ x\ \beta_{2i} * con.id_{i,t} \\
\begin{bmatrix}\beta_{0i} \\ \beta_{2i}\end{bmatrix} \sim \text{N}\left(\begin{bmatrix}\beta_0 \\ \beta_2\end{bmatrix}, \Sigma\right) \\
\beta_0, \beta_1, \beta_2 \sim \text{flat}, \Sigma \sim \text{LKJ(1)}, \sigma \sim \text{student-t(3, 0, 2.5)}, \theta \sim \text{uniform(0, minRT)}
\end{gathered}$$

4.1.1 Proactive - Baseline LWPC Effect for Biased Items

Table 1: Proactive - Baseline LWPC Effect for Biased Items

Term	Estimate	SE	HDI	pd
Baseline Congruent	6.0065	0.0210	[5.97, 6.05]	100%
Baseline Incongruent	0.2796	0.0096	[0.26, 0.3]	100%
Proactive Congruent	0.0387	0.0118	[0.02, 0.06]	99.96%
Proactive x Incongruent	-0.1038	0.0057	[-0.11, -0.09]	100%

Note. The intercept term 'Baseline Congruent' refers to the average [shifted log-normal] RT to make a correct response for Baseline congruent trials. 'Proactive x Incongruent' is the key LWPC effect and refers to the difference in Stroop effect between the Proactive and Baseline mode for biased items.

There is decisive evidence for a reduced Stroop interference effect in Proactive versus Baseline mode for biased items (β = -0.1, se = 0.01, HDI = [-0.11, -0.09], pd = 100%).

4.1.2 Proactive - Baseline LWPC Effect for PC-50 Items

Table 2: Proactive - Baseline LWPC Effect for PC-50 items

Term	Estimate	SE	HDI	pd
Baseline Congruent	5.9091	0.0247	[5.86, 5.96]	100%
Baseline Incongruent	0.2767	0.0122	[0.25, 0.3]	100%
Proactive Congruent	0.0154	0.0115	[-0.01, 0.04]	91.15%
Proactive x Incongruent	-0.0432	0.0058	[-0.05, -0.03]	100%

Note. The intercept term 'Baseline Congruent' refers to the average [shifted log-normal] RT to make a correct response for Baseline congruent trials. 'Proactive x Incongruent' is the key LWPC effect and refers to the difference in Stroop effect between the Proactive and Baseline mode for diagnostic items.

There is decisive evidence for a reduced Stroop interference effect in Proactive versus Baseline mode for PC-50 items (β = -0.04, se = 0.01, HDI = [-0.05, -0.03], pd = 100%).

4.2 LWPC Effect 2020

These analyses test for a consistent pattern in the 2020 dataset, using the 2018 estimates as informed priors.

4.2.1 Proactive - Baseline LWPC effect for Biased items

Table 1: Proactive - Baseline LWPC Effect for Biased Items

Term	Estimate	SE	HDI	pd
Baseline Congruent	5.9939	0.0165	[5.96, 6.03]	100%
Baseline Incongruent	0.2839	0.0077	[0.27, 0.3]	100%
Proactive Congruent	0.0032	0.0038	[0, 0.01]	80.21%
Proactive x Incongruent	-0.1116	0.0043	[-0.12, -0.1]	100%

Note. The intercept term 'Baseline Congruent' refers to the average [shifted log-normal] RT to make a correct response for Baseline congruent trials. 'Proactive x Incongruent' is the key LWPC effect and refers to the difference in Stroop effect between the Proactive and Baseline mode for biased items.

There was decisive evidence for a reduced Stroop interference effect in Proactive versus Baseline mode for biased items (β = -0.11, se = 0, HDI = [-0.12, -0.1], pd = 100%).

Table 2: Hypothesis Test for Pro-Bas LWPC Effect for Biased Items

Hypothesis	Estimate	SE	HDI	SDR	Post.Prob
Proactive - Baseline LWPC Effect for Biased Items	-0.01	0.00	[-0.12, -0.1]	0.26	0.00

Note. The key term in this table is the SDR score, which is computed as the ratio of the posterior distribution to the prior distribution at a specific point, in this case the mean of the prior distribution to investigate whether there is an increased likelihood of this value as a function of incoming data.

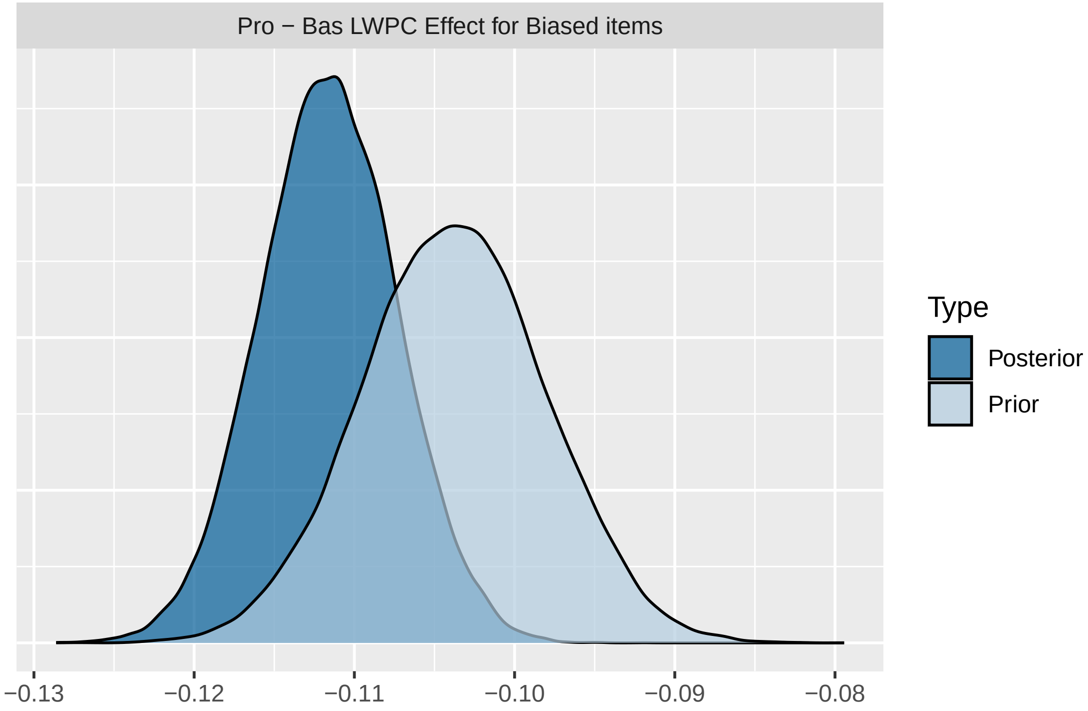

The plot indicates that the posterior shifts away from the prior distribution. The graph is centered at the mean value of 2018 estimate, and indicates the SDR to be less than one (0.26) at that point. This suggests that the prior underestimates the LWPC effect between the Proactive and Baseline modes. More generally, the pattern replicates that reported in Tang et al (2023).

4.2.2 Proactive - Baseline LWPC Effect for PC-50 items

Table 3: Proactive - Baseline LWPC Effect for PC-50 items

Term	Estimate	SE	HDI	pd
Baseline Congruent	5.9607	0.0180	[5.92, 6]	100%
Baseline Incongruent	0.2756	0.0082	[0.26, 0.29]	100%
Proactive Congruent	-0.0249	0.0041	[-0.03, -0.02]	100%
Proactive x Incongruent	-0.0480	0.0045	[-0.06, -0.04]	100%

Note. The intercept term 'Baseline Congruent' refers to the average [shifted log-normal] RT to make a correct response for Baseline congruent trials. 'Proactive x Incongruent' is the key LWPC effect and refers to the difference in Stroop effect between the Proactive and Baseline mode for PC-50 items.

There was also decisive evidence for a reduced Stroop interference effect in the Proactive versus Baseline mode for PC-50 items (β = -0.05, se = 0, HDI = [-0.06, -0.04], pd = 100%).

Table 4: Hypothesis Test for Pro-Bas LWPC Effect for PC-50 items

Hypothesis	Estimate	SE	HDI	SDR	Post.Prob
Proactive - Baseline LWPC Effect for PC-50 items	0.00	0.00	[-0.06, -0.04]	0.72	0.00

Note. The key term in this table is the SDR score, which is computed as the ratio of the posterior distribution to the prior distribution at a specific point, in this case the mean of the prior distribution to investigate whether there is an increased likelihood of this value as a function of incoming data.

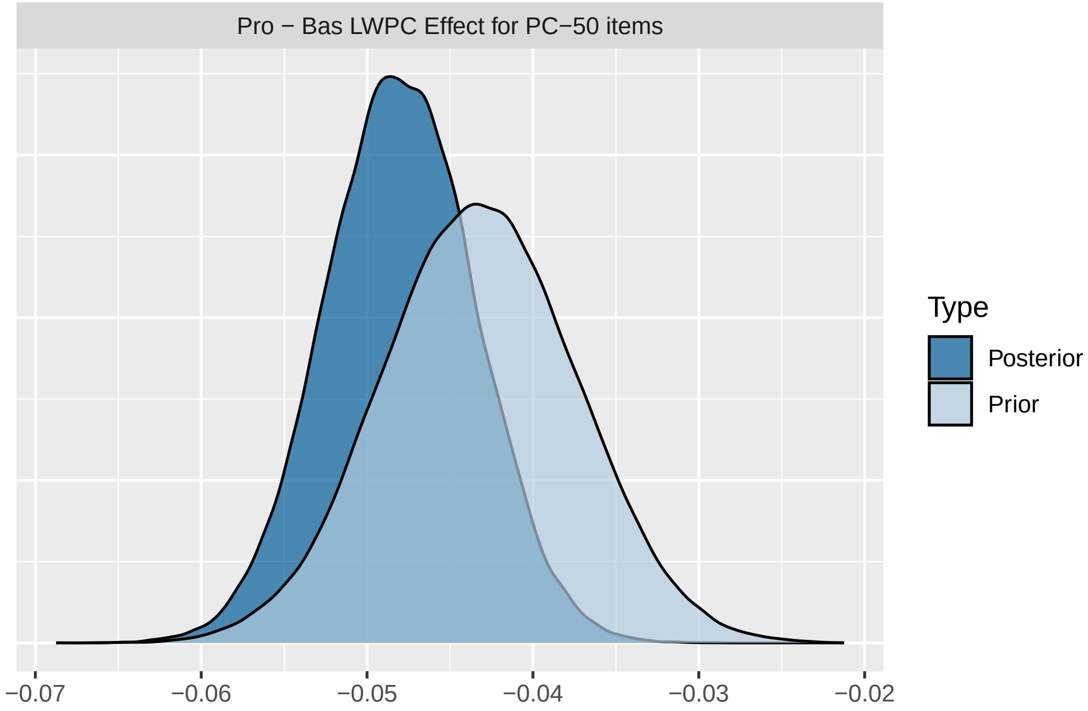

The plot indicates that the posterior shifts away from the prior distribution. The graph is centered at the mean value of 2018 estimate, and indicates the SDR to be less than one (0.72) at that point. This suggests that the prior underestimates the LWPC Effect between the Proactive and Baseline modes. More generally, the pattern replicates that reported in Tang et al (2023).

4.3 ISPC Effect 2018

The ISPC (item-specific proportion congruent) effect compares Reactive and Baseline modes. Both are list-wide mostly congruent, but in the Baseline mode the biased items were also mostly congruent (PC-75), whereas in the Reactive mode, these biased items were mostly incongruent (PC-25), and so can be compared to test for a reduction in Stroop interference in Reactive. However, the ISPC effect should not transfer to diagnostic (PC-50) items, so when these items are compared, no change in Stroop interference is expected.

Wilkinson Notation

$$RT = mode\ x\ con.id + \left(1 + con.id \mid ID\right), \text{family}\left(\text{shifted log-normal}\right)$$

Fully Indexed Notation

$$\begin{gathered}
\text{RT}_{i,t} \sim \text{shifted log-normal}\left(\mu_{i,t}, \sigma, \theta\right) \\
\log\left(\mu_{i,t} + \theta\right) = \beta_{0i} + \beta_1 * mode_t\ x\ \beta_{2i} * con.id_{i,t} \\
\begin{bmatrix}\beta_{0i} \\ \beta_{2i}\end{bmatrix} \sim \text{N}\left(\begin{bmatrix}\beta_0 \\ \beta_2\end{bmatrix}, \Sigma\right) \\
\beta_0, \beta_1, \beta_2 \sim \text{flat}, \Sigma \sim \text{LKJ}(1), \sigma \sim \text{student-t}(3, 0, 2.5), \theta \sim \text{uniform}(0, \text{minRT})
\end{gathered}$$

4.3.1 Reactive - Baseline ISPC Effect for Biased Items

Table 1: Reactive - Baseline ISPC Effect for biased items

Term	Estimate	SE	HDI	pd
Baseline Congruent	5.9894	0.0220	[5.95, 6.03]	100%
Baseline Incongruent	0.2847	0.0106	[0.26, 0.31]	100%
Reactive Congruent	-0.0275	0.0031	[-0.03, -0.02]	100%
Reactive x Incongruent	-0.0735	0.0045	[-0.08, -0.06]	100%

Note. The intercept term 'Baseline Congruent' refers to the average [shifted log-normal] RT to make a correct response for Baseline congruent trials. 'Reactive x Incongruent' is the key ISPC effect and refers to the difference in Stroop effect between the Reactive and Baseline mode for biased items.

There is decisive evidence for a reduced Stroop interference effect in the Reactive versus Baseline mode for biased items (β = -0.07, se = 0, HDI = [-0.08, -0.06], pd = 100%).

4.3.2 Reactive - Baseline ISPC Effect for PC-50 items

Table 2: Reactive - Baseline ISPC Effect

Term	Estimate	SE	HDI	pd
Baseline Congruent	5.9208	0.0238	[5.87, 5.97]	100%
Baseline Incongruent	0.2739	0.0120	[0.25, 0.3]	100%
Reactive Congruent	-0.0079	0.0038	[-0.02, 0]	98.06%
Reactive x Incongruent	0.0094	0.0055	[0, 0.02]	95.89%

Note. The intercept term 'Baseline Congruent' refers to the average [shifted log-normal] RT to make a correct response for Baseline congruent trials. 'Reactive x Incongruent' is the key ISPC effect and refers to the difference in Stroop effect between the Reactive and Baseline mode for PC50 items.

There is only weak evidence for a reduced Stroop interference effect in Reactive versus Baseline modes for PC-50 items (β = 0.01, se = 0.01, HDI = [0, 0.02], pd = 95.89%).

4.4 ISPC Effect 2020

These analyses test for a consistent pattern in the 2020 dataset, using the 2018 estimates as informed priors.

4.4.1 Reactive - Baseline ISPC effect for Biased Items

Table 1: Reactive - Baseline ISPC effect for Biased Items

Term	Estimate	SE	HDI	pd
Baseline Congruent	6.0528	0.0168	[6.02, 6.09]	100%
Baseline Incongruent	0.2772	0.0081	[0.26, 0.29]	100%
Reactive Congruent	-0.0471	0.0023	[-0.05, -0.04]	100%
Reactive x Incongruent	-0.0928	0.0033	[-0.1, -0.09]	100%

Note. The intercept term 'Baseline Congruent' refers to the average [shifted log-normal] RT to make a correct response for Baseline congruent trials. 'Reactive Incongruent' is the key ISPC Effect and refers to the difference in Stroop effect between the Reactive and Baseline mode.

There was decisive evidence for a reduced Stroop interference effect in the Reactive versus Baseline mode for biased items (β = -0.09, se = 0, HDI = [-0.1, -0.09], pd = 100%).

Table 2: Hypothesis Test for Rea-Bas ISPC Effect for Biased items

Hypothesis	Estimate	SE	HDI	SDR	Post.Prob
Reactive - Baseline ISPC Effect for Biased items	-0.02	0.00	[-0.1, -0.09]	0.00	0.00

Note. The key term in this table is the SDR score, which is computed as the ratio of the posterior distribution to the prior distribution at a specific point, in this case the mean of the prior distribution to investigate whether there is an increased likelihood of this value as a function of incoming data.

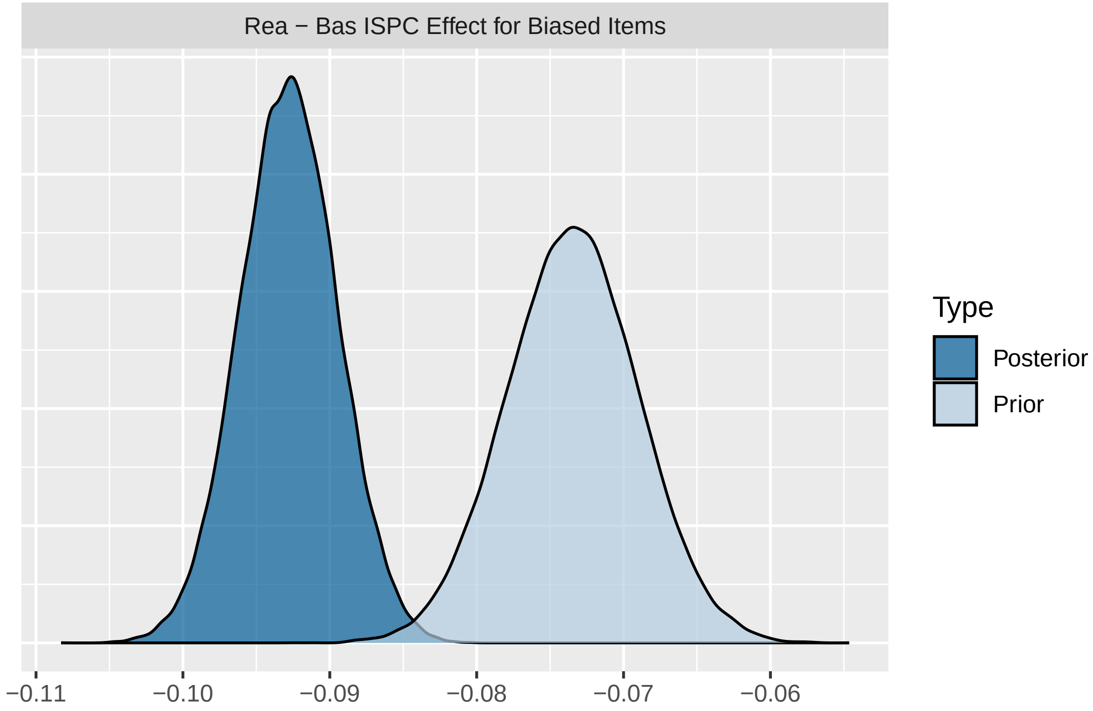

The plot indicates that the posterior is shifted away from the prior distribution. The graph is centered at the mean value of 2018 estimate, and indicates the SDR to be less than one (0) at that point. This suggests that the prior underestimates the ISPC Effect between the Reactive and Baseline modes for biased items. More generally, the pattern replicates that reported in Tang et al (2023).

4.4.2 Reactive - Baseline ISPC effect for PC-50 items

Table 3: Reactive - Baseline ISPC Effect for PC-50 items

Term	Estimate	SE	HDI	pd
Baseline Congruent	5.9643	0.0174	[5.93, 6]	100%
Baseline Incongruent	0.2730	0.0081	[0.26, 0.29]	100%
Reactive Congruent	-0.0331	0.0029	[-0.04, -0.03]	100%
Reactive x Incongruent	-0.0148	0.0042	[-0.02, -0.01]	100%

Note. The intercept term 'Baseline Congruent' refers to the average [shifted log-normal] RT to make a correct response for Baseline congruent trials. 'Reactive x Incongruent' is the key ISPC Effect and refers to the difference in Stroop effect between the Reactive and Baseline mode.

However, there was also decisive evidence for a reduced Stroop interference effect in the Reactive versus Baseline modes for PC-50 items (β = -0.01, se = 0, HDI = [-0.02, -0.01], pd = 100%).

Table 4: Hypothesis Test for Reactive-Baseline ISPC Effect for PC-50 items

Hypothesis	Estimate	SE	HDI	SDR	Post.Prob
Reactive - Baseline ISPC Effect for PC50 items	-0.02	0.00	[-0.02, -0.01]	0.00	0.00

Note. The key term in this table is the SDR score, which is computed as the ratio of the posterior distribution to the prior distribution at a specific point, in this case the mean of the prior distribution to investigate whether there is an increased likelihood of this value as a function of incoming data.

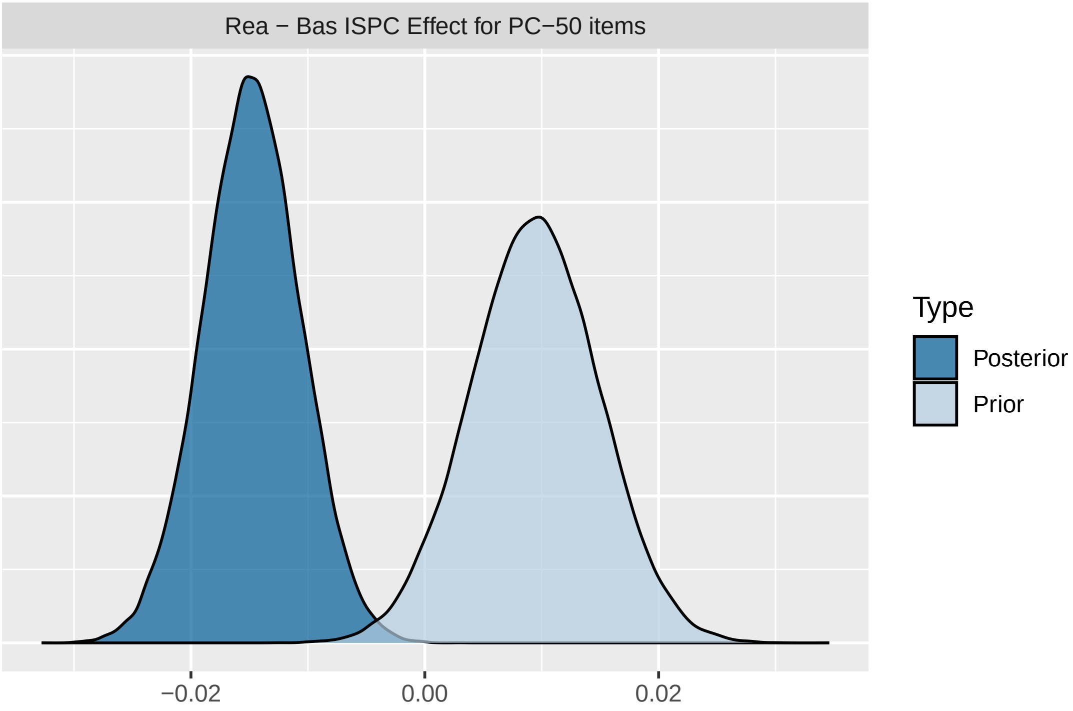

The plot indicates that the posterior is shifted away from the prior distribution. The graph is centered at the mean value of 2018 estimate, and indicates the SDR to be less than one (0) at that point. This suggests that the prior underestimates the ISPC Effect between the Reactive and Baseline modes for PC-50 items. More generally, the pattern contrasts with that reported in Tang et al (2023) in which there is no significant ISPC Effect for the PC-50 items. However, this effect was also not reliable across samples.

4.5 Transfer Cost 2018

Transfer cost compares the difference between Proactive and Reactive modes in terms of Stroop effect between PC-50/diagnostic and biased/inducer items. The predictors ‘item.id’ and ‘con.id’ are dummy coded and treated as interacting variables to make direct comparisons in the Stroop effect between diagnostic and inducer items for the Proactive and Reactive modes respectively. The predictor ‘mode’ was additionally dummy coded and treated as an interacting variable to the aforementioned predictors to make direct comparisons in transfer cost between Reactive and Proactive. Only the intercept and con.id were entered as random effects nested within subject.

Wilkinson Notation

$$RT = \ mode\ x\ con.id\ x\ item.id\ + \left(1 + \ con.id\ x\ item.id\ \ \mid ID\right),\ \text{family}\left(\text{shifted lognormal}\right)$$

Fully Indexed Notation

$$\begin{gathered}
\mathrm{RT}_{i,t} \sim \text{shifted log-normal}\left(\mu_{i,t}, \sigma, \theta\right) \\
\log\left(\mu_{i,t} + \theta\right) = \beta_{0i} + \beta_1 * mode_t\ x\ \beta_{2i} * con.id_{i,t}\ x\ \beta_{3i} * item.id_{i,t} \\
\begin{bmatrix} \beta_{0i} \\ \beta_{2i} \\ \beta_{3i} \end{bmatrix} \sim \mathrm{N}\left(\begin{bmatrix} \beta_0 \\ \beta_2 \\ \beta_3 \end{bmatrix}, \Sigma\right) \\
\beta_0, \beta_1, \beta_2, \beta_3 \sim \text{flat}, \Sigma \sim \text{LKJ(1)}, \sigma \sim \text{student-t(3, 0, 2.5)}, \theta \sim \text{uniform(0, minRT)}
\end{gathered}$$

4.5.1 Reactive - Proactive Transfer Cost

Table 1: Reactive - Proactive Transfer Cost

Term	Estimate	SE	HDI	pd
Proactive Congruent Biased	6.0370	0.0237	[5.99, 6.08]	100%
Proactive Incongruent Biased	0.1772	0.0085	[0.16, 0.19]	100%
Reactive Congruent Biased	-0.0664	0.0036	[-0.07, -0.06]	100%
Proactive Congruent PC50	0.0213	0.0055	[0.01, 0.03]	99.99%
Reactive Incongruent Biased	0.0310	0.0042	[0.02, 0.04]	100%
Proactive Incongruent PC50	0.0275	0.0058	[0.02, 0.04]	100%
Reactive Congruent PC50	0.0450	0.0052	[0.04, 0.06]	100%
Reactive Incongruent PC50	0.0161	0.0067	[0, 0.03]	99.16%

Note. The intercept term 'Proactive Congruent Biased' refers to the average [shifted log-normal] RT to make a correct response for Proactive congruent biased trials. 'Reactive Incongruent PC50' is the key effect and refers to the difference in Transfer Cost between the Reactive and Proactive mode.

There was strong evidence for a Transfer Cost in the Reactive versus Proactive mode (β = 0.02, se = 0.01, HDI = [0, 0.03], pd = 99.16%).

4.6 Transfer Cost 2020

These analyses test for a consistent pattern in the 2020 dataset, using the 2018 estimates as informed priors.

4.6.1 Reactive - Proactive Transfer Cost

Table 1: Reactive - Proactive Transfer Cost

Term	Estimate	SE	HDI	pd
Proactive Congruent Biased	6.0857	0.0168	[6.05, 6.12]	100%
Proactive Incongruent Biased	0.1682	0.0057	[0.16, 0.18]	100%
Reactive Congruent Biased	-0.0710	0.0023	[-0.08, -0.07]	100%
Proactive Congruent PC50	0.0174	0.0035	[0.01, 0.02]	100%
Reactive Incongruent Biased	0.0188	0.0027	[0.01, 0.02]	100%
Proactive Incongruent PC50	0.0286	0.0039	[0.02, 0.04]	100%
Reactive Congruent PC50	0.0441	0.0033	[0.04, 0.05]	100%
Reactive Incongruent PC50	0.0123	0.0044	[0, 0.02]	99.73%

Note. The intercept term 'Proactive Congruent Biased' refers to the average [shifted log-normal] RT to make a correct response for Proactive congruent biased trials. 'Reactive Incongruent PC50' is the key effect and refers to the difference in Transfer Cost between the Reactive and Proactive mode.

There was strong evidence for a Transfer Cost in Reactive versus Proactive mode (β = 0.01, se = 0, HDI = [0, 0.02], pd = 99.73%).

Table 2: Hypothesis Test for Rea-Pro Transfer Cost

Hypothesis	Estimate	SE	HDI	SDR	Post.Prob
Rea-Pro Transfer Cost	0.00	0.00	[0, 0.02]	1.35	1.00

Note. The key term in this table is the SDR score, which is computed as the ratio of the posterior distribution to the prior distribution at a specific point, in this case the mean of the prior distribution to investigate whether there is an increased likelihood of this value as a function of incoming data.

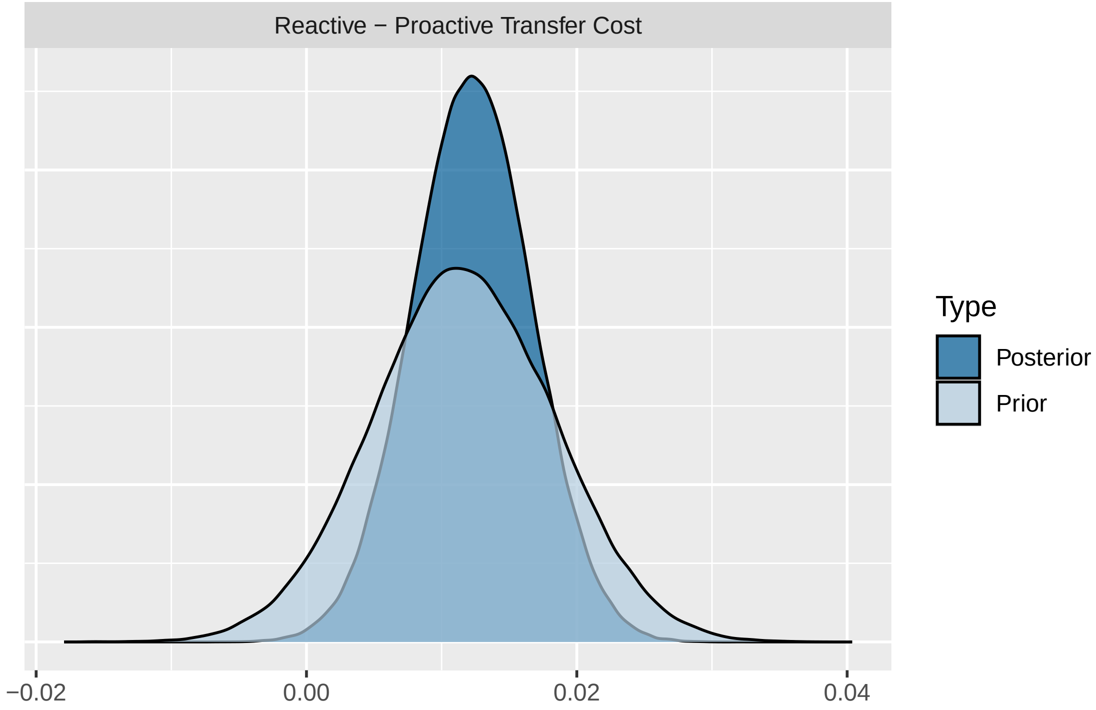

The plot indicates that the posterior closely overlaps the prior distribution. The graph is centered at the mean value of 2018 estimate, and the SDR is found to be greater than one (1.35) at that point. This suggests that the general estimate Reactive - Proactive Transfer Cost does not significantly differ across samples. More generally, the pattern across the 2018 and 2020 datasets confirms the Reactive > Proactive Transfer Cost effect from Tang et al (2023).

4.7 Congruency Cost 2018

The congruency cost is modeled as the difference in [shifted log-normal] RTs between modes to make a correct response for a congruent trial. Only PC-50/diagnostic items are compared to test the prediction that RT will be greater in Proactive relative to Reactive. 'Mode' was dummy coded, but only congruent trials were used to compute differences in congruent [shifted-lognormal] RTs between modes.

Wilkinson Notation

$$RT = mode + \left(1 + mode \mid ID\right), \text{ family}\left(\text{shifted log-normal or Gaussian or ex-Gaussian}\right)$$

Fully Indexed Notation

$$\begin{gathered}\text{RT}_{i,t} \sim \text{shifted log-normal}\left(\mu_{i,t}, \sigma, \theta\right)\\ \log\left(\mu_{i,t} + \theta\right) = \beta_{0i} + \beta_{1i} * mode_{i,t}\\ \begin{bmatrix}\beta_{0i}\\ \beta_{1i}\end{bmatrix} \sim \text{N}\left(\begin{bmatrix}\beta_0\\ \beta_1\end{bmatrix}, \Sigma\right)\\ \beta_0, \beta_1 \sim \text{flat}, \Sigma \sim \text{LKJ(1)}, \sigma \sim \text{student-t(3, 0, 2.5)}, \theta \sim \text{uniform(0, minRT)}\end{gathered}$$

$$\begin{gathered}\text{RT}_{i,t} \sim \text{Gaussian}\left(\mu_{i,t}, \sigma\right)\\ \mu_{i,t} = \beta_{0i} + \beta_{1i} * mode_{i,t}\\ \begin{bmatrix}\beta_{0i}\\ \beta_{1i}\end{bmatrix} \sim \text{N}\left(\begin{bmatrix}\beta_0\\ \beta_1\end{bmatrix}, \Sigma\right)\\ \beta_0, \beta_1 \sim \text{flat}, \Sigma \sim \text{LKJ(1)}, \sigma \sim \text{student-t(3, 0, 132)}\end{gathered}$$

$$\begin{gathered}\text{RT}_{i,t} \sim \text{ex-Gaussian}\left(\mu_{i,t}, \sigma, \tau\right)\\ \left(\mu_{i,t}\right) = \beta_{0i} + \beta_{1i} * mode_{i,t}\\ \begin{bmatrix}\beta_{0i}\\ \beta_{1i}\end{bmatrix} \sim \text{N}\left(\begin{bmatrix}\beta_0\\ \beta_1\end{bmatrix}, \Sigma\right)\\ \beta_0, \beta_1 \sim \text{flat}, \Sigma \sim \text{LKJ(1)}, \sigma \sim \text{student-t(3, 0, 132)}, \tau \sim \text{gamma(1, .1)}\end{gathered}$$

4.7.1 Proactive - Reactive Congruency Cost (Shifted Log-normal)

Table 1: Proactive - Reactive Congruency Cost (Shifted Log-normal)

Term	Estimate	SE	HDI	pd
Reactive Congruent	5.8775	0.0269	[5.82, 5.93]	100%
Proactive Congruent	0.0246	0.0105	[0, 0.05]	98.99%

Note. The intercept term 'Reactive Congruent' refers to the [shifted log-normal] RT to make a correct response for Reactive congruent trials. 'Proactive Congruent' is the key effect (congruency cost) and refers to the difference in performance on congruent trials between the Reactive and Proactive mode on diagnostic items.

There is strong evidence for a Congruency Cost in the Proactive versus Reactive mode for diagnostic items while assuming a shifted log-normal distribution (β = 0.02, se = 0.01, HDI = [0, 0.05], pd = 98.99%).

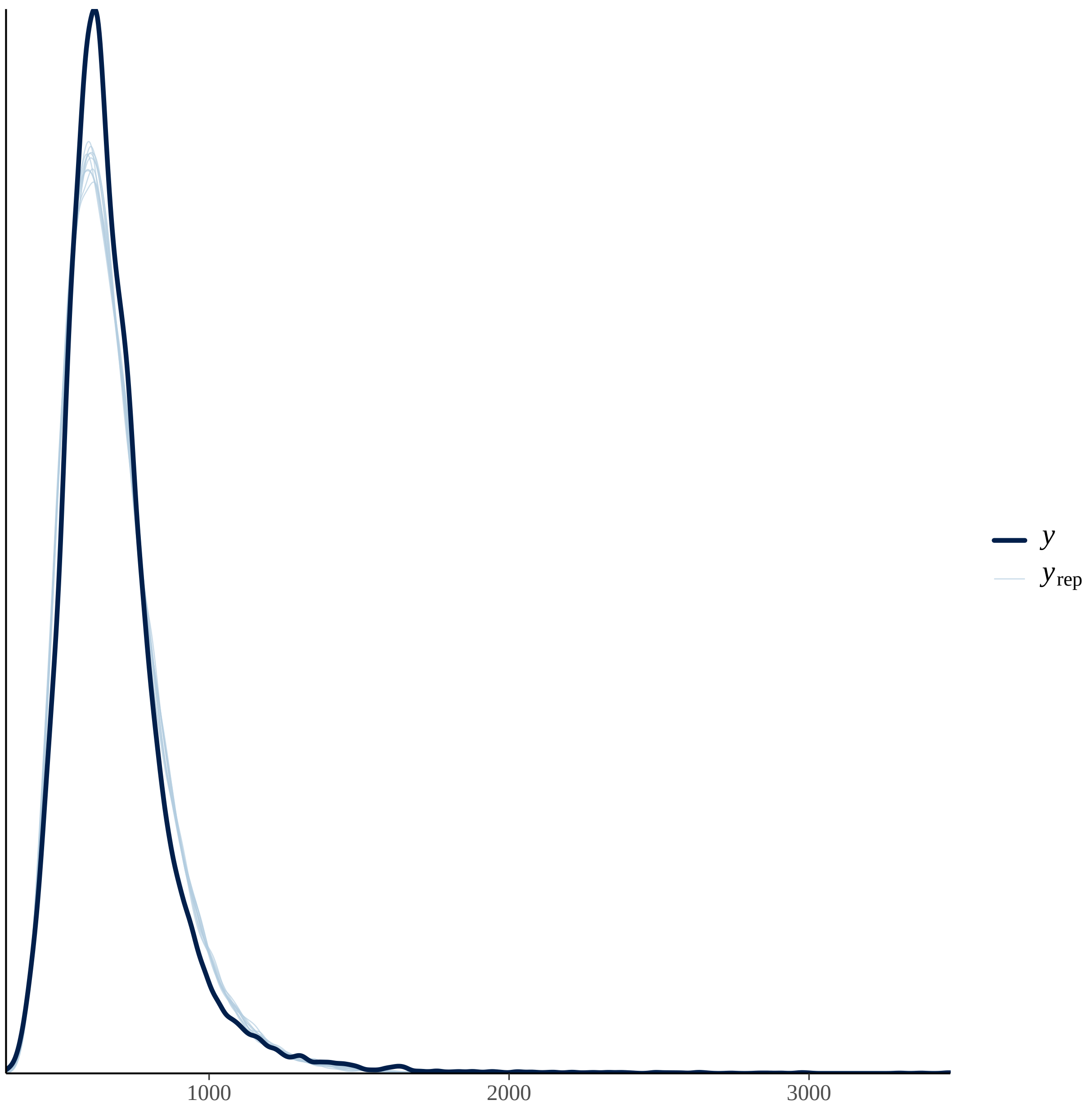

Shifted Log-normal PPC

4.7.2 Proactive - Reactive Congruency Cost (Gaussian)

Table 2: Proactive - Reactive Congruency Cost (Gaussian)

Term	Estimate	SE	HDI	pd
Reactive Congruent	681.7071	10.3385	[660.76, 701.45]	100%
Proactive Congruent	8.9225	5.3700	[-1.81, 19.27]	95.09%

Note. The intercept term 'Reactive Congruent' refers to the average RT to make a correct response for Reactive congruent trials. 'Proactive Congruent' is the key effect (congruency cost) and refers to the difference in performance on congruent trials between the Reactive and Proactive mode on diagnostic items.

However, there is not strong evidence for a Congruency Cost in the Proactive versus Reactive mode for diagnostic items while assuming a Gaussian distribution (β = 8.92, se = 5.37, HDI = [-1.81, 19.27], pd = 95.09%).

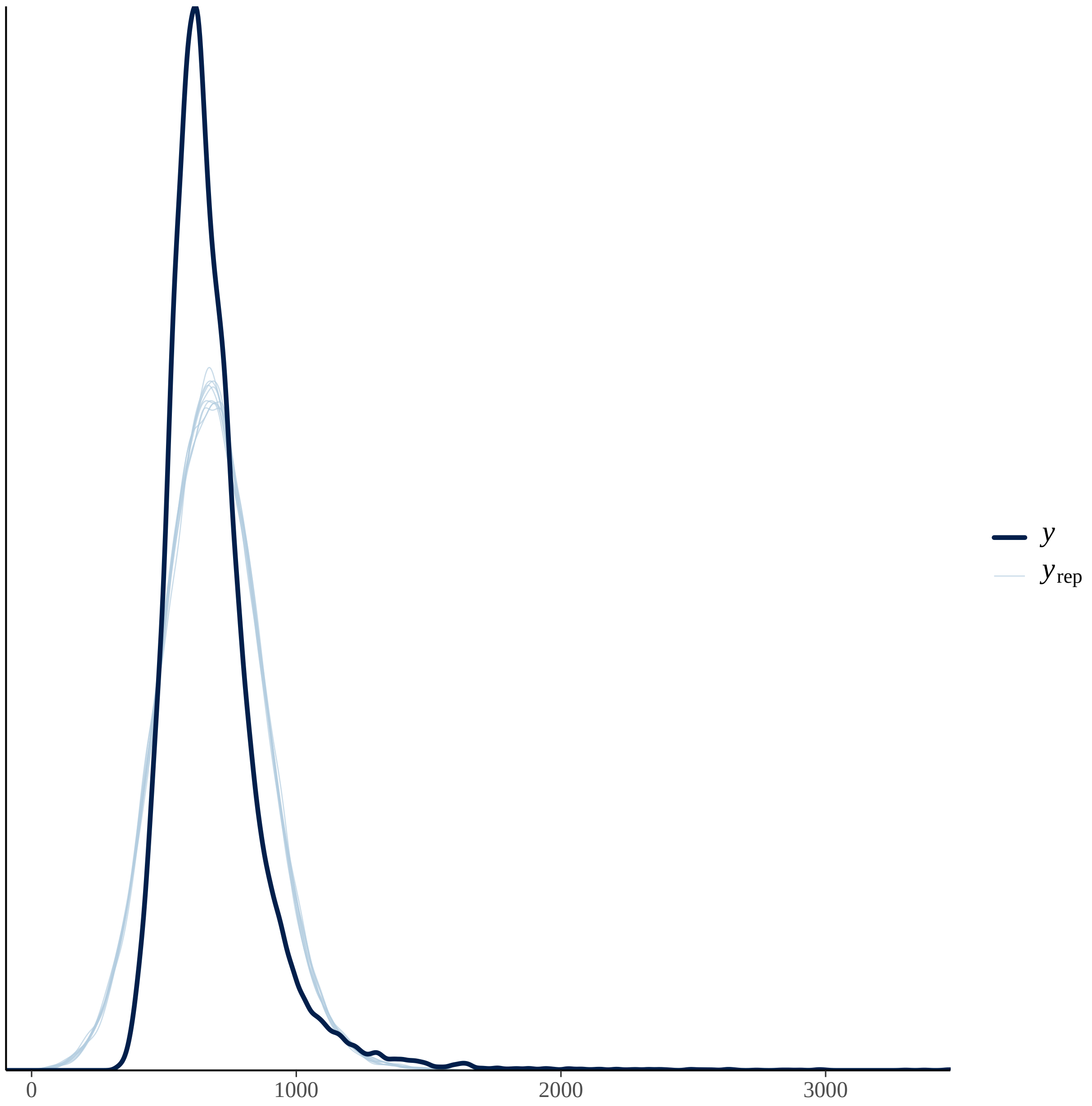

Gaussian PPC

4.7.3 Proactive - Reactive Congruency Cost (ex-Gaussian)

Table 3: Proactive - Reactive Congruency Cost (ex-Gaussian)

Term	Estimate	SE	HDI	pd
Reactive Congruent	680.5517	7.4891	[665.82, 695.2]	100%
Proactive Congruent	9.8665	3.0271	[3.98, 15.8]	99.94%

Note. The intercept term 'Reactive Congruent' refers to the average RT to make a correct response for Reactive congruent trials. 'Proactive Congruent' is the key effect (Congruency Cost) and refers to the difference in performance on congruent trials between the Reactive and Proactive mode.

There is strong evidence for a Congruency Cost in the Proactive versus Reactive mode for diagnostic items while assuming an ex-Gaussian distribution (β = 9.87, se = 3.03, HDI = [3.98, 15.8], pd = 99.94%).

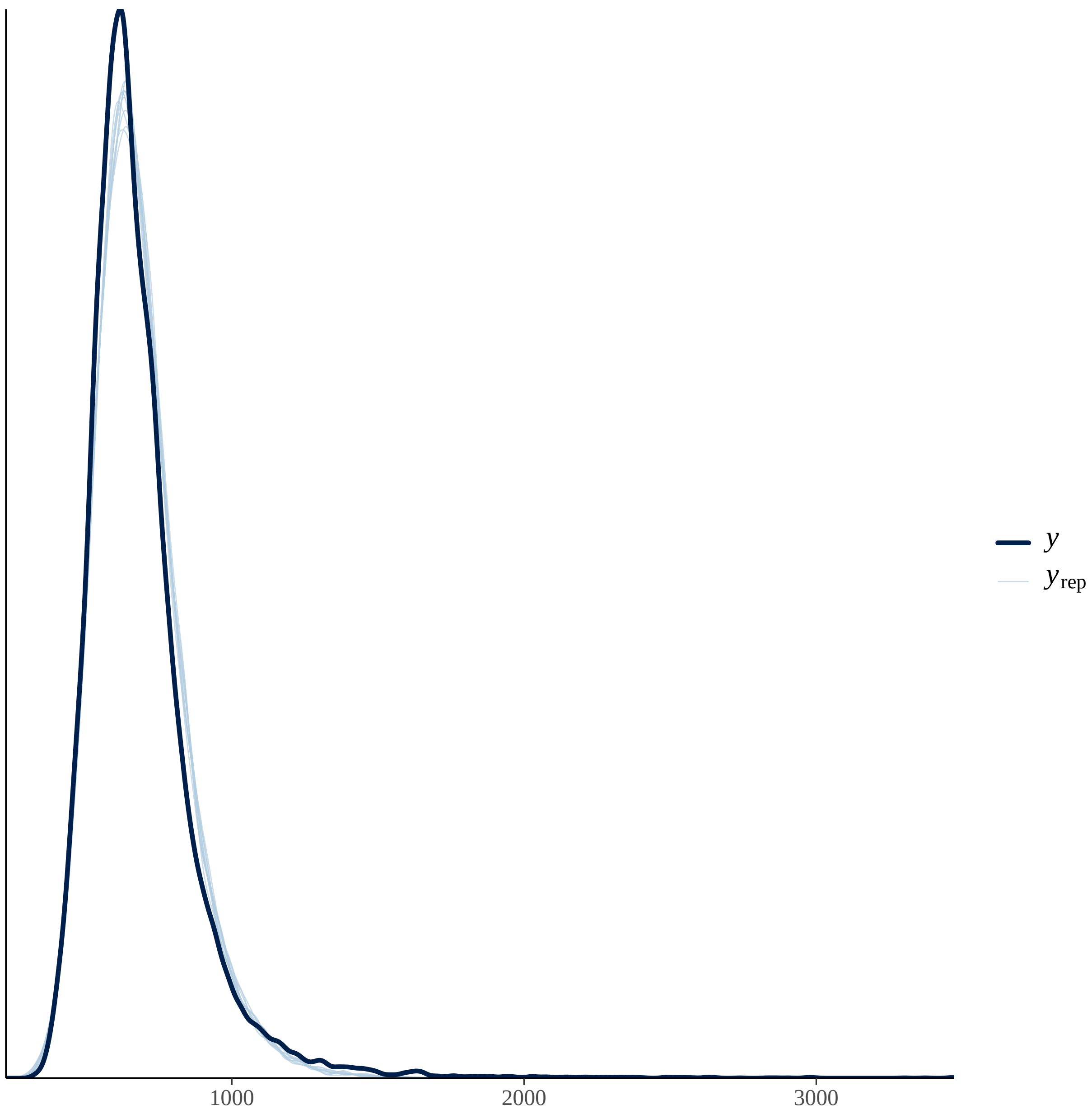

ex-Gaussian PPC

```
## Bayes Factors for Model Comparison
##
##     Model                                         BF
## [1] 0 + Intercept + mode + (1 + mode | ID) 7.40e+24
## [2] 0 + Intercept + mode + (1 + mode | s)  0.00e+00
##
## * Against Denominator: [3] 0 + Intercept + mode + (1 + mode | s)
## *   Bayes Factor Type: marginal likelihoods (bridgesampling)
```

For BF model comparison, we define the ex-Gaussian model as M_0, the shifted log-normal model as M_1 and the Gaussian model as M_2. The BF_{10} » 10, indicating strong evidence favoring M_1 (shifted log-normal) over M_0 (ex-Gaussian). In contrast, BF_{20} is « 1/10, indicating that M_2 (Gaussian) is a substantially worse fit than M_0.

```
## Bayes Factors for Model Comparison
##
##     Model                                        BF
## [1] 0 + Intercept + mode + (1 + mode | s) 1.35e-25
## [2] 0 + Intercept + mode + (1 + mode | s) 0.00e+00
##
## * Against Denominator: [3] 0 + Intercept + mode + (1 + mode | ID)
## *   Bayes Factor Type: marginal likelihoods (bridgesampling)
```

```
## Recompiling the model with 'rstan'
## Recompilation done
## No problematic observations found.  Returning the original 'loo' object.
## Recompiling the model with 'rstan'
## Recompilation done
## No problematic observations found.  Returning the original 'loo' object.

##                       elpd_diff se_diff
## Stroop_reapro_log         0.0       0.0
## Stroop_reapro_exgauss   -66.9      85.8
## Stroop_reapro_gauss   -7563.9     338.3
```

For Loo comparison however, the elpd difference between M_0 (ex-Gaussian) and M_1 (shifted log-normal) is not bigger than twice its standard deviation. This indicates that the numerical difference favoring M_1 over M_0 is not meaningful. However, both M_0 and M_1 have a much higher predictive accuracy than M_2 (Gaussian).

4.7.4 Proactive - Reactive Congruency Cost (inverse Gaussian)

Table 4: Proactive - Reactive Congruency Cost (inverse Gaussian)

Term	Estimate	SE	HDI	pd
Reactive Congruent	680.9999	1.5690	[677.92, 684.06]	100%
Proactive Congruent	9.0448	2.2345	[4.6, 13.33]	99.94%

Note. The intercept term 'Reactive Congruent' refers to the average RT to make a correct response for Reactive congruent trials. 'Proactive Congruent' is the key effect (Congruency Cost) and refers to the difference in performance on congruent trials between the Reactive and Proactive mode.

There is strong evidence for a congruency cost in the Proactive versus Reactive mode for diagnostic items while assuming an inverse Gaussian distribution (β = 9.04, se = 2.23, HDI = [4.6, 13.33], pd = 99.94%).

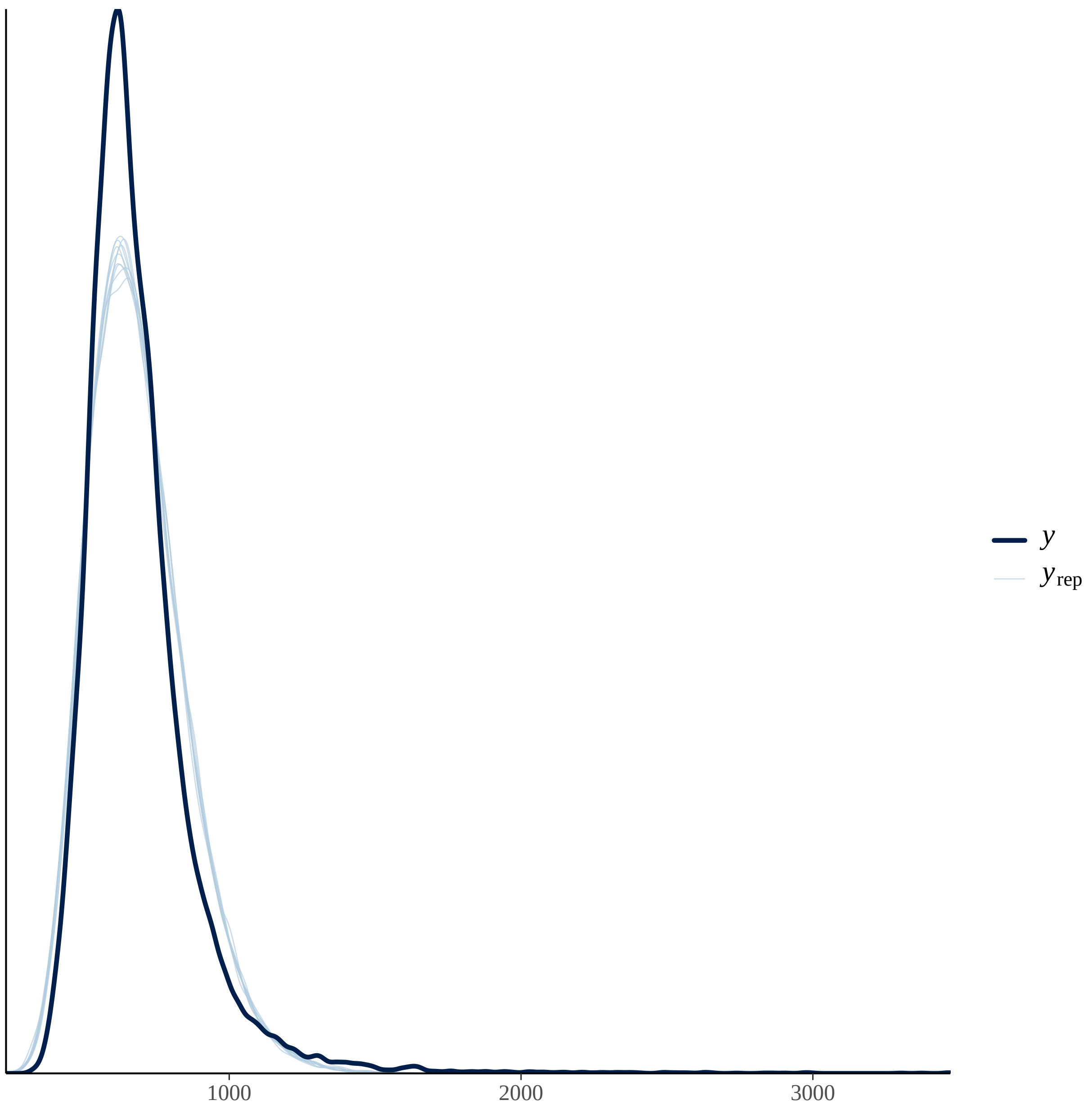

inverse Gaussian PPC

```
## Bayes Factors for Model Comparison
##
##     Model                                         BF
## [1] 0 + Intercept + mode + (1 + mode | ID) 7.43e+24
## [2] 0 + Intercept + mode + (1 + mode | s)  0.00e+00
## [3] 0 + Intercept + mode                   0.00e+00
##
## * Against Denominator: [4] 0 + Intercept + mode + (1 + mode | s)
## *   Bayes Factor Type: marginal likelihoods (bridgesampling)
```

From BF model comparison, the inverse Gaussian model with only fixed effects performs worse than the rest of the models, even the Gaussian model, that contain random effects.

```
## Recompiling the model with 'rstan'
## Recompilation done
## No problematic observations found.  Returning the original 'loo' object.

##                       elpd_diff se_diff
## Stroop_reapro_log         0.0       0.0
## Stroop_reapro_exgauss   -66.9      85.8
## Stroop_reapro_gauss   -7563.9     338.3
## Stroop_reapro_inverse -7919.6     131.0
```

The same conclusion can be drawn from the loo model comparison values.

4.8 Congruency Cost 2020

These analyses test for a consistent pattern in the 2020 dataset, using the 2018 estimates as informed priors.

4.8.1 Proactive - Reactive Congruency Cost (Shifted Log-normal)

Table 1: Proactive - Reactive Congruency Cost

Term	Estimate	SE	HDI	pd
Reactive Congruent	5.8818	0.0199	[5.84, 5.92]	100%
Proactive Congruent	0.0283	0.0082	[0.01, 0.04]	99.97%

Note. The intercept term 'Reactive Congruent' refers to the average [shifted lognormal] RT to make a correct response for Reactive congruent trials. 'Proactive Congruent' is the key effect (Congruency Cost) and refers to the difference in performance on congruent trials between the Reactive and Proactive mode.

There was strong evidence for a Congruency Cost in the Proactive versus Reactive mode for diagnostic items while assuming a shifted log-normal distribution (β = 0.03, se = 0.01, HDI = [0.01, 0.04], pd = 99.97%).

Table 2: Hypothesis Test for Pro-Rea Congruency Cost

Hypothesis	Estimate	SE	HDI	SDR	Post.Prob
Proactive - Reactive Congruency Cost	0.00	0.01	[0.01, 0.04]	1.15	1.00

Note. The key term in this table is the SDR score, which is computed as the ratio of the posterior distribution to the prior distribution at a specific point, in this case the mean of the prior distribution to investigate whether there is an increased likelihood of this value as a function of incoming data.

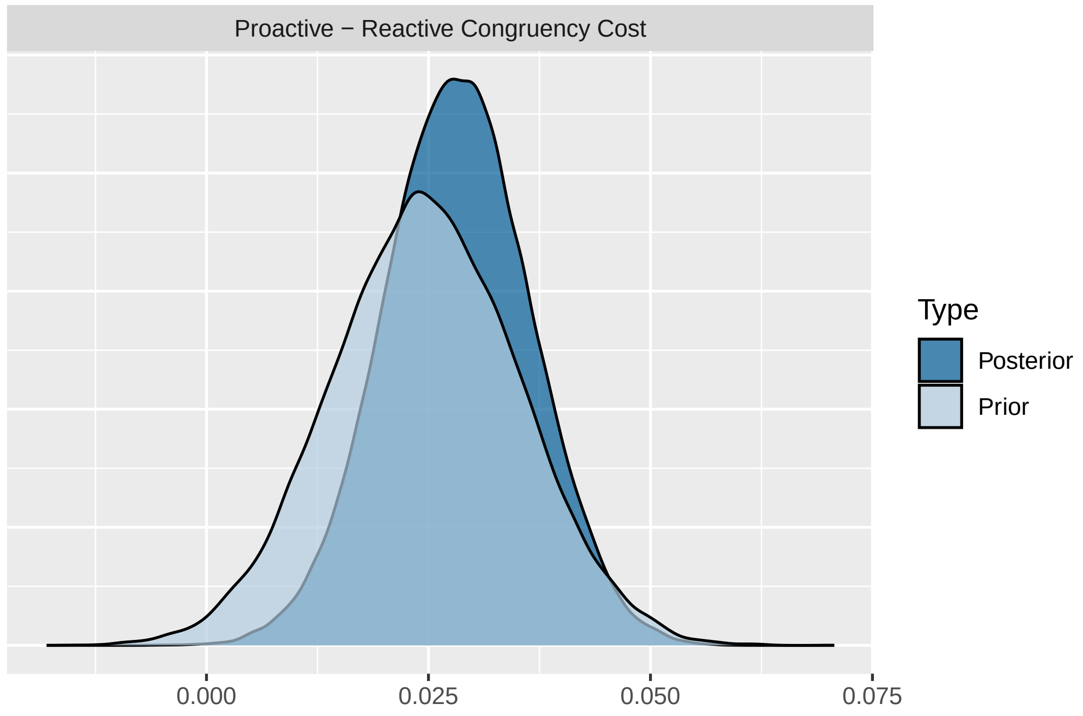

The plot indicates that the posterior closely overlaps with the prior distribution. The graph is centered at the mean value of the 2018 estimate, and the SDR is found to be greater than one (1.15) at that point. This suggests that the posterior values don't differ greatly from the prior, showing that the Congruency Cost for the Proactive versus Reactive mode is stable across samples. More generally, the pattern across the 2018 and 2020 datasets provides stronger evidence in favor of a Congruency Cost than reported in Tang et al (2023).

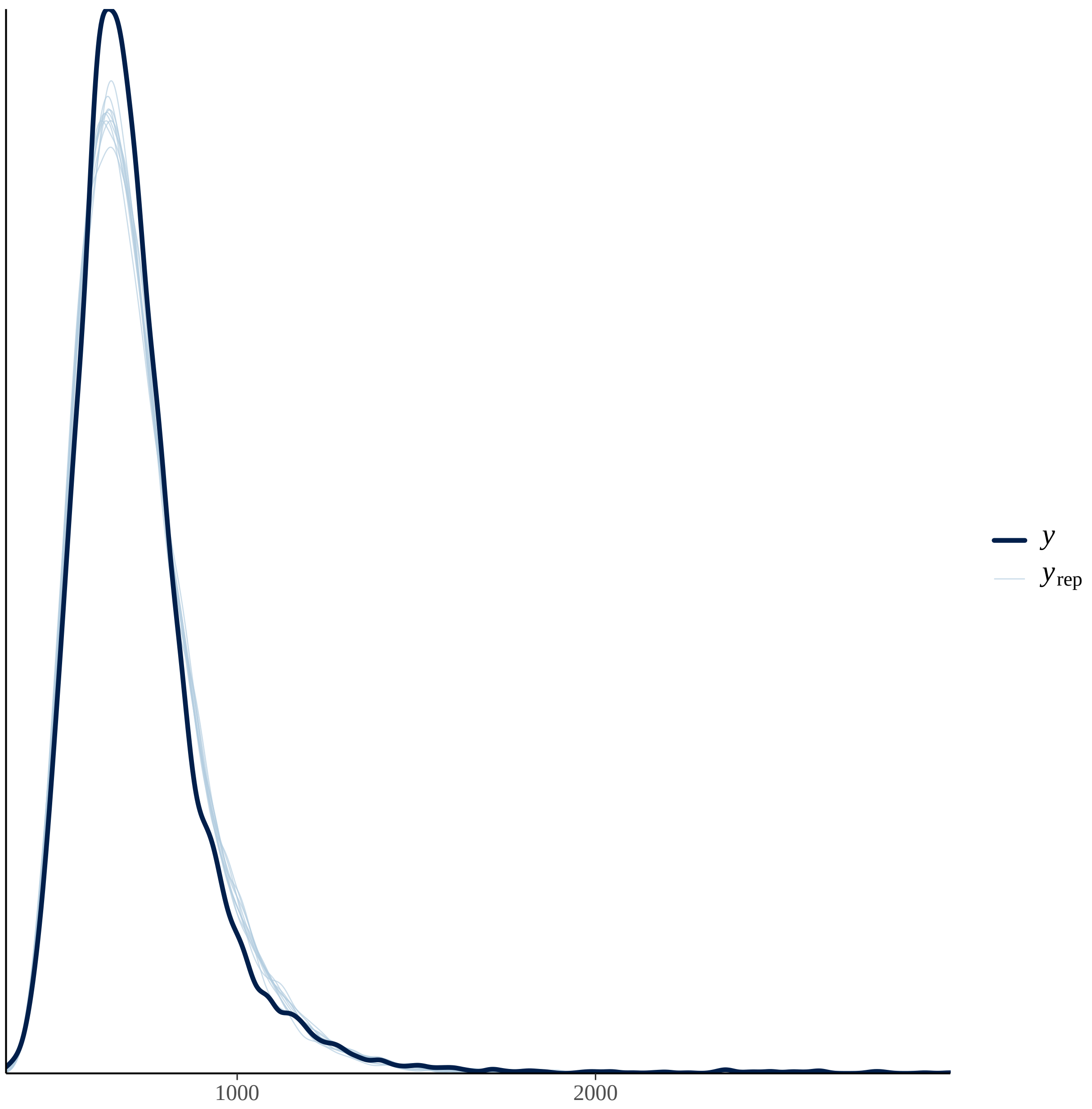

Shifted Log-normal PPC

4.8.2 Proactive - Reactive Congruency Cost (Gaussian)

Table 3: Pro - Rea Congruency Cost (Gaussian)

Term	Estimate	SE	HDI	pd
Reactive Congruent	698.1124	7.7058	[683.06, 713.24]	100%
Proactive Congruent	11.5450	3.8822	[3.98, 19.21]	99.83%

Note. The intercept term 'Reactive Congruent' refers to the average RT to make a correct response for Reactive congruent trials. 'Proactive Congruent' is the key effect (Congruency Cost) and refers to the difference in performance on congruent trials between the Reactive and Proactive mode.

There was strong evidence for a congruency cost in the Proactive versus Reactive mode for diagnostic items while assuming a Gaussian distribution (β = 11.54, se = 3.88, HDI = [3.98, 19.21], pd = 99.83%).

Table 4: Hypothesis Test for Pro-Rea Congruency Cost

Hypothesis	Estimate	SE	HDI	SDR	Post.Prob
Proactive - Reactive Congruency Cost	2.62	3.88	[3.98, 19.21]	1.10	1.00

Note. The key term in this table is the SDR score, which is computed as the ratio of the posterior distribution to the prior distribution at a specific point, in this case the mean of the prior distribution to investigate whether there is an increased likelihood of this value as a function of incoming data.

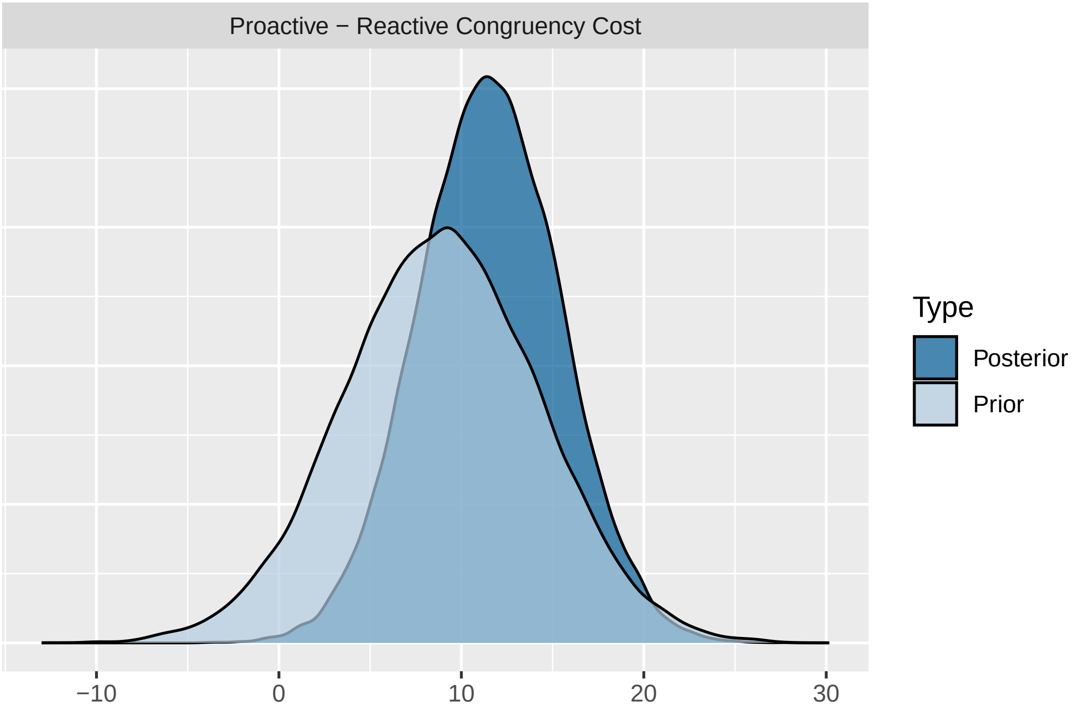

The plot indicates that the posterior closely overlaps the prior distribution. The graph is centered at the mean value of 2018 estimate, and the SDR is found to be greater than one (1.1) at that point. This suggests that the posterior values don't differ greatly from prior, despite the difference in the presence of a Congruency Cost for the Proactive versus Reactive models while assuming a Gaussian distribution across samples.

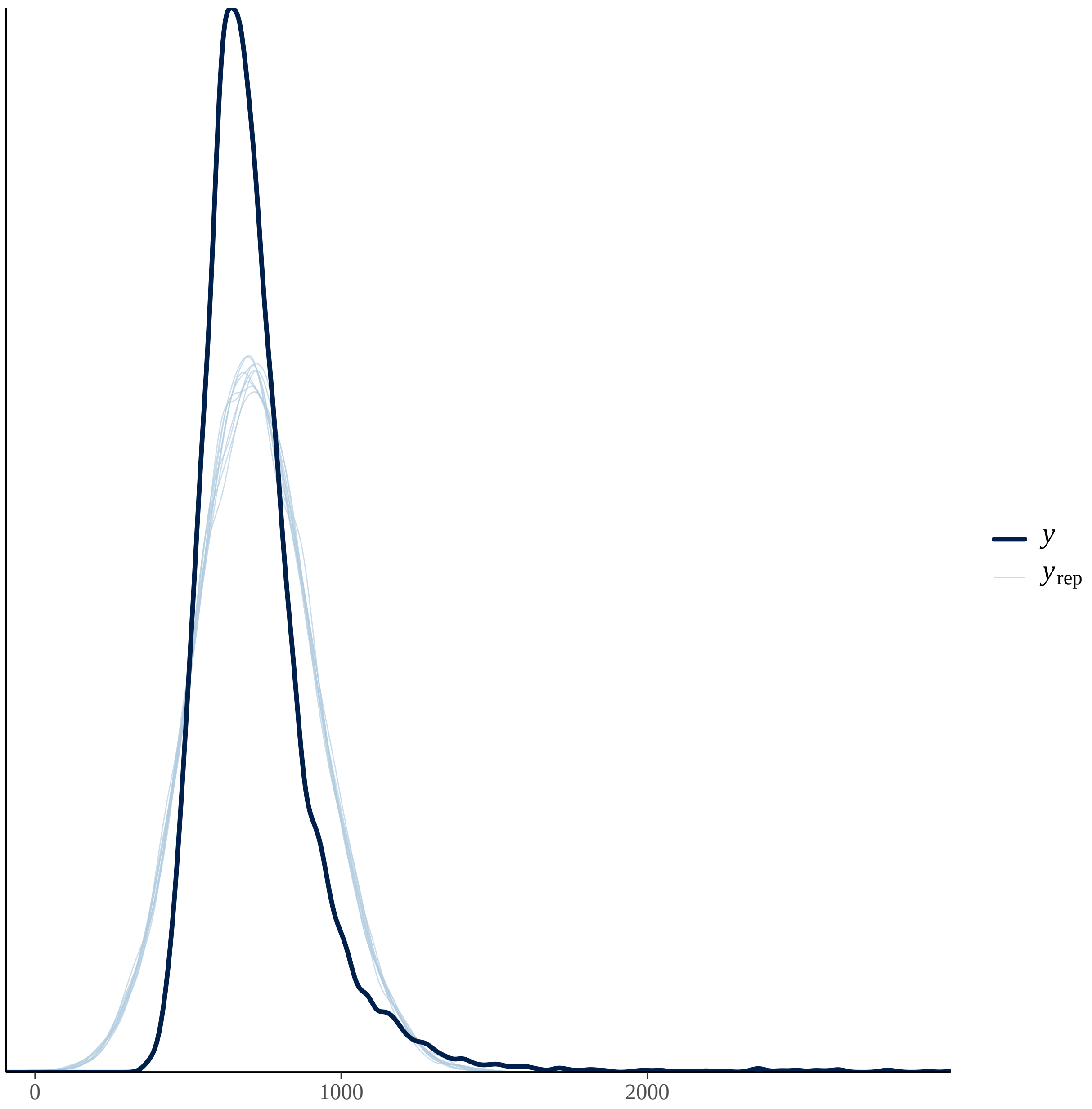

Gaussian PPC

4.8.3 Proactive - Reactive Congruency Cost (ex-Gaussian)

Table 5: Proactive - Reactive Congruency Cost (ex-Gaussian distribution)

Term	Estimate	SE	HDI	pd
Reactive Congruent	696.5408	5.7973	[685.08, 707.83]	100%
Proactive Congruent	11.1111	2.4478	[6.22, 15.77]	100%

Note. The intercept term 'Reactive Congruent' refers to the average RT to make a correct response for baseline congruent trials. 'Proactive Congruent' is the key effect (Congruency Cost) and refers to the difference in performance on congruent trials between the reactive and proactive mode.

There was decisive evidence for a Congruency Cost in the Proactive versus Reactive mode for diagnostic items while assuming an ex-Gaussian distribution (β = 11.11, se = 2.45, HDI = [6.22, 15.77], pd = 100%).

Table 6: Hypothesis Test for Pro-Rea Congruency Cost

Hypothesis	Estimate	SE	HDI	SDR	Post.Prob
Proactive - Reactive Congruency Cost	1.24	2.45	[6.22, 15.77]	1.08	1.00

Note. The key term in this table is the SDR score, which is computed as the ratio of the posterior distribution to the prior distribution at a specific point, in this case the mean of the prior distribution to investigate whether there is an increased likelihood of this value as a function of incoming data.

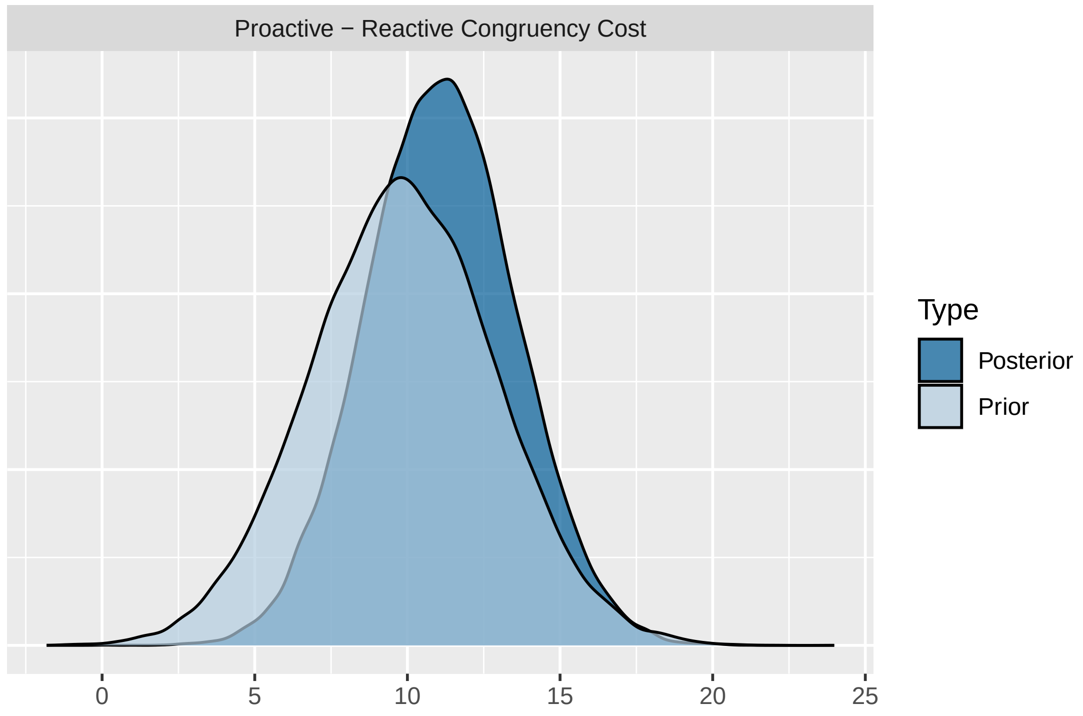

The plot indicates that the posterior distribution closely overlaps with the prior distribution. The graph is centered at the mean value of 2018 estimate, and the SDR is found to be greater than one (1.08) at that point. This suggests that the posterior values don't differ greatly from the prior, showing that the Congruency Cost for the Proactive versus Reactive mode is stable across samples while assuming an ex-Gaussian distribution. More generally, the pattern across the 2018 and 2020 datasets provides stronger evidence in favor of a Congruency Cost than reported in Tang et al (2023).

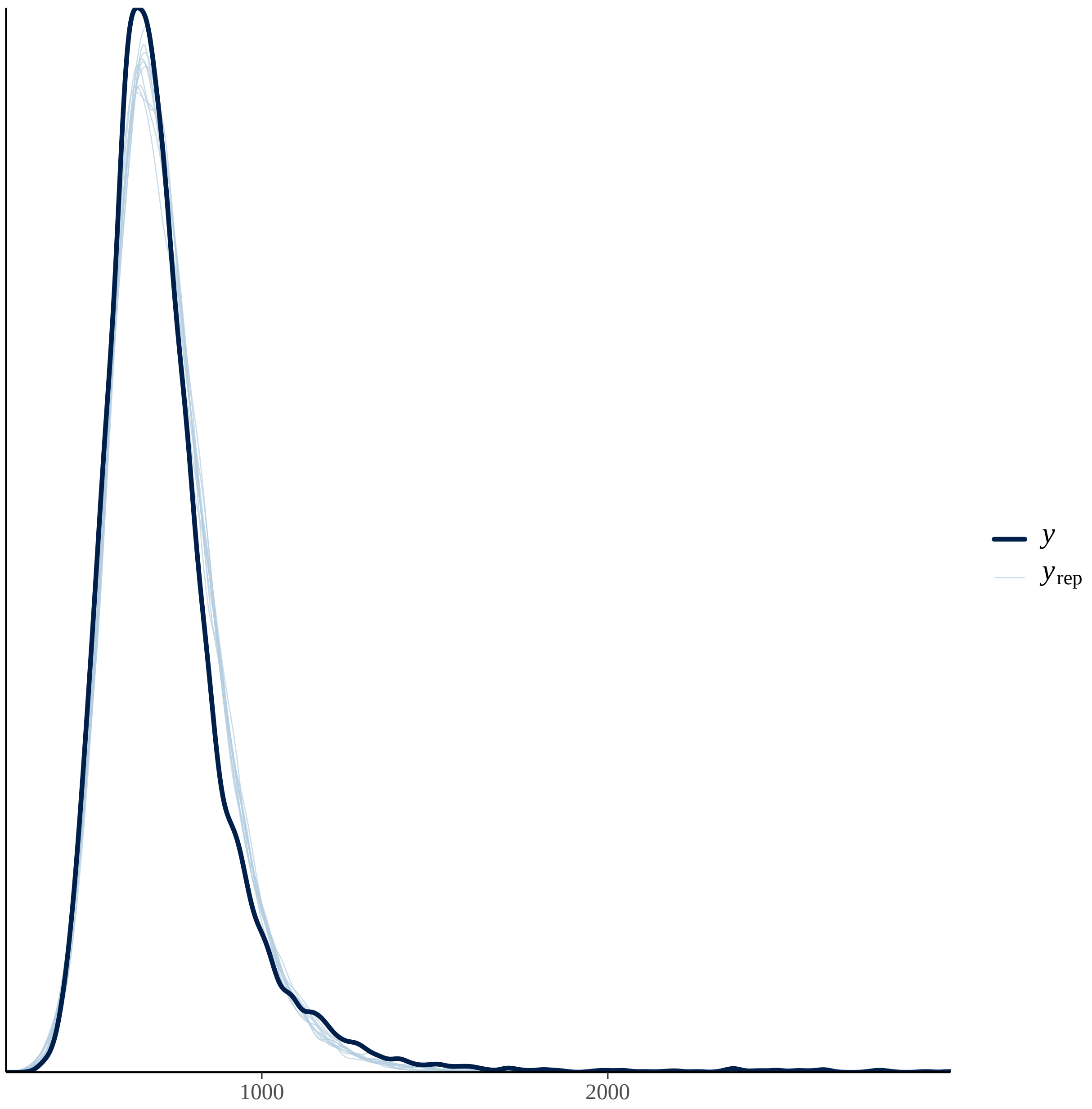

ex-Gaussian PPC

```
## Bayes Factors for Model Comparison
##
##     Model                                          BF
## [1] 0 + Intercept + mode + (1 + mode | s) 8.40e+21
## [2] 0 + Intercept + mode + (1 + mode | s) 0.00e+00
##
## * Against Denominator: [3] 0 + Intercept + mode + (1 + mode | s)
## *   Bayes Factor Type: marginal likelihoods (bridgesampling)
```

For BF model comparison, we define the ex-Gaussian model as M_0, the shifted log-normal model as M_1 and the Gaussian model as M_2. The BF_{10} » 10, indicating strong evidence favoring M_1 (shifted log-normal) over M_0 (ex-Gaussian). In contrast, BF_{20} was BF « 1/10, indicating that M_2 (Gaussian) is a substantially worse fit than M_0.

```
## Recompiling the model with 'rstan'
## Recompilation done
## No problematic observations found.  Returning the original 'loo' object.
## No problematic observations found.  Returning the original 'loo' object.
## No problematic observations found.  Returning the original 'loo' object.

##                        elpd_diff se_diff
## Stroop_reapro_log          0.0       0.0
## Stroop_reapro_exgauss    -36.4      58.3
## Stroop_reapro_gauss    -4079.3     226.9
```

For Loo comparison however, the elpd difference between M_0 (ex-Gaussian) and M_1 (shifted log-normal) was not bigger than twice its standard deviation. This indicates that the numerical difference favoring M_1 over M_0 is not meaningful. However, both M_0 and M_1 have a much higher predictive accuracy than M_2 (Gaussian).

4.9 Stroop Single Model Output

Based on the Reviewers' request, we also ran a single model of the 2018/2020 Stroop data that includes all analyzed predictor levels, and then subsequently extracted relevant contrasts via posterior linear combinations.

4.9.1 2018 [LW/IS]PC Single Model Output

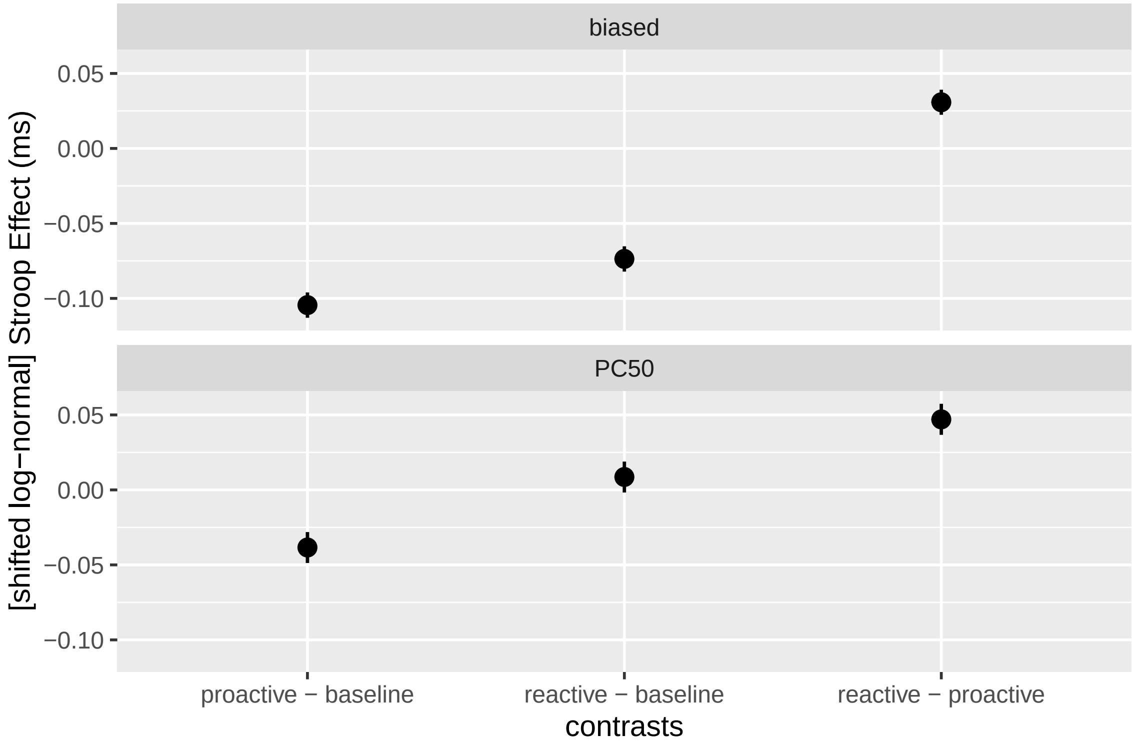

Table 2: PC-Effect Contrasts

Term	Estimate	HDI	pd
proactive - baseline biased	-0.1045	[-0.11, -0.1]	100%
proactive - baseline PC50	-0.0384	[-0.05, -0.03]	100%
reactive - baseline biased	-0.0737	[-0.08, -0.07]	100%
reactive - baseline PC50	0.0086	[0, 0.02]	94.85%
reactive - proactive biased	0.0308	[0.02, 0.04]	100%
reactive - proactive PC50	0.0470	[0.04, 0.06]	100%

Note. Each of these terms reflect the contrasting effects of mode. The key effects for the LWPC effect are ’proactive - baseline’ for both biased and PC-50 items, and the key effects for the ISPC effect are ’reactive - baseline’ for biased but not PC-50 items. This refers to the difference in the Stroop effect between Proactive/Reactive and Baseline for specific item types.

4.9.2 2020 [LW/IS]PC Single Model Output

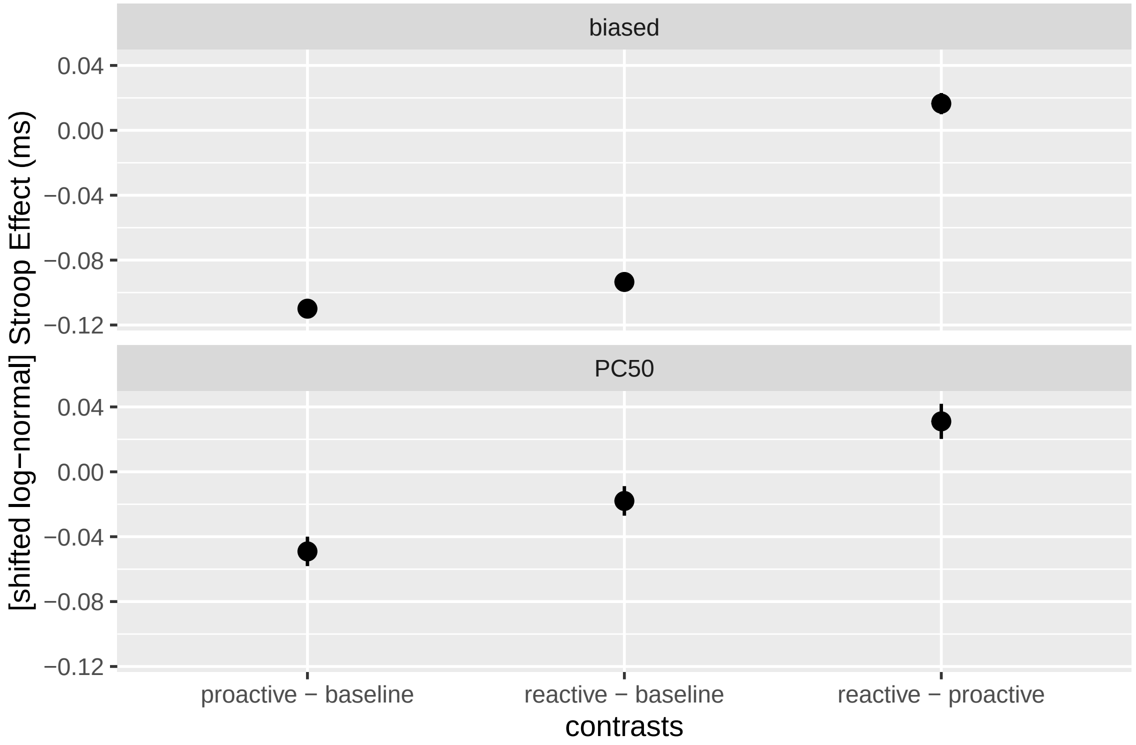

Table 3: PC-Effect Contrasts

Term	Estimate	HDI	pd
proactive - baseline biased	-0.1099	[-0.12, -0.1]	100%
proactive - baseline PC50	-0.0491	[-0.06, -0.04]	100%
reactive - baseline biased	-0.0935	[-0.1, -0.09]	100%
reactive - baseline PC50	-0.0180	[-0.03, -0.01]	99.99%
reactive - proactive biased	0.0165	[0.01, 0.02]	100%
reactive - proactive PC50	0.0311	[0.02, 0.04]	100%

Note. Each of these terms reflect the contrasting effects of mode. The key effects for the LWPC effect are 'proactive - baseline' for both biased and PC-50 items, and the key effects for the ISPC effect are 'reactive - baseline' for biased but not PC-50 items. This refers to the difference in the Stroop effect between Proactive/Reactive and Baseline for specific item types.

4.9.3 2018 Transfer Cost Single Model Output

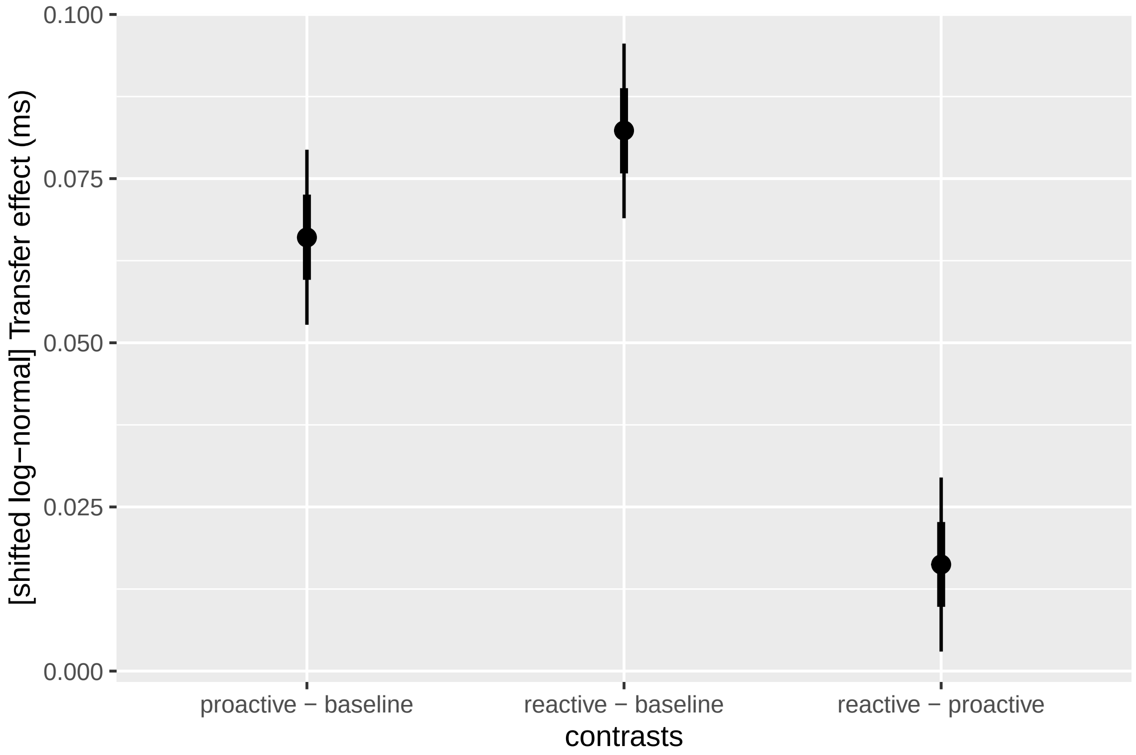

Table 3: Transfer Cost Contrasts

Term	Estimate	HDI	pd
proactive - baseline	0.0660	[0.05, 0.08]	100%
reactive - baseline	0.0823	[0.07, 0.1]	100%
reactive - proactive	0.0162	[0, 0.03]	99.16%

Note. Each of these terms reflect the contrasting effects of mode. The key effects are 'reactive - proactive', which refer to the difference in the transfer cost between the Reactive and Proactive modes.

4.9.4 2020 Transfer Cost Single Model Output

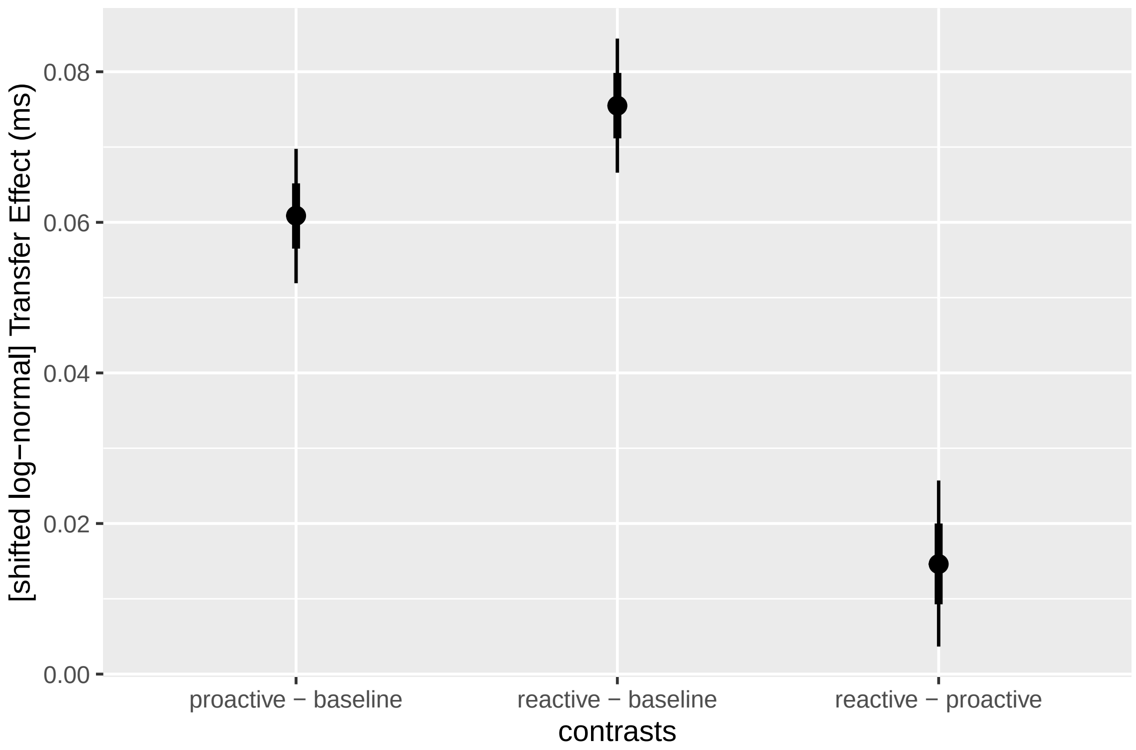

Table 4: Transfer Cost Contrasts

Term	Estimate	HDI	pd
proactive - baseline	0.0609	[0.05, 0.07]	100%
reactive - baseline	0.0755	[0.07, 0.08]	100%
reactive - proactive	0.0146	[0, 0.03]	99.54%

Note. Each of these terms reflect the contrasting effects of mode. The key effects are 'reactive - proactive', which refer to the difference in the Transfer Cost between the Reactive and Proactive modes.

4.9.5 2018 Congruency Cost Single Model Output

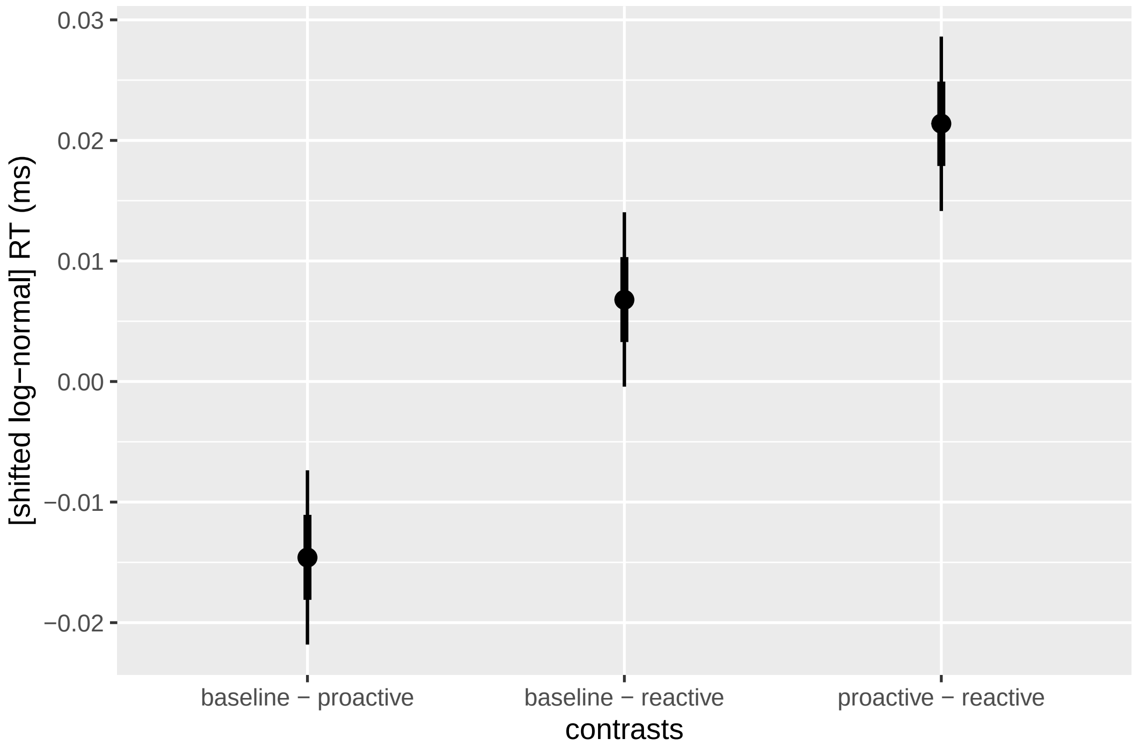

Table 4: Congruency Cost Contrasts

Term	Estimate	HDI	pd
baseline - proactive	-0.0146	[-0.02, -0.01]	100%
baseline - reactive	0.0068	[0, 0.01]	96.74%
proactive - reactive	0.0214	[0.01, 0.03]	100%

Note. Each of these terms reflect the contrasting effects of mode. The key terms are 'proactive - reactive', which refers to the difference in the shifted log-normal RT to make a correct response for diagnostic congruent trials between the Proactive and Reactive modes.

4.9.6 2020 Congruency Cost Single Model Output

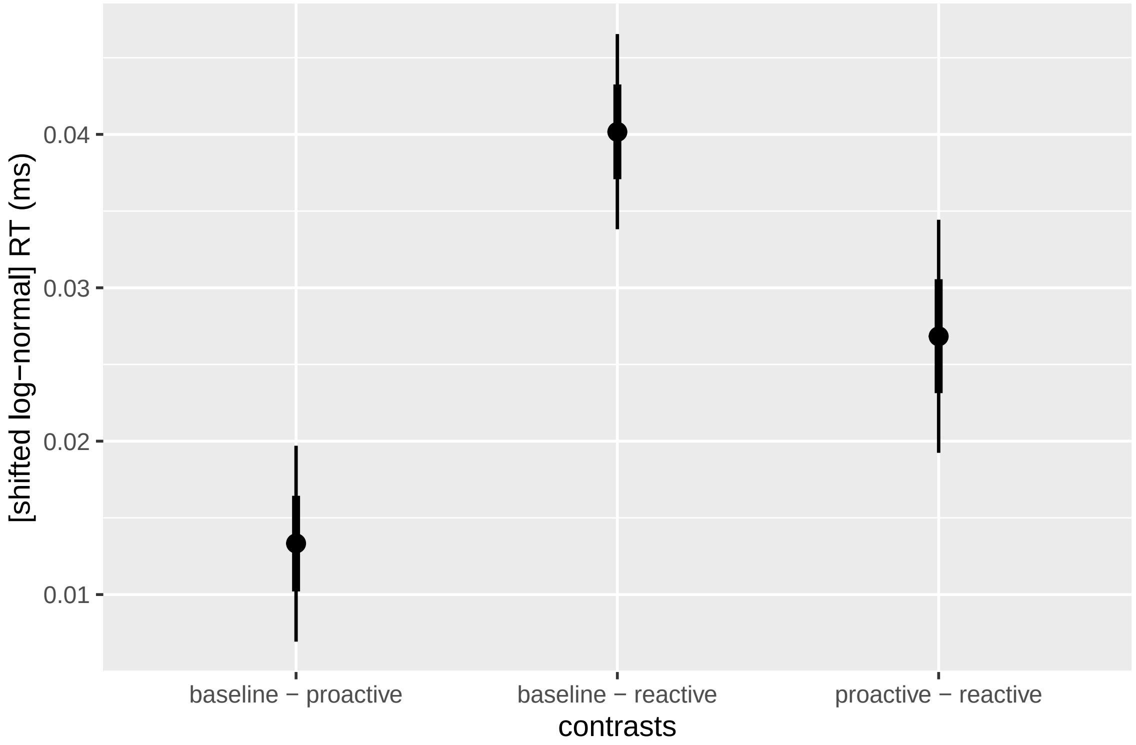

Table 5: Congruency Cost Contrasts

Term	Estimate	HDI	pd
baseline - proactive	0.0133	[0.01, 0.02]	100%
baseline - reactive	0.0402	[0.03, 0.05]	100%
proactive - reactive	0.0268	[0.02, 0.03]	100%

Note. Each of these terms reflect the contrasting effects of mode. The key terms are 'proactive - reactive', which refers to the difference in the [shifted log-normal] RT to make a correct response for diagnostic congruent trials between the Proactive and Reactive modes.

4.10 Stroop Results with Multimodality (MM)

To check the importance of this criterion, these LWPC/ISPC/Transfer/Congruency Cost analyses remove the unimodality assumption, given the disproportionate number of DMCC Stroop participants that were removed because of this criterion.

4.10.1 2018 PC-Effects with MM

Table 1: PC-Effect Contrasts with MM

Term	Estimate	HDI	pd
proactive - baseline biased	-0.1051	[-0.11, -0.1]	100%
proactive - baseline PC50	-0.0398	[-0.05, -0.03]	100%
reactive - baseline biased	-0.0771	[-0.09, -0.07]	100%
reactive - baseline PC50	0.0095	[0, 0.02]	96%
reactive - proactive biased	0.0280	[0.02, 0.04]	100%
reactive - proactive PC50	0.0494	[0.04, 0.06]	100%

Note. Each of these terms reflect the contrasting effects of mode. The key effects for the LWPC effect are 'proactive - baseline' for both biased and PC-50 items, and the key effects for the ISPC effect are 'reactive - baseline' for biased but not PC-50 items. This refers to the difference in the Stroop effect between Proactive/Reactive and Baseline for specific items

4.10.4 2020 PC-Effects with MM

Table 1: PC-Effect Contrasts with MM

Term	Estimate	HDI	pd
proactive - baseline biased	-0.0977	[-0.11, -0.09]	100%
proactive - baseline PC50	-0.0360	[-0.05, -0.02]	100%
reactive - baseline biased	-0.0879	[-0.1, -0.08]	100%
reactive - baseline PC50	-0.0070	[-0.02, 0.01]	83.09%
reactive - proactive biased	0.0097	[0, 0.02]	95.03%
reactive - proactive PC50	0.0290	[0.01, 0.04]	100%

Note. Each of these terms reflect the contrasting effects of mode. The key effects for the LWPC effect are 'proactive - baseline' for both biased and PC-50 items, and the key effects for the ISPC effect are 'reactive - baseline' for biased but not PC-50 items. This refers to the difference in the Stroop effect between Proactive/Reactive and Baseline for specific item types

4.10.2 2018 Transfer Cost with MM

Table 2: Transfer Cost Contrasts

Term	Estimate	HDI	pd
proactive - baseline	0.0652	[0.05, 0.08]	100%
reactive - baseline	0.0866	[0.07, 0.1]	100%
reactive - proactive	0.0214	[0.01, 0.04]	99.87%

Note. Each of these terms reflect the contrasting effects of mode. The key effects are 'reactive - proactive', which refer to the difference in the Transfer Cost between the Reactive and Proactive modes.

4.10.2 2020 Transfer Cost with MM

Table 2: Transfer Cost (Reactive vs Proactive)

Term	Estimate	HDI	pd
proactive - baseline	0.0616	[0.04, 0.08]	100%
reactive - baseline	0.0809	[0.06, 0.1]	100%
reactive - proactive	0.0192	[0, 0.04]	97.96%

Note. Each of these terms reflect the contrasting effects of mode. The key effects are 'reactive - proactive', which refer to the difference in the transfer cost between the Reactive and Proactive modes.

4.10.3 2018 Congruency Cost with MM

Table 3: Congruency Cost Contrast assuming shifted log-normal distribution

Term	Estimate	HDI	pd
proactive - reactive	0.0249	[0, 0.05]	98.95%

Note. Each of these terms reflect the contrasting effects of mode. The key terms are 'proactive - reactive', which refers to the difference in the RT to make a correct response for diagnostic congruent trials between the Proactive and Reactive modes.

Table 4: Congruency Cost Contrast assuming Gaussian distribution

Term	Estimate	HDI	pd
proactive - reactive	7.4974	[-3.82, 17.89]	91.13%

Note. Each of these terms reflect the contrasting effects of mode. The key terms are 'proactive - reactive', which refers to the difference in the RT to make a correct response for diagnostic congruent trials between the Proactive and Reactive modes.

Table 5: Congruency Cost Contrast assuming ex-Gaussian distribution

Term	Estimate	HDI	pd
proactive - reactive	9.9755	[4.15, 16.02]	99.95%

Note. Each of these terms reflect the contrasting effects of mode. The key terms are 'proactive - reactive', which refers to the difference in the RT to make a correct response for diagnostic congruent trials between the Proactive and Reactive modes.

4.10.6 2020 Congruency Cost with MM

Table 3: Congruency Cost (Proactive vs Reactive) assuming shifted log-normal distribution

Term	Estimate	HDI	pd
proactive - reactive	0.0257	[0.01, 0.04]	99.82%

Note. Each of these terms reflect the contrasting effects of mode. The key terms are 'proactive - reactive', which refers to the difference in the RT to make a correct response for diagnostic congruent trials between the Proactive and Reactive modes.

Table 4: Congruency Cost (Proactive vs Reactive) assuming Gaussian distribution

Term	Estimate	HDI	pd
proactive - reactive	7.0536	[-12.66, 28.57]	75.5%

Note. Each of these terms reflect the contrasting effects of mode. The key terms are 'proactive - reactive', which refers to the difference in the RT to make a correct response for diagnostic congruent trials between the Proactive and Reactive modes.

Table 5: Congruency Cost (Proactive vs Reactive) assuming ex-Gaussian distribution

Term	Estimate	HDI	pd
proactive - reactive	8.3704	[-0.36, 17.26]	96.79%

Note. Each of these terms reflect the contrasting effects of mode. The key terms are 'proactive - reactive', which refers to the difference in the RT to make a correct response for diagnostic congruent trials between the Proactive and Reactive modes.

5 Cued-TS

While the below sections will go into more detail for each Cued-TS indicator, we briefly describe the expected results for differences in performance across conditions.

RT performance on non-incentivized [biased] congruent trials: We predict that subjects will be 1) faster in the Proactive versus Baseline mode, and 2) faster in the Reactive versus Baseline mode.

TRCE Error: We predict that subjects will have 1) a reduction in the TRCE Error in the Reactive versus Baseline mode 2) a reduction in the TRCE Error in the Reactive versus Proactive mode.

TRCE RT: We predict that subjects will have 1) an increase in the TRCE RT in the Reactive versus Baseline mode. 2) an increase in the TRCE RT in the Reactive versus Proactive mode.

5.1 Non-Incentivized Congruent RT 2018

The Non-incentivized Congruent RT Effect was examined as the [shifted log-normal] RT to make a correct response for non-incentivized congruent trials. Mode was dummy coded to compare conditions directly. Only non-incentivized and congruent trials were included to not only make direct comparisons between the Baseline and Proactive mode, but also to show a double dissociation between the Proactive and Reactive mode.

$$RT = mode + \left(1 + mode \mid ID\right), \text{family}\left(\text{shifted lognormal}\right)$$

$$\begin{gathered}
\text{RT}_{i,t} \sim \text{shifted log-normal}\left(\mu_{i,t}, \sigma, \theta\right) \\
\log\left(\mu_{i,t} + \theta\right) = \beta_{0i} + \beta_{1i} * mode_{i,t} \\
\begin{bmatrix} \beta_{0i} \\ \beta_{1i} \end{bmatrix} \sim \text{N}\left(\begin{bmatrix} \beta_0 \\ \beta_1 \end{bmatrix}, \Sigma\right) \\
\beta_0, \beta_1 \sim \text{flat}, \Sigma \sim \text{LKJ}(1), \sigma \sim \text{student-t}(3,0,\ 2.5), \theta \sim \text{uniform}(0,\ \text{minRT})
\end{gathered}$$

5.1.1 Proactive - Baseline Non-Inc Congruent RT Effect

Table 1: Baseline versus Proactive for non-incentivized congruent trials

Term	Estimate	SE	HDI	pd
Baseline Congruent	6.3401	0.0294	[6.28, 6.4]	100%
Proactive Congruent	-0.3082	0.0131	[-0.33, -0.28]	100%

Note. The intercept term 'Baseline Congruent' refers to the average [shifted log-normal] RT to make a correct response for Baseline congruent trials. 'Proactive Congruent' is the key e˙ect and refers to the difference in RT performance across the Proactive and Baseline modes.

There was decisive evidence for a greater Non-Inc Congruent RT Effect in the Proactive versus Baseline mode (β = -0.31, se = 0.01, HDI = [-0.33, -0.28], pd = 100%).

5.1.2 Proactive - Reactive Non-Inc Congruent RT Effect

Table 2: Reactive versus Proactive for non-incentivized congruent trials

Term	Estimate	SE	HDI	pd
Reactive Congruent	6.4522	0.0300	[6.4, 6.51]	100%
Proactive Congruent	-0.4256	0.0169	[-0.46, -0.39]	100%

Note. The intercept term 'Reactive Congruent' refers to the average [shifted log-normal] RT to make a correct response for Baseline congruent trials. 'Proactive Congruent' is the key effect and refers to the difference in RT performance across the Proactive and Reactive modes.

There was also decisive evidence for a greater Non-Inc Congruent RT Effect in the Proactive versus Reactive mode (β = -0.43, se = 0.02, HDI = [-0.46, -0.39], pd = 100%).

5.2 Non-Incentivized Congruent RT 2020

These analyses test for a consistent pattern in the 2020 dataset, using the 2018 estimates as informative priors.

5.2.1 Proactive - Baseline Non-Inc Congruent RT Effect

Table 1: Baseline versus Proactive for non-incentivized congruent trials

Term	Estimate	SE	HDI	pd
Baseline Congruent	6.3969	0.0219	[6.35, 6.44]	100%
Proactive Congruent	-0.3283	0.0102	[-0.35, -0.31]	100%

Note. The intercept term 'Baseline Congruent' refers to the average [shifted log-normal] RT to make a correct response for baseline congruent trials. 'Proactive Congruent' is the key effect and refers to the difference in RT performance between the proactive and baseline mode.

There is decisive evidence that the Non-Inc Congruent RT Effect was greater in the Proactive versus Baseline mode (β = -0.33, se = 0.01, HDI = [-0.35, -0.31], pd = 100%).

Table 2: Hypothesis Test for Pro-Bas non-inc cong RT

Hypothesis	Estimate	SE	HDI	SDR	Post.Prob
Proactive - Baseline non-inc cong RT	-0.02	0.01	[-0.35, -0.31]	0.20	0.00

Note. The key term in this table is the SDR score, which is computed as the ratio of the posterior distribution to the prior distribution at a specific point, in this case the mean of the prior distribution to investigate whether there is an increased likelihood of this value as a function of incoming data.

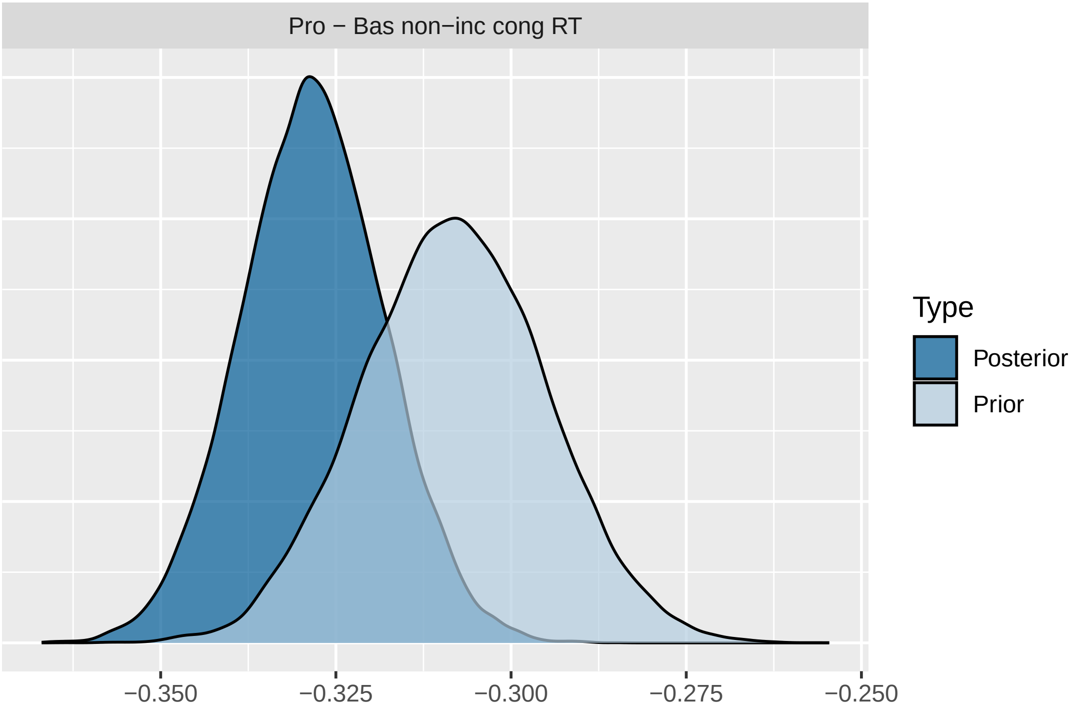

The plot indicates that the posterior is shifted away from the prior. The graph is centered at the mean value of 2018 estimate, and indicates the SDR to be less than one (0.2) at that point. This finding suggests that the prior underestimates the difference in the non-Inc congruent RT e˙ect between the Proactive and Baseline modes. More generally the pattern across the 2018 and 2020 datasets replicates that reported in Tang et al (2023).

5.2.2 Proactive - Reactive Non-Inc Congruent RT Effect

Table 3: Proactive versus Reactive for non-inc cong trials

Term	Estimate	SE	HDI	pd
Reactive Congruent	6.4636	0.0221	[6.42, 6.51]	100%
Proactive Congruent	-0.3684	0.0137	[-0.4, -0.34]	100%

Note. The intercept term 'Reactive Congruent' refers to the average [shifted log-normal] RT to make a correct response for baseline congruent trials. 'Proactive Congruent' is the key effect and refers to the difference in RT performance between the proactive and reactive mode.

There is decisive evidence that the Non-Inc Congruent RT Effect was greater in the Proactive versus Reactive mode (β = -0.37, se = 0.01, HDI = [-0.4, -0.34], pd = 100%).

Table 4: Hypothesis Test for Pro-Rea non-inc congruent trials

Hypothesis	Estimate	SE	HDI	SDR	Post.Prob
Proactive - Reactive non-inc cong RT	0.06	0.01	[-0.4, -0.34]	0.00	0.00

Note. The key term in this table is the SDR score, which is computed as the ratio of the posterior distribution to the prior distribution at a specific point, in this case the mean of the prior distribution to investigate whether there is an increased likelihood of this value as a function of incoming data.

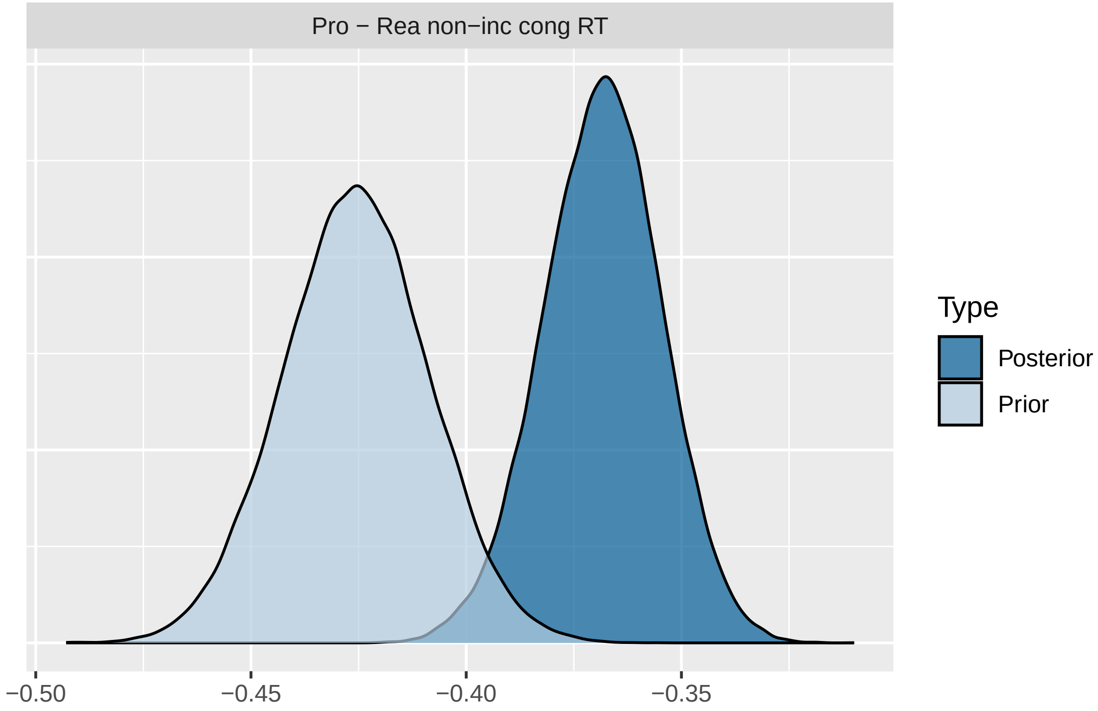

The plot indicates that the posterior is shifted away from the prior. The graph is centered at the mean value of 2018 estimate, and indicates the SDR to be less than one (0) at that point. This suggests that the prior overestimates the di˙erence between the non-Inc congruent RT effect.

5.3 TRCE Error 2018

The task-rule congruency effect (TRCE) is defined as the increase in both errors and RT on incongruent trials (the two task rules are associated with different responses to the target stimulus) relative to congruent trials (the two task rules are associated with the same response to the target stimulus).

TRCE Error was examined as the log odds to make an error on incongruent trials relative to congruent ones. Non-incentivized [biased] trials were included to directly compare across modes, since these are well-matched. TRCE Error was predicted to be higher in Reactive relative to Baseline and Proactive. Mode and con.id are dummy coded to compute the difference in the TRCE across sessions. For models adopting a maximal random effect structure, the intercept, con.id and mode terms were all nested within subject ID. However the results within the main Results section only included the intercept and con.id as random effects, to more directly match the logistic HBR models with the error rate ones and illustrate the specific advantages of logistic regression.

Wilkinson Notation

$$Correct = mode\ x\ con.id\ + \left(1 + con.id * mode \mid ID\right),\ \text{family}\left(\text{binomial}\right)$$

Fully Indexed Notation

$$\begin{gathered}
\text{Correct}_{i,t} \sim \text{Bernoulli}\left(p_{i,t}\right) \\
\text{logit}\left(p_{i,t}\right) = \beta_{0i} + \beta_{1i} * mode_{i,t}\ x\ \beta_{2i} * con.id_{i,t} \\
\begin{bmatrix} \beta_{0i} \\ \beta_{1i} \\ \beta_{2i} \end{bmatrix} \sim \text{N}\left(\begin{bmatrix} \beta_0 \\ \beta_1 \\ \beta_2 \end{bmatrix}, \Sigma \right) \\
\beta_0, \beta_1, \beta_2 \sim \text{flat}, \Sigma \sim \text{LKJ}(1)
\end{gathered}$$

5.3.1 Reactive - Baseline TRCE Error Logistic [with Max Random Effect Structure]

Table 1: Reactive - Baseline TRCE Error (null)

Term	Estimate	SE	HDI	pd
Baseline Congruent	-3.8391	0.1035	[-4.04, -3.64]	100%
Baseline Incongruent	1.4504	0.1042	[1.25, 1.66]	100%
Reactive Congruent	-0.8125	0.0901	[-0.99, -0.63]	100%

Note. The intercept term 'Baseline Congruent' refers to the log odds to make an error for Baseline congruent trials.

Table 2: Reactive - Baseline TRCE Error (full)

Term	Estimate	SE	HDI	pd
Baseline Congruent	-3.7782	0.1057	[-3.98, -3.56]	100%
Baseline Incongruent	1.3244	0.1102	[1.11, 1.54]	100%
Reactive Congruent	-1.0246	0.1138	[-1.27, -0.82]	100%
Reactive x Incongruent	0.3959	0.1483	[0.12, 0.7]	99.42%

Note. The intercept term 'Baseline Congruent' refers to the log odds to make an error for Baseline congruent trials. 'Reactive x Incongruent' is the key effect and refers to the difference in TRCE Error across the Reactive and Baseline modes.

There was strong evidence for a greater TRCE Error in the Reactive versus Baseline mode (β = 0.4, se = 0.15, HDI = [0.12, 0.7], pd = 99.42%), while assuming a maximal random effect structure. In contrast, this effect was numerically less in Tang et al. but not significant (2023).

5.3.2 Reactive - Proactive TRCE Error Logistic [with Max Random Effect Structure]

Table 3: Reactive - Proactive TRCE Error (null)

Term	Estimate	SE	HDI	pd
Proactive Congruent	-3.4937	0.0870	[-3.69, -3.34]	100%
Reactive Incongruent	1.5237	0.1041	[1.32, 1.73]	100%
Reactive Congruent	-1.0783	0.0515	[-1.18, -0.98]	100%

Note. The intercept term 'Proactive Congruent' refers to the average log odds to make an error for Proactive congruent trials.

Table 4: Reactive - Proactive TRCE Error (full)

Term	Estimate	SE	HDI	pd
Proactive Congruent	-3.4625	0.0852	[-3.63, -3.3]	100%
Proactive Incongruent	1.4721	0.1114	[1.26, 1.69]	100%
Reactive Congruent	-1.3356	0.1100	[-1.56, -1.13]	100%
Reactive x Incongruent	0.2123	0.1396	[-0.06, 0.49]	93.4%

Note. The intercept term 'Proactive Congruent' refers to the average log odds to make an error for Proactive congruent trials. 'Reactive x Incongruent' is the key effect and refers to the difference in TRCE Error across the Reactive and Proactive modes.

There was little evidence for a difference in the TRCE Error between the Proactive and Reactive modes (β = 0.21, se = 0.14, HDI = [-0.06, 0.49], pd = 93.4%), while assuming a maximal random effect structure. This contrasts with the pattern observed in Tang et al (2023), in which a reduced TRCE Error was found for Reactive.

5.3.3 Reactive - Baseline TRCE Error Logistic

Table 5: Reactive - Baseline TRCE Error (null)

Term	Estimate	SE	HDI	pd
Baseline Congruent	-3.8100	0.0949	[-3.99, -3.62]	100%
Baseline Incongruent	1.4451	0.1002	[1.25, 1.63]	100%
Reactive Congruent	-0.7783	0.0548	[-0.88, -0.67]	100%

Note. The intercept term 'Baseline Congruent' refers to the log odds to make an error for Baseline congruent trials.

Table 6: Reactive - Baseline TRCE Error (full)

Term	Estimate	SE	HDI	pd
Baseline Congruent	-3.7711	0.1000	[-3.97, -3.58]	100%
Baseline Incongruent	1.3282	0.1094	[1.12, 1.55]	100%
Reactive Congruent	-0.9263	0.0726	[-1.07, -0.79]	100%
Reactive x Incongruent	0.3667	0.1122	[0.15, 0.58]	99.98%

Note. The intercept term 'Baseline Congruent' refers to the log odds to make an error for Baseline congruent trials. 'Reactive x Incongruent' is the key effect and refers to the difference in TRCE Error across the Reactive and Baseline modes.

There was strong evidence for a greater TRCE Error in the Reactive versus Baseline mode (β = 0.37, se = 0.11, HDI = [0.15, 0.58], pd = 99.98%). In contrast, this effect was numerically less in Tang et al. but not significant (2023).

5.3.4 Reactive - Proactive TRCE Error Logistic

Table 7: Reactive - Proactive TRCE Error (null)

Term	Estimate	SE	HDI	pd
Proactive Congruent	-3.4937	0.0870	[-3.69, -3.34]	100%
Reactive Incongruent	1.5237	0.1041	[1.32, 1.73]	100%
Reactive Congruent	-1.0783	0.0515	[-1.18, -0.98]	100%

Note. The intercept term 'Proactive Congruent' refers to the average log odds to make an error for Proactive congruent trials.

Table 8: Reactive - Proactive TRCE Error (full)

Term	Estimate	SE	HDI	pd
Proactive Congruent	-3.4838	0.0881	[-3.65, -3.32]	100%
Proactive Incongruent	1.4878	0.1076	[1.28, 1.7]	100%
Reactive Congruent	-1.1383	0.0716	[-1.29, -1.01]	100%
Reactive x Incongruent	0.1395	0.1095	[-0.08, 0.35]	89.62%

Note. The intercept term 'Proactive Congruent' refers to the average log odds to make an error for Proactive congruent trials. 'R eactive x Incongruent' is the key effect and refers to the di˙erence in TRCE Error across the Reactive and Proactive modes.

There was little evidence for a difference in the TRCE Error between the Proactive and Reactive modes (β = 0.14, se = 0.11, HDI = [-0.08, 0.35], pd = 89.62%). This contrasts with the pattern observed in Tang et al (2023), in which a reduced TRCE Error was found for Reactive.

5.3.5 Main Reactive and Baseline Logistic

Table 9: Reactive and Baseline Cued-TS Error Main Effect

Term	Estimate	SE	HDI	pd
Intercept	-3.6441	0.0772	[-3.8, -3.49]	100%

Note. The intercept term 'Intercept' refers to the average log odds to make an error for Baseline and Reactive trials.

Table 10: Reactive - Baseline Cued-TS Error Main Effect

Term	Estimate	SE	HDI	pd
Baseline	-3.3366	0.0792	[-3.49, -3.49]	100%
Reactive	-0.7547	0.0538	[-0.86, -3.49]	100%

Note. The intercept term 'Baseline' refers to the average log odds to make an error for Baseline trials. 'Reactive' is the difference between trials across the Reactive and Baseline modes.

There was decisive evidence for a main effect across the Baseline and Reactive modes (β = -0.75, se = 0.05, HDI = [-0.86, -3.49], pd = 100%). This result shows the Reactive task manipulation reduced the log odds to make an error on biased trials.

5.3.6 Main Reactive and Proactive Effect Logistic

Table 11: Reactive and Proactive Cued-TS Error

Term	Estimate	SE	HDI	pd
Intercept	-3.3695	0.0632	[-3.5, -3.25]	100%

Note. The intercept term 'Intercept' refers to the average log odds to make an error for Proactive and Reactive trials.

Table 12: Reactive - Proactive Cued-TS Error Main Effect

Term	Estimate	SE	HDI	pd
Proactive	-2.9721	0.0702	[-3.11, -2.83]	100%
Reactive	-1.0350	0.0516	[-1.14, -0.94]	100%

Note. The intercept term 'Intercept' refers to the average log odds to make an error for Proactive and Reactive trials.

There was decisive evidence for a main effect across the Proactive and Reactive modes (β = -1.03, se = 0.05, HDI = [-1.14, -0.94], pd = 100%). This result further supports the Reactive task manipulation reduces the log odds to make an error on biased trials.

5.3.7 Incongruent Reactive and Baseline Logistic

Table 13: Reactive and Baseline Incongruent Cued-TS Error Main Effect

Term	Estimate	SE	HDI	pd
Incongruent	-2.6906	0.0877	[-2.86, -2.52]	100%

Note. The intercept term 'Incongruent' refers to the average log odds to make an error for Baseline and Reactive Incongruent trials.

Table 14: Reactive - Baseline Incongruent Cued-TS Error Main Effect

Term	Estimate	SE	HDI	pd
Baseline Incongruent	-2.4384	0.0952	[-2.63, -2.25]	100%
Reactive Incongruent	-0.5621	0.0850	[-0.73, -0.39]	100%

Note. The intercept term 'Baseline Incongruent' refers to the average log odds to make an error for Baseline Incongruent trials. 'R eactive Incongruent' is the difference between incongruent trials across the Reactive and Baseline modes.

There was decisive evidence for a main effect of mode across the Baseline and Reactive modes (β = -0.56, se = 0.09, HDI = [-.73, -.39], pd = 100%). This result more specifically shows the Reactive task manipulation reduces the log odds to make an error on biased incongruent trials.

5.3.8 Incongruent Reactive and Proactive Logistic

Table 15: Reactive and Proactive Cued-TS Error Incongruent Effect

Term	Estimate	SE	HDI	pd
Intercept	-2.3871	0.0863	[-2.56, -2.22]	100%

Note. The intercept term 'Incongruent' refers to the average log odds to make an error for Reactive and Proactive Incongruent trials.

Table 16: Reactive - Proactive Cued-TS Error Incongruent Effect

Term	Estimate	SE	HDI	pd
Proactive Incongruent	-1.9906	0.0898	[-2.18, -1.82]	100%
Reactive Incongruent	-0.9984	0.0774	[-1.15, -0.85]	100%

Note. The intercept term 'Proactive Incongruent' refers to the average log odds to make an error for Proactive Incongruent trials. 'R eactive Incongruent' is the difference between incongruent trials across the Reactive and Proactive modes.

There was decisive evidence of mode across the Proactive and Reactive modes (β = -1, se = 0.08, HDI = [-1.15, -0.85], pd = 100%). This result more specificall y shows the Reactive task manipulation reduces the log odds to make an error on biased incongruent trials.

5.3.9 Marginal Means Reactive and Baseline Effect Logistic

Table 17: Cued-TS Log odds Error (Baseline vs Reactive)

Term	Estimate	SE	HDI	pd
Baseline Congruent	-3.7696	0.0989	[-3.96, -3.57]	100%
Baseline Incongruent	-2.4427	0.0966	[-2.64, -2.26]	100%
Reactive Congruent	-4.6967	0.1107	[-4.91, -4.48]	100%
Reactive x Incongruent	-3.0057	0.1065	[-3.22, -2.8]	100%

Note. The marginal means for each con.id and mode. The intercept was removed for this analysis

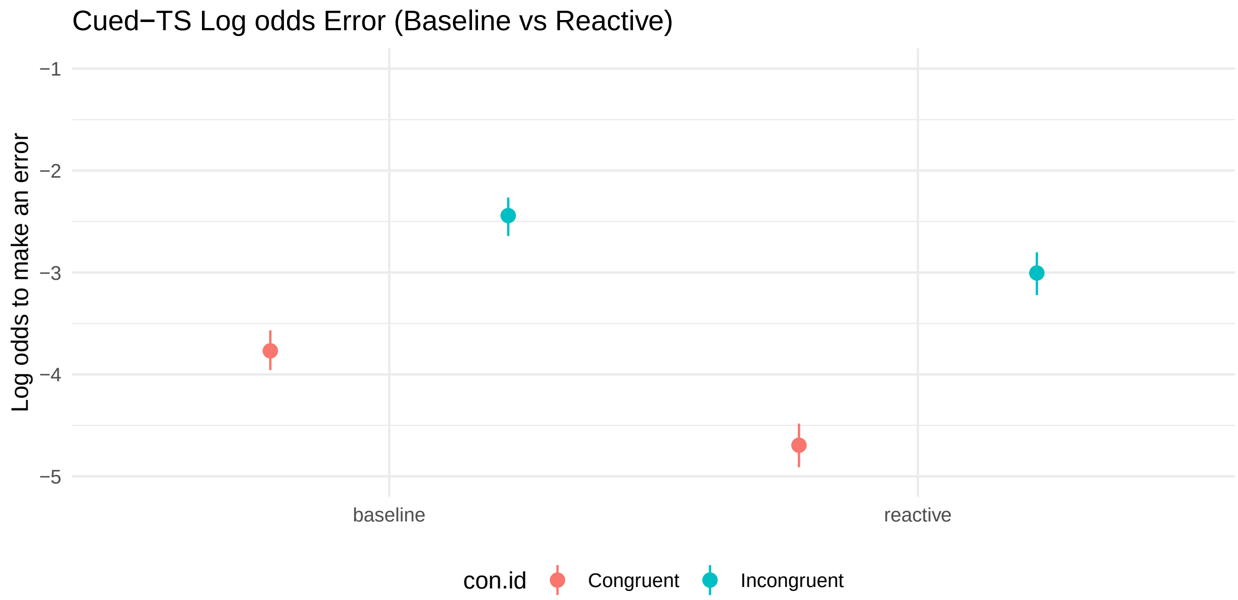

5.3.10 Marginal Means Reactive and Proactive effect Logistic

Table 18: Cued-TS Log odds Error (Reactive vs Proactive)

Term	Estimate	SE	HDI	pd
Proactive Congruent	-3.4817	0.0857	[-3.65, -3.32]	100%
Proactive Incongruent	-1.9921	0.0917	[-2.18, -1.81]	100%
Reactive Congruent	-4.6197	0.0985	[-4.81, -4.42]	100%
Reactive x Incongruent	-2.9904	0.1043	[-3.2, -2.79]	100%

Note. The marginal means for each con.id and mode. The intercept was removed for this analysis.

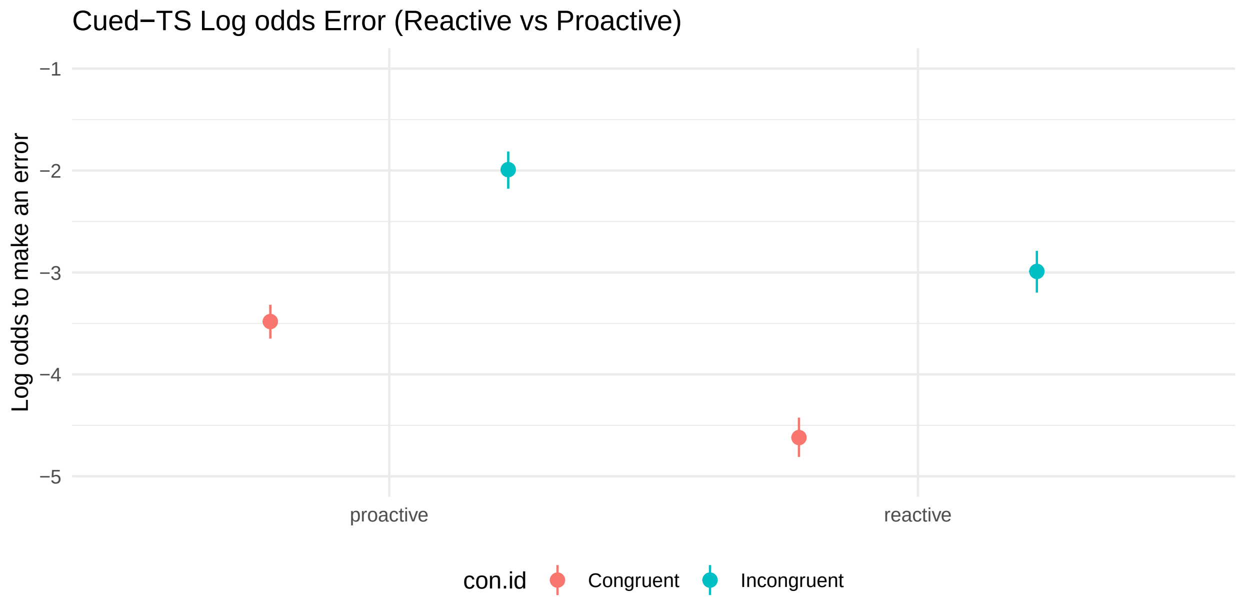

5.3.11 Reactive - Baseline TRCE Error Rate

Table 19: Reactive - Baseline TRCE Error (null)

Term	Estimate	SE	HDI	pd
Baseline Congruent	0.0386	0.0032	[0.03, 0.04]	100%
Baseline Incongruent	0.0579	0.0066	[0.04, 0.07]	100%
Reactive Congruent	-0.0292	0.0035	[-0.04, -0.02]	100%

Note. The intercept term 'Baseline Congruent' refers to the error rate for Baseline congruent trials.

Table 20: Reactive - Baseline TRCE Error (full)

Term	Estimate	SE	HDI	pd
Baseline Congruent	0.0341	0.0036	[0.03, 0.04]	100%
Baseline Incongruent	0.0670	0.0075	[0.05, 0.08]	100%
Reactive Congruent	-0.0201	0.0049	[-0.03, -0.01]	99.99%
Reactive x Incongruent	-0.0182	0.0069	[-0.03, 0]	99.56%

Note. The intercept term 'Baseline Congruent' refers to the error rate for Baseline congruent trials. 'R eactive x Incongruent' is the key TRCE error effect and refers to the difference in TRCE effects across the Reactive and Baseline modes.

There was strong evidence for a reduced TRCE Error in the Reactive versus Baseline mode for error rates (β = -0.02, se = 0.01, HDI = [-0.03, 0], pd = 99.56%). This effect is notably different from what was found in the Reactive - Baseline model using logistic regression.

5.3.12 Reactive - Proactive TRCE Error Rate

Table 21: Reactive - Proactive TRCE Error (null)

Term	Estimate	SE	HDI	pd
Proactive Congruent	0.0553	0.0039	[0.05, 0.06]	100%
Reactive Incongruent	0.0775	0.0075	[0.06, 0.09]	100%
Reactive Congruent	-0.0558	0.0045	[-0.06, -0.05]	100%

Note. The intercept term 'Proactive Congruent' refers to the average log odds to make an error for Proactive congruent trials.

Table 22: Reactive - Proactive TRCE Error (full)

Term	Estimate	SE	HDI	pd
Proactive Congruent	0.0410	0.0043	[0.03, 0.05]	100%
Proactive Incongruent	0.1062	0.0086	[0.09, 0.12]	100%
Reactive Congruent	-0.0270	0.0060	[-0.04, -0.02]	100%
Reactive x Incongruent	-0.0574	0.0084	[-0.07, -0.04]	100%

Note. The intercept term 'Proactive Congruent' refers to the error rate for Proactive congruent trials. 'Reactive x Incongruent' is the key effect and refers to the difference in TRCE Error across the Reactive and Proactive modes.

There was decisive evidence for a reduced TRCE Error in the Reactive mode vesus Proactive mode for error rates (β = -0.06, se = 0.01, HDI = [-0.07, -0.04], pd = 100%). This effect is notably different from the Reactive - Proactive model using logistic regression.

5.3.13 Main Reactive and Baseline Error Rates

Table 23: Reactive and Baseline Cued-TS Error Main Effect

Term	Estimate	SE	HDI	pd
Intercept	0.0530	0.0040	[0.04, 0.06]	100%

Note. The intercept term 'Intercept' refers to the average error rate for Baseline and Reactive trials.

Table 24: Reactive - Baseline Cued-TS Error Main Effect

Term	Estimate	SE	HDI	pd
Baseline	0.0676	0.0049	[0.06, 0.08]	100%
Reactive	-0.0291	0.0054	[-0.04, -0.02]	100%

Note. The intercept term 'Baseline' refers to the average error rate for Baseline trials. 'Reactive' is the difference between trials across the Reactive and Baseline modes.

There was decisive evidence for a main effect of mode in the Reactive versus Baseline mode for error rates (β = -0.03, se = 0.01, HDI = [-0.04, -0.02], pd = 100%). This result shows the Reactive task manipulation reduces error rate on biased trials.

5.3.14 Main Reactive and Proactive Error Rates

Table 25: Reactive and Proactive Cued-TS Error Main Effect

Term	Estimate	SE	HDI	pd
Intercept	0.0664	0.0040	[0.06, 0.07]	100%

Note. The intercept term 'Intercept' refers to the average error rate to make an error for Proactive and Reactive trials.

Table 26: Reactive - Proactive Cued-TS Error Main Effect

Term	Estimate	SE	HDI	pd
Proactive	0.0941	0.0053	[0.08, 0]	100%
Reactive	-0.0557	0.0069	[-0.07, 0]	100%

Note. The intercept term 'Proactive' refers to the average error rate for Proactive trials. 'Reactive' is the difference between trials across the Reactive and Proactive modes.

There was decisive evidence for a main effect of mode in the Reactive versus Proactive mode for error rates (β = -0.06, se = 0.01, HDI = [-0.07, 0], pd = 100%). This result shows the Reactive task manipulation reduces error rate on biased trials.

5.3.15 Incongruent Reactive and Baseline Error Rates

Table 27: Reactive and Baseline Cued-TS Error Incongruent Effect

Term	Estimate	SE	HDI	pd
Incongruent	0.0817	0.0069	[0.07, 0.09]	100%

Note. The intercept term 'Incongruent' refers to the average error rate for Baseline and Reactive Incongruent trials.

Table 28: Reactive - Baseline Cued-TS Error Incongruent Effect

Term	Estimate	SE	HDI	pd
Baseline Incongruent	0.1009	0.0076	[0.09, 0.12]	100%
Reactive Incongruent	-0.0383	0.0068	[-0.05, -0.03]	100%

Note. The intercept term 'Baseline Incongruent' refers to the average error rate for Baseline Incongruent trials. 'Reactive Incongruent' is the difference between incongruent trials across the Reactive and Baseline modes.

There was decisive evidence for an effect of mode across the Baseline and Reactive modes (β = -0.04, se = 0.01, HDI = [-0.05, -0.03], pd = 100%). This result more specifically shows the Reactive task manipulation reduces error rate on biased incongruent trials.

5.3.16 Incongruent Reactive and Proactive Error Rates

Table 29: Reactive and Proactive Cued-TS Error Incongruent Effect

Term	Estimate	SE	HDI	pd
Incongruent	0.1051	0.0072	[0.09, 0.12]	100%

Note. The intercept term 'Incongruent' refers to the average error rate for Proactive and Reactive Incongruent trials.

Table 30: Reactive - Proactive Cued-TS Error Incongruent Effect

Term	Estimate	SE	HDI	pd
Proactive Incongruent	0.1474	0.0084	[0.09, 0.12]	100%
Reactive Incongruent	-0.0846	0.0086	[0.09, 0.12]	100%

Note. The intercept term 'Proactive Incongruent' refers to the average error rate for Proactive Incongruent trials. 'Reactive Incongruent' is the difference between incongruent trials across the Reactive and Proactive modes.

There was decisive evidence for an effect of mode across the Proactive and Reactive modes (β = -0.08, se = 0.01, HDI = [0.09, 0.12], pd = 100%). This result more specifically shows the Reactive task manipulation reduces error rate on biased incongruent trials.

5.3.17 Marginal Means Reactive and Baseline Error Rates

Table 31: Cued-TS Error Rates (Reactive vs Baseline)

Term	Estimate	SE	HDI	pd
Baseline Congruent	0.0341	0.0036	[0.03, 0.04]	100%
Baseline Incongruent	0.1011	0.0073	[0.09, 0.12]	100%
Reactive Congruent	0.0140	0.0036	[0.01, 0.02]	99.99%
Reactive Incongruent	0.0628	0.0073	[0.05, 0.08]	100%

Note. The marginal means for each con.id and mode. The intercept was removed for this analysis

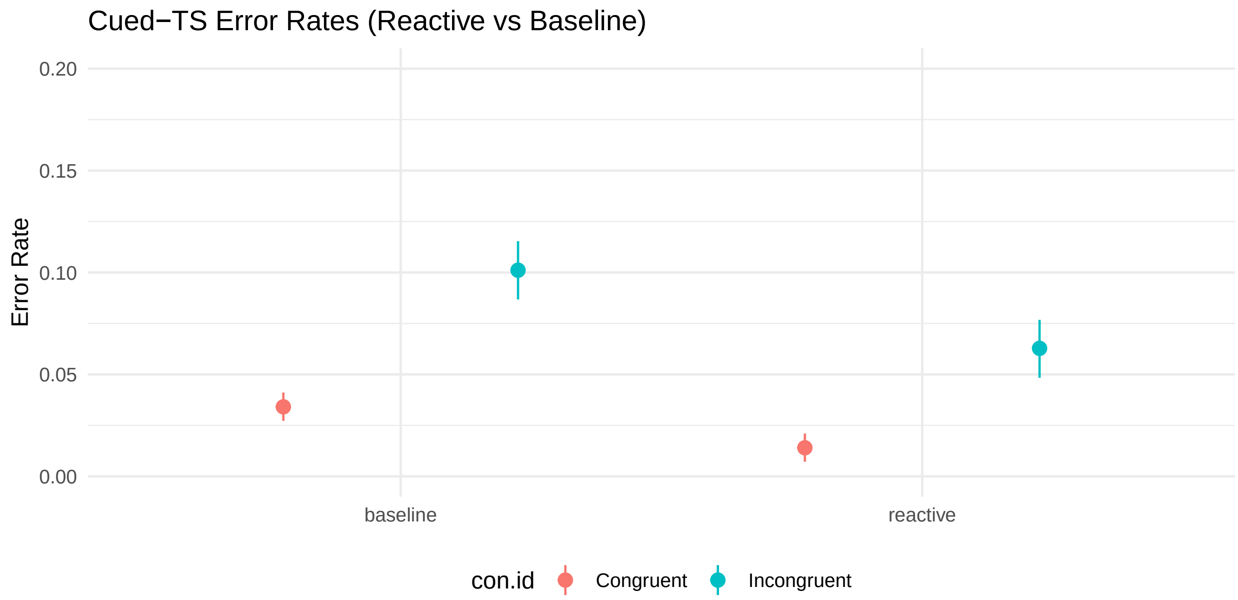

5.3.18 Marginal Means Reactive and Proactive Error Rates

Table 32: Cued-TS Error Rates (Proactive vs Reactive)

Term	Estimate	SE	HDI	pd
Proactive Congruent	0.0410	0.0043	[0.03, 0.05]	100%
Proactive Incongruent	0.1472	0.0078	[0.13, 0.16]	100%
Reactive Congruent	0.0140	0.0043	[0.01, 0.02]	99.94%
Reactive Incongruent	0.0628	0.0078	[0.05, 0.08]	100%

Note. The marginal means for each con.id and mode. The intercept was removed for this analysis

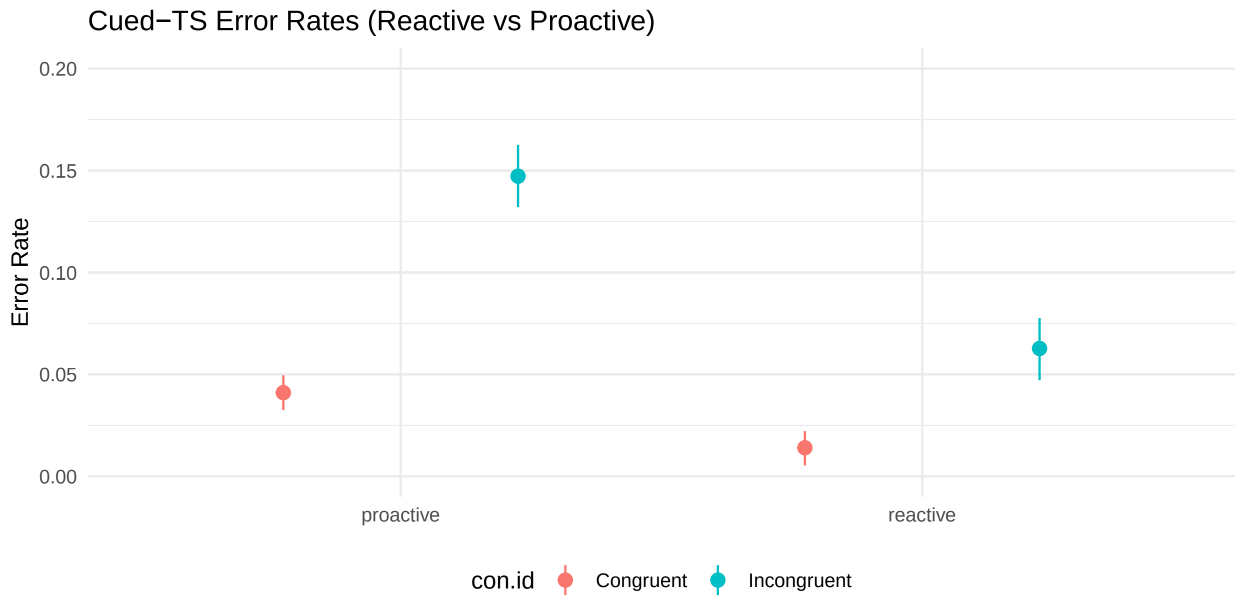

5.4 TRCE Error 2020

These analyses test for a consistent pattern across the 2018 and 2020 datasets, using the 2018 estimates as prior information.

5.4.1 Reactive - Baseline TRCE Error Logistic [with Max Random Effect Structure]

Table 1: Reactive - Baseline TRCE Error (null)

Term	Estimate	SE	HDI	pd
Baseline Congruent	-3.8113	0.0736	[-3.95, -3.66]	100%
Baseline Incongruent	1.4024	0.0739	[1.26, 1.55]	100%
Reactive Congruent	-0.7643	0.0722	[-0.9, -0.62]	100%

Note. The intercept term 'Baseline Congruent' refers to the log odds to make an error for Baseline congruent trials.

Table 2: Reactive - Baseline TRCE Error (full)

Term	Estimate	SE	HDI	pd
Baseline Congruent	-3.7541	0.0736	[-3.9, -3.61]	100%
Baseline Incongruent	1.2744	0.0773	[1.13, 1.43]	100%
Reactive Congruent	-0.8985	0.0634	[-1.02, -0.77]	100%
Reactive x Incongruent	0.3246	0.0902	[0.15, 0.5]	99.99%

Note. The intercept term 'Baseline Congruent' refers to the log odds to make an error for Baseline congruent trials. 'R eactive x Incongruent' is the key effect and refers to the difference in TRCE Error across the Reactive and Baseline modes.

There was strong evidence for a greater TRCE Error in the Reactive versus Baseline mode (β = 0.32, se = 0.09, HDI = [0.15, 0.5], pd = 99.99%). Again this effect was opposite to what was observed in Tang et al. (2023).

Table 3: Hypothesis Test for Rea-Bas TRCE Error

Hypothesis	Estimate	SE	HDI	SDR	Post.Prob
Reactive - Baseline TRCE Error	-0.07	0.09	[0.15, 0.5]	0.99	0.00

Note. The key term in this table is the SDR score, which is computed as the ratio of the posterior distribution to the prior distribution at a specific point, in this case the mean of the prior distribution to investigate whether there is an increased likelihood of this value as a function of incoming data.

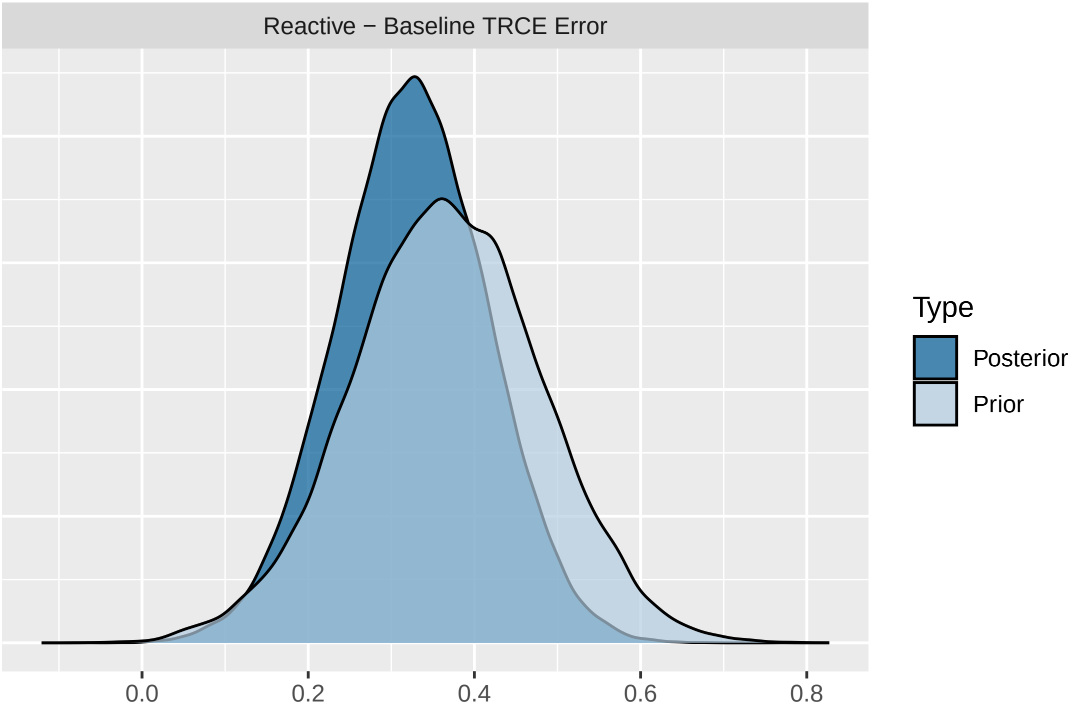

The plot indicates that the posterior does not overlap with the prior distribution. The graph is centered at the mean value of 2018 estimate, and indicates the SDR to be less than one (0.99) at that point. This suggests that the prior distribution slightly overestimates the TRCE Error between the Reactive and Baseline modes.

```
## Bayes Factors for Model Comparison
##
##     Model                                                    BF
## [1] 0 + Intercept + con.id * mode + (1 + con.id * mode | ID) 0.872
##
## * Against Denominator: [2] 0 + Intercept + con.id + mode + (1 + con.id + mode | ID)
## *   Bayes Factor Type: marginal likelihoods (bridgesampling)
```

The BF_{10} is greater than 1/10 but less than 10, indicating little practical evidence for a difference in the TRCE Error between the Reactive and Baseline modes.

5.4.2 Reactive - Proactive TRCE Error Logistic [with Max Random Effect Structure]

Table 4: Reactive - Proactive TRCE Error (null)

Term	Estimate	SE	HDI	pd
Proactive Congruent	-3.3948	0.0655	[-3.52, -3.27]	100%
Proactive Incongruent	1.4265	0.0751	[1.28, 1.58]	100%
Reactive Congruent	-1.2243	0.0704	[-1.36, -1.09]	100%

Note. The intercept term 'Proactive Congruent' refers to the average log odds to make an error for Proactive congruent trials. 'Reactive x Incongruent' is the key effect and refers to the difference in TRCE Error across the Reactive and Proactive modes.

Table 5: Reactive - Proactive TRCE Error (full)

Term	Estimate	SE	HDI	pd
Proactive Congruent	-3.3727	0.0654	[-3.5, -3.24]	100%
Proactive Incongruent	1.3577	0.0777	[1.21, 1.51]	100%
Reactive Congruent	-1.3188	0.0862	[-1.48, -1.15]	100%
Reactive x Incongruent	0.1950	0.1087	[-0.02, 0.41]	96.1%

Note. The intercept term 'Proactive Congruent' refers to the average log odds to make an error for Proactive congruent trials. 'Reactive x Incongruent' is the key effect and refers to the difference in the TRCE effect across the Reactive and Proactive modes.

There was not strong evidence of an increased TRCE Error between the Reactive and Proactive modes (β = 0.2, se = 0.11, HDI = [-0.02, 0.41], pd = 96.1%). Again this differs from what was found in Tang et al. (2023), in which a significant reduction in the TRCE Error was observed for Reactive.

Table 6: Hypothesis Test for Rea-Pro TRCE Error

Hypothesis	Estimate	SE	HDI	SDR	Post.Prob
Reactive - Proactive TRCE Error	-0.02	0.11	[-0.02, 0.41]	1.29	1.00

Note. The key term in this table is the SDR score, which is computed as the ratio of the posterior distribution to the prior distribution at a specific point, in this case the mean of the prior distribution to investigate whether there is an increased likelihood of this value as a function of incoming data.

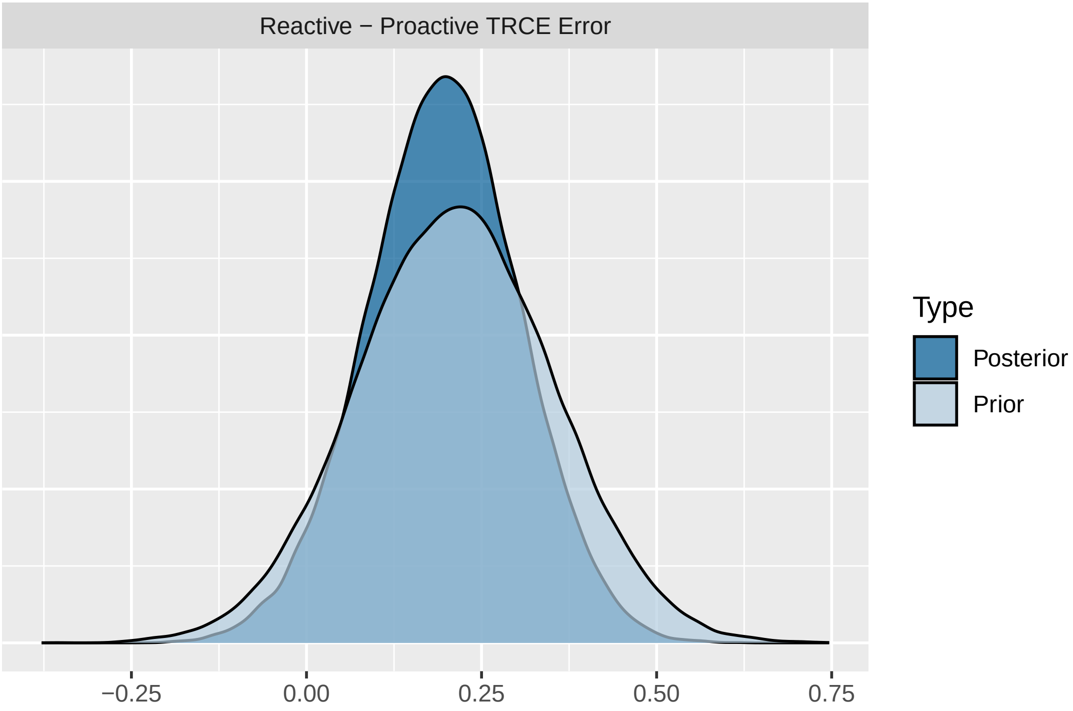

The plot indicates that the posterior closely overlaps with the prior distribution. The graph is centered at the mean value of 2018 estimate, and indicates the SDR to be greater than one (1.29) at that point. This suggests that the TRCE Error between the Reactive and Proactive modes is reliable across samples.

```
## Bayes Factors for Model Comparison
##
##     Model                                                   BF
## [1] 0 + Intercept + con.id * mode + (1 + con.id * mode | ID) 4.12
##
## * Against Denominator: [2] 0 + Intercept + con.id + mode + (1 + con.id + mode | ID)
## *   Bayes Factor Type: marginal likelihoods (bridgesampling)
```

The BF_{10} is greater than 1/10 but less than 10, meaning there was not strong evidence for the interaction effect.

5.4.3 Reactive - Baseline TRCE Error Logistic

Table 7: Reactive - Baseline TRCE Error (null)

Term	Estimate	SE	HDI	pd
Baseline Congruent	-3.7910	0.0711	[-3.93, -3.65]	100%
Baseline Incongruent	1.4055	0.0723	[1.26, 1.55]	100%
Reactive Congruent	-0.6704	0.0433	[-0.76, -0.59]	100%

Note. The intercept term 'Baseline Congruent' refers to the log odds to make an error for Baseline congruent trials.

Table 8: Reactive - Baseline TRCE Error (full)

Term	Estimate	SE	HDI	pd
Baseline Congruent	-3.7551	0.0742	[-3.9, -3.61]	100%
Baseline Incongruent	1.2651	0.0768	[1.12, 1.42]	100%
Reactive Congruent	-0.7826	0.0529	[-0.89, -0.68]	100%
Reactive x Incongruent	0.4077	0.0825	[0.25, 0.57]	100%

Note. The intercept term 'Baseline Congruent' refers to the log odds to make an error for Baseline congruent trials. 'Reactive x Incongruent' is the key effect and refers to the difference in TRCE Error across the Reactive and Baseline modes.

There was strong evidence for a greater TRCE Error in the Reactive versus Baseline mode (β = 0.41, se = 0.08, HDI = [0.25, 0.57], pd = 100%). Again this effect was opposite to what was observed in Tang et al. (2023).

Table 9: Hypothesis Test for Rea-Bas TRCE Error

Hypothesis	Estimate	SE	HDI	SDR	Post.Prob
Reactive - Baseline TRCE Error	0.04	0.08	[0.25, 0.57]	1.21	1.00

Note. The key term in this table is the SDR score, which is computed as the ratio of the posterior distribution to the prior distribution at a specific point, in this case the mean of the prior distribution to investigate whether there is an increased likelihood of this value as a function of incoming data.

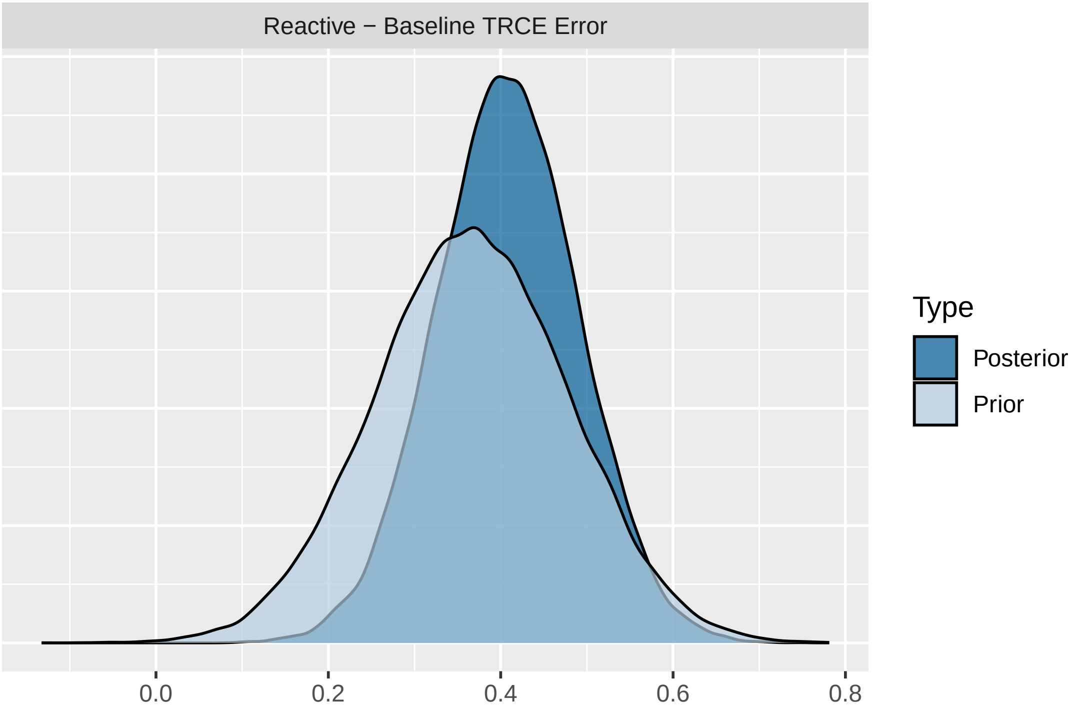

The plot indicates that the posterior closely overlaps the prior distribution. The graph is centered at the mean value of 2018 estimate, and indicates the SDR to be greater than one (1.21) at that point. This suggests that the posterior and prior distributions for the TRCE error effect between the Reactive and Baseline modes do not significantly differ from one another.

```
## Bayes Factors for Model Comparison
##
##     Model                                                   BF
## [1] 0 + Intercept + con.id * mode + (1 + con.id | ID) 12.86
##
## * Against Denominator: [2] 0 + Intercept + con.id + mode + (1 + con.id | ID)
## *   Bayes Factor Type: marginal likelihoods (bridgesampling)
```

The BF_{10} is greater than 10, indicating strong evidence for a difference in a greater TRCE Error in the Reactive versus Baseline modes.

5.4.4 Reactive - Proactive TRCE Error Logistic

Table 10: Reactive - Proactive TRCE Error (null)

Term	Estimate	SE	HDI	pd
Proactive Congruent	-3.4013	0.0670	[-3.53, -3.27]	100%
Proactive Incongruent	1.4242	0.0736	[1.28, 1.57]	100%
Reactive Congruent	-1.0190	0.0402	[-1.1, -0.94]	100%

Note. The intercept term 'Proactive Congruent' refers to the average log odds to make an error for Proactive congruent trials. 'Reactive x Incongruent' is the key TRCE error effect and refers to the difference in the TRCE effect across the Reactive and Proactive modes.

Table 11: Reactive - Proactive TRCE Error (full)

Term	Estimate	SE	HDI	pd
Proactive Congruent	-3.3929	0.0666	[-3.52, -3.26]	100%
Proactive Incongruent	1.3692	0.0759	[1.22, 1.52]	100%
Reactive Congruent	-1.0636	0.0509	[-1.16, -0.96]	100%
Reactive x Incongruent	0.2095	0.0796	[0.05, 0.36]	99.61%

Note. The intercept term 'Proactive Congruent' refers to the average log odds to make an error for Proactive congruent trials. 'Reactive x Incongruent' is the key effect and refers to the difference in the TRCE Error across the Reactive and Proactive modes.

There was also strong evidence of an increased TRCE Error between the Reactive and Proactive modes (β = 0.21, se = 0.08, HDI = [0.05, 0.36], pd = 99.61%). Again this differs from what was found in Tang et al. (2023), in which a significant reduction in the TRCE Error was observed for Reactive.

Table 12: Hypothesis Test for Rea-Pro TRCE Error

Hypothesis	Estimate	SE	HDI	SDR	Post.Prob
Reactive - Proactive TRCE Error	0.07	0.08	[0.05, 0.36]	0.95	0.00

Note. The key term in this table is the SDR score, which is computed as the ratio of the posterior distribution to the prior distribution at a specific point, in this case the mean of the prior distribution to investigate whether there is an increased likelihood of this value as a function of incoming data.

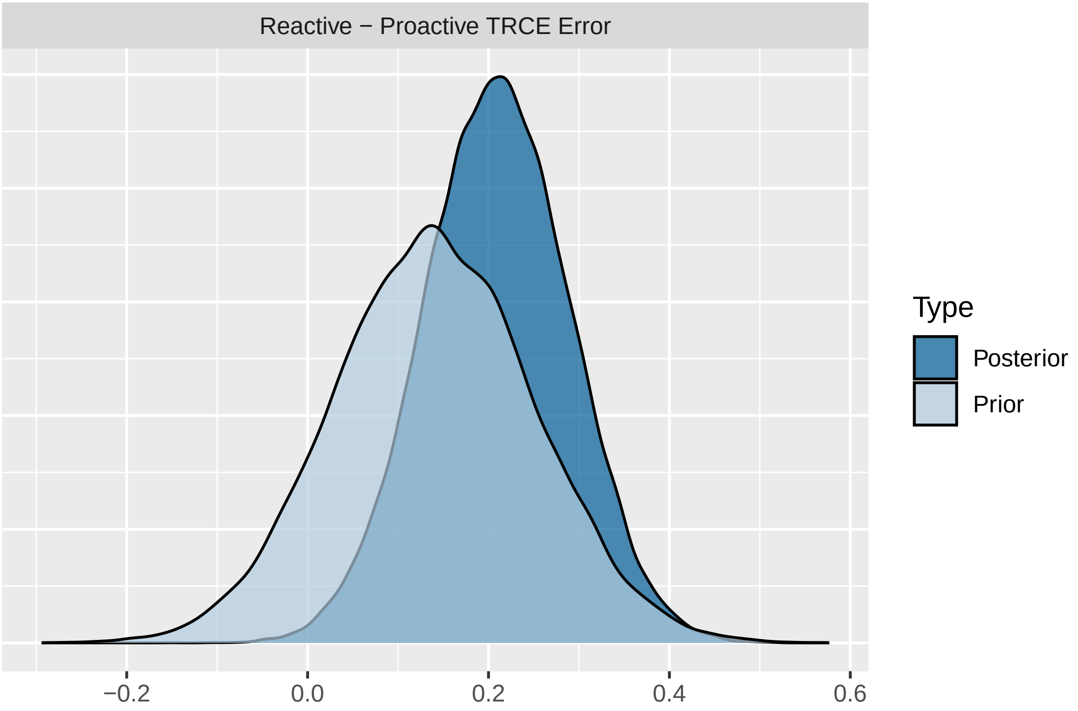

The plot indicates that the posterior shifts away from the prior distribution. The graph is centered at the mean value of 2018 estimate, and indicates the SDR to be less than one (0.95) at that point. This suggests that the prior underestimates the difference in the TRCE Error between the Reactive and Proactive modes.

```
## Bayes Factors for Model Comparison
##
##     Model                                               BF
## [1] 0 + Intercept + con.id * mode + (1 + con.id | ID) 6.26
##
## * Against Denominator: [2] 0 + Intercept + con.id + mode + (1 + con.id | ID)
## *   Bayes Factor Type: marginal likelihoods (bridgesampling)
```

The BF_{10} is greater than 1/10 but less than 10, indicating little practical evidence for a difference in a greater TRCE Error in the Reactive versus Baseline modes.

5.6.5 Main Reactive and Baseline Logistic

Table 13: Reactive and Baseline Cued-TS Error

Term	Estimate	SE	HDI	pd
Intercept	-3.6279	0.0602	[-3.75, -3.51]	100%

Note. The intercept term 'Intercept' refers to the average log odds to make an error for Baseline and Reactive trials.

Table 14: Reactive - Baseline Cued-TS Error Main E˙ect

Term	Estimate	SE	HDI	pd
Baseline	-3.3331	0.0609	[-3.45, -3.21]	100%
Reactive	-0.6516	0.0420	[-0.73, -0.57]	100%

Note. The intercept term 'Baseline' refers to the average log odds to make an error for Baseline trials. 'Reactive' is the difference between trials across the Reactive and Baseline modes.

There was decisive evidence for a main effect between the Baseline and Reactive modes (β = -0.65, se = 0.04, HDI = [-0.73, -0.57], pd = 100%). This result shows the Reactive task manipulation reduced the log odds to make an error on biased trials.

```
## Recompilation done
```

```
## Bayes Factors for Model Comparison
##
##     Model                                BF
## [1] 0 + Intercept + mode + (1 | ID) 9.22e+08
##
```

```
## * Against Denominator: [2] 0 + Intercept + (1 | ID)
## *   Bayes Factor Type: marginal likelihoods (bridgesampling)
```

The BF_{10} is much greater than 10, indicating a main effect of mode between the Proactive and Reactive conditions is significant for biased trials.

5.6.6 Main Reactive and Proactive Logistic

Table 15: Reactive and Proactive Cued-TS Error

Term	Estimate	SE	HDI	pd
Intercept	-3.3152	0.0511	[-3.42, -3.22]	100%

Note. The intercept term 'Intercept' refers to the average log odds to make an error for Proactive and Reactive trials.

Table 16: Reactive - Proactive Error Main E˙ect

Term	Estimate	SE	HDI	pd
Proactive	-2.9176	0.0544	[-3.03, -2.81]	100%
Reactive	-0.9785	0.0400	[-1.05, -0.9]	100%

Note. The intercept term 'Intercept' refers to the average log odds to make an error for Proactive and Reactive trials.

There was decisive evidence for a main effect between the Reactive and Proactive modes (β = -0.98, se = 0.04, HDI = [-1.05, -0.9], pd = 100%). This result shows the Reactive task manipulation reduced the log odds to make an error on biased trials.

```
## Bayes Factors for Model Comparison
##
##     Model                                 BF
## [1] 0 + Intercept + mode + (1 | ID) 8.23e+46
##
```

```
## * Against Denominator: [2] 0 + Intercept + (1 | ID)
## *   Bayes Factor Type: marginal likelihoods (bridgesampling)
```

The BF_{10} was much greater than 10, indicating that the full model M_1 was strongly favored over the null model M_0. This finding provides stronger evidence for a TRCE Error than that obtained in Tang et al (2023).

5.6.7 Incongruent Reactive and Baseline Logistic

Table 17: Reactive and Baseline Cued-TS Incongruent Effect

Term	Estimate	SE	HDI	pd
Incongruent	-2.6893	0.0690	[-2.82, -2.55]	100%

Note. The intercept term 'Incongruent' refers to the average log odds to make an error for Baseline and Reactive Incongruent trials.

Table 18: Reactive - Baseline Cued-TS Incongruent Effect

Term	Estimate	SE	HDI	pd
Baseline Incongruent	-2.4539	0.0738	[-2.6, -2.31]	100%
Reactive Incongruent	-0.4738	0.0679	[-0.61, -0.34]	100%

Note. The intercept term 'Baseline Incongruent' refers to the average log odds to make an error for Baseline Incongruent trials. 'Reactive Incongruent' is the difference between incongruent trials across the Reactive and Baseline modes.

There was a decisive effect of condition across the Baseline and Reactive modes (β = -0.47, se = 0.07, HDI = [-0.61, -0.34], pd = 100%). This result more specifically shows the Reactive task manipulation reduces the log odds to make an error on biased incongruent trials.

```
## Bayes Factors for Model Comparison
##
##     Model                                BF
## [1] 0 + Intercept + mode + (1 | ID) 5.19
##
## * Against Denominator: [2] 0 + Intercept + (1 | ID)
## *   Bayes Factor Type: marginal likelihoods (bridgesampling)
```

The BF_{10} is greater than 1/10 but less than 10, indicating inconclusive evidence on whether there was a meaningful difference in the log odds to make an error between the Baseline and Reactive modes for biased incongruent trials.

5.6.8 Incongruent Reactive and Proactive Logistic

Table 19: Reactive - Proactive Cued-TS Incongruent Error Effect

Term	Estimate	SE	HDI	pd
Incongruent	-2.3887	0.0663	[-2.52, -2.26]	100%

Note. The intercept term 'Proactive Incongruent' refers to the average log odds to make an error for Proactive Incongruent trials. 'Reactive Incongruent' is the difference between incongruent trials across the Reactive and Proactive modes.

Table 20: Reactive and Proactive Cued-TS Error Incongruent Effect

Term	Estimate	SE	HDI	pd
Proactive Incongruent	-2.0022	0.0685	[-2.14, -1.87]	100%
Reactive Incongruent	-0.9153	0.0629	[-1.04, -0.79]	100%

Note. The intercept term 'Proactive Incongruent' refers to the average log odds to make an error for Proactive Incongruent trials. 'Reactive Incongruent' is the difference between incongruent trials across the Reactive and Proactive modes

There was decisive evidence for an effect of mode across the Reactive and Proactive modes (β = -0.92, se = 0.06, HDI = [-1.04, -0.79], pd = 100%). This result more specifically shows the Reactive task manipulation reduces the log odds to make an error on biased incongruent trials.

```
## Bayes Factors for Model Comparison
##
##     Model                                   BF
## [1] 0 + Intercept + mode + (1 | ID) 1.15e+10
##
```

```
## * Against Denominator: [2] 0 + Intercept + (1 | ID)
## *   Bayes Factor Type: marginal likelihoods (bridgesampling)
```

The BF_{10} was much greater than 10, indicating the difference in the log odds to make an error between the Proactive and Reactive mode is strong for biased incongruent trials.

5.6.9 Marginal Means Reactive and Baseline Logistic

Table 21: Reactive - Baseline TRCE Error

Term	Estimate	SE	HDI	pd
Baseline Congruent	-3.8260	0.0702	[-3.95, -3.68]	100%
Baseline Incongruent	-2.5246	0.0702	[-2.66, -2.38]	100%
Reactive Congruent	-4.5238	0.0754	[-4.68, -4.38]	100%
Reactive x Incongruent	-2.9284	0.0766	[-3.08, -2.77]	100%

Note. The marginal means for each con.id and mode. The intercept was removed for this analysis

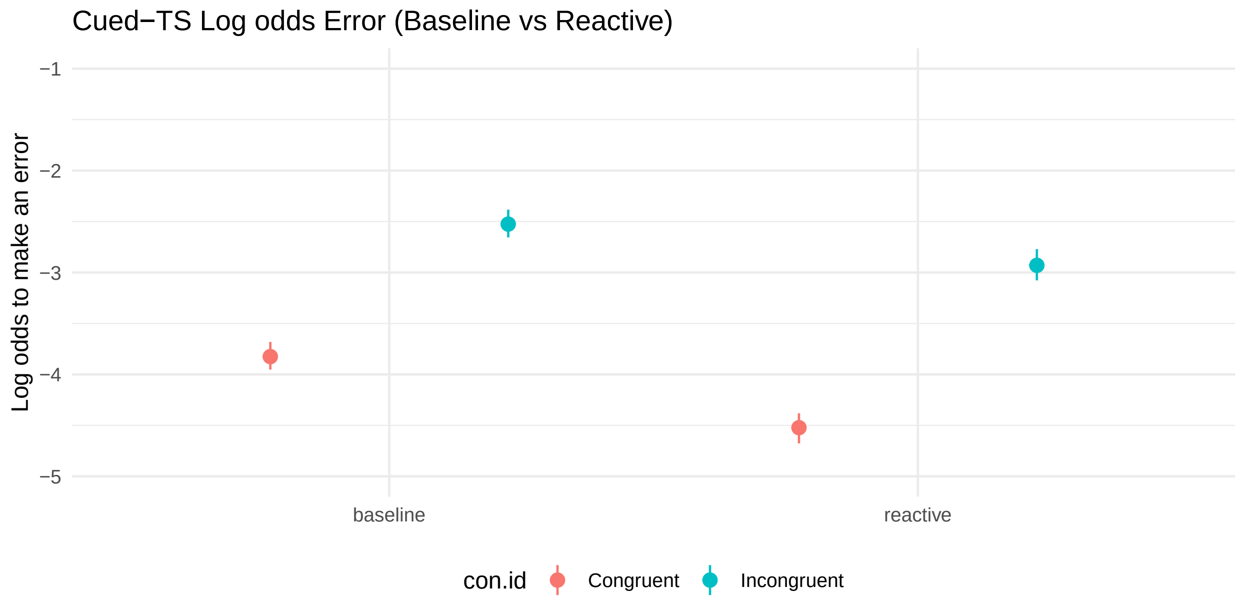

5.6.10 Marginal Means Reactive and Proactive Logistic

Table 22: Reactive - Proactive TRCE Error

Term	Estimate	SE	HDI	pd
Proactive Congruent	-3.4122	0.0620	[-3.54, -3.29]	100%
Proactive Incongruent	-2.0855	0.0675	[-2.21, -1.95]	100%
Reactive Congruent	-4.5000	0.0711	[-4.64, -4.36]	100%
Reactive x Incongruent	-2.9227	0.0784	[-3.07, -2.76]	100%

Note. The marginal means for each con.id and mode. The intercept was removed for this analysis.

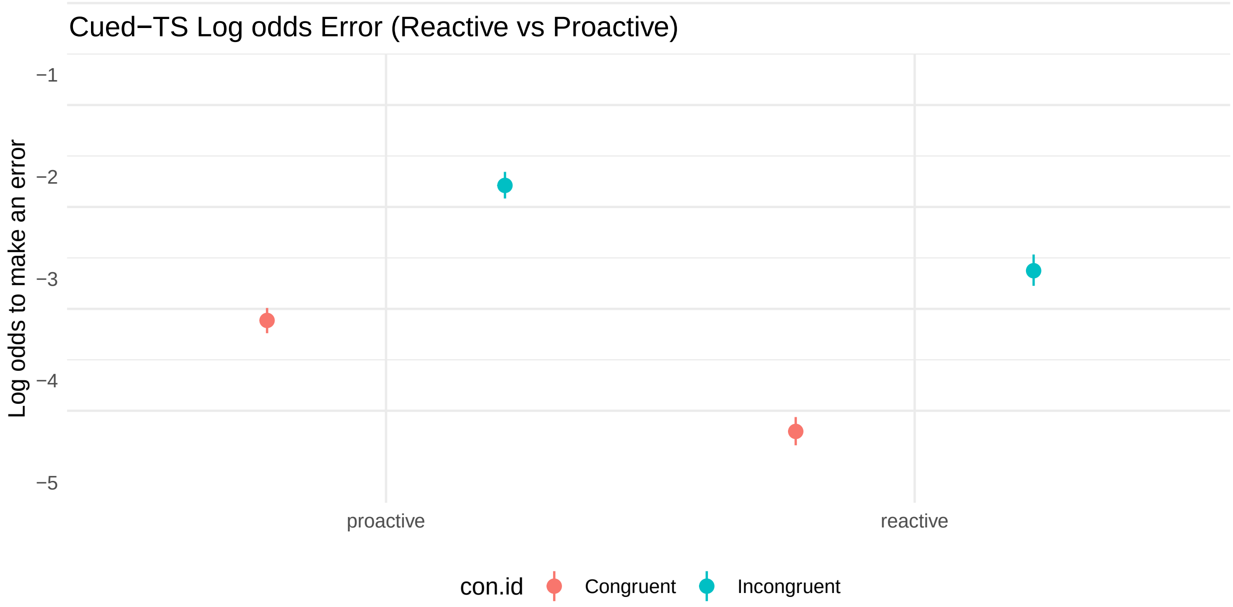

5.6.11 Reactive - Baseline TRCE Error Rate

Table 23: Reactive - Baseline TRCE Error (null)

Term	Estimate	SE	HDI	pd
Baseline Congruent	0.0396	0.0025	[0.03, 0.04]	100%
Baseline Incongruent	0.0589	0.0050	[0.05, 0.07]	100%
Reactive Congruent	-0.0253	0.0027	[-0.03, -0.02]	100%

Note. The intercept term 'Baseline Congruent' refers to the log odds to make an error for Baseline congruent trials.

Table 24: Reactive - Baseline TRCE Error (full)

Term	Estimate	SE	HDI	pd
Baseline Congruent	0.0352	0.0028	[0.03, 0.04]	100%
Baseline Incongruent	0.0667	0.0055	[0.06, 0.08]	100%
Reactive Congruent	-0.0159	0.0035	[-0.02, -0.01]	100%
Reactive x Incongruent	-0.0123	0.0050	[-0.02, 0]	99.36%

Note. The intercept term 'Baseline Congruent' refers to the log odds to make an error for Baseline congruent trials. 'Reactive x Incongruent' is the key effect and refers to the difference in TRCE effects across the Reactive and Baseline modes.

There is strong evidence for a reduced TRCE Error in the Reactive versus Baseline mode for error rates (β = -0.01, se = 0, HDI = [-0.02, 0], pd = 99.36%). This effect is notably different from what was found in the Reactive - Baseline model using logistic regression.

```
## Bayes Factors for Model Comparison
##
##     Model                                                  BF
## [1] 0 + Intercept + con.id * mode + (1 + con.id | ID) 1.01
##
## * Against Denominator: [2] 0 + Intercept + con.id + mode + (1 + con.id | ID)
## *   Bayes Factor Type: marginal likelihoods (bridgesampling)
```

The BF_{10} is greater than 1/10 but less than 10, indicating strong evidence for a reduction in TRCE Error between the Baseline and Reactive mode for biased trials.

5.6.12 Reactive - Proactive TRCE Error Rate

Table 25: Reactive - Proactive TRCE Error (null)

Term	Estimate	SE	HDI	pd
Proactive Congruent	0.0589	0.0030	[0.05, 0.06]	100%
Reactive Incongruent	0.0762	0.0057	[0.06, 0.09]	100%
Reactive Congruent	-0.0520	0.0034	[-0.06, -0.05]	100%

Note. The intercept term 'Proactive Congruent' refers to the average log odds to make an error for Proactive congruent trials.

Table 26: Reactive - Proactive TRCE Error (full)

Term	Estimate	SE	HDI	pd
Proactive Congruent	0.0451	0.0032	[0.04, 0.05]	100%
Proactive Incongruent	0.1027	0.0063	[0.09, 0.12]	100%
Reactive Congruent	-0.0239	0.0042	[-0.03, -0.02]	100%
Reactive x Incongruent	-0.0498	0.0061	[-0.06, -0.04]	100%

Note. The intercept term 'Proactive Congruent' refers to the error rate for Proactive congruent trials. 'Reactive x Incongruent' is the key effect and refers to the difference in TRCE Error across the Reactive and Proactive modes.

There is decisive evidence for a reduced TRCE Error in the Reactive mode versus Proactive mode for error rates (β = -0.05, se = 0.01, HDI = [-0.06, -0.04], pd = 100%). This effect is notably different from the Reactive - Proactive model using logistic regression.

```
## Bayes Factors for Model Comparison
##
##     Model                                                    BF
## [1] 0 + Intercept + con.id * mode + (1 + con.id | ID) 4.68
##
## * Against Denominator: [2] 0 + Intercept + con.id + mode + (1 + con.id | ID)
## *   Bayes Factor Type: marginal likelihoods (bridgesampling)
```

The BF_{10} is greater than 1/10 but less than 10, indicating little evidence for a meaningful reduction in TRCE Error between the Proactive and Reactive modes for biased trials.

5.6.13 Main Reactive and Baseline Error rate

Table 27: Reactive and Baseline Cued-TS Error Main Effect

Term	Estimate	SE	HDI	pd
Intercept	0.0550	0.0032	[0.05, 0.06]	100%

Note. The intercept term 'Intercept' refers to the average error rate to make an error for Baseline and Reactive trials.

Table 28: Reactive - Baseline Cued-TS Error Main Effect

Term	Estimate	SE	HDI	pd
Baseline	0.0691	0.0038	[0.06, 0.08]	100%
Reactive	-0.0246	0.0040	[-0.03, -0.02]	100%

Note. The intercept term 'Baseline' refers to the average error rate for Baseline trials. 'Reactive' is the difference between trials across the Reactive and Baseline modes.

There is decisive evidence for a greater main effect of mode in the Reactive versus Baseline mode for error rates (β = -0.02, se = 0, HDI = [-0.03, -0.02], pd = 100%). This result shows the Reactive task manipulation reduces error rate on biased trials.

```
## Bayes Factors for Model Comparison
##
##     Model                              BF
## [1] 0 + Intercept + mode + (1 | ID) 24.14
##
```

```
## * Against Denominator: [2] 0 + Intercept + (1 | ID)
## *   Bayes Factor Type: marginal likelihoods (bridgesampling)
```

The BF_{10} was much greater than 10, indicating strong evidence for a main effect between the Baseline and Reactive mode for biased trials.

5.6.14 Main Reactive and Proactive Error Rates

Table 29: Reactive and Proactive Cued-TS Error Main Effect

Term	Estimate	SE	HDI	pd
Intercept	0.0690	0.0032	[0.06, 0.08]	100%

Note. The intercept term 'Intercept' refers to the average error rate to make an error for Proactive and Reactive trials.

Table 30: Reactive and Proactive Cued-TS Error Main Effect

Term	Estimate	SE	HDI	pd
Proactive	0.0969	0.0040	[0.09, 0.1]	100%
Reactive	-0.0518	0.0050	[-0.06, -0.04]	100%

Note. The intercept term 'Intercept' refers to the average error rate for Proactive and Reactive trials.

There is decisive evidence for a main effect of mode in the Reactive versus Proactive mode for error rates (β = -0.02, se = 0, HDI = [-0.03, -0.02], pd = 100%). This result shows the Reactive task manipulation reduces error rate on biased trials.

```
## Bayes Factors for Model Comparison
##
##     Model                                BF
## [1] 0 + Intercept + mode + (1 | ID) 7.93e+07
##
```

```
## * Against Denominator: [2] 0 + Intercept + (1 | ID)
## *   Bayes Factor Type: marginal likelihoods (bridgesampling)
```

The BF_{10} was much greater than 10, indicating strong evidence for a strong effect between the Proactive and Reactive mode for biased trials.

5.6.15 Incongruent Reactive and Baseline Error Rates

Table 31: Reactive and Baseline Cued-TS Error Incongruent Effect

Term	Estimate	SE	HDI	pd
Incongruent	0.0845	0.0054	[0.07, 0.09]	100%

Note. The intercept term 'Incongruent' refers to the average error rate for Baseline and Reactive Incongruent trials.

Table 32: Reactive - Baseline Cued-TS Error Incongruent Effect

Term	Estimate	SE	HDI	pd
Baseline Incongruent	0.1028	0.0058	[0.09, 0.11]	100%
Reactive Incongruent	-0.0329	0.0056	[-0.04, -0.02]	100%

Note. The intercept term 'Baseline Incongruent' refers to the average error rate for Baseline Incongruent trials. 'Reactive Incongruent' is the difference between incongruent trials across the Reactive and Baseline modes.

There is a decisive effect of mode across the Baseline and Reactive modes (β = -0.03, se = 0.01, HDI = [-0.04, -0.02], pd = 100%). This result more specifically shows the Reactive task manipulation reduces the error rate on biased incongruent trials.

```
## Bayes Factors for Model Comparison
##
##     Model                              BF
## [1] 0 + Intercept + mode + (1 | ID) 3.86
##
## * Against Denominator: [2] 0 + Intercept + (1 | ID)
## *   Bayes Factor Type: marginal likelihoods (bridgesampling)
```

The BF_{10} is greater than 1/10 but less than 10, indicating the difference in error rates between the Reactive and Baseline mode is notable for biased incongruent trials.

5.6.16 Incongruent Reactive and Proactive Error Rates

Table 33: Reactive and Proactive Cued-TS Error Incongruent Effect

Term	Estimate	SE	HDI	pd
Incongruent	0.1071	0.0057	[0.1, 0.12]	100%

Note. The intercept term 'Incongruent' refers to the average error rate for Reactive and Proactive Incongruent trials.

Table 34: Reactive - Proactive Cued-TS Error Incongruent Effect

Term	Estimate	SE	HDI	pd
Proactive Incongruent	0.1482	0.0064	[0.14, 0.16]	100%
Reactive Incongruent	-0.0780	0.0068	[-0.09, -0.06]	100%

Note. The intercept term 'Proactive Incongruent' refers to the average error rate for Proactive Incongruent trials. 'Reactive Incongruent' is the difference between incongruent trials across the Reactive and Proactive modes.

There is decisive evidence for an effect of mode across the Proactive and Reactive modes (β = -0.08, se = 0.01, HDI = [-0.09, -0.06], pd = 100%). This result more specificall y shows the Reactive task manipulation reduces the error rate on biased incongruent trials.

```
## Bayes Factors for Model Comparison
##
##     Model                                   BF
## [1] 0 + Intercept + mode + (1 | ID) 9.43e+04
##
## * Against Denominator: [2] 0 + Intercept + (1 | ID)
## *   Bayes Factor Type: marginal likelihoods (bridgesampling)
```

The BF_{10} was much greater than 10, indicating the difference in error rates between the Proactive and Reactive mode is notable for biased incongruent trials.

5.6.17 Marginal Means Reactive and Baseline Error Rates

Table 35: Cued-TS Error Rates (Reactive vs Baseline)

Term	Estimate	SE	HDI	pd
Baseline Congruent	0.0345	0.0029	[0.03, 0.04]	100%
Baseline Incongruent	0.0967	0.0052	[0.09, 0.11]	100%
Reactive Congruent	0.0159	0.0029	[0.01, 0.02]	100%
Reactive Incongruent	0.0695	0.0051	[0.06, 0.08]	100%

Note. The marginal means for each con.id and mode. The intercept was removed for this analysis.

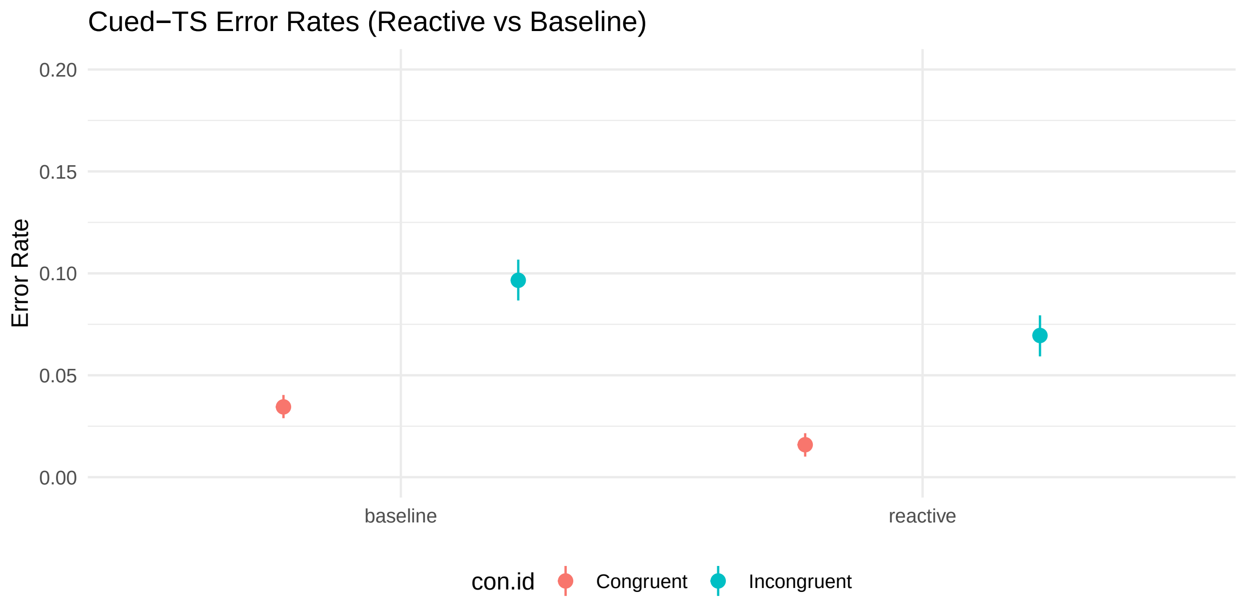

5.6.18 Marginal Means Reactive and Proactive Error Rates

Table 36: Cued-TS Error Rates (Proactive vs Reactive)

Term	Estimate	SE	HDI	pd
Proactive Congruent	0.0455	0.0035	[0.04, 0.05]	100%
Proactive Incongruent	0.1412	0.0057	[0.13, 0.15]	100%
Reactive Congruent	0.0160	0.0035	[0.01, 0.02]	100%
Reactive x Incongruent	0.0692	0.0057	[0.06, 0.08]	100%

Note. The marginal means for each con.id and mode. The intercept was removed for this analysis

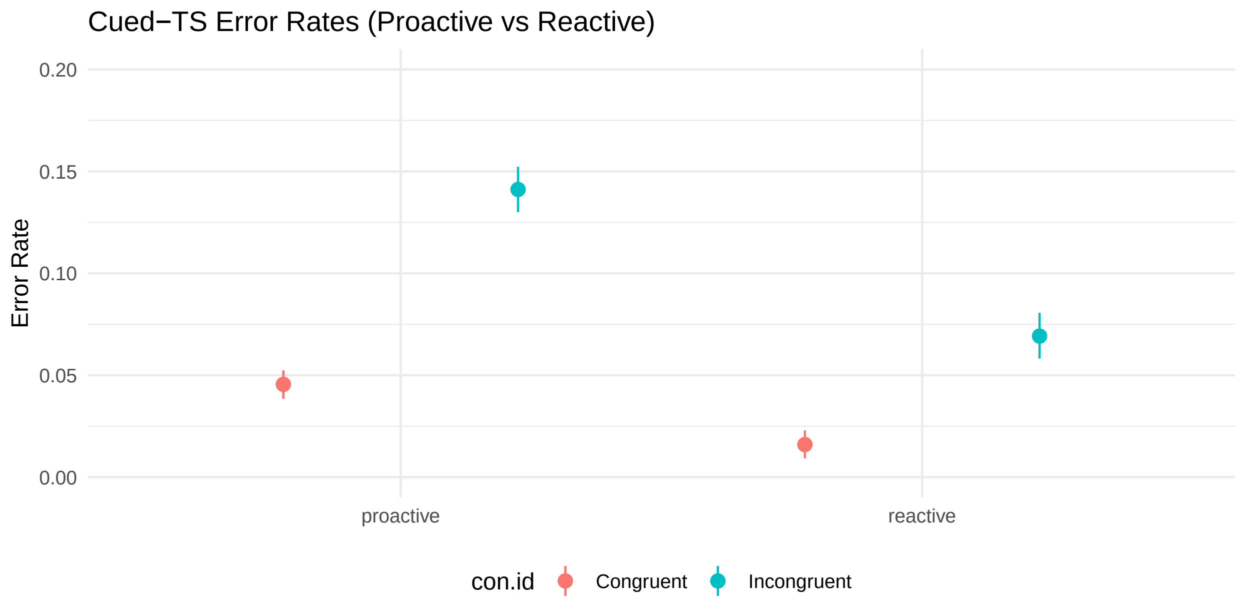

5.5 TRCE RT 2018

The task-rule congruency effect (TRCE) is defined as the increase in both errors and RT on incongruent trials (the two task rules are associated with different responses to the target stimulus) relative to congruent trials (the two task rules are associated with the same response to the target stimulus).

TRCE RT was examined as the [shifted log-normal] RT to make a correct response on incongruent trials relative to congruent ones. Non-incentivized [biased] trials were included to directly compare across modes, since these are well-matched. TRCE RT was predicted to be higher in Reactive relative to Baseline and Proactive. Mode and con.id are dummy coded to compute the difference in the TRCE effect across sessions. The intercept and con.id were entered as random effects nested within subject to account for trial-level variability.

Wilkinson Notation

$$RT = mode\ x\ con.id\ + \left(1 + con.id \mid ID\right), \text{family}\left(\text{shifted lognormal}\right)$$

Fully Indexed Notation

$$\begin{gathered}
\text{RT}_{i,t} \sim \text{shifted log-normal}\left(\mu_{i,t}, \sigma, \theta\right) \\
\log\left(\mu_{i,t} + \theta\right) = \beta_{0i} + \beta_1 * mode_t\ x\ \beta_{2i} * con.id_{i,t} \\
\begin{bmatrix}\beta_{0i} \\ \beta_{2i}\end{bmatrix} \sim \text{N}\left(\begin{bmatrix}\beta_0 \\ \beta_2\end{bmatrix}, \Sigma\right) \\
\beta_0, \beta_1, \beta_2 \sim \text{flat}, \Sigma \sim \text{LKJ}(1), \sigma \sim \text{student-t}(3,0,\ 2.5), \theta \sim \text{uniform}(0,\ \text{minRT})
\end{gathered}$$

5.5.1 Reactive - Baseline TRCE RT

Table 1: Reactive - Baseline TRCE RT (null))

Term	Estimate	SE	HDI	pd
Baseline Congruent	6.3274	0.0293	[6.27, 6.38]	100%
Baseline Incongruent	0.1059	0.0122	[0.08, 0.13]	100%
Reactive Congruent	0.1239	0.0040	[0.12, 0.13]	100%

Note. The intercept term 'Baseline Congruent' refers to the [shifted log-normal] RT make a correct response for Baseline congruent trials.

Table 2: Reactive - Baseline TRCE RT (full)

Term	Estimate	SE	HDI	pd
Baseline Congruent	6.3283	0.0296	[6.27, 6.39]	100%
Baseline Incongruent	0.0991	0.0133	[0.07, 0.13]	100%
Reactive Congruent	0.1218	0.0045	[0.11, 0.13]	100%
Reactive x Incongruent	0.0137	0.0112	[-0.01, 0.04]	88.7%

Note. The intercept term 'Baseline Congruent' refers to the [shifted log-normal] RT make a correct response for Baseline congruent trials. 'R eactive x Incongruent' is the key effect and refers to the difference in TRCE Error across the Reactive and Baseline modes.

The TRCE RT was not reliably different across the Reactive and Baseline mode while assuming a shifted log-normal distribution (β = 0.01, se = 0.01, HDI = [-0.01, 0.04], pd = 88.7%).

Table 3: Reactive - Baseline TRCE RT (Gaussian)

Term	Estimate	SE	HDI	pd
Baseline Congruent	906.2639	24.0788	[860.18, 953.29]	100%
Baseline Incongruent	75.7962	11.9586	[52.56, 99.5]	100%
Reactive Congruent	85.7282	4.0015	[78.02, 93.66]	100%
Reactive x Incongruent	12.6081	10.0386	[-7.18, 32.17]	89.48%

Note. The intercept term 'Baseline Congruent' refers to the RT make a correct response for Baseline congruent trials. 'Reactive x Incongruent' is the key effect and refers to the difference in TRCE RT across the Reactive and Baseline modes.

The TRCE RT was not reliably different across the Reactive versus Baseline mode while assuming a Gaussian distribution (β = 12.61, se = 10.04, HDI = [-7.18, 32.17], pd = 89.48%).

Table 4: Reactive - Baseline TRCE RT (ex-Gaussian)

Term	Estimate	SE	HDI	pd
Baseline Congruent	922.7390	9.4725	[860.18, 953.29]	100%
Baseline Incongruent	20.5190	3.7400	[52.56, 99.5]	100%
Reactive Congruent	51.9307	1.7634	[78.02, 93.66]	100%
Reactive x Incongruent	3.6279	4.5454	[-7.18, 32.17]	78.89%

Note. The intercept term 'Baseline Congruent' refers to the RT make a correct response for Baseline congruent trials. 'Reactive x Incongruent' is the key effect and refers to the difference in TRCE RT across the Reactive and Baseline modes.

The TRCE RT was not reliably different in the Reactive and Baseline mode while assuming an ex-Gaussian distribution (β = 3.63, se = 4.55, HDI = [-7.18, 32.17], pd = 78.89%).

5.5.2 Reactive - Proactive TRCE RT

Table 5: Reactive - Proactive TRCE RT (null)

Term	Estimate	SE	HDI	pd
Proactive Congruent	6.0295	0.0299	[5.97, 6.09]	100%
Proactive Incongruent	0.1062	0.0119	[0.08, 0.13]	100%
Reactive Congruent	0.4262	0.0037	[0.42, 0.43]	100%

Note. The intercept term 'Proactive Congruent' refers to the [shifted log-normal] RT to make a correct response for Proactive congruent trials.

Table 6: Reactive - Proactive TRCE RT (full)

Term	Estimate	SE	HDI	pd
Proactive Congruent	6.0305	0.0281	[5.98, 6.09]	100%
Proactive Incongruent	0.0974	0.0130	[0.07, 0.12]	100%
Reactive Congruent	0.4236	0.0041	[0.42, 0.43]	100%
Reactive x Incongruent	0.0168	0.0103	[0, 0.04]	94.79%

Note. The intercept term 'Proactive Congruent' refers to the average [shifted log-normal] RT to make a correct response for Proactive congruent trials. 'Reactive Incongruent' is the key effect and refers to the difference in TRCE RT across the Proactive and Reactive modes.

There was no reliable difference in TRCE RT between the Reactive versus Proactive modes while assuming a shifted log-normal distribution (β = 0.02, se = 0.01, HDI = [0, 0.04], pd = 94.79%). The Reactive vs. Proactive contrast was *not* actually reported in Tang et al main text.

Table 7: Reactive - Proactive TRCE RT (Gaussian)

Term	Estimate	SE	HDI	pd
Proactive Congruent	714.3253	22.2063	[671.66, 758.52]	100%
Proactive Incongruent	56.8583	10.9574	[35.34, 78.12]	100%
Reactive Congruent	273.7648	3.5087	[266.95, 280.62]	100%
Reactive x Incongruent	32.5493	8.9161	[14.33, 49.37]	99.98%

Note. The intercept term 'Proactive Congruent' refers to the average RT to make a correct response for Proactive congruent trials. 'Reactive Incongruent' is the key effect and refers to the difference in TRCE RT across the Proactive and Reactive modes.

There was strong evidence for an increased TRCE RT for the Reactive versus Proactive mode while assuming a Gaussian distribution (β = 32.55, se = 8.92, HDI = [14.33, 49.37], pd = 99.98%).

Table 8: Reactive - Proactive TRCE RT (ex-Gaussian)

Term	Estimate	SE	HDI	pd
Proactive Congruent	796.0764	8.8975	[778.51, 813.18]	100%
Proactive Incongruent	15.0894	3.4114	[8.46, 21.78]	100%
Reactive Congruent	116.7468	1.5804	[113.55, 119.76]	100%
Reactive x Incongruent	10.9033	3.9878	[3, 18.64]	99.7%

Note. The intercept term 'Proactive Congruent' refers to the average RT to make a correct response for Proactive congruent trials. 'Reactive Incongruent' is the key effect and refers to the difference in TRCE RT across the Proactive and Reactive modes.

There was also strong evidence for an increased TRCE RT for the Reactive versus Proactive mode while assuming an ex-Gaussian distribution (β = 10.9, se = 3.99, HDI = [3, 18.64], pd = 99.7%).

5.6 TRCE RT 2020

These analyses test for a consistent pattern across the 2018 and 2020 datasets, using the 2018 estimates as prior information.

5.6.1 Reactive - Baseline TRCE RT

Table 1: Reactive - Baseline TRCE RT (null)

Term	Estimate	SE	HDI	pd
Baseline Congruent	6.3275	0.0294	[6.27, 6.39]	100%
Baseline Incongruent	0.1059	0.0123	[0.08, 0.13]	100%
Reactive Congruent	0.1238	0.0040	[0.12, 0.13]	100%

Note. The intercept term 'Baseline Congruent' refers to the [shifted log-normal] RT make a correct response for Baseline congruent trials

Table 2: Reactive - Baseline TRCE RT (full)

Term	Estimate	SE	HDI	pd
Baseline Congruent	6.3026	0.0225	[6.26, 6.35]	100%
Baseline Incongruent	0.0972	0.0093	[0.08, 0.12]	100%
Reactive Congruent	0.0401	0.0037	[0.03, 0.05]	100%
Reactive x Incongruent	-0.0342	0.0087	[-0.05, -0.02]	100%

*Note.*The intercept term 'Baseline Congruent' refers to the [shifted log-normal] RT make a correct response for Baseline congruent trials.
'Reactive x Incongruent' is the key effect and refers to the difference in TRCE RT across the Reactive and Baseline modes.

There was strong evidence for a reduced TRCE RT in the Reactive relative to Baseline mode while assuming a shifted log-normal distribution (β = -0.03, se = 0.01, HDI = [-0.05, -0.02], pd = 100%). NOTE that this pattern was opposite in direction from that reported in Tang et al (2023).

Table 3: Hypothesis Test for Rea-Bas TRCE RT

Hypothesis	Estimate	SE	HDI	SDR	Post.Prob
Reactive - Baseline TRCE RT	-0.05	0.01	[-0.05, -0.02]	0.00	0.00

Note. The key term in this table is the SDR score, which is computed as the ratio of the posterior distribution to the prior distribution at a specific point, in this case the mean of the prior distribution to investigate whether there is an increased likelihood of this value as a function of incoming data.

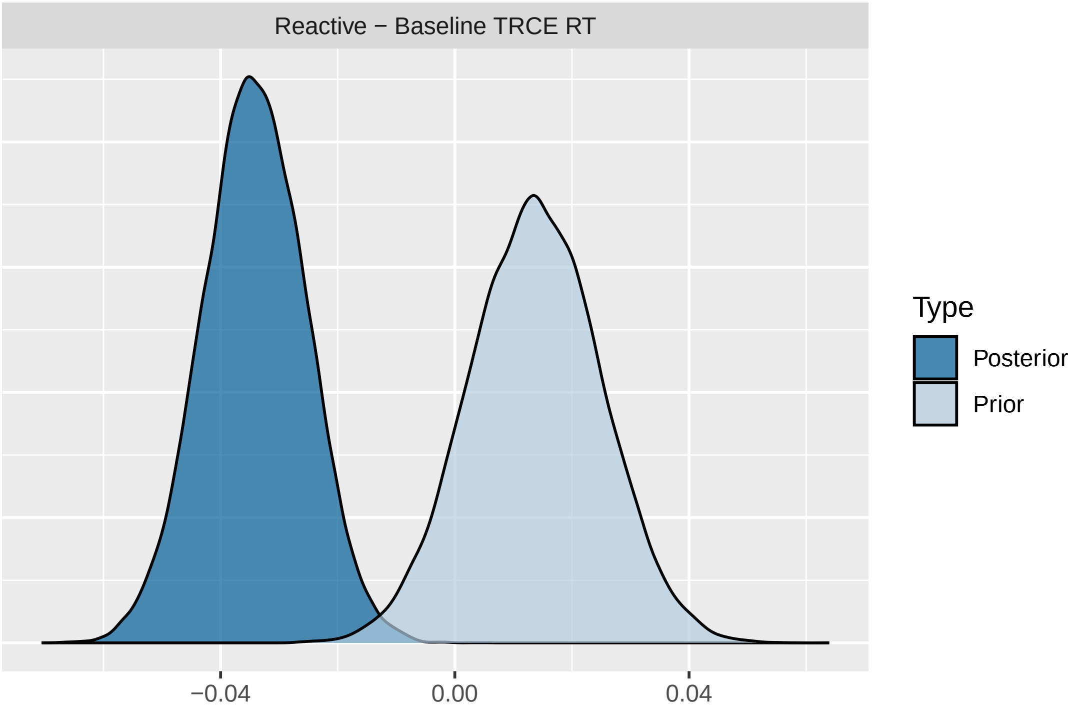

The plot indicates that the posterior is shifted away from the prior distribution. The graph is centered at the mean value of 2018 estimate, and indicates the SDR to be less than one (0) at that point. This suggests that the prior underestimates the reduction in the TRCE RT across the Reactive and Baseline modes.

```
## Bayes Factors for Model Comparison
##
##     Model                                                  BF
## [1] 0 + Intercept + con.id * mode + (1 + con.id | ID) Inf
##
## * Against Denominator: [2] 0 + Intercept + con.id + mode + (1 + con.id | ID)
## *   Bayes Factor Type: marginal likelihoods (bridgesampling)
```

The BF_{10} » 10, indicating strong evidence favoring the inclusion of the interaction term for the Reactive - Baseline model.

```
##                    elpd_diff se_diff
## CuedTS_basrea           0.0       0.0
## CuedTS_basrea_null -8838.4     189.4
```

For LOO-CV, the elpd difference between M_1 (full model) and M_0 (null model) was greater than both four and twice its standard deviation. This indicates that the inclusion of the interaction term for the Reactive-Baseline model meaningfully improved predictive accuracy.

Table 4: Reactive - Baseline TRCE RT (Gaussian)

Term	Estimate	SE	HDI	pd
Baseline Congruent	918.7980	17.9650	[883.56, 954.72]	100%
Baseline Incongruent	70.1947	7.4071	[55.34, 84.32]	100%
Reactive Congruent	15.8845	3.1586	[9.76, 22.14]	100%
Reactive x Incongruent	-32.2487	7.5116	[-46.73, -17.46]	100%

Note. The intercept term 'Baseline Congruent' refers to the RT make a correct response for Baseline congruent trials. 'Reactive x Incongruent' is the key effect and refers to the difference in TRCE RT across the Reactive and Baseline modes.

There was also strong evidence for a reduced TRCE RT in the Reactive versus Baseline mode while assuming a Gaussian distribution (β = -32.25, se = 7.51, HDI = [-46.73, -17.46], pd = 100%). NOTE that this pattern was opposite in direction from that reported in Tang et al (2023).

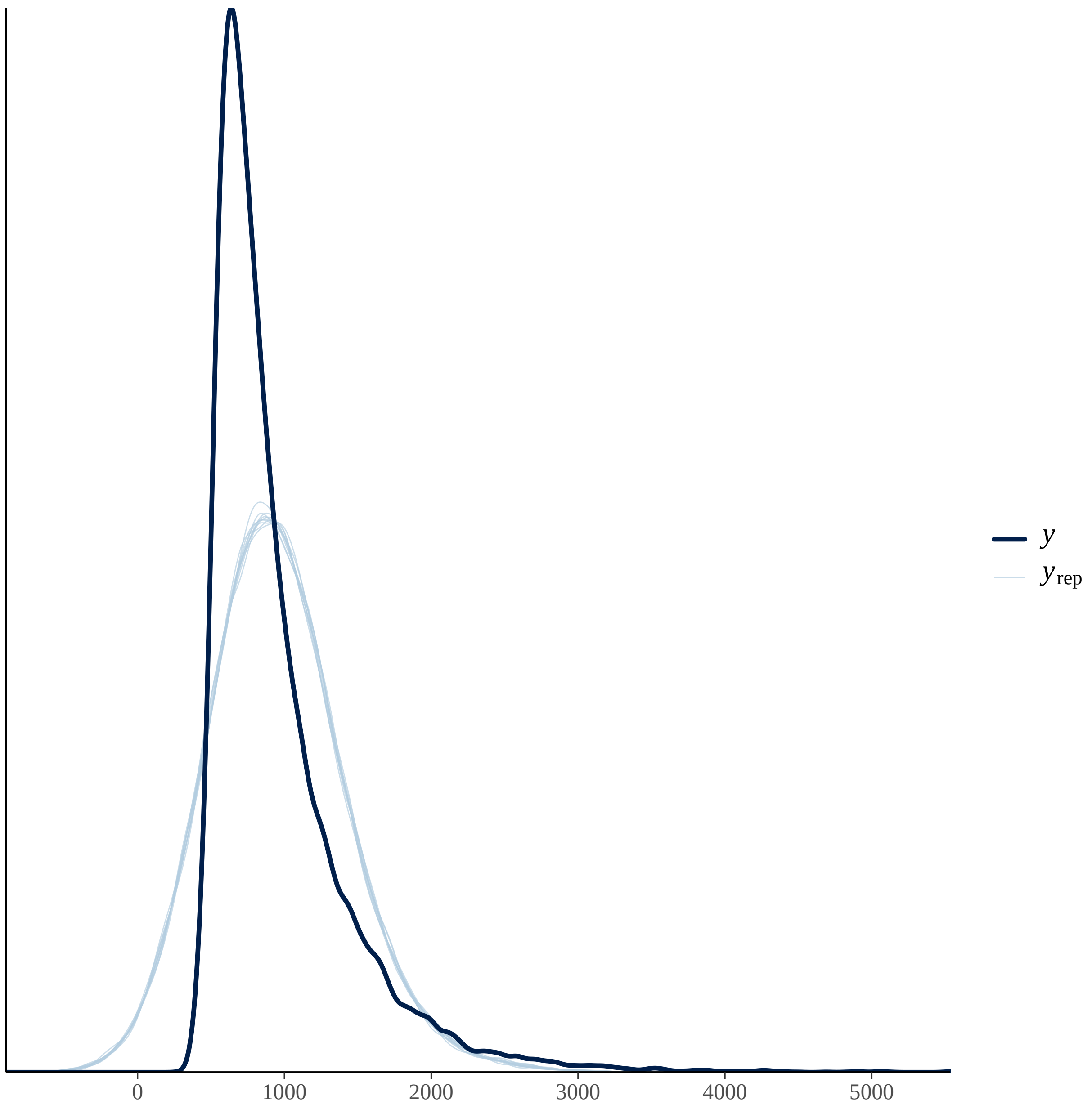
y
y rep
0
1000
2000
3000
4000
5000

Table 5: Reactive - Baseline TRCE RT (ex-Gaussian)

Term	Estimate	SE	HDI	pd
Baseline Congruent	926.1294	7.6642	[911.04, 940.96]	100%
Baseline Incongruent	22.7549	2.7813	[17.4, 28.25]	100%
Reactive Congruent	29.6642	1.4351	[26.83, 32.45]	100%
Reactive x Incongruent	-9.4917	3.3575	[-15.92, -2.8]	100%

Note. The intercept term 'Baseline Congruent' refers to the RT make a correct response for Baseline congruent trials. 'Reactive x Incongruent' is the key effect and refers to the difference in TRCE RT across the Reactive and Baseline modes.

Finally, there was strong evidence for a reduced TRCE RT in the Reactive versus Baseline mode while assuming an ex-Gaussian distribution (β = -9.4917, se = 3.3575, HDI = [-15.92, -2.8], pd = 100%). NOTE that this pattern was opposite in direction from that reported in Tang et al (2023).

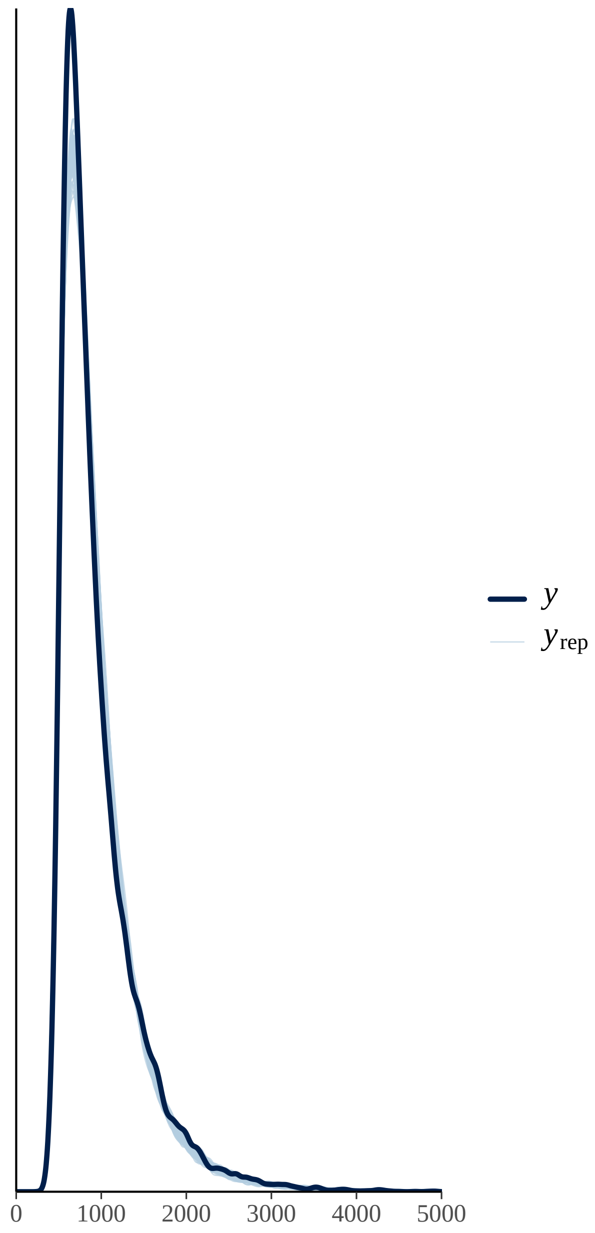
y
y rep
0
1000
2000
3000
4000
5000

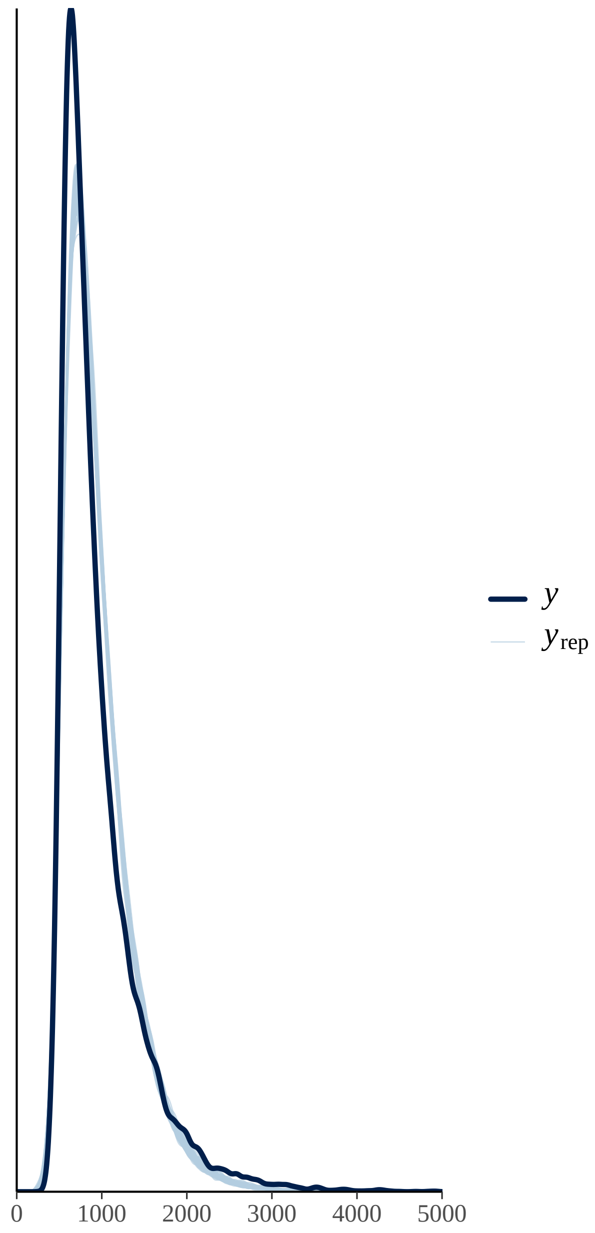
y
y rep
0
1000
2000
3000
4000
5000

```
## Bayes Factors for Model Comparison
##
##     Model                                                       BF
## [1] 0 + Intercept + con.id * mode + (1 + con.id | ID)      Inf
## [2] 0 + Intercept + con.id * mode + (1 + con.id | ID) 0.00e+00
##
## * Against Denominator: [3] 0 + Intercept + con.id * mode + (1 + con.id | ID)
## *   Bayes Factor Type: marginal likelihoods (bridgesampling)
```

For BF model comparison, we define the ex-Gaussian model as M_0, the shifted log-normal model as M_1 and the Gaussian model as M_2. The BF_{10} » 10, indicating strong evidence favoring M_1 (shifted log-normal) over M_0 (ex-Gaussian). In contrast, BF_{20} was BF « 1/10, indicating that M_2 (Gaussian) is a substantially worse fit than M_0.

```
##                       elpd_diff se_diff
## CuedTS_basrea             0.0       0.0
## CuedTS_basrea_exgauss -2209.7      96.9
## CuedTS_basrea_gauss   -8887.8     189.7
```

For LOO-CV, the elpd difference between M_1 (shifted log-normal) and M_0 (ex-Gaussian) / M_2 (Gaussian) were greater than both four and twice their respective standarddeviations. This indicates that M_1 was a substantially better fit than the other models.

5.6.2 Reactive - Proactive TRCE RT

Table 6: Reactive - Proactive TRCE RT (null)

Term	Estimate	SE	HDI	pd
Proactive Congruent	5.9536	0.0231	[5.91, 6]	100%
Proactive Incongruent	0.0896	0.0083	[0.07, 0.11]	100%
Reactive Congruent	0.3877	0.0034	[0.38, 0.39]	100%

Note. The intercept term 'Proactive Congruent' refers to the average [shifted log-normal] RT to make a correct response for Proactive congruent trials.

Table 7: Reactive - Proactive TRCE RT (full)

Term	Estimate	SE	HDI	pd
Proactive Congruent	5.9663	0.0236	[5.92, 6.01]	100%
Proactive Incongruent	0.1000	0.0089	[0.08, 0.12]	100%
Reactive Congruent	0.3818	0.0035	[0.38, 0.39]	100%
Reactive x Incongruent	-0.0365	0.0086	[-0.05, -0.02]	100%

Note. The intercept term 'Proactive Congruent' refers to the average [shifted log-normal] RT to make a correct response for Proactive congruent trials. 'R eactive x Incongruent' is the key effect and refers to the difference in TRCE RT across the Reactive and Proactive modes.

There was also a strongly reduced TRCE RT in the Reactive relative to Proactive mode while assuming a shifted log-normal distribution (β = -0.04, se = 0.01, HDI = [-0.05, -0.02], pd = 100%). The Reactive vs. Proactive contrast was *not* actually reported in Tang et al main text.

Table 8: Hypothesis Test for Rea-Pro TRCE RT

Hypothesis	Estimate	SE	HDI	SDR	Post.Prob
Reactive - Proactive TRCE RT	-0.04	0.01	[-0.05, -0.05]	0.00	0.00

Note. The key term in this table is the SDR score, which is computed as the ratio of the posterior distribution to the prior distribution at a specific point, in this case the mean of the prior distribution to investigate whether there is an increased likelihood of this value as a function of incoming data.

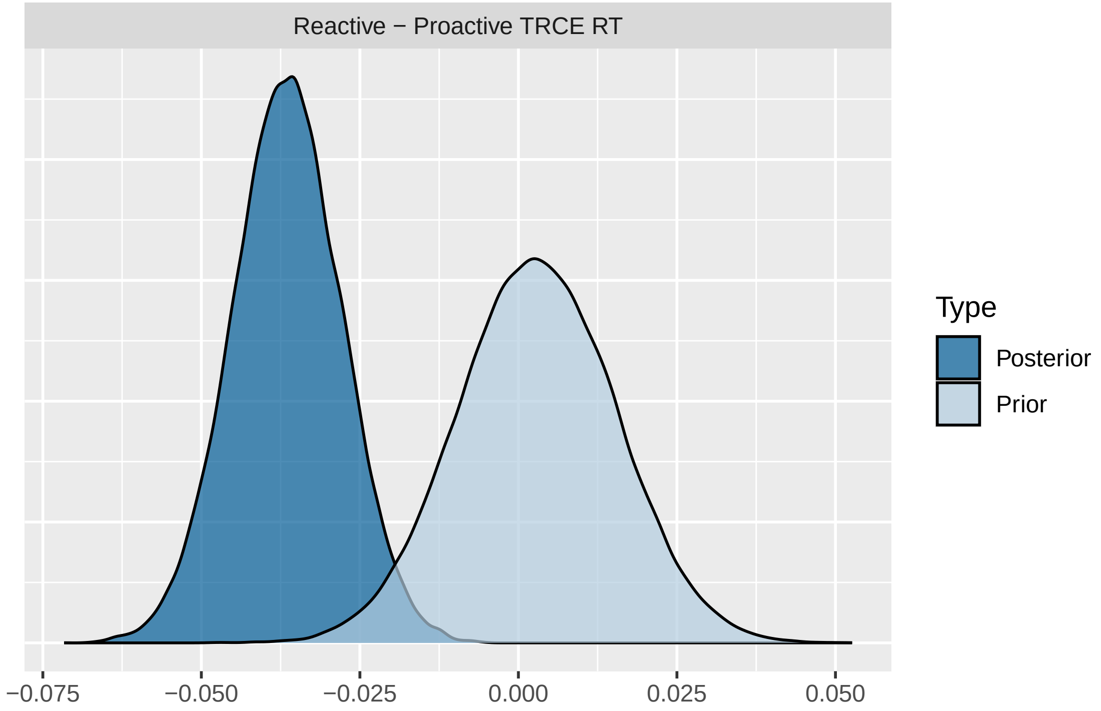

The plot indicates that the posterior is shifted away from the prior distribution. The graph is centered at the mean value of 2018 estimate, and indicates the SDR to be less than one (0) at that point. This suggests that the prior underestimates the reduction in the TRCE RT across the Reactive and Proactive modes, while assuming a shifted log-normal distribution.

```
## Bayes Factors for Model Comparison
##
##     Model                                                    BF
## [1] 0 + Intercept + con.id * mode + (1 + con.id | ID) 2.77e+21
##
## * Against Denominator: [2] 0 + Intercept + con.id + mode + (1 + con.id | ID)
## *   Bayes Factor Type: marginal likelihoods (bridgesampling)
```

The BF_{10} » 10, indicating strong evidence favoring the inclusion of the interaction term for the Reactive - Proactive model.

```
##                     elpd_diff se_diff
## CuedTS_reapro         0.0       0.0
## CuedTS_reapro_null -39.9       4.1
```

For LOO-CV, the elpd difference between M_1 (full model) and M_0 (null model) was bigger than twice its standard deviation. This indicates that the inclusion of the interaction term for the Reactive-Proactive model notably improved predictive accuracy.

Table 9: Reactive - Proactive TRCE RT (Gaussian)

Term	Estimate	SE	HDI	pd
Proactive Congruent	698.8960	13.7696	[671.27, 726.02]	100%
Proactive Incongruent	44.2259	6.3314	[31.94, 56.61]	100%
Reactive Congruent	226.3223	2.6203	[221.3, 231.5]	100%
Reactive x Incongruent	4.1405	6.4661	[-8.4, 16.92]	74.1%

Note. The intercept term 'Proactive Congruent' refers to the average RT to make a correct response for Proactive congruent trials. 'Reactive x Incongruent' is the key effect and refers to the difference in TRCE RT across the Reactive and Proactive modes.

There was little evidence for a difference in the TRCE RT between the Reactive versus Proactive modes while assuming a Gaussian distribution (β = 4.14, se = 6.47, HDI = [-8.4, 16.92], pd = 74.1%).

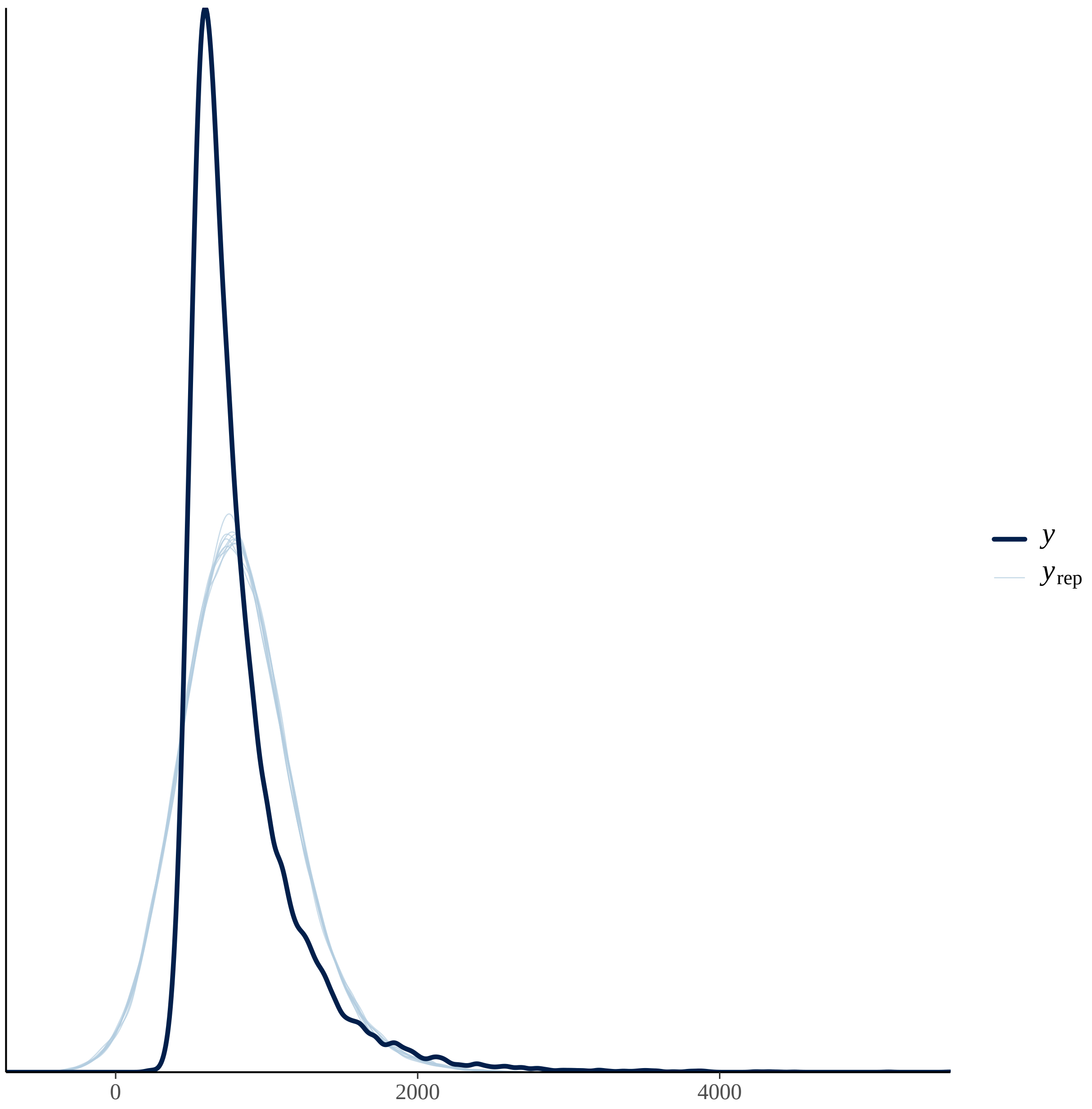

0
2000
4000
y
yrep

Table 10: Reactive - Proactive TRCE RT (ex-Gaussian)

Term	Estimate	SE	HDI	pd
Proactive Congruent	775.9792	6.7890	[763.09, 789.74]	100%
Proactive Incongruent	15.6216	2.4967	[10.77, 20.49]	100%
Reactive Congruent	106.5167	1.2531	[104.15, 109.06]	100%
Reactive x Incongruent	4.2237	2.9399	[-1.56, 9.96]	92.35%

Note. The intercept term 'Proactive Congruent' refers to the average RT to make a correct response for Proactive congruent trials. 'Reactive x Incongruent' is the key TRCE RT and refers to the difference in TRCE RT across the Proactive and Reactive modes.

There was not strong evidence for an increased TRCE RT between the Reactive versus Proactive mode while assuming an ex-Gaussian distribution (β = 4.22, se = 2.94, HDI = [-1.56, 9.96], pd = 92.35%).

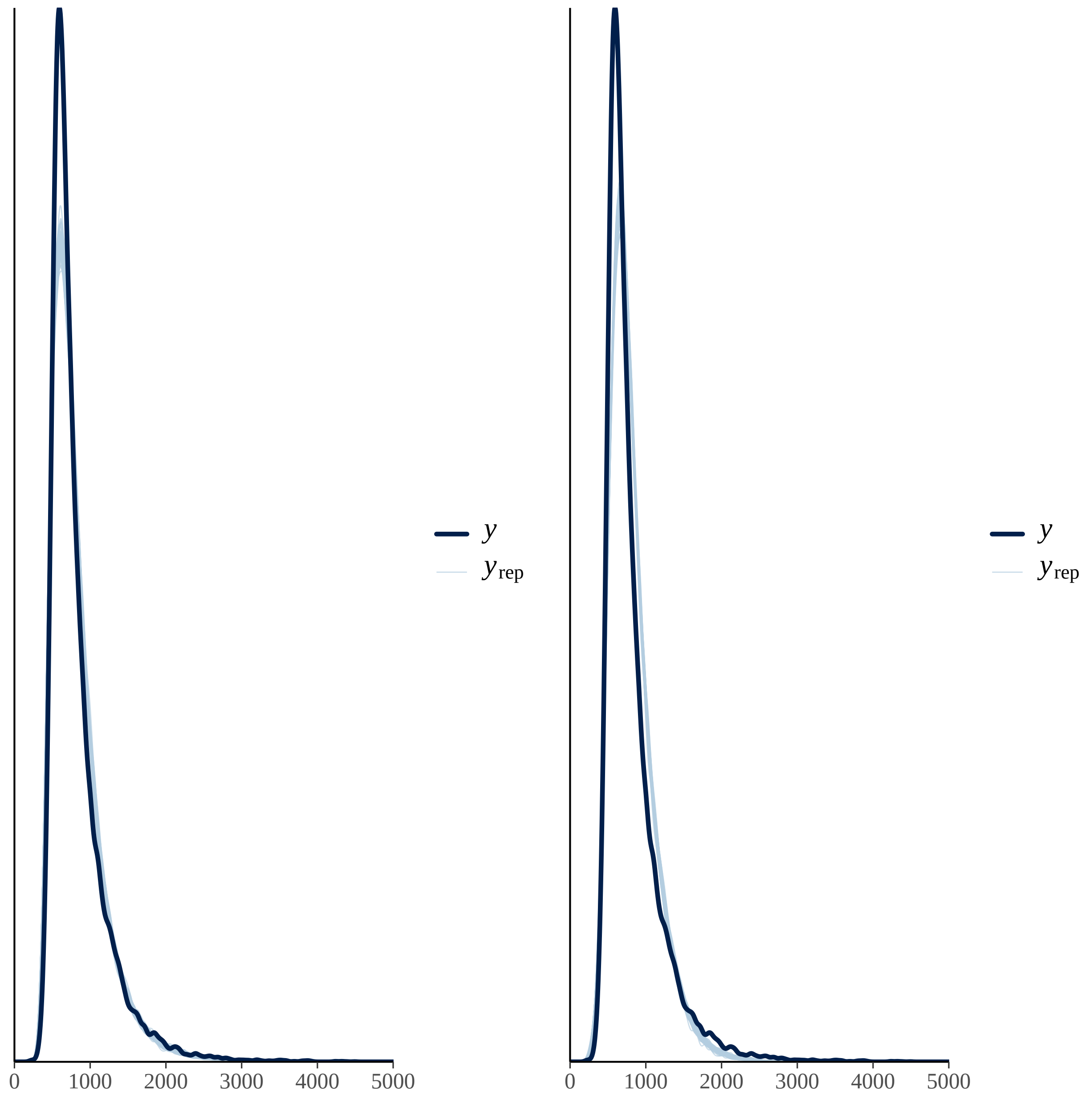

0
1000
2000
3000
4000
5000
y
y_{rep}
0
1000
2000
3000
4000
5000
y
y_{rep}

```
## Bayes Factors for Model Comparison
##
##     Model                                                        BF
## [1] 0 + Intercept + con.id * mode + (1 + con.id | ID)      Inf
## [2] 0 + Intercept + con.id * mode + (1 + con.id | ID) 0.00e+00
##
## * Against Denominator: [3] 0 + Intercept + con.id * mode + (1 + con.id | ID)
## *   Bayes Factor Type: marginal likelihoods (bridgesampling)
```

For BF model comparison, we define the ex-Gaussian model as M_0, the shifted log-normal model as M_1 and the Gaussian model as M_2. The BF_{10} » 10, indicating strong evidence favoring M_1 (shifted log-normal) over M_0 (ex-Gaussian). In contrast, BF_{20} was « 1/10, indicating that M_2 (Gaussian) is a substantially worse fit than M_0.

```
##                       elpd_diff se_diff
## CuedTS_reapro             0.0       0.0
## CuedTS_reapro_exgauss -1878.7     121.2
## CuedTS_reapro_gauss   -8756.7     243.4
```

For LOO-CV, the elpd difference between M_1 (shifted log-normal) and M_0 (ex-Gaussian) / M_2 (Gaussian) was bigger than twice their respective standard deviations. This indicates that M_1 was a substantially better fit than the other models.

5.7 Cued-TS Single Model Output

Based on the Reviewers' request, we also ran a single model of the 2018/2020 Cued-TS data that includes all analyzed predictor levels, and then subsequently extracted relevant contrasts via posterior linear combinations.

5.7.1 2018 Cued-TS Non-Inc Congruent RT Single Model Output

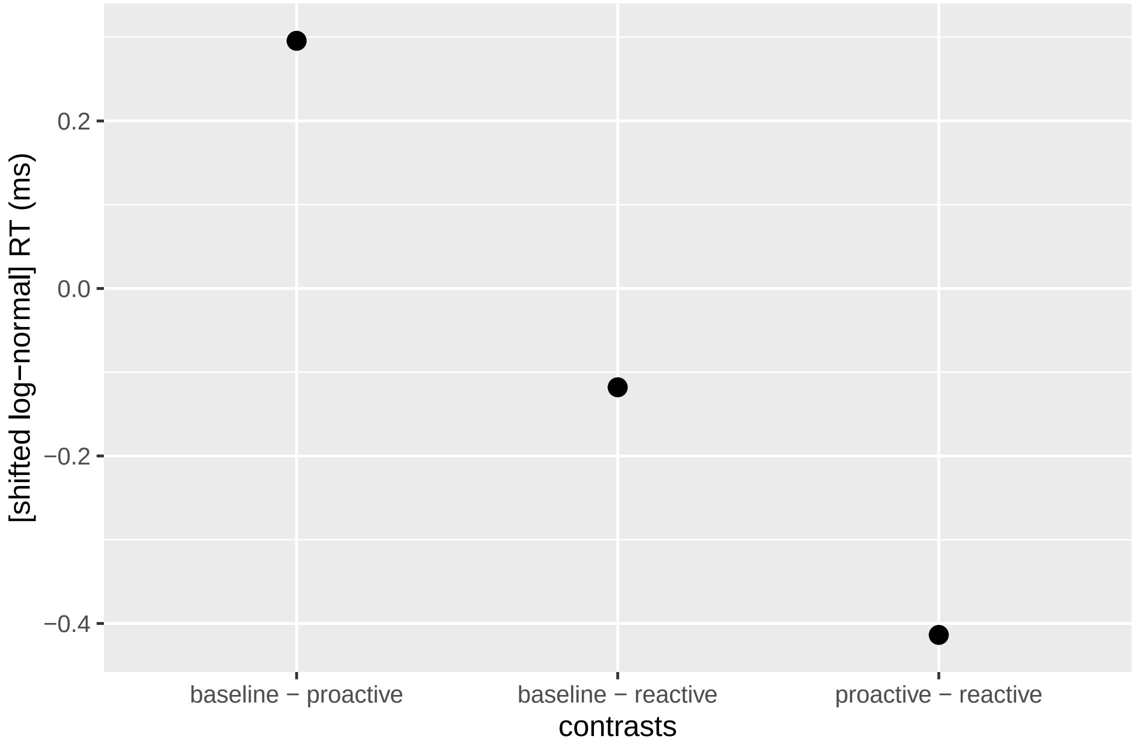

Table 1: Non-Inc Congruent RTs (Reactive vs Baseline/Proactive)

Term	Estimate	HDI	pd
baseline - proactive	0.2957	[0.3, 0.29]	100%
baseline - reactive	-0.1180	[-0.12, -0.13]	100%
proactive - reactive	-0.4137	[-0.41, -0.42]	100%

Note. Each of these terms reflect the contrasting effects of mode. The key effects for the non-incentivized RT effect are 'proactive - baseline' and 'proactive - reactive'. They refer to the difference in the shifted log-normal RT to make a correct response between the Proactive and Baseline/Reactive modes for congruent biased items.

5.7.2 2020 Cued-TS Non-Inc Congruent RT Single Model Output

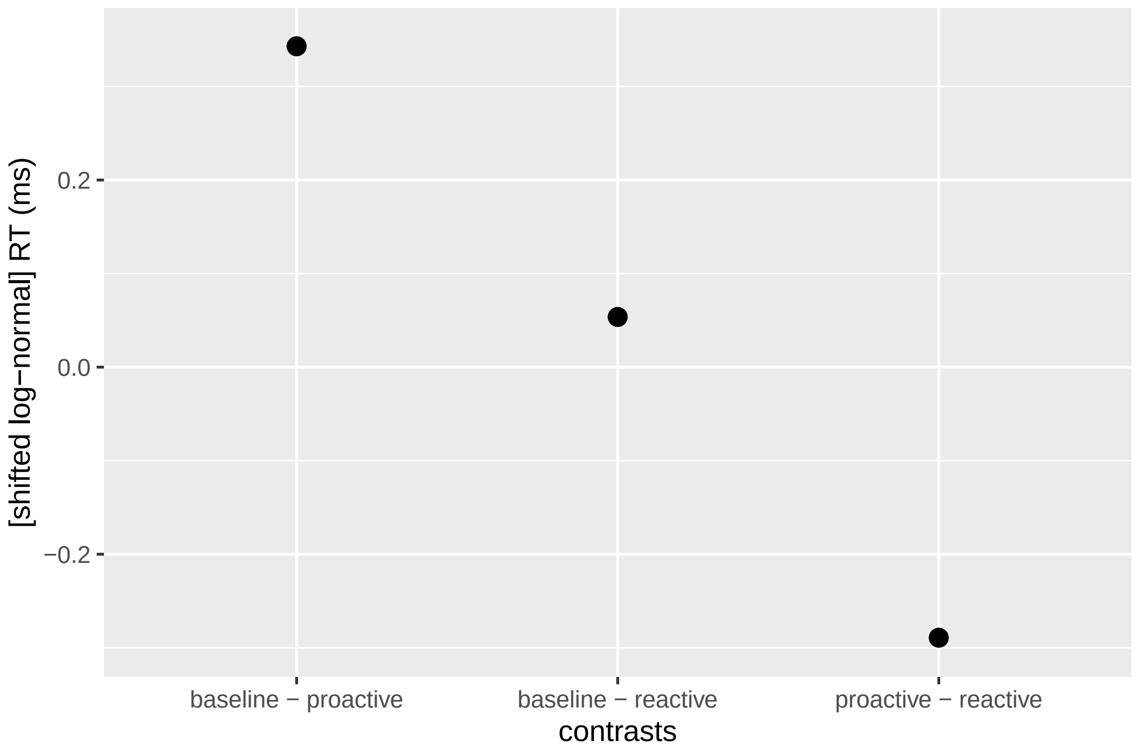

Table 2: Non-Inc Congruent RTs (Reactive vs Baseline/Proactive)

Term	Estimate	HDI	pd
baseline - proactive	0.3430	[0.34, 0.33]	100%
baseline - reactive	0.0536	[0.05, 0.05]	100%
proactive - reactive	-0.2893	[-0.29, -0.3]	100%

Note. Each of these terms reflect the contrasting effects of mode. The key e˙ects for the non-incentivized RT effect are ’proactive - baseline’ and ’proactive - reactive’. They refer to the difference in the shifted log-normal RT to make a correct response between the Proactive and Baseline/Reactive modes for congruentbiased items.

5.7.3 2018 TRCE Error Single Model Output, Logistic

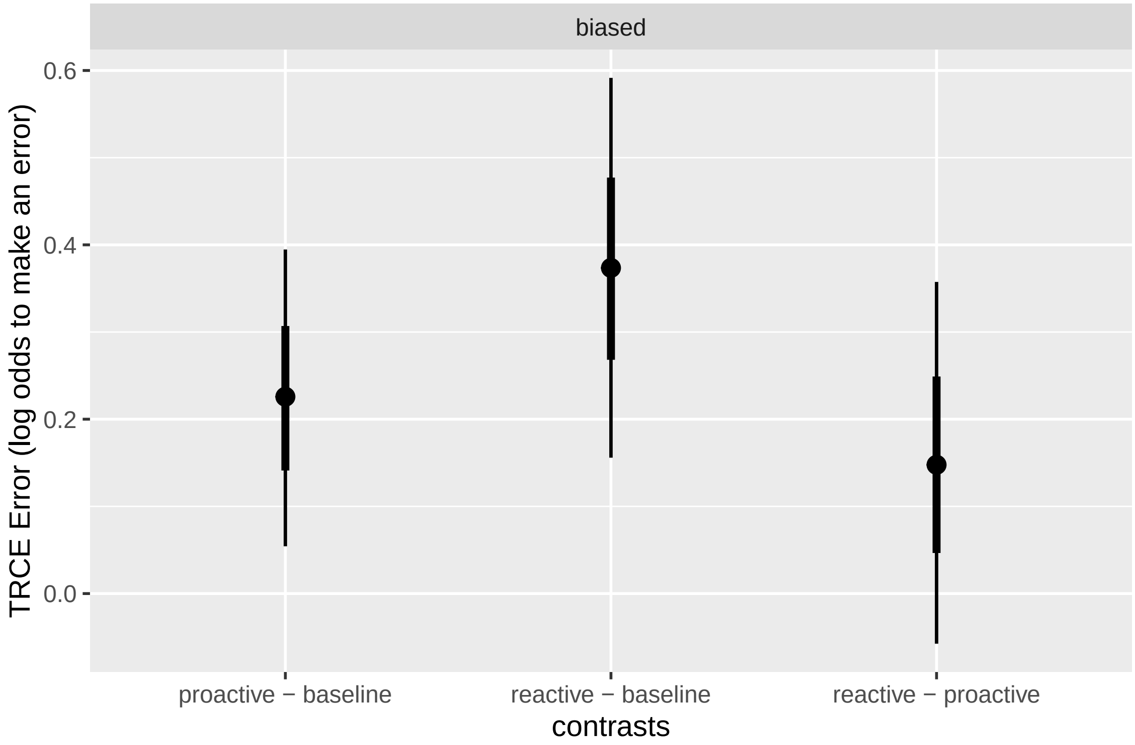

Table 3: TRCE Error (Reactive vs Baseline/Proactive)

Term	Estimate	HDI	pd
proactive - baseline	0.2258	[0.05, 0.4]	99.41%
reactive - baseline	0.3735	[0.15, 0.59]	99.94%
reactive - proactive	0.1477	[-0.06, 0.35]	91.97%

Note. Each of these terms reflect the contrasting effects of mode. The key terms are 'reactive - baseline' and 'reactive - proactive', which refer to the difference in TRCE Error between the Reactive and Proactive/Baseline modes for biased items.

5.7.4 2020 TRCE Error Single Model Output, Logistic

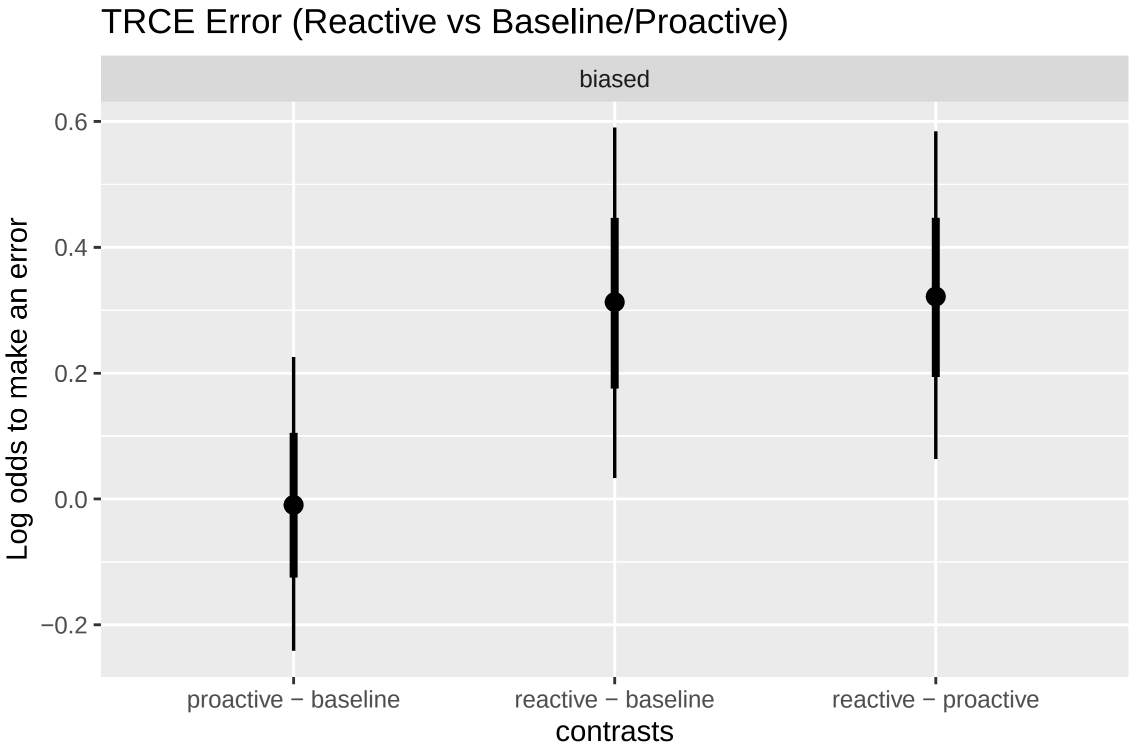

Table 4: TRCE Error (Reactive vs Baseline/Proactive)

Term	Estimate	HDI	pd
proactive - baseline	-0.0095	[-0.25, 0.22]	53.41%
reactive - baseline	0.3129	[0.03, 0.59]	98.54%
reactive - proactive	0.3217	[0.06, 0.59]	99.26%

Note. Each of these terms reflect the contrasting effects of mode. The key terms are ’reactive - baseline’ and ’reactive - proactive’, which refer to the difference in the TRCE Error between the Reactive and Proactive/Baseline modes for biased items.

5.7.5 2018 Cued-TS Error Main Effect Single Model Output, Logistic

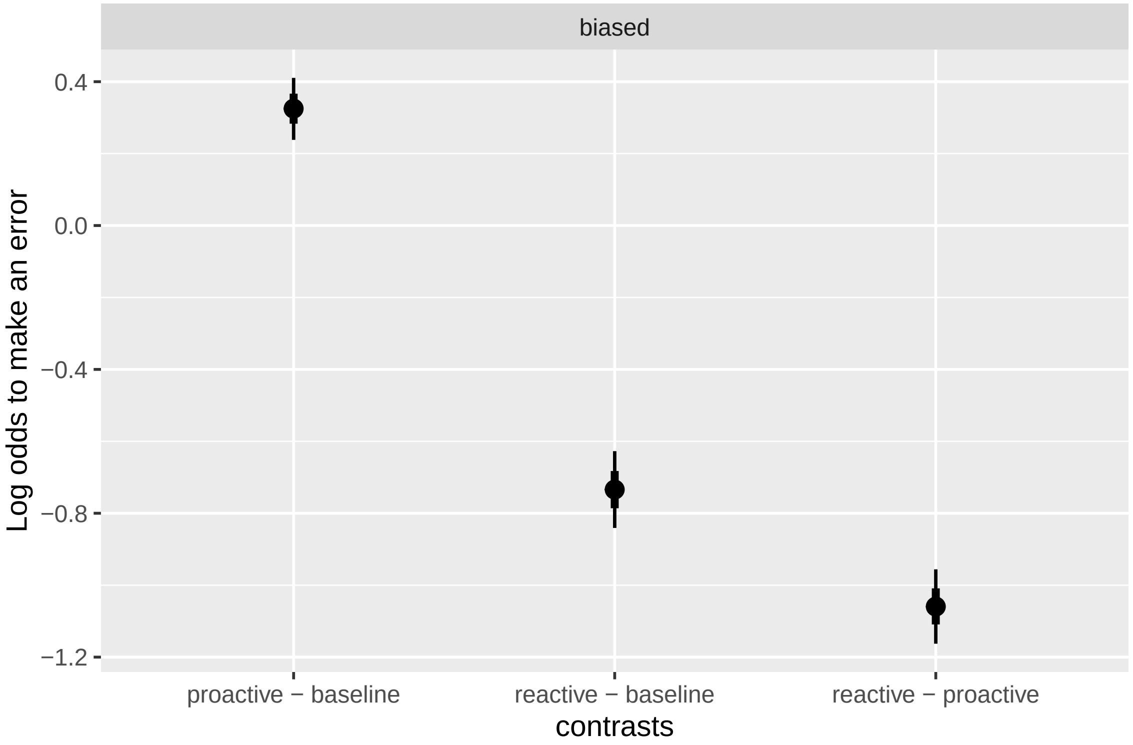

Table 5: Cued-TS Main Effect (Reactive vs Proactive/Baseline)

Term	Estimate	HDI	pd
proactive - baseline	0.3251	[0.24, 0.41]	100%
reactive - baseline	-0.7341	[-0.84, -0.63]	100%
reactive - proactive	-1.0595	[-1.16, -0.96]	100%

Note. Each of these terms reflect the contrasting effects of mode. The key terms are 'reactive - baseline' and 'reactive - proactive', which refer to the difference in the log odds to make an error between the Reactive and Proactive/Baseline modes for biased items, averaged across congruent and incongruent items.

5.7.6 2020 Cued-TS Error Main Effect Single Model Output, logistic

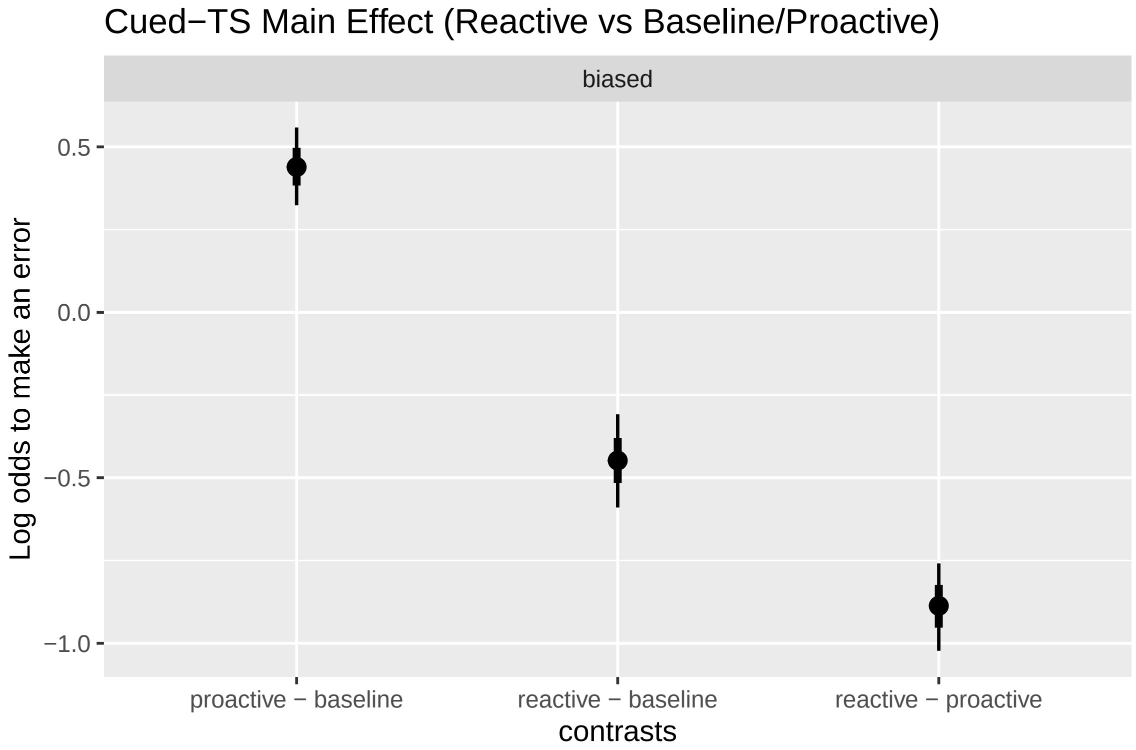

Table 6: Cued-TS Error Main Effect (Reactive vs Proactive/Baseline)

Term	Estimate	HDI	pd
proactive - baseline	0.4390	[0.32, 0.56]	100%
reactive - baseline	-0.4478	[-0.59, -0.31]	100%
reactive - proactive	-0.8868	[-1.02, -0.76]	100%

Note. Each of these terms reflect the contrasting effects of mode. The key terms are 'reactive - baseline' and 'reactive - proactive', which refer to the di˙erence in the log odds to make an error between the Reactive and Proactive/Baseline modes for biased items, averaged across congruent and incongruent items.

5.7.7 2018 Cued-TS Error Incongruent Effect Single Model Output, Logistic

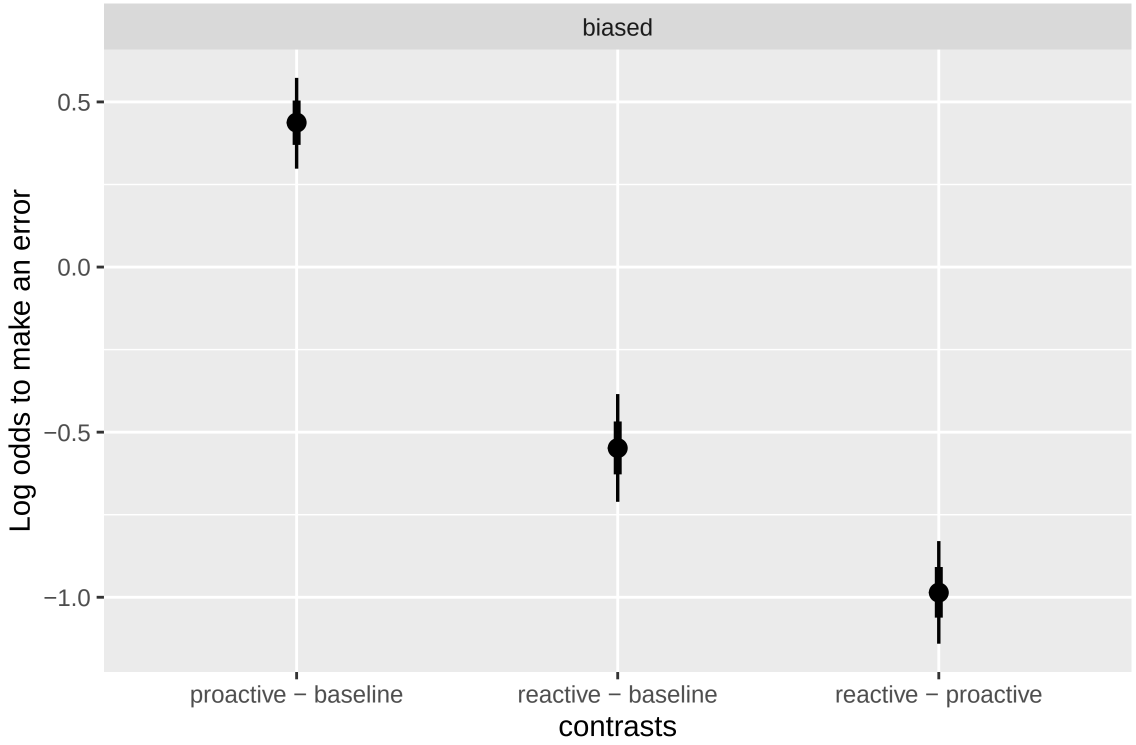

Table 7: Cued-TS Error Incongruent Effect (Reactive vs Baseline/Proactive)

Term	Estimate	HDI	pd
proactive - baseline	0.4374	[0.3, 0.58]	100%
reactive - baseline	-0.5483	[-0.71, -0.39]	100%
reactive - proactive	-0.9858	[-1.14, -0.83]	100%

Note. Each of these terms reflect the contrasting effects of mode for incongruent items. The key terms are 'reactive - baseline' and 'reactive - proactive', which refer to the difference in the log odds to make an error between the Reactive and Proactive/Baseline modes for biased items, specifically for incongruent items.

5.7.8 2020 Cued-TS Error Incongruent Effect Single Model Output, logistic

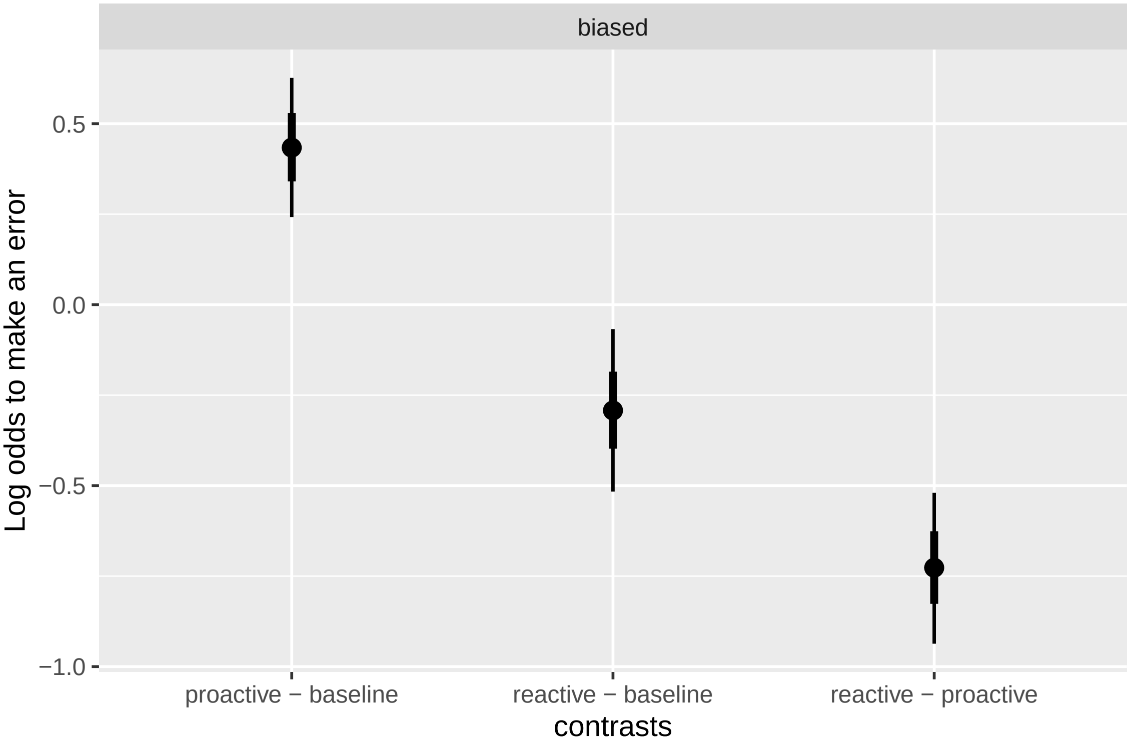

Table 8: Cued-TS Error Incongruent Effect (Reactive vs Baseline/Proactive)

Term	Estimate	HDI	pd
proactive - baseline	0.4337	[0.24, 0.62]	100%
reactive - baseline	-0.2924	[-0.52, -0.07]	99.5%
reactive - proactive	-0.7269	[-0.93, -0.52]	100%

Note. Each of these terms reflect the contrasting effects of mode for incongruent items. The key terms are 'reactive - baseline' and 'reactive - proactive', which refer to the difference in the log odds to make an error between the Reactive and Proactive/Baseline modes for biased items, specifically for incongruent items.

5.7.9 2018 TRCE Error Single Model Output, Error Rates

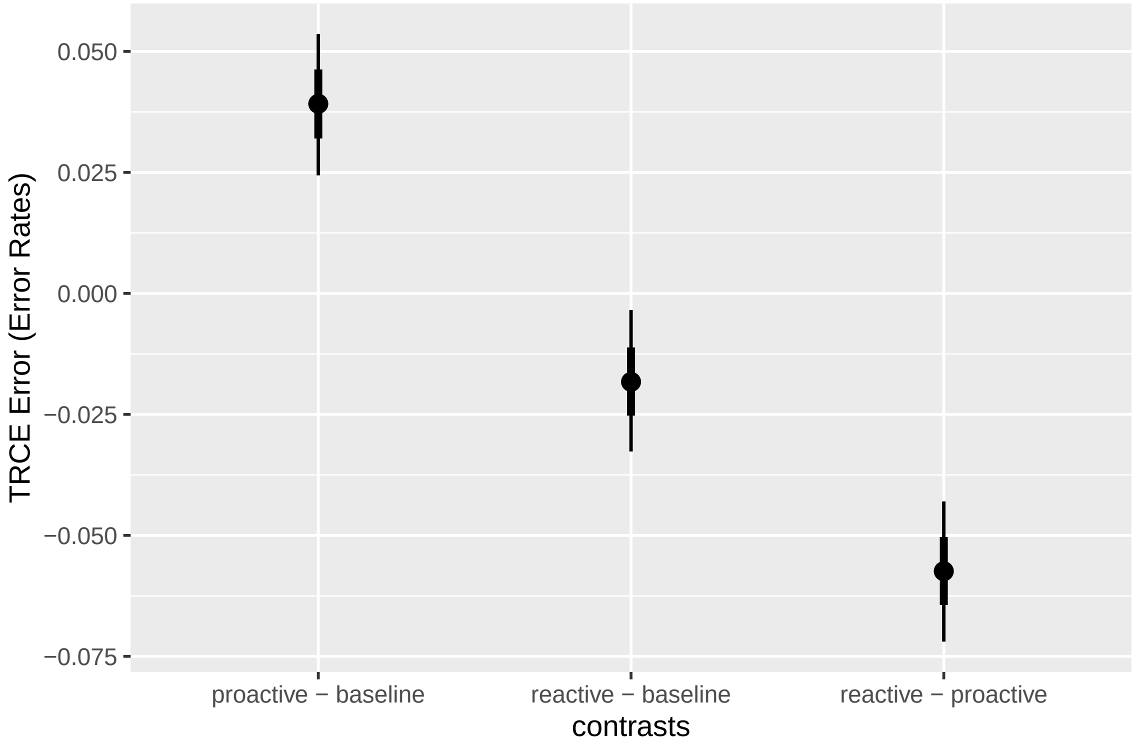

Table 9: TRCE Error Rates (Reactive vs Baseline/Proactive)

Term	Estimate	HDI	pd
proactive - baseline	0.0392	[0.02, 0.05]	100%
reactive - baseline	-0.0183	[-0.03, 0]	99.31%
reactive - proactive	-0.0574	[-0.07, -0.04]	100%

Note. Each of these terms reflect the contrasting effects of mode. The key terms are 'reactive - baseline' and 'reactive - proactive', which refer to the difference in the TRCE Error between the Reactive and Proactive/Baseline modes for biased items.

5.7.10 2020 TRCE Error Single Model Output, Error Rates

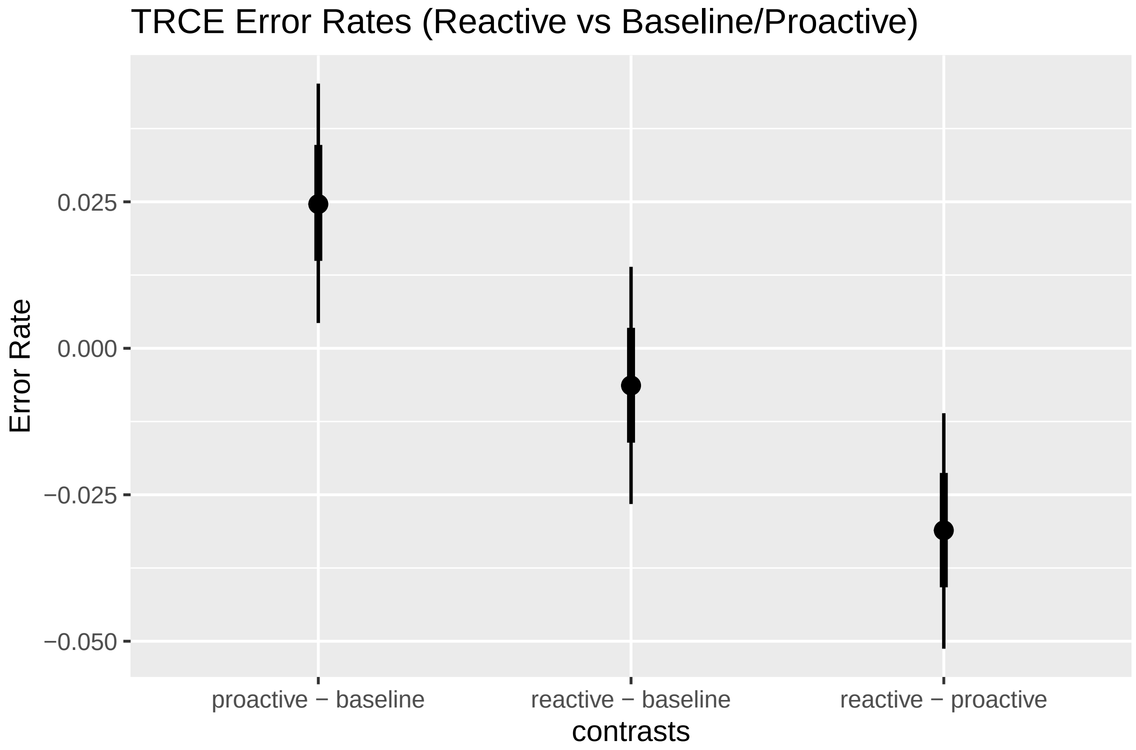

Table 10: TRCE Error Rates (Reactive vs Baseline/Proactive)

Term	Estimate	HDI	pd
proactive - baseline	0.0246	[0, 0.05]	99.12%
reactive - baseline	-0.0064	[-0.03, 0.01]	73.42%
reactive - proactive	-0.0311	[-0.05, -0.01]	99.82%

Note. Each of these terms reflect the contrasting e˙ects of mode. The key terms are 'reactive - baseline' and 'reactive - proactive', which refer to the difference in TRCE Error between the Reactive and Proactive/Baseline modes for biased items.

5.7.11 2018 Cued-TS Main Effect Single Model Output, Error Rates

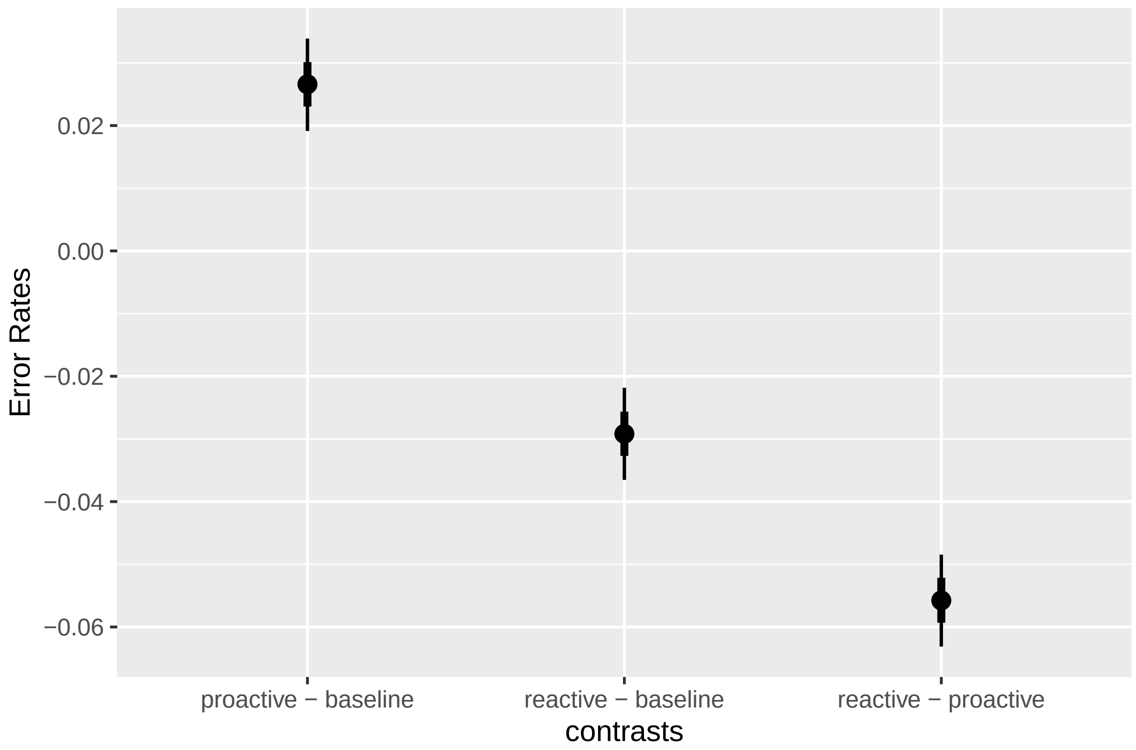

Table 11: Cued-TS Main Effect (Reactive vs Proactive/Baseline)

Term	Estimate	HDI	pd
proactive - baseline	0.0266	[0.02, 0.03]	100%
reactive - baseline	-0.0292	[-0.04, -0.02]	100%
reactive - proactive	-0.0558	[-0.06, -0.05]	100%

Note. Each of these terms reflect the contrasting e˙ects of mode. The key terms are 'reactive - baseline' and 'reactive - proactive', which refer to the difference in the error rates between the Reactive and Proactive/Baseline modes for biased items, averaged across congruent and incongruent items.

5.7.12 2020 Cued-TS Error Main Effect Single Model Output, Error Rates

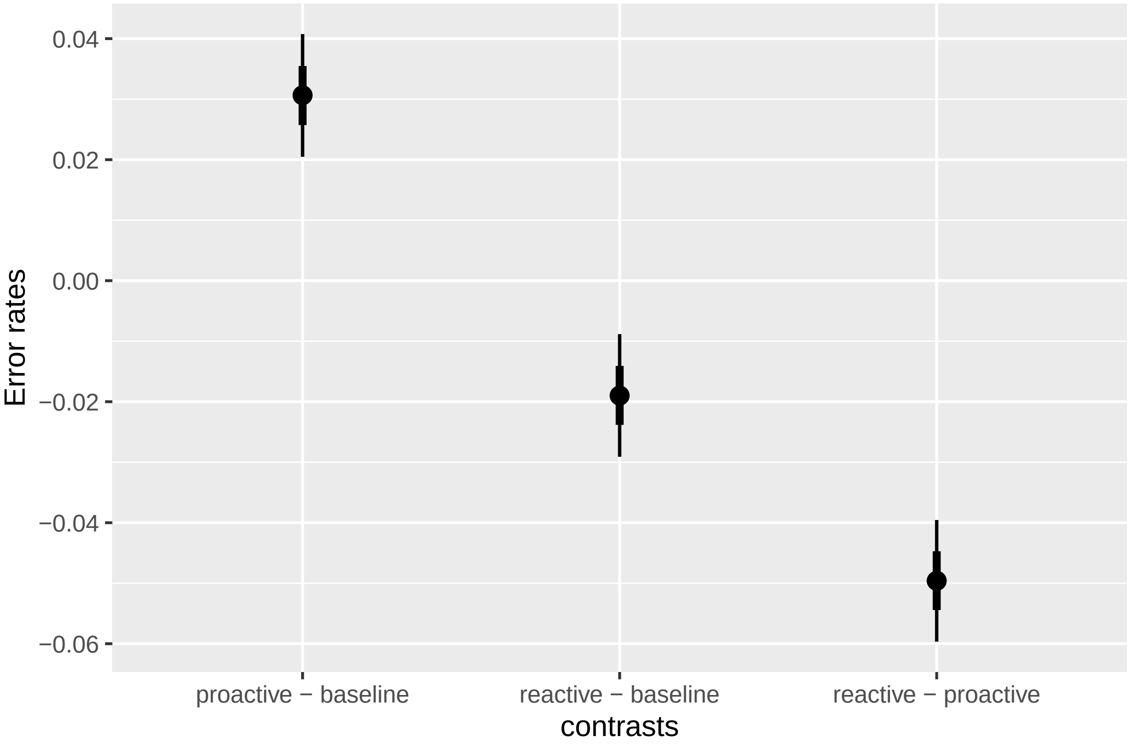

Table 12: Cued-TS Error Main Effect (Reactive vs Proactive/Baseline)

Term	Estimate	HDI	pd
proactive - baseline	0.0306	[0.02, 0.04]	100%
reactive - baseline	-0.0190	[-0.03, -0.01]	100%
reactive - proactive	-0.0496	[-0.06, -0.04]	100%

Note. Each of these terms reflect the contrasting effects of mode. The key terms are 'reactive - baseline' and 'reactive - proactive', which refer to the di˙erence in the error rates between the Reactive and Proactive/Baseline modes for biased items, averaged across congruent and incongruent items.

5.7.13 2018 Cued-TS Incongruent Effect Single Model Output, Error Rates

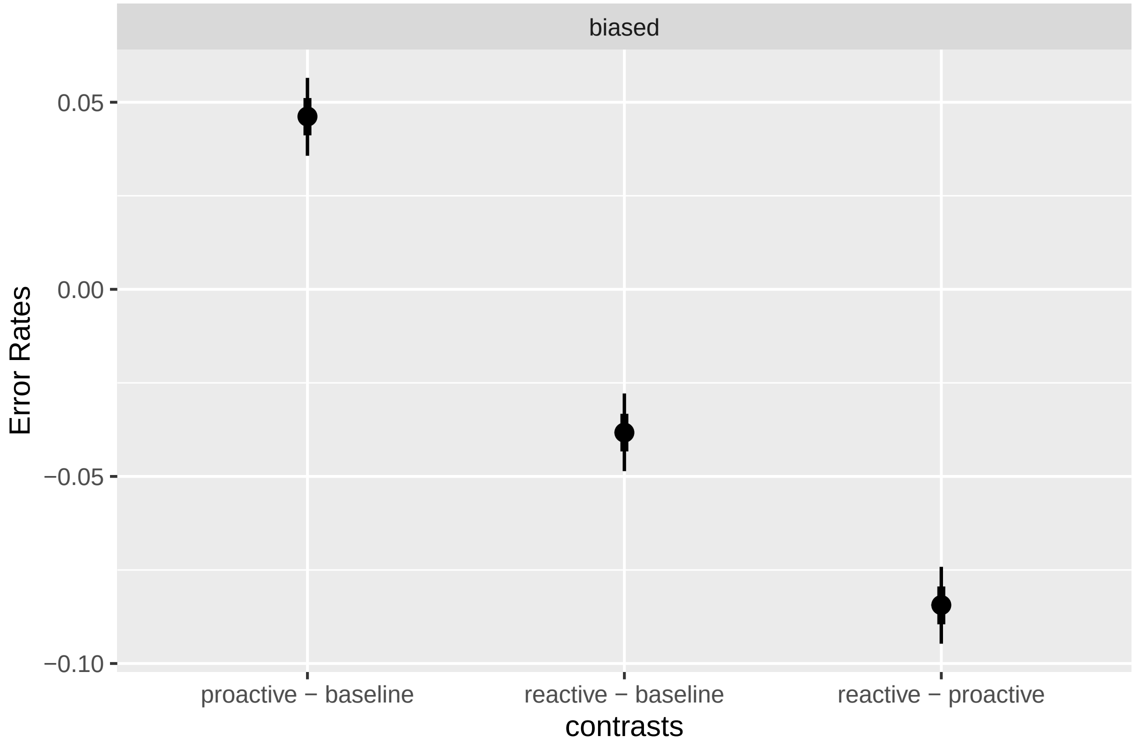

Table 13: Cued-TS Error Incongruent Effect (Reactive vs Baseline/Proactive)

Term	Estimate	HDI	pd
proactive - baseline	0.0462	[0.04, 0.06]	100%
reactive - baseline	-0.0383	[-0.05, -0.03]	100%
reactive - proactive	-0.0844	[-0.09, -0.07]	100%

Note. Each of these terms reflect the contrasting effects of mode for incongruent items. The key terms are 'reactive - baseline' and 'reactive - proactive', which refer to the difference in the error rates between the Reactive and Proactive/Baseline modes for biased items, specifically for incongruent items.

5.7.14 2020 Cued-TS Error Incongruent Effect Single Model Output, Error Rates

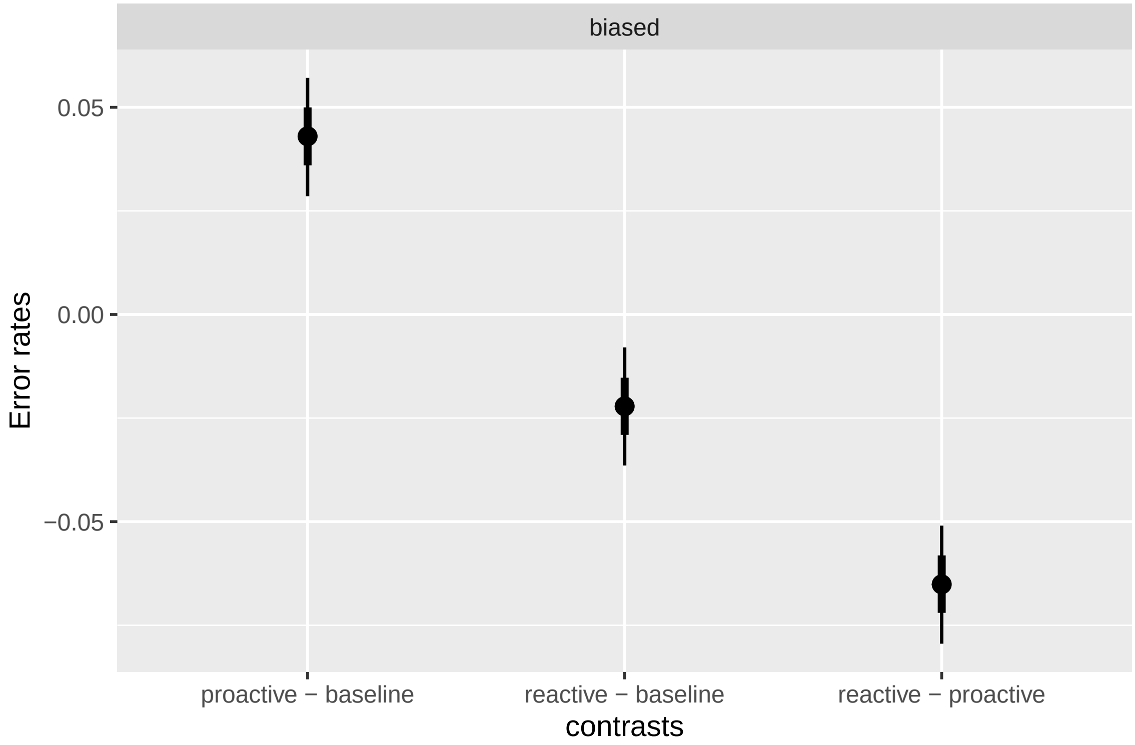

Table 14: Cued-TS Error Incongruent Effect (Reactive vs Baseline/Proactive)

Term	Estimate	HDI	pd
proactive - baseline	0.0430	[0.03, 0.06]	100%
reactive - baseline	-0.0222	[-0.04, -0.01]	99.87%
reactive - proactive	-0.0651	[-0.08, -0.05]	100%

Note. Each of these terms reflect the contrasting effects of mode for incongruent items. The key terms are 'reactive - baseline' and 'reactive - proactive', which refer to the difference in the error rates between the Reactive and Proactive/Baseline modes for biased items, specifically for incongruent items.

5.7.15 2018 TRCE RT Single Model Output

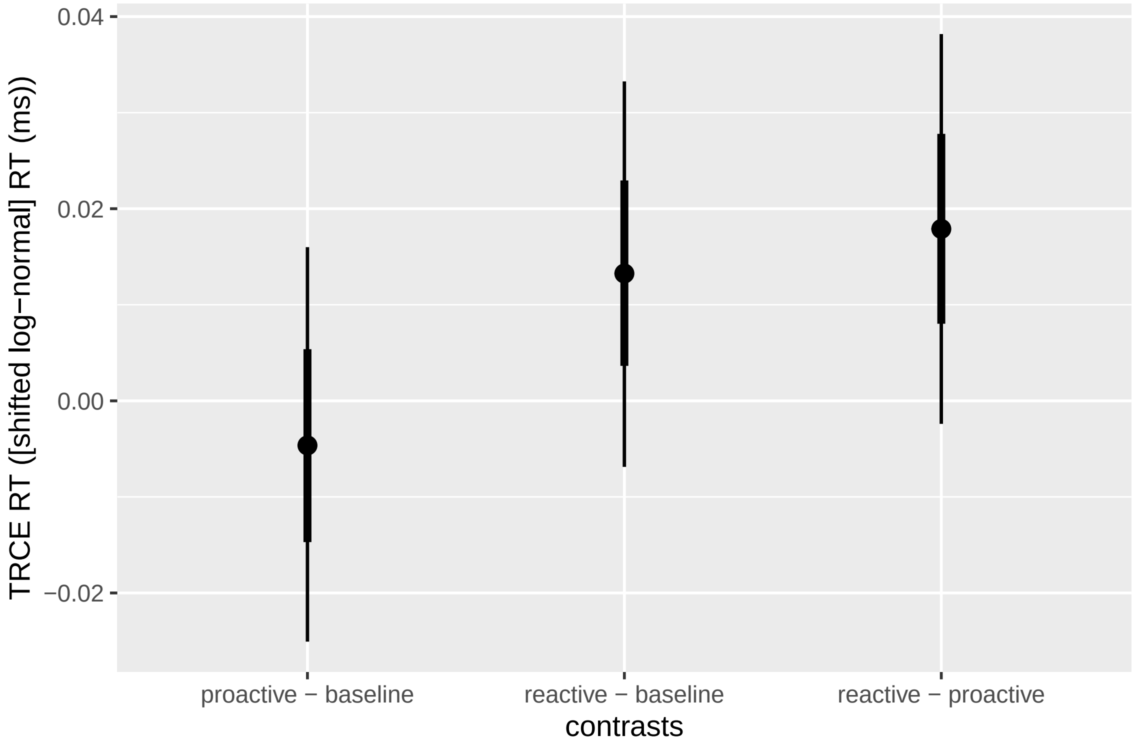

Table 15: TRCE RT (Reactive vs Baseline/Proactive)

Term	Estimate	HDI	pd
proactive - baseline	-0.0046	[0, -0.03]	67.32%
reactive - baseline	0.0133	[0.01, -0.01]	90.3%
reactive - proactive	0.0179	[0.02, 0]	95.76%

Note. Each of these terms reflect the contrasting effects of mode. The key effects are 'reactive - baseline' and 'reactive - proactive'. They refer to the difference in the TRCE RT between the Reactive and Proactive/Baseline modes for biased items.

5.7.16 2020 TRCE RT Single Model Output

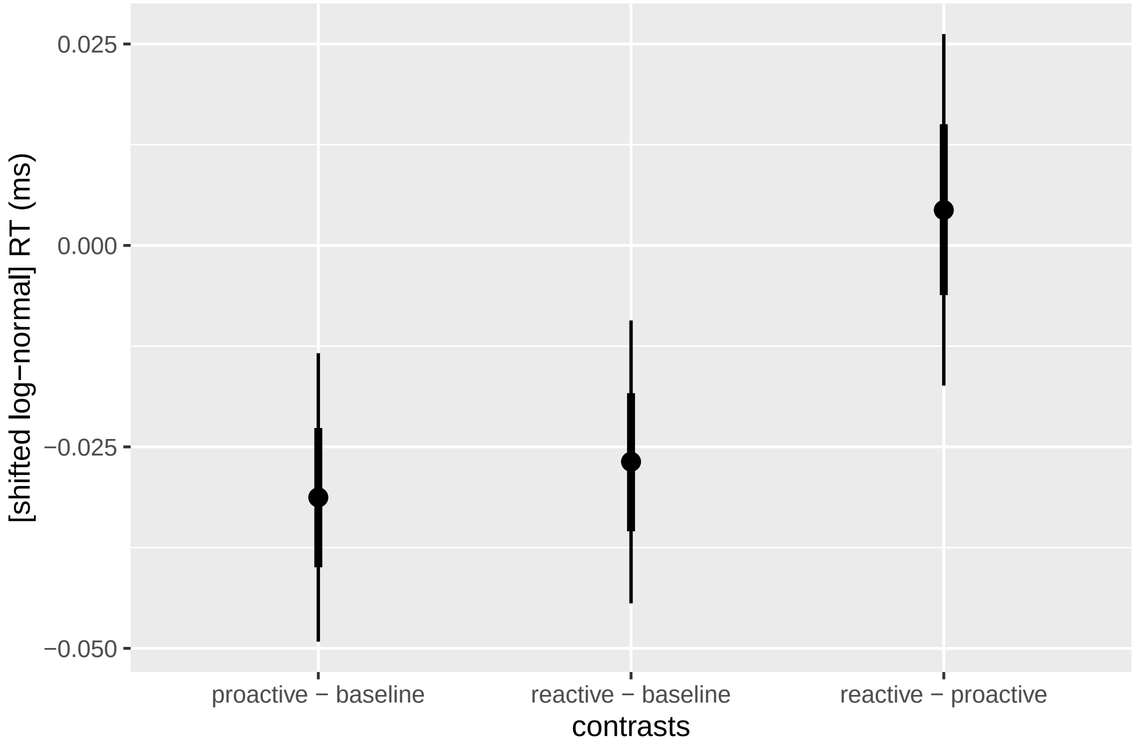

Table 16: TRCE RT (Reactive vs Baseline/Proactive)

Term	Estimate	HDI	pd
proactive - baseline	-0.0313	[-0.03, -0.05]	99.99%
reactive - baseline	-0.0268	[-0.03, -0.04]	99.84%
reactive - proactive	0.0044	[0, -0.02]	65.63%

Note. Each of these terms reflect the contrasting effects of mode. The key effects are 'reactive - baseline' and 'reactive - proactive'. They refer to the difference in the TRCE RT between the Reactive and Proactive/Baseline modes for biased items.
